# Lsp2 links early-life diet to adult translation and lifespan in *Drosophila*

**DOI:** 10.1038/s41586-026-11031-3

**Published:** 2026-09-23

**Authors:** Hina Kosakamoto, Rina Okada, Clive S. Barker, Ayako Isomura-Matoba, Jun Seita, Naoshi Dohmae, Koshi Imami, Fumiaki Obata

**Affiliations:** 1 Laboratory for Nutritional Biology, RIKEN Center for Biosystems and Dynamics Research, Kobe, Japan; 2 Laboratory for Integrative Genomics, RIKEN Center for Integrative Medical Sciences, Yokohama, Japan; 3 Biomolecular Characterization Unit, RIKEN Center for Sustainable Resource Science, Wako, Japan; 4 Proteome Homeostasis Research Unit, RIKEN Center for Integrative Medical Sciences, Yokohama, Japan; 5 Laboratory of Molecular Cell Biology and Development, Graduate School of Biostudies, Kyoto University, Kyoto, Japan; 6 Present Address: Champalimaud Foundation, Lisbon, Portugal

**Keywords:** Ageing, Metabolism

## Abstract

Nearly a century ago, restricting diet during early-life periods was suggested to extend lifespan in rats and in *Daphnia*^1,2^. The effect of juvenile diet on adult physiology and lifespan has subsequently been described in other model organisms, including fruit flies^3–5^ and mice^6–8^; however, its mechanism remains poorly understood. Here, using *Drosophila* as a model, we show that restricting protein intake during the larval stage (early-life protein restriction; ePR) promotes adult lifespan by reducing the levels of storage proteins. Using stable-isotope tracing, we show that dietary amino acids obtained in the larval stage are retained into early adulthood, and are incorporated into ribosomal proteins in particular. This is mediated by larval serum protein 2 (Lsp2), a major storage protein, the expression of which is durably downregulated by ePR in the early adult stage. Genetic silencing of *Lsp2* phenocopies ePR, attenuating ribosomal-protein abundance and translational activity in early adulthood, and extending lifespan. Restricting specific amino acids that are especially enriched in these storage proteins, such as phenylalanine and tyrosine, is sufficient to decrease the levels of early-life Lsp2 and promote longevity. These findings identify Lsp2 as a molecular carrier of nutritional history across developmental transitions, linking juvenile nutritional status to adult translational capacity and lifespan. Our study uncovers a previously unrecognized mechanism of nutritional memory that links early-life diet to lifelong organismal health.

## Abstract (web summary)

Isotope tracing is used to determine the fate of larval dietary amino acids in adult flies, and identifies Lsp2 as a key regulator of translation and lifespan that forms the molecular basis of the effects of early-life protein restriction.

## Main

Dietary restriction is a robust intervention that delays ageing and extends lifespan across diverse species. Transient nutritional manipulations not only during adulthood but also during early life stages can have long-lasting effects on adult physiology and lifespan, which can be positive or negative depending on the context^4,5,7,9,10^. This phenomenon, often referred to as nutritional programming, has been observed across taxa^11,12^. Several hypotheses have been proposed to explain this programming, including irreversible changes in organ structure; epigenetic modifications; trade-offs between growth, reproduction and somatic maintenance; metabolic and endocrine homeostasis; or gut microbiota^13–17^.

*Drosophila* has emerged as a powerful model for dissecting the mechanisms that underlie the effects of early-life dietary restriction. Although some reports suggest that larval yeast restriction produces adults that are small but that have a normal lifespan^18^, other studies provide evidence of considerable lifespan extension, depending on sex and the adult environment^4,5,19^. At the molecular level, transcriptomic shifts in ribosomal and translational machinery^19^ or altered production of lipid autotoxins (cuticular hydrocarbons)^5^ have been observed in long-lived adults. But so far, the molecular entity that encodes, preserves and transmits early-life nutritional information across developmental transitions has not been identified.

## Early-life protein restriction extends lifespan

**Fig. 1:**
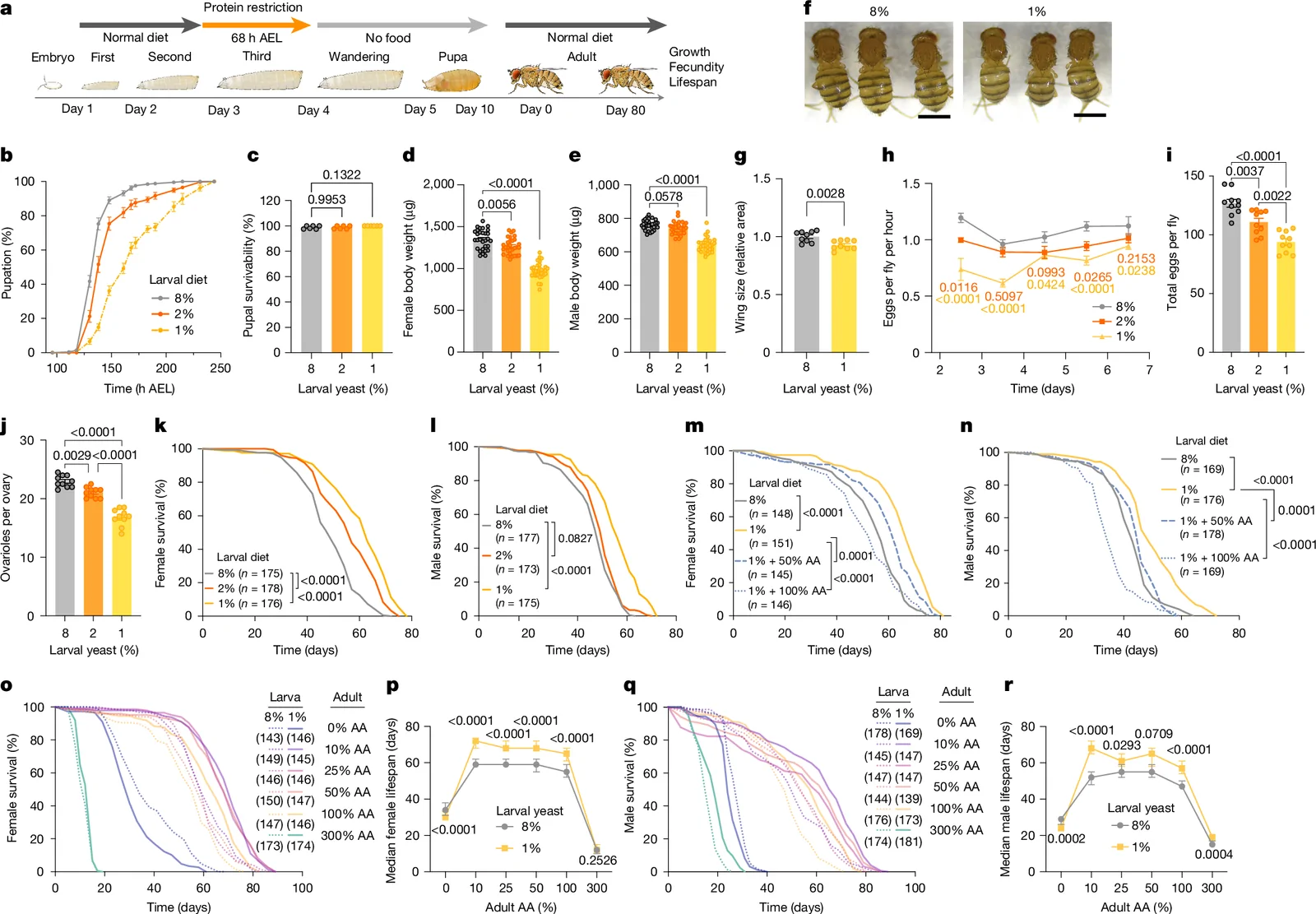
Effects of larval diet on development, physiology and lifespan. **a**, Schematic representation of the ePR protocol. **b**,**c**, Developmental duration (**b**) and eclosion rate (**c**) of Canton-S flies. *n* = 6. **d**,**e**, Body weight of Canton-S female (**d**) and male (**e**) flies at adult day 1. *n* = 30. **f**, Representative images of Canton-S females at adult day 1. Scale bars, 1 mm. **g**, Wing size of Canton-S female flies. *n* = 9. **h**,**i**, Daily fecundity (**h**) and cumulative egg production (**i**) of Canton-S females at adult day 2–7. *n* = 10. **j**, Number of ovarioles per ovary in Canton-S female flies at day 4. *n* = 10. **k**,**l**, Lifespan of Canton-S female (**k**) and male (**l**) flies. **m**,**n**, Lifespan of Canton-S female (**m**) and male (**n**) flies after ePR with or without amino acid (AA) supplementation in the larval diet. **o**–**r**, Lifespan curve (**o**,**q**) and corresponding median lifespan (**p**,**r**) of Canton-S female (**o**,**p**) or male (**q**,**r**) flies. The ePR-flies are fed with a synthetic diet of varying amino acid concentrations at the adult stage. Error bars in **p**,**r** represent 95% confidence intervals. Sample sizes (*n*) for lifespan experiments are given. Data are mean ± s.e.m. in **b**–**j**. Symbols represent biological replicates. Statistical significance was determined by one-way ANOVA with Dunnett’s multiple comparison test (**c**–**e**), two-tailed Student’s *t*-test (**g**), two-way ANOVA with Dunnett’s multiple comparison test (**h**), one-way ANOVA with Holm–Šídák’s multiple comparison test (**i**,**j**) or log-rank test (**k**–**r**). Source data

To establish an experimental set-up for elucidating the mechanisms of nutritional memory, we implemented dietary manipulation exclusively during the larval stage. Because yeast serves as the major dietary source of amino acids and protein for *Drosophila* under standard laboratory conditions, we manipulated yeast concentration during larval development to induce transient early-life protein restriction (ePR). Specifically, late-second-instar larvae were transferred to a low-yeast diet approximately 68 h after egg-laying (AEL). After eclosion, adult flies were returned to a standard yeast-based diet (Fig. 1a). We first assessed the effects of ePR on developmental timing, eclosion rate and systemic growth (Fig. 1b–e). Reducing the yeast content in the larval diet from 8% to 2% or 1% delayed development (Fig. 1b); however, once larvae reached the pupal stage, the eclosion rate was unaffected (Fig. 1c). Protein restriction decreased adult body weight in a dose-dependent manner in both sexes (Fig. 1d,e), with female flies exhibiting a reduction of up to 28% (Fig. 1d,f). Wing size, a reliable indicator of overall body growth, was significantly decreased, but only by 7.2% (Fig. 1g). In addition to the reduced size, the adult flies after ePR (hereafter, ePR-flies) exhibited a lighter coloration of the body-wall, particularly in the abdomen (Fig. 1f). This suggests inadequate pigmentation, potentially derived from a deficiency in precursors such as tyrosine (Tyr), which is essential for melanin synthesis^20–22^.

**Extended Data Fig. 1:**
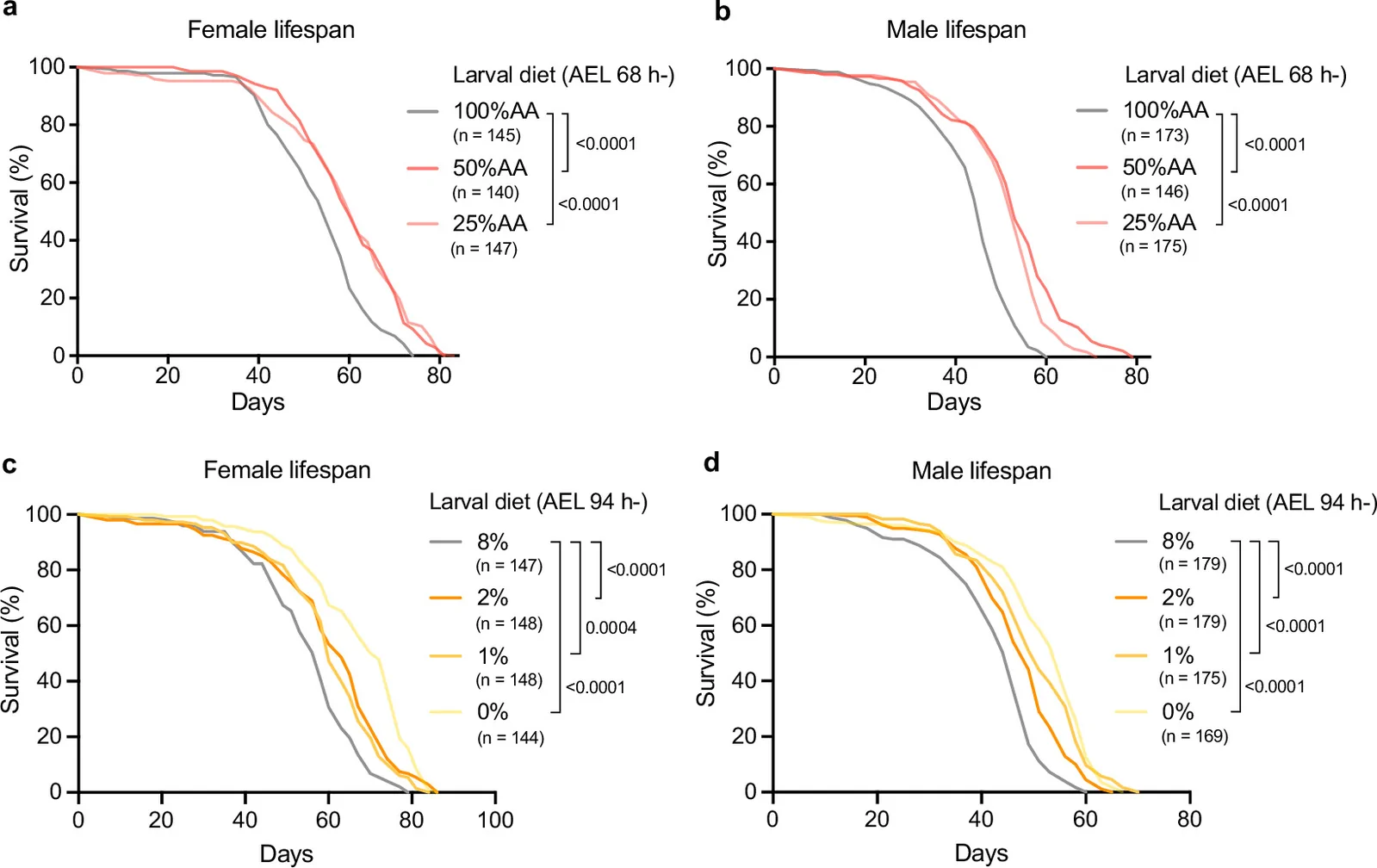
Lifespan analysis of ePR-flies under varying concentrations of amino acids. **a**,**b**, Lifespan of Canton-S females (**a**) and males (**b**) subjected to larval-specific amino acid restriction. **c**,**d**, Survival curves of Canton-S females (**c**) and males (**d**) subjected to ePR starting from 94 h after egg-laying (AEL). Sample sizes (n) for lifespan experiments are indicated within the respective panels. Statistical significance was determined by a log-rank test. Source data

Next, we characterized the adult phenotypes of these ePR-flies. Despite being reared on a standard diet after eclosion, female ePR-flies showed compromised fecundity, particularly during early adulthood (Fig. 1h,i). This was accompanied by a dose-dependent decrease in the number of ovarioles (Fig. 1j), consistent with reports that ovariole count is determined during the larval stage and is sensitive to larval nutrition^18,23^. These results indicate that ePR has irreversible developmental effects on both somatic growth and reproductive organs. When fed a standard diet in the adult stage, ePR-flies exhibited an increased lifespan in both sexes (Fig. 1k,l). To determine whether this longevity effect was attributable to reduced protein (amino acid) levels specifically, rather than other yeast-derived nutrients, we supplemented the 1% yeast larval diet with an amino acid cocktail based on the defined composition of a synthetic diet^24–26^ (see Methods). This supplementation effectively abolished the lifespan extension in ePR-flies of both sexes (Fig. 1m,n), suggesting that ePR-induced longevity is driven by amino acid availability. This was further corroborated using HolFast, a chemically defined diet designed for larvae^27^. Decreasing dietary amino acids to half or one-quarter specifically during the larval stage extended adult lifespan under standard diet conditions (Extended Data Fig. 1a,b).

We then investigated whether the timing of ePR influenced the lifespan-extension effect. ePR initiated at the third-instar stage (94 h AEL)—a point at which larvae can survive even under complete deprivation of yeast—also increased lifespan (Extended Data Fig. 1c,d). Notably, complete yeast deprivation (0% yeast diet) at this stage resulted in a greater lifespan extension than did the 1% or 2% yeast diets (Extended Data Fig. 1c,d). Together, these results show that restricting amino acid availability during juvenile stages extends lifespan.

To determine whether this longevity effect depends on adult dietary context, we examined the lifespan of ePR-flies across a range of adult dietary amino acid concentrations. ePR-flies exhibited lifespan extension across a broad physiological range of amino acid concentrations (10–100%), and this effect was abolished under extreme dietary conditions (0% or 300% amino acids) (Fig. 1o–r). Consistent with these observations, Cox proportional hazards analysis detected no significant interaction between larval and adult diets within the physiological range of amino acid concentrations, although a significant interaction emerged in female flies when the extreme dietary conditions were included (Supplementary Table 1). These results indicate that the lifespan extension induced by ePR is maintained across a broad range of adult amino acid levels and is therefore not restricted to a specific adult amino acid condition.

## ePR remodels early-life nutrient signalling

**Extended Data Fig. 2:**
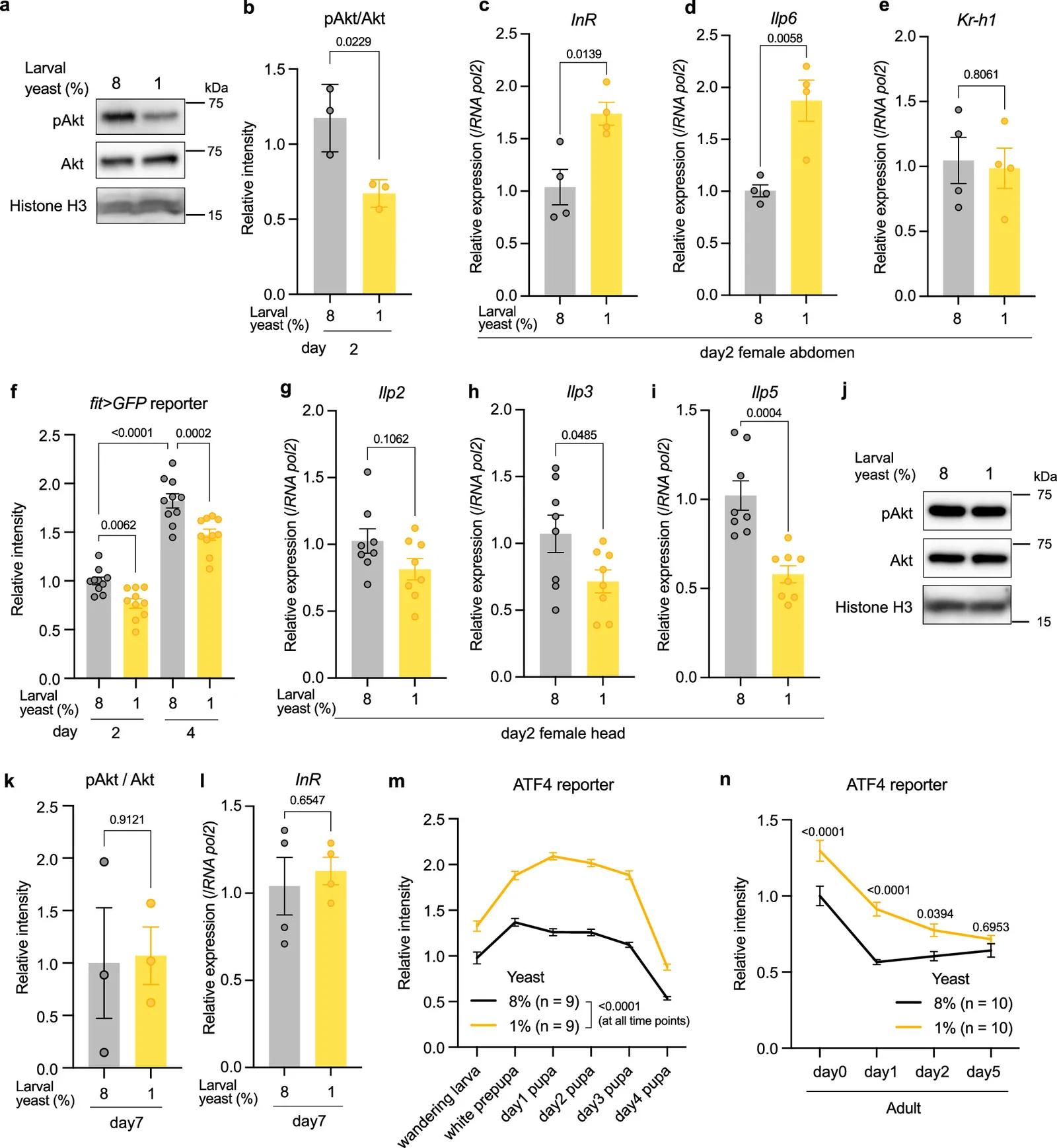
Effects of ePR on systemic nutritional signalling in adults. **a**,**b**, Representative immunoblots (**a**) and quantification (**b**) of Akt phosphorylation levels in adult abdominal carcasses of Canton-S female flies at day 2 with ePR. Antibodies used are anti-phosphorylated Akt (pAkt), anti-Akt, and anti-histone H3 (loading control). n = 3. **c**–**e**, qRT–PCR analysis of *InR* (**c**), *Ilp6* (**d**), and *Kr-h1* (**e**) in the abdominal carcass of Canton-S female flies at adult day 2 with ePR. n = 4. **f**, Quantification of *fit*>*GFP* reporter fluorescence in the abdomen of female flies at adult day 2 and day 4 with ePR. n = 10. **g**–**i**, qRT–PCR analysis of *Ilp2* (**g**), *Ilp3* (**h**), and *Ilp5* (**i**) in the heads of Canton-S female flies at adult day 2 with ePR. n = 8. **j**,**k**, Representative immunoblots (**j**) and quantification (**k**) of Akt phosphorylation levels in abdominal carcasses of Canton-S female flies at day 7 with ePR. Antibodies used are anti-phosphorylated Akt (pAkt), anti-Akt, and anti-histone H3 (loading control). n = 3. **l**, qRT–PCR analysis of *InR* in the abdominal carcasses of Canton-S female flies at day 7 with ePR. n = 4. **m**,**n**, Quantification of ATF4 reporter (*4E-BP*^*intron*^*-dsRed*) fluorescence of larvae and pupae (**m**) and sin adult female flies (**n**) with ePR. n = 9 (**m**) or 10 (**n**). Data are presented as mean ± s.e.m. Symbols represent biological replicates. Statistical significance was determined by a two-tailed Student’s *t*-test (**b**–**e**,**g**–**i**,**k**,**l**), one-way ANOVA with Holm-Šídák’s multiple comparison test (**f**), or two-way ANOVA with Šídák’s multiple comparison test (**m**,**n**). Source data

Lifespan extension is closely linked to the downregulation of insulin signalling^28^. It has been reported that yeast deprivation during the late larval stage decreases both insulin and juvenile hormone (JH) signalling in adult flies^18^, both of which can regulate *Drosophila* lifespan^29^. We found that ePR reduced the levels of phosphorylated Akt (p-Akt) in the abdominal carcass (enriched with fat body) of 2-day-old adult females, indicating suppressed insulin signalling (Extended Data Fig. 2a,b). Consistent with this suppression, we observed the activation of the transcription factor FoxO (forkhead box, subgroup O), which is typically repressed by insulin signalling. This was evidenced by the upregulation of FoxO target genes, including *insulin-like receptor* (*InR*) and *insulin-like peptide 6* (*Ilp6*), in the abdominal carcass (Extended Data Fig. 2c,d). By contrast, the expression of *Kr transcription factor homolog 1* (*Kr-h1*), a target gene of JH signalling, remained unchanged by ePR (Extended Data Fig. 2e). Furthermore, the expression of *female-specific independent of transformer* (*fit*), visualized by a GFP reporter, was suppressed in the abdomen of ePR-flies, reflecting a reduction in systemic insulin–mechanistic target of rapamycin (mTOR) signalling activity^30,31^ (Extended Data Fig. 2f). A decrease in the expression of insulin-like peptides in the head further corroborated the overall suppression of the insulin signalling axis (Extended Data Fig. 2g–i). Notably, this suppression was transient; p-Akt levels and *InR* expression returned to control levels by day 7 after eclosion (Extended Data Fig. 2j–l). Activating transcription factor 4 (ATF4) is another transcription factor that is activated by a decrease in dietary amino acid^32^. We monitored its activity using the *4E-BP*^*intron*^*-dsRed* reporter line^33^ and found that ATF4 remained active in the ePR-flies until day 2 of adulthood. However, this activity returned to baseline levels by day 5 (Extended Data Fig. 2m,n). Collectively, these findings indicate that larval nutrient restriction modulates nutrient-sensing pathways only transiently during the early adult stage.

## Larval diet shapes the adult proteome

Our phenotypic analysis suggests that early-life protein intake has a prolonged effect on early-adult physiology and lifespan. Previous efforts to track larval-derived nutrients into adulthood have used stable-isotope-labelled yeast (^15^N, ^13^C versus ^14^N, ^12^C) to quantify the turnover of carbon and nitrogen^34,35^. These analyses, combined with adult dietary restriction, have been used to evaluate nutrient investment strategies in both somatic and reproductive tissues. It was found that larval-derived nitrogen is slowly replaced by adult-derived nitrogen provisioned to eggs and in somatic tissues when adult diet is restricted. Those observations pose fundamental questions: how are specific larval proteins carried into the adult stage, and how do animals transition from using larval-derived amino acids to adult-acquired ones for the synthesis of specific proteins? To answer these questions requires the proteome-wide turnover of proteins to be analysed.

**Fig. 2:**
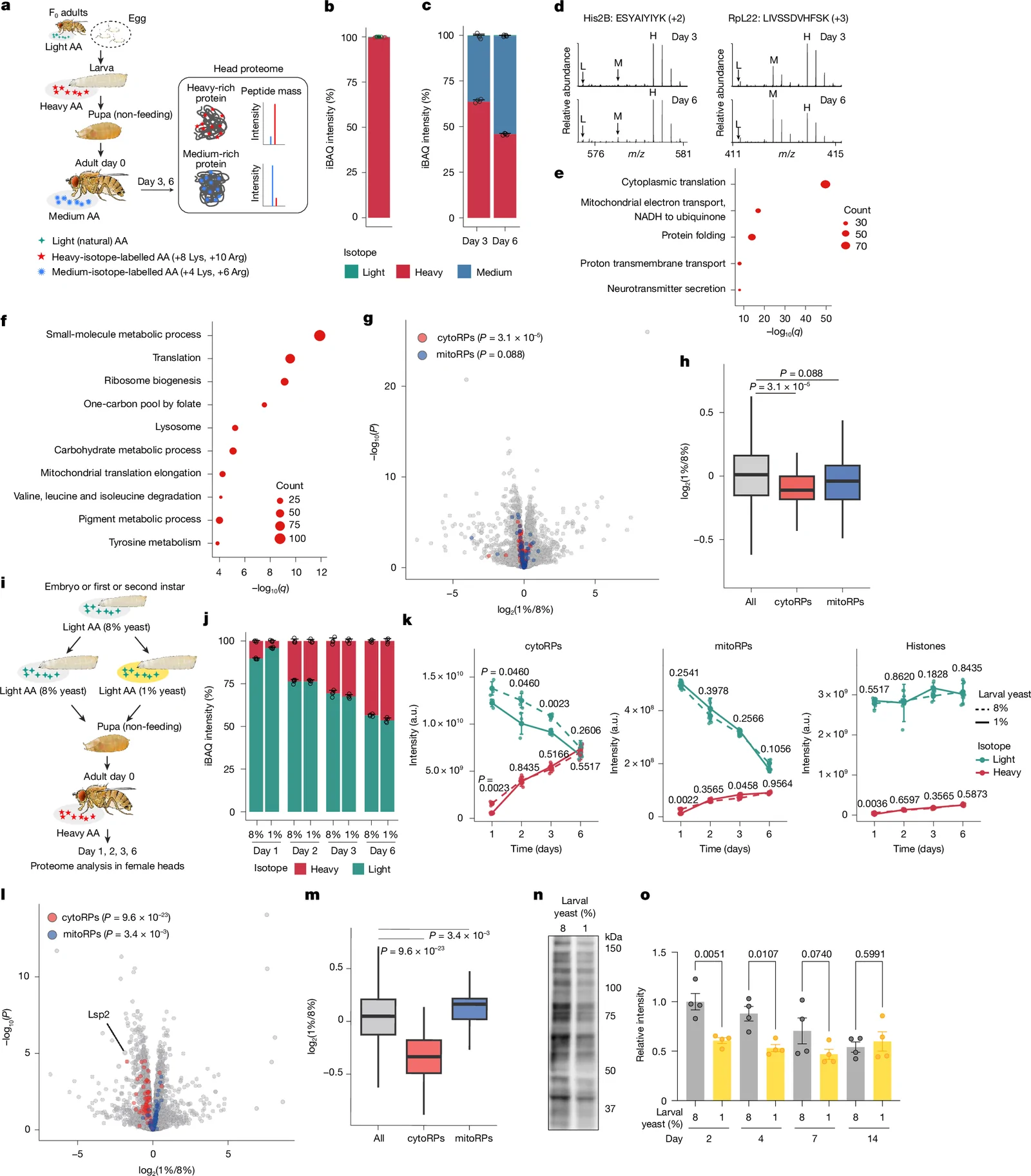
Larval diet influences proteome turnover, proteomic profile and translation. **a**, Experimental workflow for in vivo pSILAC. **b**,**c**, Relative abundance of light-, medium- and heavy-labelled proteins in wandering larvae (**b**) and adult heads (**c**) of Canton-S female flies. Protein abundance was estimated by intensity-based absolute quantification (iBAQ). *n* = 4. **d**, Mass spectra of His2B and RpL22 peptides. L, light; M, medium; H, heavy. **e**, GO enrichment analysis of the top 25% most abundant heavy-labelled proteins in adult heads at day 3. The *q* values were calculated using adjusted *P* values for multiple testing with Benjamini–Hochberg, and –log_10_ transformed. **f**–**h**, Proteome analysis in heads of Canton-S female flies at day 2. **f**, GO enrichment analysis of proteins decreased by ePR (*P* < 0.01 and log_2_(1%/8%) < –0.1). **g**, Volcano plot showing differential protein abundance. CytoRPs and mitoRPs are highlighted in red and blue, respectively. **h**, Distribution of log_2_ fold changes for all proteins (*n* = 6,025), cytoRPs (*n* = 81) and mitoRPs (*n* = 72). **i**, Experimental scheme of in vivo pSILAC analysis combined with ePR. Canton-S larvae were fed a 1% or 8% yeast diet, then adults were fed a synthetic diet containing heavy-isotope-labelled amino acids for 1, 2, 3 or 6 days. **j**, Proportions of light- and heavy-labelled protein abundance in the heads of female flies. **k**, Intensities of light- and heavy-labelled cytoRPs, mitoRPs and histones. Dots represent the median intensity of each protein group. *n* = 4. a.u., arbitrary unit. **l**, Volcano plot showing the differential abundance of light-labelled (larval-derived) proteins in adult heads at day 2. **m**, Distribution of log_2_ fold changes for light-labelled proteins for all proteins (*n* = 7,443), cytoRPs (*n* = 81) and mitoRPs (*n* = 71). **n**,**o**, Representative immunoblot at day 2 (**n**) and quantification (**o**) of protein synthesis in adult female heads. *n* = 4. Data are mean ± s.d. (**b**,**c**,**j**,**k**); box plots showing the median, quartiles and range (**h**,**m**); or mean ± s.e.m. (**o**). Symbols represent biological replicates. Statistical significance was determined by two-sided Wilcoxon rank-sum test (**g**,**h**,**l**,**m**), two-sided Welch’s *t*-test with Benjamini–Hochberg correction (**k**) or one-way ANOVA with Holm–Šídák’s multiple comparison test (**o**). Source data

To trace the fate of amino acids and proteins during the transition into adulthood, we used in vivo pulse stable-isotope labelling by amino acids in cell culture (in vivo pSILAC) through a synthetic diet. Larvae were reared on a ‘heavy’ synthetic diet containing isotope-labelled lysine (+8 Lys; ^13^C_6_, ^15^N_2_) and arginine (+10 Arg; ^13^C_6_, ^15^N_4_) in place of their ‘light’ (natural) counterparts (Fig. 2a). This approach allowed for the complete replacement of these dietary amino acids with stable isotopes without perturbing larval development, enabling precise quantification of proteome turnover under physiological conditions. After eclosion, adult flies were switched to a synthetic diet containing ‘medium’ isotope-labelled Lys (+4 Lys; D_4_) and Arg (+6 Arg; ^13^C_6_). After three and six days of adult feeding, we quantified the isotopic composition of proteins using nano-liquid chromatography and tandem mass spectrometry (nanoLC–MS/MS). We used head samples that contained various tissues including the brain, epidermis, muscle, haemocytes and fat body, but not the ovary and gut. Because somatic stem cells in adult *Drosophila* are localized mainly in the gut, cell division is negligible in the head, allowing for a straightforward assessment of proteome turnover independent of cell proliferation.

**Extended Data Fig. 3:**
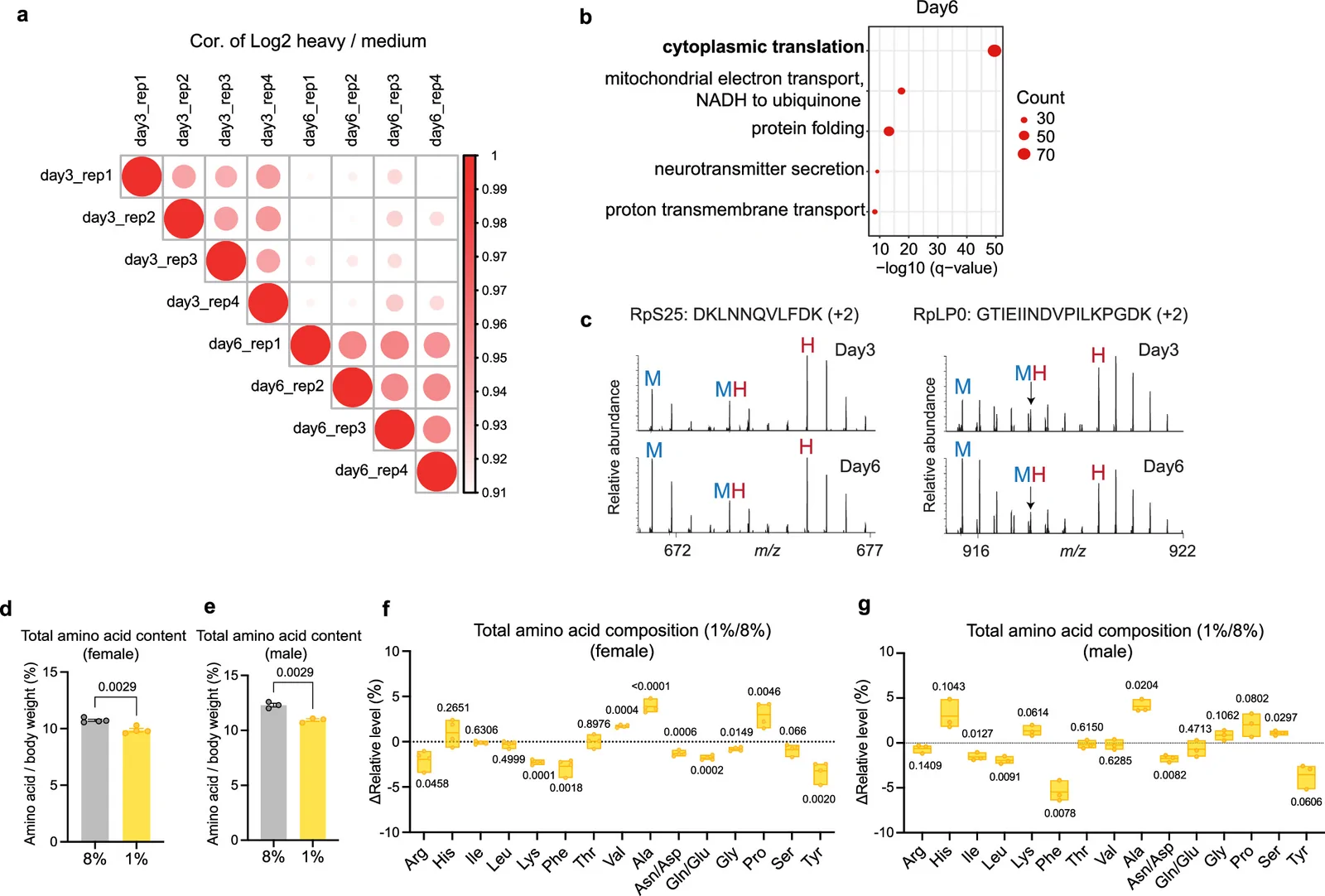
pSILAC analysis and amino acid profiling of ePR-flies. **a**, Multi-scatter plots showing the correlation of log_2_(heavy/medium) ratios between biological replicates, demonstrating high experimental reproducibility. **b**, GO enrichment analysis of the top 25% most abundant heavy-labelled proteins in heads of Canton-S female flies at day 6 (*P* < 0.01 and log_2_ 1%/8% < −0.1). *q* values are computed based on adjusted *P* values for multiple testing with Benjamini–Hochberg and –log_10_-transformed. **c**, Representative mass spectra of missed-cleaved tryptic peptides which contain two labelled lysine derived from RpS25 and RpLP0 proteins. Owing to amino acid recycling after protein degradation, chimera peaks containing medium and heavy amino acids can be observed. **d**–**g**, The total amino acid content (**d**,**e**) and relative amino acid composition (**f**,**g**) of Canton-S female flies at day 3 (**d**,**f**) and male flies at day 0 (**e**,**g**) with ePR. *n* = 4 (**d**,**f**) or 3 (**e**,**g**). Data are presented as mean ± s.e.m. (**d**,**e**) or as box plots showing the median, quartiles, and range (**f**,**g**). Symbols represent biological replicates. Statistical significance was determined by a two-tailed Student’s *t*-test (**d**–**g**). Source data

The proteomic analysis yielded highly reproducible results, as evidenced by the strong correlation of heavy/medium labelling ratios among biological replicates (Extended Data Fig. 3a). We first confirmed that the labelling efficiency at the end of the larval stage was 99.4%, demonstrating the successful incorporation of heavy amino acids into the larval proteome (Fig. 2b and Supplementary Table 2). At day 3 of adulthood, larva-derived heavy proteins constituted 63.7% of the total proteome, outnumbering the newly synthesized medium proteins. This proportion subsequently declined to 45.8% by day 6 (Fig. 2c and Supplementary Table 2). These findings indicate that the early-adult proteome retains a substantial fraction of proteins made from larval amino acids (Fig. 2c). Note that these larva-derived proteins could be either (1) long-lived proteins carried over intact from the larval stage or (2) proteins that are newly synthesized during metamorphosis or early adulthood by recycling the larval proteins. Consistent with the former possibility, histones, such as histone H2B (H2B), which is known to be long-lived^36,37^, were significantly enriched with the heavy isotope (Fig. 2d).

Gene Ontology (GO) enrichment analysis of the abundant proteins labelled by heavy isotopes at both day 3 and day 6 identified cytoplasmic translation as the top hit, followed by mitochondrial electron transport (Fig. 2e and Extended Data Fig. 3b). Notably, 77 ribosomal proteins (RPs) (73 cytosolic and 4 mitochondrial) were found to be heavy-rich (top 25%) in the early-adult proteome (Supplementary Table 2). For instance, 60S ribosomal protein L22 (RpL22) exhibited high heavy-isotope levels at day 3; however, the heavy/medium ratio declined rapidly by day 6, indicating a higher turnover rate compared with histones (Fig. 2d). These data suggest that RPs in early adulthood are either carried over from the developmental stage or synthesized using larva-derived amino acid pools. To distinguish between protein carryover and amino acid recycling, we analysed incompletely digested chimeric peptides^38^ from RpS25 and RpLP0. These peptides contained both heavy- and medium-labelled Lys in the same peptide chain in day-3 and day-6 head samples, which indicates that the peptides are synthesized from both larva-derived recycled amino acids and adult-derived amino acids (Extended Data Fig. 3c). This finding provides evidence that larval-derived proteins are not merely carried over intact but also synthesized de novo from amino acids derived from proteins produced in developmental stages.

## ePR reduces adult ribosomes and translation

To comprehensively understand how ePR influences adult protein abundance and expression profiles, we performed amino acid quantification, proteome analysis and RNA sequencing (RNA-seq) analysis on early-adult flies. For amino acid quantification, whole-body samples were subjected to acid hydrolysis (see Methods) to determine the total amino acid content, encompassing both protein-bound and free amino acids. ePR-flies of both sexes showed a significant reduction in total amino acid levels, even after normalizing for body weight (Extended Data Fig. 3d,e). Beyond this quantitative reduction, we observed distinct alterations in amino acid composition (Extended Data Fig. 3f,g). For instance, in female flies, valine, alanine and proline were increased by ePR, whereas arginine, lysine, phenylalanine (Phe) and Tyr were decreased (Extended Data Fig. 3f). These compositional shifts suggest that ePR fundamentally modifies the adult amino acid profile, potentially through a bias in the carryover of specific amino acids and proteins from the larval stage.

**Extended Data Fig. 4:**
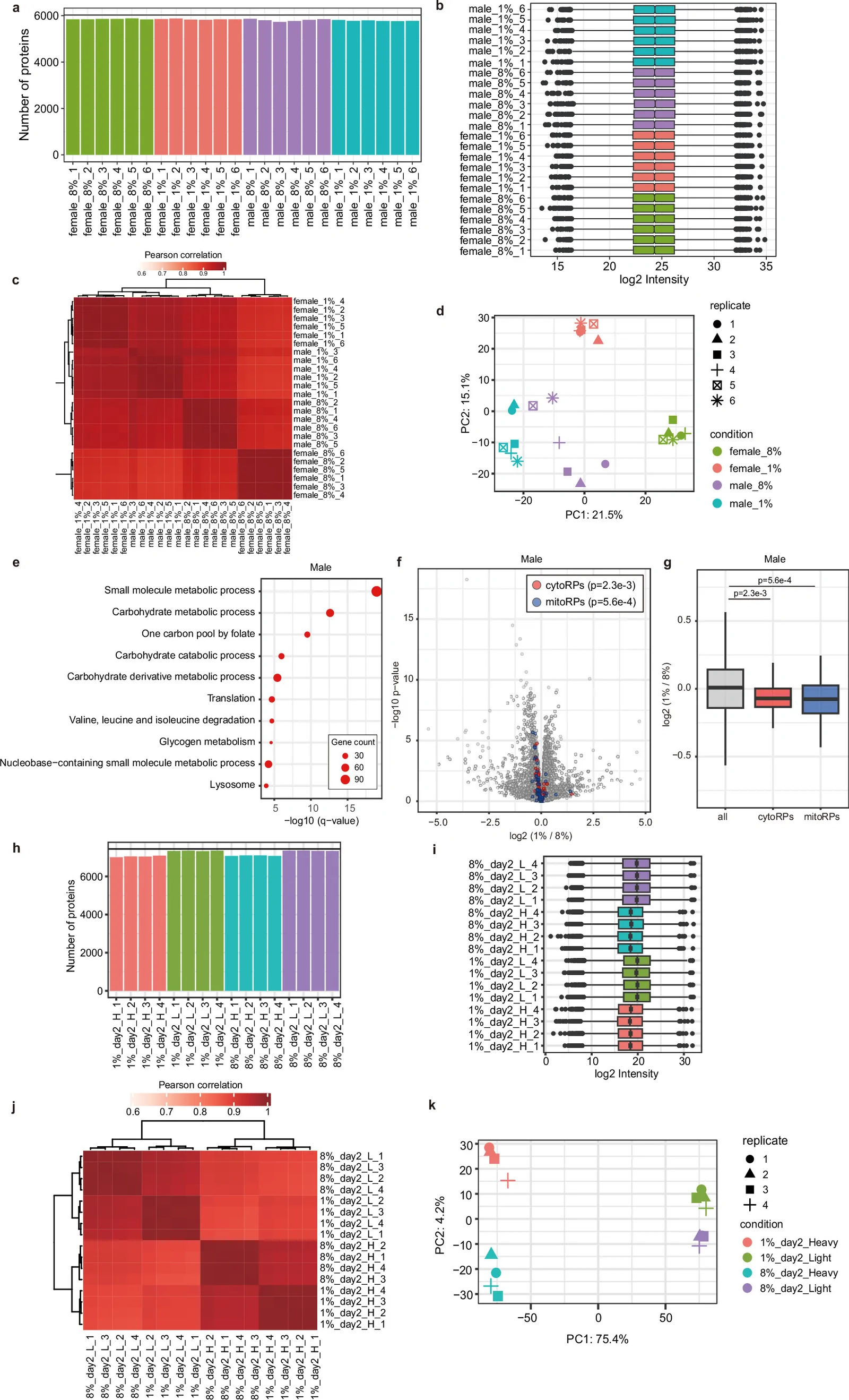
Quality control and proteomic profiling of ePR-flies. **a**, Number of proteins quantified in each sample: Out of 6,559 total identified proteins, 6,025 proteins were consistently quantified across all six biological replicates in at least one condition and included in the subsequent analysis. **b**–**d**, Data distribution and reproducibility metrics for the adult head proteome, including log_2_ protein intensities (**b**), Pearson correlation matrix (**c**), and principal component analysis (PCA) plot (**d**) of individual biological replicates. The number of proteins included in the box plots corresponds to that shown in (**a**). **e**, GO enrichment analysis of proteins whose expression was significantly downregulated by ePR in heads of Canton-S male flies at day 2 (*P* < 0.01 and log_2_ 1%/8% < −0.1). *q* values are computed based on adjusted *P* values for multiple testing with Benjamini–Hochberg and –log_10_-transformed. **f**, Volcano plot showing differential protein abundance in the head of Canton-S male flies under ePR at adult day 2. Red and blue circles denote cytoRPs and mitoRPs, respectively. **g**, Distribution of log_2_ fold changes (1%/8%) in male heads for all proteins (n = 6,025), cytoRPs (n = 81), and mitoRPs (n = 72). **h**–**k**, Quality control metrics for the pSILAC proteomic datasets, including the number of quantified proteins (**h**), log_2_ protein intensities (**i**), Pearson correlation matrix (**j**), and PCA plot (**k**). The number of proteins included in the box plots corresponds to that shown in **h**. Box plots in **b**,**i** show the median (centre line), the interquartile range (box), and the smallest and largest values within 1.5× the interquartile range (whiskers). Values beyond the whiskers are shown as individual points. Box plots in **g** show the median (centre line), the interquartile range (box), and the minimum and maximum values (whiskers). Symbols represent biological replicates. Statistical significance was determined by a two-sided Wilcoxon rank-sum test (**f**,**g**). Source data

We then performed proteomic analysis on the heads of day-2 adult flies to identify the proteins most affected by ePR (Extended Data Fig. 4a–d and Supplementary Table 3). GO enrichment analysis revealed that biological processes related to translation, ribosome biogenesis and mitochondrial translation elongation, as well as metabolic processes, were significantly downregulated in response to ePR (Fig. 2f). Indeed, the levels of cytosolic ribosomal proteins (cytoRPs) were lower in day-2 adult females and males after ePR (Fig. 2g,h and Extended Data Fig. 4e–g). We also noted a decrease in Tyr and pigment metabolism (Fig. 2f), which aligns with the pale body coloration observed in ePR-flies (Fig. 1f).

Consistently, the results from RNA-seq analysis showed a trend towards the suppression of genes encoding cytoRPs in ePR-flies (Supplementary Table 4), whereas genes encoding mitochondrial ribosomal proteins (mitoRPs) remained unaffected (Supplementary Table 4). Furthermore, GO analyses showed that ePR upregulates cuticle-related genes and stress-response pathways in both the head and the abdomen, whereas metabolic genes involved in amino acid, carbohydrate and nucleotide metabolism are downregulated in the head (Supplementary Table 4). These findings suggest that an adaptive transcriptional response to ePR relates to translation and metabolism.

To determine whether ePR modulates adult proteome turnover, we performed a time-course in vivo pSILAC assay (Fig. 2i, Extended Data Fig. 4h–k and Supplementary Table 2). In this experiment, larvae were reared on either an 8% or a 1% yeast diet (containing light amino acids), and the newly eclosed adults were switched to a synthetic diet containing heavy amino acids (+8 Lys/+10 Arg). We prepared samples at days 1, 2, 3 and 6 to monitor the proteome turnover over time (Fig. 2i). Consistent with the results in Fig. 2c, the heavy-labelled proteomes were enriched over time and reached about half at day 6 (Fig. 2j). At day 1, ePR-flies exhibited a lower heavy fraction; however, from day 2 onwards, the heavy/light ratio became nearly identical to that of controls (Fig. 2j). The reduced incorporation of adult-derived (heavy) amino acids at day 1 in ePR-flies suggests not only reduced protein synthesis, but also decreased feeding rates. Nevertheless, after day 2, the relative reliance on larva- versus adult-derived amino acids for the global proteome did not differ significantly between the dietary groups (Fig. 2j).

We then specifically examined the turnover of RPs. In control flies, both cytoRPs and mitoRPs were gradually replaced by heavy-labelled (adult-derived) proteins, with cytoRPs showing faster replacement than mitoRPs (Fig. 2k). In ePR-flies, however, the levels of light-labelled (larva-derived) cytoRPs were significantly lower during the first three days (Fig. 2k). Using a volcano plot from the day-2 samples, we confirmed that the levels of larva-derived cytoRPs were lower in ePR-flies (Fig. 2l,m). Notably, although heavy-labelled (adult-derived) cytoRPs also showed a transient decrease at day 1, their levels became comparable with controls by day 2 (Fig. 2k). The transient decrease at day 1 in heavy-labelled proteins was commonly observed across cytoRPs, mitoRPs and histones, reflecting a general reduction in protein synthesis or feeding at the onset of adulthood by ePR (Fig. 2j,k). Together, these data suggest that ePR selectively reduces the pool of larva-derived cytoRPs without affecting the synthesis of new RPs from adult-acquired amino acids.

Given the global reduction in cytoRPs, we hypothesized that ePR suppresses translation capacity in early adulthood. Using a SUnSET (surface sensing of translation) assay^39^ to monitor the incorporation of puromycin into nascent peptides, we confirmed that protein synthesis was indeed downregulated in early-adult ePR-flies (Fig. 2n,o). In control flies, we found that the protein synthesis rate was high at first in early adulthood and declined with age (Fig. 2o). The suppressive effect of ePR on translation lasted only one week (Fig. 2o), consistent with the fact that an ePR-induced decrease in the abundance of cytoRPs was no longer observed at day 6 (Fig. 2k).

Although we cannot provide direct evidence to connect the decrease in cytoRPs and translation in early adulthood to ePR-associated longevity (because no experimental tool is at present available that allows us to increase the levels of cytoRPs specifically), inhibiting the early-adult spike in translation during the first ten days of flies’ adult lives has been shown to extend lifespan^40^. Collectively, our results suggest that larval protein intake influences early-adult translation levels via the altered RP components, thereby regulating organismal lifespan.

## ePR reduces Lsp2 carryover and synthesis

Our data indicate that ePR significantly reduces the levels of cytoRPs, which correlates with decreased translation in early adulthood. We hypothesized that a protein, or proteins, that is affected by larval nutrition and carried over from the larval stage could mediate the nutritional-memory effect of ePR. From these criteria, we identified the storage protein Lsp2 in a pSILAC assay (Fig. 2l). Larval serum proteins (LSPs) are storage proteins that are best known for their function in holometabolous insects, which face nutrient restriction during non-feeding pupal development^41,42^. Detailed functional studies of these storage proteins in recent years have revealed that they are a specific requirement for late-developed adult tissues^43^. These proteins are synthesized mainly in the larval fat body, secreted into the haemolymph and subsequently taken up by the fat body during pupal development. *Lsp2* and other storage-protein genes are highly expressed in late larvae and early pupae, and their expression typically declines sharply towards the end of metamorphosis^43^. Although long considered mere transient reservoirs for development, Lsp2 is known to persist and be expressed in adult flies^44^. Indeed, Lsp2 is one of the major soluble proteins in the early-adult haemolymph^40^. However, the physiological role of adult Lsp2 remains mostly uninvestigated, except for suggestions that storage proteins support adult fecundity^45,46^.

**Fig. 3:**
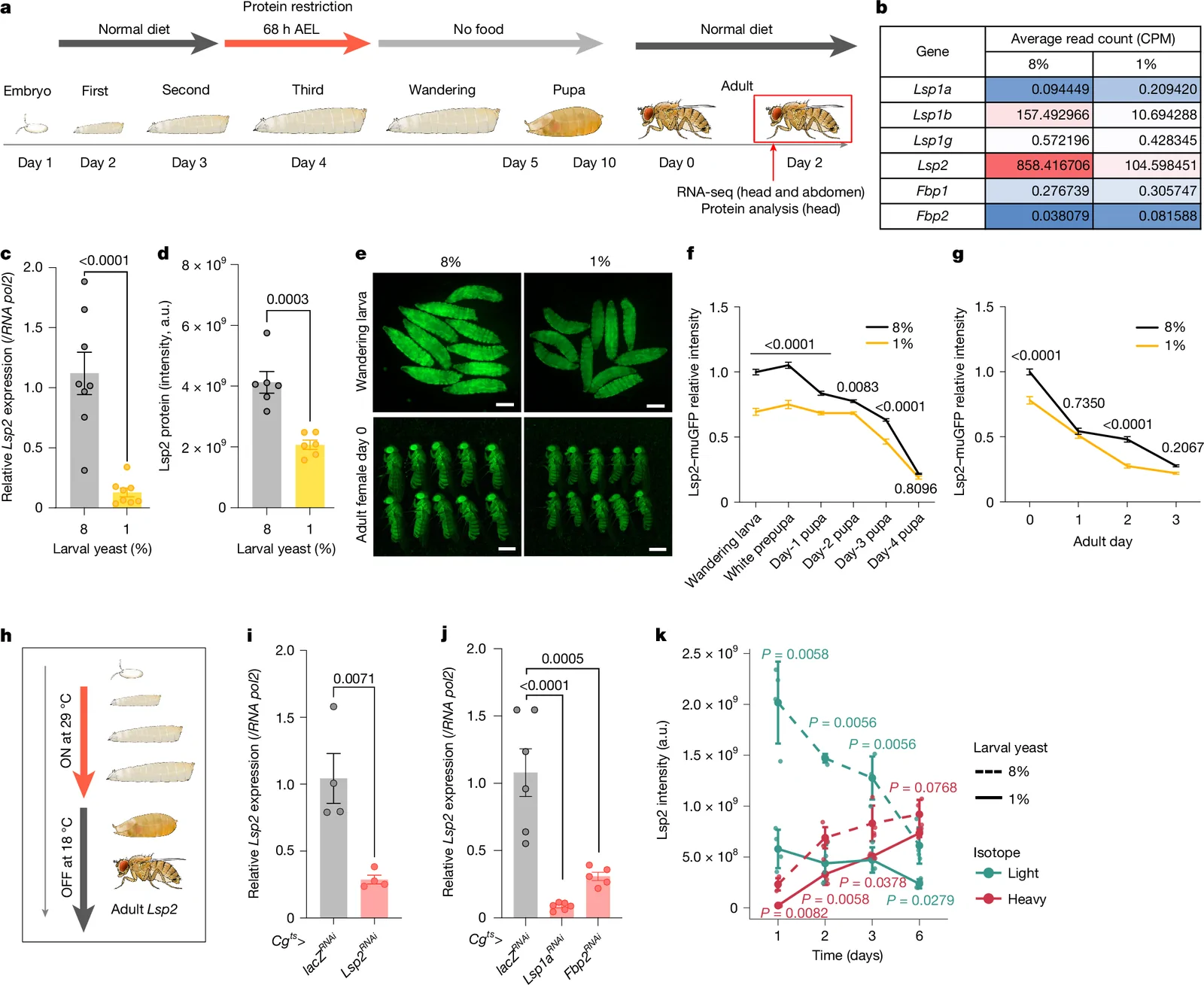
Larval protein restriction decreases Lsp2 in early life stages. **a**, Experimental workflow for adult transcriptome and proteome analyses after ePR. **b**, Average read counts (in counts per million; CPM) of genes encoding storage proteins, from transcriptome analysis in Canton-S female flies at adult day 2. *n* = 4. **c**,**d**, *Lsp2* abundance at the mRNA level quantified by qRT–PCR (**c**, *n* = 8) and Lsp2 abundance at the protein level quantified by proteome analysis (**d**) in the heads of Canton-S female flies at adult day 2. a.u., arbitrary unit. **e**–**g**, Representative fluorescence images (**e**) and quantification (**f**,**g**) of Lsp2–muGFP reporter activity in larvae (**e**), pupae (**f**) and adult female flies (**e**,**g**). Scale bars, 1 mm. *n* = 10 (larvae and adults) or 8 (pupae). **h**–**j**, Experimental scheme (**h**) and qRT–PCR analysis of *Lsp2* after larva-specific knockdown of *Lsp2* (**i**), *Lsp1a* (**j**) or *Fbp2* (**j**) using the fat-body driver *Cg-Gal4* with *tub-Gal80*^*ts*^. *n* = 4 (**i**), 5 (**j**, *Fbp2*^*RNAi*^) or 6 (**j**, *Lsp2*^*RNAi*^, *Lsp1a*^*RNAi*^). **k**, Intensities of light- and heavy-labelled Lsp2 protein in the heads of Canton-S female flies on the indicated days, on the basis of the in vivo pSILAC described in Fig. 2i. *n* = 4. Data are mean ± s.e.m. (**c**,**d**,**f**,**g**,**i**,**j**) or mean ± s.d. (**k**). Symbols represent biological replicates. Statistical significance was determined by two-tailed Student’s *t*-test (**c**,**d**,**i**), two-way ANOVA with Šídák’s multiple comparison test (**f**,**g**), one-way ANOVA with Dunnett’s multiple comparison test (**j**) or two-sided Welch’s *t*-test with Benjamini–Hochberg correction (**k**). Source data

**Extended Data Fig. 5:**
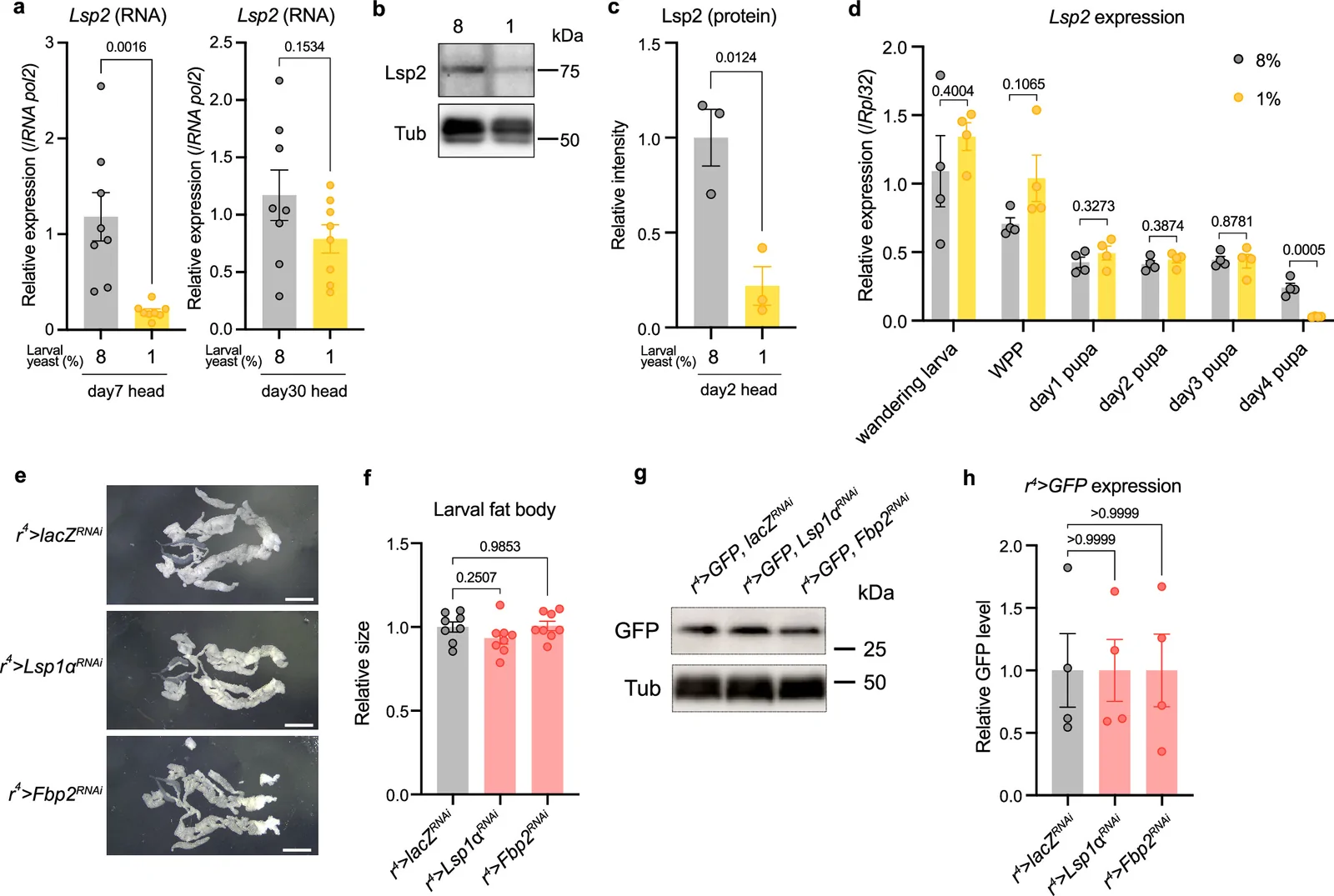
Lsp2 expression levels in ePR-flies and fat-body sizes with storage-protein knockdown. **a**, qRT–PCR analysis of *Lsp2* mRNA levels in the head of Canton-S female flies at adult day 7 and 30 with ePR. n = 8. **b**,**c**, Representative immunoblots (**b**) and quantification (**c**) of Lsp2 protein levels in the heads of Canton-S female flies at adult day 2 with ePR. Antibodies used are anti-Lsp2 and anti-α + β tubulin (loading control). n = 3. **d**, qRT–PCR analysis of *Lsp2* in the whole bodies of Canton-S female flies with ePR. n = 4. **e**,**f**, Representative images of larval fat bodies (**e**) and quantification of larval fat-body size (**f**) with control (*lacZ*^*RNAi*^), *Lsp1α*^*RNAi*^, or *Fbp2*^*RNAi*^ using fat-body driver (*r*^*4*^*-Gal4*). n = 8. Scale bar = 1 mm. **g**,**h**, Representative immunoblots (**g**) and quantification (**h**) of GFP protein levels in the heads of Canton-S female flies with control (*lacZ*^*RNAi*^), *Lsp1α*^*RNAi*^, or *Fbp2*^*RNAi*^ using fat-body driver (*r*^*4*^*-Gal4*). Antibodies used are anti-GFP and anti-α + β tubulin (loading control). n = 4. Data are presented as mean ± s.e.m. Symbols represent biological replicates. Statistical significance was determined by a two-tailed Student’s *t*-test (**a**,**c**,**d**) or one-way ANOVA with Dunnett’s multiple comparison test (**f**,**h**). Source data

Our RNA-seq analysis confirmed that *Lsp2* is expressed in the heads of adult female flies; however, other storage proteins, with the exception of *Lsp1b*, exhibited much lower levels of expression (Fig. 3a,b). Notably, ePR led to a significant decrease in *Lsp2* transcript levels in both the head and the abdominal carcass of adult flies at day 2 (Fig. 3b and Supplementary Table 4). We further corroborated this downregulation by quantitative PCR with reverse transcription (qRT–PCR), which showed that *Lsp2* transcript levels were reduced in female heads at both day 2 and day 7 after eclosion, but had recovered by day 30 (Fig. 3c and Extended Data Fig. 5a). Proteomic analysis further identified Lsp2 as one of the most abundant proteins in the adult head, and showed that its levels were decreased by ePR (Fig. 3d and Supplementary Table 5). We confirmed the reduction of Lsp2 protein in the head at day 2 by western blot analysis (Extended Data Fig. 5b,c).

To visualize Lsp2 protein dynamics with greater precision, we generated *Lsp2-monomeric ultrastable green fluorescent protein* (*muGFP*) knock-in flies using CRISPR–Cas9. As expected, Lsp2–muGFP was systemically detected during the late larval stage (Fig. 3e), with levels gradually declining throughout the pupal and early-adult stages (Fig. 3f,g). Notably, ePR further reduced Lsp2–muGFP intensity across all of these developmental stages (Fig. 3e–g).

Given the observed reduction in adult *Lsp2* mRNA and protein levels, we first hypothesized that *Lsp2* transcription might be constitutively downregulated from the larval stage onwards. However, *Lsp2* mRNA expression remained unaffected by ePR during the wandering larval and early pupal stages, showing a significant decrease only from day 4 of the pupal stage (Extended Data Fig. 5d).

To test whether larval levels of Lsp2 protein directly influence subsequent expression in adulthood, we transiently knocked down *Lsp2* in the larval fat body using the *Cg-Gal4* driver combined with temperature-sensitive *Gal80* (*Gal80*^*ts*^). This larval-specific knockdown was sufficient to reduce *Lsp2* mRNA expression in early adults (Fig. 3h,i). Notably, when we knocked down genes that encode other major storage proteins, including *Larval serum protein 1α* (*Lsp1a*) and *Fat body protein 2* (*Fbp2*), using RNA interference (RNAi) lines previously verified for their specificity^43^, *Lsp2* expression in adult flies was significantly decreased (Fig. 3j). This manipulation did not cause apparent defects in fat-body development (Extended Data Fig. 5e–h). These results suggest the existence of a feed-forward regulatory mechanism that maintains storage-protein expression, a concept also demonstrated in the accompanying study^47^.

We used a combination of in vivo pSILAC and ePR to study the turnover of Lsp2 protein in early adulthood. In control flies, Lsp2 at day 1 was predominantly light labelled (larva-derived), and was gradually replaced by heavy-labelled (adult-derived) Lsp2 over time (Fig. 3k and Supplementary Table 2). By day 6, the heavy-labelled fraction exceeded the light-labelled pool. As expected, the levels of light-labelled Lsp2 were significantly lower in ePR-flies across all time points (days 1, 2, 3 and 6), confirming a reduced carryover of larval protein reserves. Notably, the levels of heavy-labelled Lsp2 were also significantly lower in ePR-flies than they were in control flies during the first three days of adulthood (Fig. 3k), indicating that ePR suppresses both the legacy of larval Lsp2 and its de novo synthesis in early adulthood.

Collectively, these results support a model of ‘nutritional memory’ driven by ePR: protein restriction during larval development triggers a sustained reduction in Lsp2 at both the mRNA and the protein level that persists into adulthood. Because storage proteins constitute a crucial reservoir of amino acids for the synthesis of new proteins during the non-feeding pupal stage, the depletion of Lsp2 is likely to constrain the production of cytoRPs during metamorphosis. This deficit in cytoRPs is then carried over into early adulthood, in which it limits global translation capacity, potentially leading to lifespan extension. Furthermore, the observation that de novo Lsp2 synthesis remains suppressed in adult ePR-flies suggests that Lsp2 exerts other regulatory influences beyond its conventional role as an amino acid source during metamorphosis.

## Lsp2 regulates translation and longevity

**Fig. 4:**
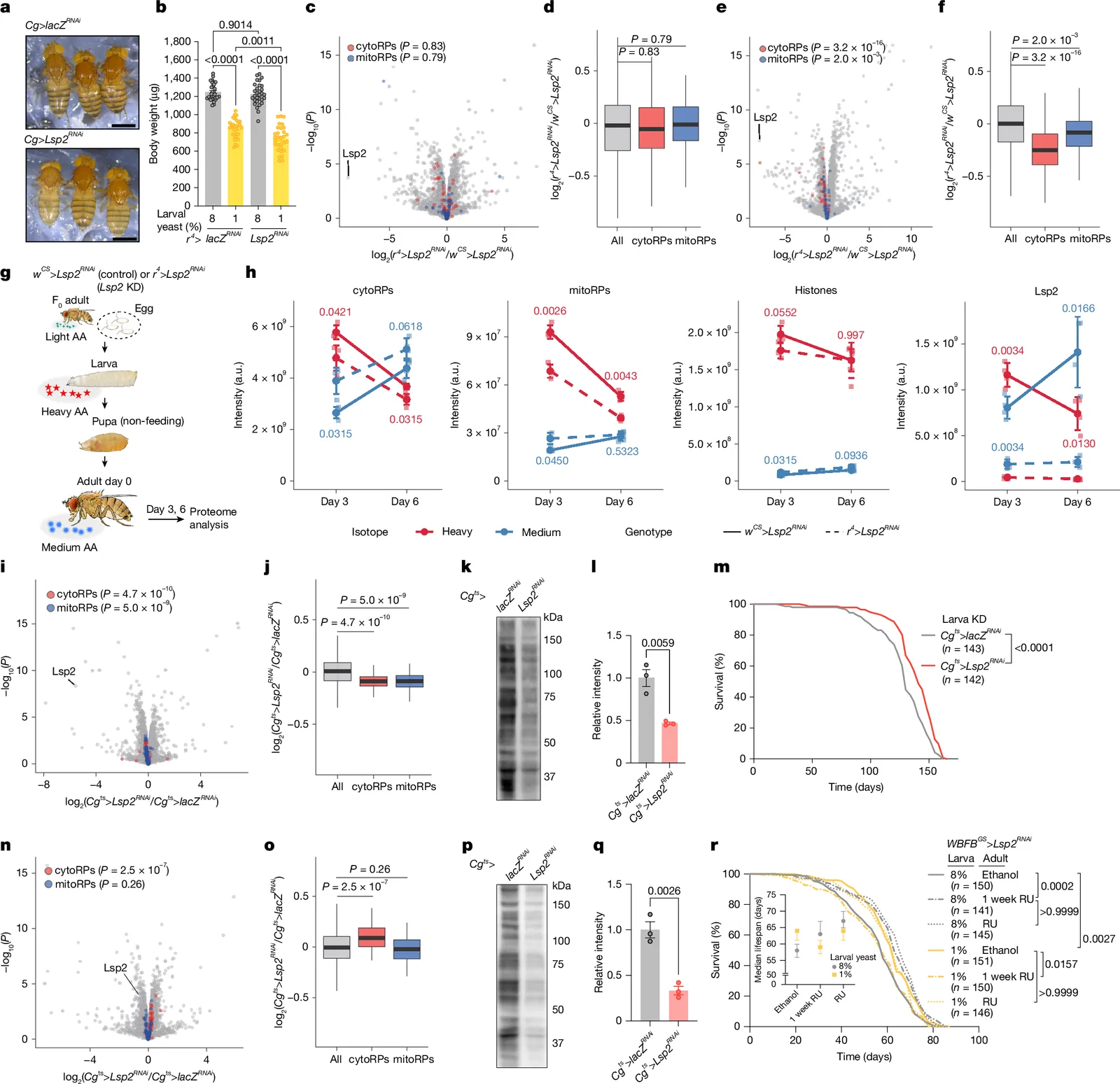
Knockdown of *Lsp2* suppresses early-adult translation and extends lifespan. **a**, Adult female flies at day 0 with *Lsp2* knockdown using the fat-body driver *Cg-Gal4*. Scale bars, 1 mm. **b**, Body weight of female flies at day 1 with *Lsp2* knockdown (*r*^*4*^*-Gal4*). *n* = 30. **c**–**f**, Volcano (**c**,**e**) and box (**d**,**f**) plots showing differential protein abundance in larvae at 96 h AEL (**c**,**d**) or in white prepupae (**e**,**f**). CytoRPs and mitoRPs are highlighted in red and blue, respectively. *n* = 4,511 (all), 76 (cytoRPs) and 57 (mitoRPs) in **d**, and 6,921 (all), 85 (cytoRPs) and 71 (mitoRPs) in **f**. **g**, Experimental workflow for in vivo pSILAC combined with *Lsp2* knockdown (KD). **h**, Intensities of heavy- and medium-labelled cytoRPs, mitoRPs, histones and Lsp2 in the heads of female flies at days 3 and 6. *n* = 4. a.u., arbitrary unit. **i**,**j**, Volcano (**i**) and box (**j**) plots showing differential protein abundance in the heads of adult female flies at day 4 after larval- and pupal-specific *Lsp2* knockdown (*Cg-Gal4*, *tub-Gal80*^*ts*^). *n* = 8,115 (all), 83 (cytoRPs) and 72 (mitoRPs). **k**,**l**, Representative immunoblot (**k**) and quantification (**l**) of protein synthesis in the heads of female flies at day 3–4 after larval- and pupal-specific *Lsp2* knockdown. *n* = 3. **m**, Survival curves of female flies with larval-specific *Lsp2* knockdown (*Cg-Gal4*, *tub-Gal80*^*ts*^). **n**,**o**, Volcano (**n**) and box (**o**) plots showing differential protein abundance in adult heads at day 2 after adult-specific (day 0–2) *Lsp2* knockdown. *n* = 8,114 (all), 83 (cytoRPs) and 72 (mitoRPs). **p**,**q**, Representative immunoblot (**p**) and quantification (**q**) of protein synthesis in the heads of female flies at day 2–3 after adult-specific (day 0–3) *Lsp2* knockdown. *n* = 3. **r**, Survival curves and median lifespan (inset) of female flies after adult-specific *Lsp2* knockdown using the inducible fat-body driver *WBFB*^*GS*^. RU486 (200 μM) was administered either during the first week or throughout adulthood. Error bars indicate 95% confidence intervals (**r**). Sample sizes (*n*) for lifespan experiments are indicated. Data are mean ± s.e.m. (**b**,**l**,**q**); mean ± s.d. (**h**); or box plots showing the median, quartiles and range (**d**,**f**,**j**,**o**). Symbols represent biological replicates. Statistical significance was determined by one-way ANOVA with Šídák’s multiple comparison test (**b**), two-sided Wilcoxon rank-sum test (**c**–**f**,**i**,**j**,**n**,**o**), two-sided Welch’s *t*-test with Benjamini–Hochberg correction (**h**), two-tailed Student’s *t-*test (**l**,**q**) or log-rank test (**m**,**r**). Source data

**Extended Data Fig. 6:**
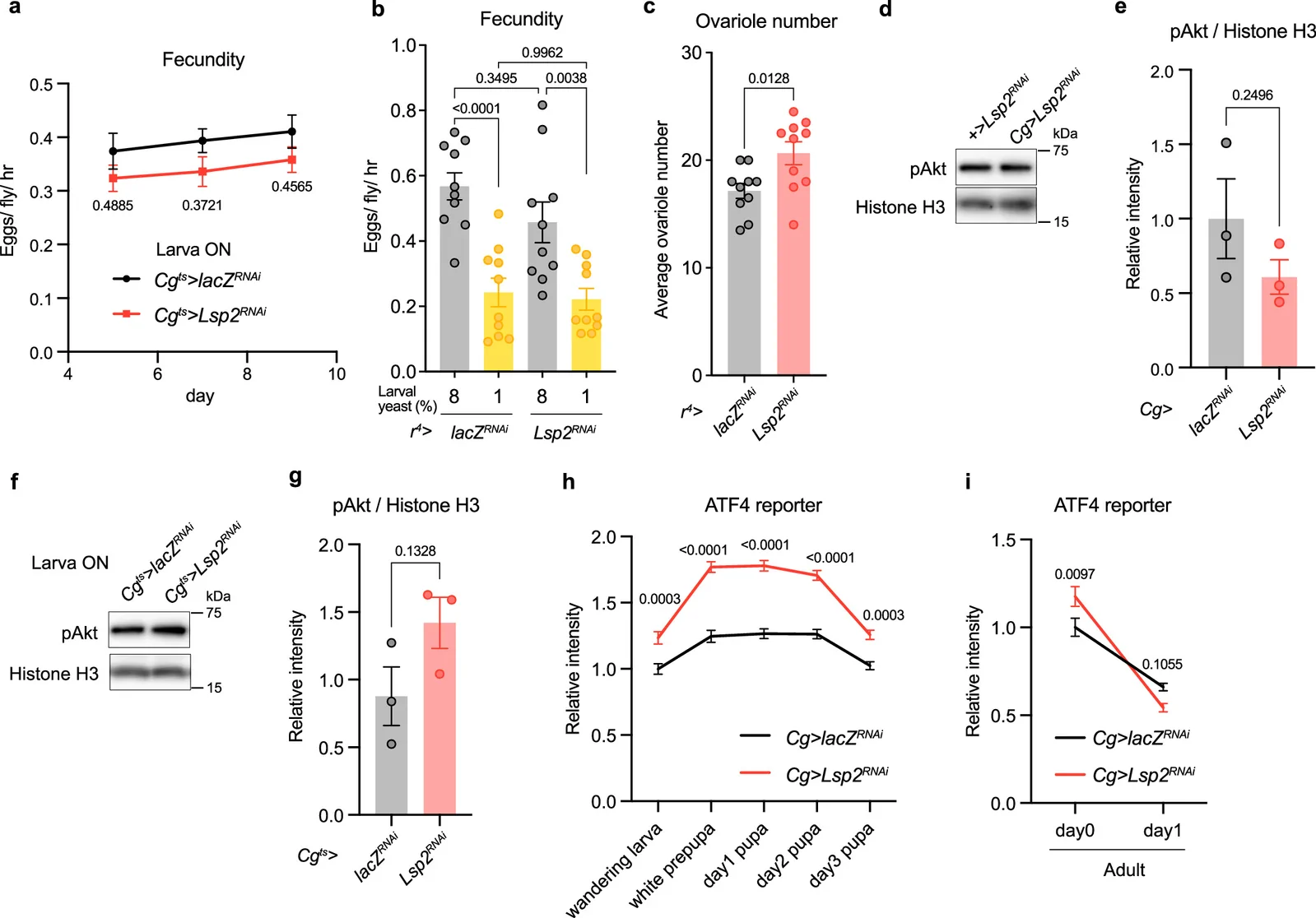
Effects of *Lsp2* knockdown on reproduction and nutritional signalling pathways. **a**, Daily fecundity of females from adult day 5 to 9 with larval-specific *Lsp2* knockdown using fat body driver *Cg-Gal4* combined with *tub-Gal80*^*ts*^. n = 10. **b**, Fecundity of female flies at adult day 4–5 with *Lsp2* knockdown using fat body driver *r*^*4*^*-Gal4* combined with ePR. n = 10. **c**, Ovariole number per ovary in female flies at adult day 9 with *Lsp2* knockdown using *r*^*4*^*-Gal4*. n = 10. **d**–**g**, Representative immunoblots (**d**,**f**) and quantification (**e**,**g**) of Akt phosphorylation levels in adult abdominal carcasses of adult female flies with *Lsp2* knockdown using fat body driver *Cg-Gal4* (**d**,**e**) or *Cg-Gal4* combined with *tub-Gal80*^*ts*^ (**f**,**g**). For transient manipulation (**f**,**g**), knockdown was performed during the larval stage. Samples were collected at adult day 2 (**d**,**e**) or day 6 (**f**,**g**). Antibodies used are anti-phosphorylated Akt (pAkt) and anti-histone H3 (loading control). n = 3. **h**,**i**, Quantification of ATF4 reporter (*4E-BP*^*intron*^*-dsRed*) fluorescence in whole larvae and pupae (**h**) or adult abdomens (**i**) with *Lsp2* knockdown using fat-body driver *Cg-Gal4*. n = 10. Data are presented as mean ± s.e.m. Symbols represent biological replicates. Statistical significance was determined by two-way ANOVA with Šídák’s multiple comparison test (**a**,**h**,**i**), one-way ANOVA with Šídák’s multiple comparison test (**b**), or a two-tailed Student’s *t*-test (**c**,**e**,**g**). Source data

To test the functional contribution of Lsp2 to the phenotypes observed in ePR-flies, we performed fat-body-specific knockdown of *Lsp2*. We found that *Lsp2* knockdown by RNAi throughout the larval and adult stages resulted in a lighter body colour, particularly in the abdominal pigmentation, mirroring the effect observed under ePR (Fig. 4a). Of note, knockdown of *Lsp2* did not affect adult body weight in nutrient-rich conditions, but reduced it significantly in the context of a low-protein diet, suggesting that Lsp2 acts as a buffering system to maintain growth during limited protein intake (Fig. 4b). In contrast to ePR, *Lsp2* knockdown did not significantly compromise early-adult female fecundity (Extended Data Fig. 6a,b) or ovariole number (Extended Data Fig. 6c), which indicates that the developmental and reproductive deficits induced by ePR are not mediated by Lsp2. In addition, abdominal insulin signalling was unaffected by *Lsp2* knockdown (Extended Data Fig. 6d–g), whereas ATF4 activity was persistently increased during development (Extended Data Fig. 6h,i).

**Extended Data Fig. 7:**
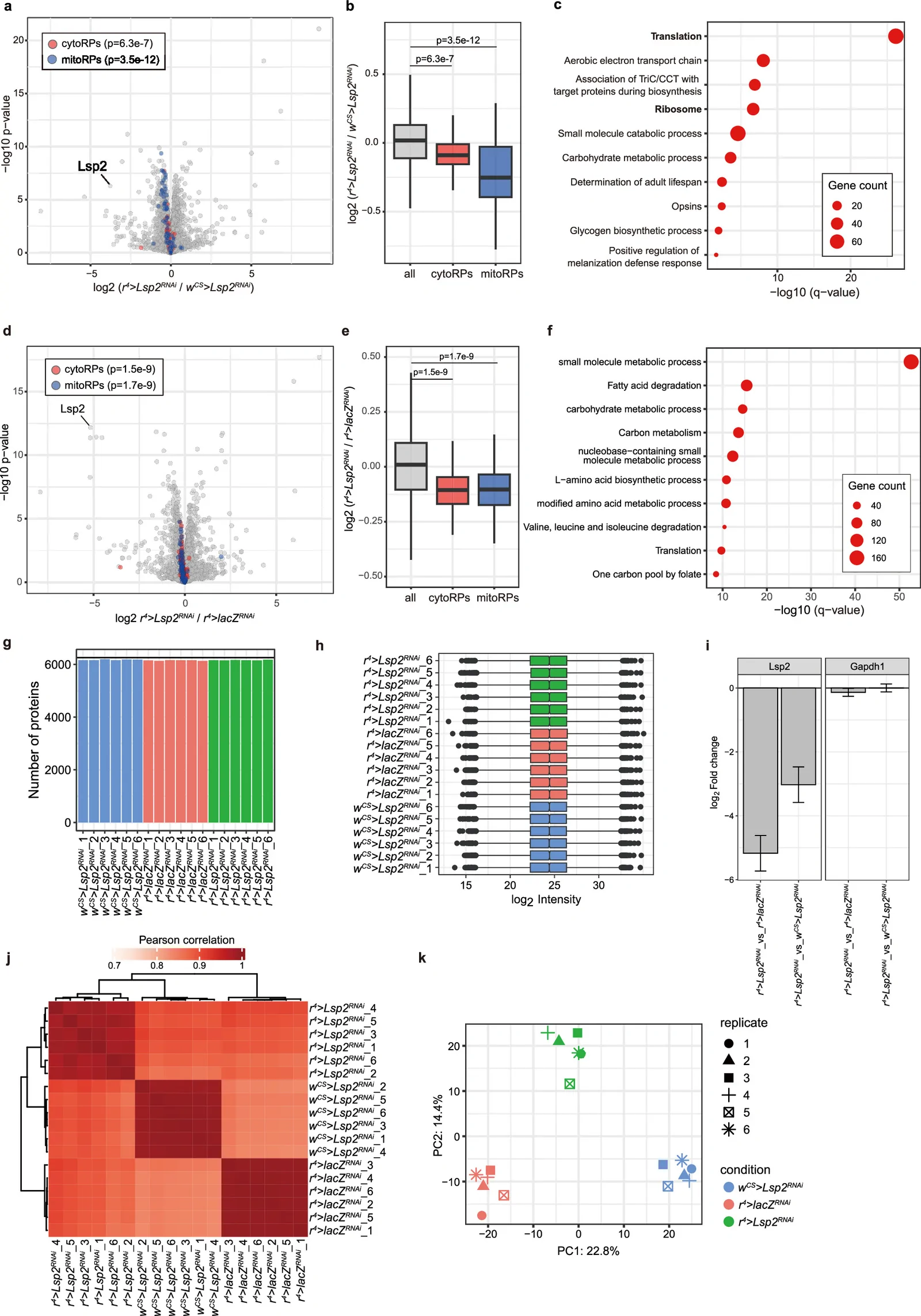
Comprehensive proteomic profiling of adult fly heads after *Lsp2* knockdown. **a**–**c**, Differential protein abundance in adult female heads at day 2 following *Lsp2* knockdown using the *r*^*4*^*-Gal4* driver, compared to the *w*^*CS*^ genetic control. Data are presented as a volcano plot (**a**), a corresponding box plot showing log_2_ fold changes for all proteins (n = 6258), cytoRPs (n = 82), and mitoRPs (n = 72) (**b**), and GO enrichment analysis of significantly downregulated proteins (**c**; *P* < 0.01 and log_2_ *r*^*4*^*>Lsp2*^*RNAi*^ / *w*^*CS*^*>Lsp2*^*RNAi*^ < −0.1). **d**–**f**, Differential protein abundance in adult female heads at day 2 following *Lsp2* knockdown compared to the *UAS-lacZ*^*RNAi*^ control. Data include a volcano plot (**d**), a box plot for specific protein categories (**e**), and GO enrichment analysis of downregulated proteins (**f**; *P* < 0.01 and log_2_ *r*^*4*^*>Lsp2*^*RNAi*^ / *r*^*4*^*>lacZ*^*RNAi*^ < −0.1). **g**,**h**, Quality control metrics for the proteomic dataset, including the number of quantified proteins per sample (**g**) and the distribution of log_2_ protein intensities across replicates (**h**). The number of proteins quantified in each sample: Out of 6,778 total identified proteins, 6,258 proteins were consistently quantified across all six biological replicates in at least one condition and included in the analysis. The number of proteins included in the box plots corresponds to that shown in **g**. **i**, Relative abundance (log_2_ fold change) of averaged levels of Lsp2 and Gapdh1 (control) proteins derived from the proteomic dataset with *Lsp2* knockdown using fat-body driver *r*^*4*^*-Gal4*. Error bars indicate 95% confidence intervals. **j**,**k**, Pearson correlation matrix (**j**) and PCA plot (**k**) demonstrating high reproducibility among biological replicates. Data are presented as box plots (**b**,**e**,**h**) or as mean ± s.d. (**i**). Box plots in **b**,**e** show the median (centre line), the interquartile range (box), and the minimum and maximum values (whiskers). Box plots in **h** show the median (centre line), the interquartile range (box), and the smallest and largest values within 1.5× the interquartile range (whiskers). Values beyond the whiskers are shown as individual points. Symbols represent biological replicates. Statistical significance was determined by a two-sided Wilcoxon rank-sum test (**a**,**b**,**d**,**e**). Source data

**Extended Data Fig. 8:**
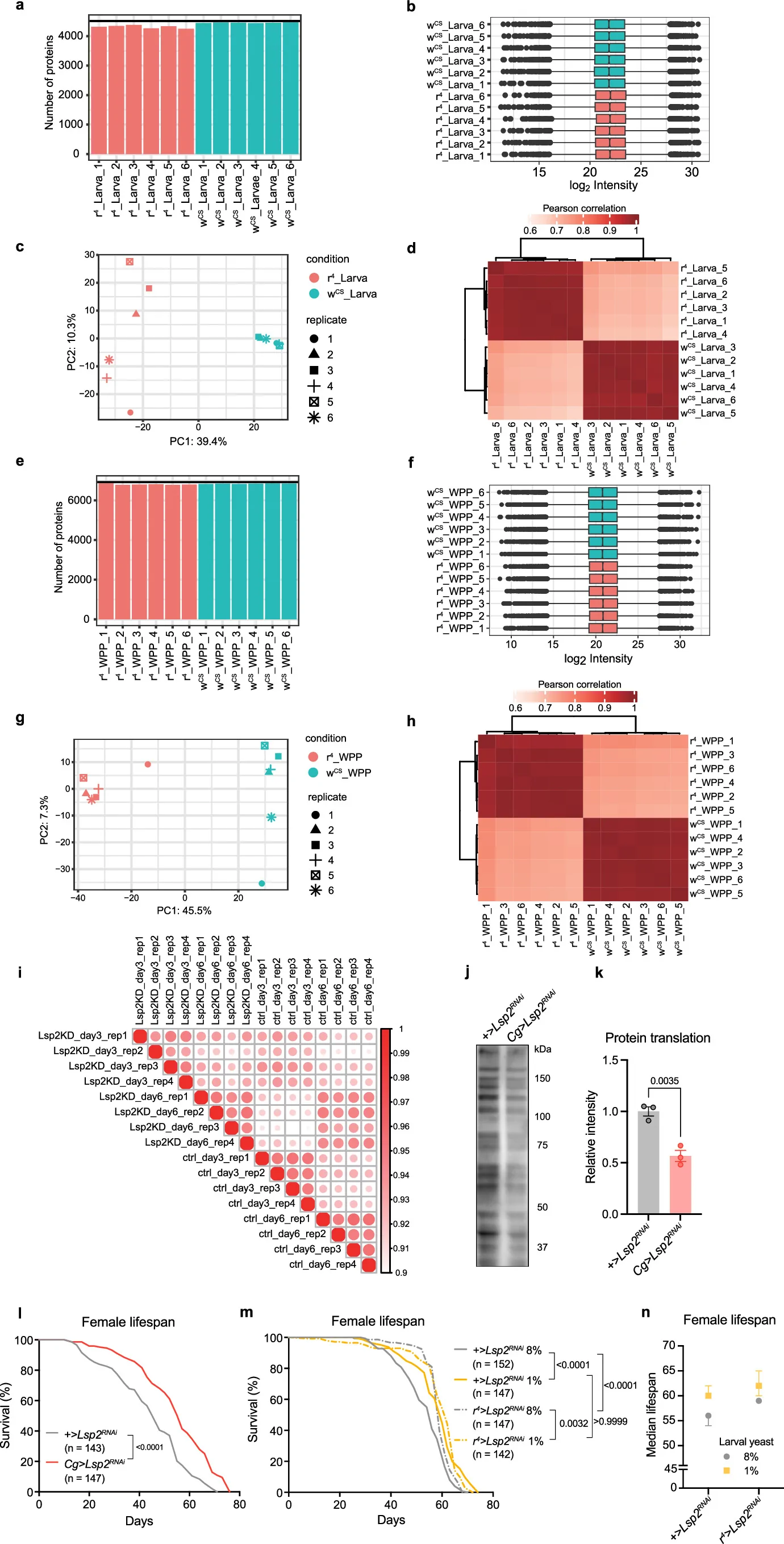
The effect of *Lsp2* knockdown on proteome, translation, and lifespan. **a**–**d**, Quality control metrics for the larval proteome dataset, including the number of quantified proteins (**a**), distribution of log_2_ protein intensities (**b**), PCA plot (**c**) and Pearson correlation matrix (**d**) of individual biological replicates. Proteins quantified across all six replicates in at least one condition were included in the analysis. The number of proteins included in the box plots corresponds to that shown in **a**. **e**–**h**, Quality control metrics for the white prepupal proteome dataset, including the number of quantified proteins (**e**), log_2_ protein intensities (**f**), PCA plot (**g**) and Pearson correlation matrix (**h**). The number of proteins included in the box plots corresponds to that shown in **e**. **i**, Multi-scatter plots showing the correlation of log_2_ (heavy/medium) ratios between biological replicates, demonstrating high experimental reproducibility. **j**,**k**, Representative immunoblot (**j**) and quantification (**k**) of protein synthesis (SUnSET assay) in the heads of female flies at day 2–3 with *Lsp2*-knockdown using fat-body driver *Cg-Gal4*. Anti-puromycin antibody is used. n = 3. **l**, Survival curves of female flies with *Lsp2* knockdown using fat-body driver *Cg-Gal4* or control (*w*^*CS*^). *Cg-Gal4* is backcrossed to *w*^*CS*^ for eight generations. **m**,**n**, Survival curves (**m**) and median lifespan (**n**) of female flies with *Lsp2* knockdown using fat-body driver *r*^*4*^*-Gal4* or control (*w*^*CS*^) combined with ePR. *r*^*4*^*-Gal4* is backcrossed to *w*^*CS*^ for eight generations. Error bars in **n** represent 95% confidence intervals. Sample sizes (n) for lifespan experiments are indicated within the respective panels. Data are presented as box plots (**b**,**f**) or as mean ± s.e.m. (**k**). Box plots in **b**,**f** show the median (centre line), the interquartile range (box), and the smallest and largest values within 1.5× the interquartile range (whiskers). Values beyond the whiskers are shown as individual points. Symbols represent biological replicates. Statistical significance was determined by a two-tailed Student’s *t*-test (**k**) or a log-rank test (**l**–**n**). Source data

To characterize the organismal response to *Lsp2* loss of function, we performed transcriptome, proteome and in vivo pSILAC analyses in *Lsp2*-RNAi flies. As expected, we observed a trend towards the transcriptional suppression of cytoRPs, whereas mitoRP genes remained unaffected (Supplementary Table 4). Proteomic analysis of *Lsp2-*knockdown flies revealed the suppression of both cytoRPs and metabolic proteins (Extended Data Fig. 7a–k and Supplementary Table 3), which is reminiscent of the effects of ePR. Unlike what is seen with ePR, mitoRPs are also downregulated by *Lsp2*-RNAi (Extended Data Fig. 7a,b,d,e), which is also reported in the accompanying paper^47^. Notably, *Lsp2* knockdown did not affect the levels of cytoRPs during the larval stage, but it reduced them by the prepupal stage, suggesting that the Lsp2-dependent regulation of cytoRPs begins during metamorphosis (Fig. 4c–f, Extended Data Fig. 8a–h and Supplementary Table 3). Consistent with this, in vivo pSILAC analysis revealed that larva-derived cytoRPs were decreased by *Lsp2* knockdown, whereas adult-derived cytoRPs seemed instead to be increased (Fig. 4g,h, Extended Data Fig. 8i and Supplementary Table 2). This increase could be due to increased food intake or the compensatory incorporation of adult-derived amino acids. *Lsp2* knockdown also decreased the levels of larva-derived mitoRPs (Fig. 4h). Therefore, its effect on the translation process might be more extensive than that of ePR.

Like ePR-flies, *Lsp2* knockdown flies exhibited suppressed translation activity in early adulthood (Extended Data Fig. 8j,k). Consistently, knockdown of *Lsp2* extended the adult lifespan of female flies (Extended Data Fig. 8l–n). The lifespan extension observed with *Lsp2*-RNAi was comparable with that induced by ePR, and ePR did not further increase lifespan when combined with *Lsp2*-RNAi (Extended Data Fig. 8m,n), which suggests that the two manipulations share a mechanism of lifespan extension. Together, these findings indicate that Lsp2 has a central role in mediating the effects of ePR on adult translation and lifespan, but not growth, fecundity or insulin signalling.

**Extended Data Fig. 9:**
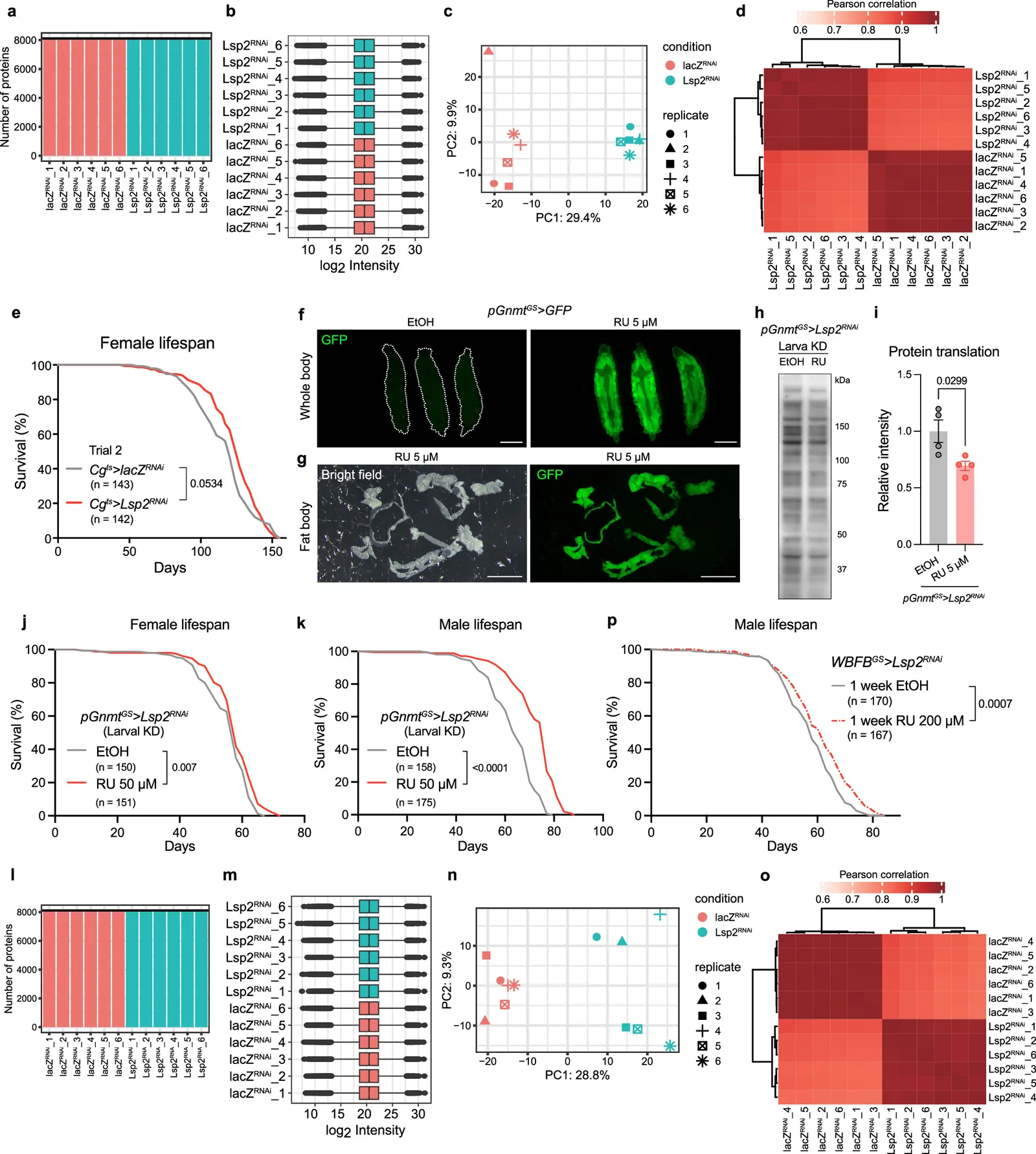
Effects of time-restricted *Lsp2* knockdown on proteome and lifespan. **a**–**d**, Quality control metrics for the proteomic dataset of heads of adult female flies at day 4 with larval and pupal-specific *Lsp2* knockdown using fat-body driver *Cg-Gal4* combined with *tub-Gal80*^*ts*^. Data include the number of quantified proteins (**a**), distribution of log_2_ protein intensities (**b**), PCA plot (**c**) and Pearson correlation matrix (**d**). The number of proteins included in the box plots corresponds to that shown in **a**. **e**, Survival curves of female flies with larval-specific *Lsp2* knockdown using fat-body driver *Cg-Gal4* combined with *tub-Gal80*^*ts*^. *UAS-lacZ*^*RNAi*^ and *UAS-Lsp2*^*RNAi*^ are backcrossed to *w*^*CS*^ flies for eight generations. **f**,**g**, Representative fluorescence images of whole larvae (**f**) and larval fat body (**g**) showing RU486-induced GFP expression using the *pGnmt*^*GS*^ driver. Scale bar = 1 mm. The similar result was repeatedly obtained at least twice. **h**,**i**, Representative immunoblot (**h**) and quantification (**i**) of protein synthesis (SUnSET assay) in the heads of female flies at day 2–3 with larval-specific *Lsp2* knockdown using larval fat-body driver *pGnmt*^*GS*^. Anti-puromycin antibody is used. n = 3. **j**,**k**, Survival curves of female (**j**) or male (**k**) flies with larval-specific *Lsp2* knockdown using larval fat-body driver *pGnmt*^*GS*^. **l**–**o**, Quality control metrics for the proteomic dataset of adult female heads at day 2 with adult-specific *Lsp2* knockdown (day 0–2) using fat-body driver *Cg-Gal4* combined with *tub-Gal80*^*ts*^. Data include the number of quantified proteins (**l**), distribution of log_2_ protein intensities (**m**), PCA plot (**n**) and Pearson correlation matrix (**o**). The number of proteins included in the box plots corresponds to that shown in **l**. **p**, Survival curves of male flies with adult-specific *Lsp2* knockdown using adult fat-body driver *WBFB*^*GS*^ during the first week of adulthood. Sample sizes (n) for lifespan experiments are indicated within the respective panels. Data are presented as box plots (**b**,**m**) or as mean ± s.e.m. (**i**). Box plots in **b**,**m** show the median (centre line), the interquartile range (box), and the smallest and largest values within 1.5× the interquartile range (whiskers). Values beyond the whiskers are shown as individual points. Symbols represent biological replicates. Statistical significance was determined by a log-rank test (**e**,**j**,**k**,**p**) or a two-tailed Student’s *t*-test (**i**). Source data

Although *Lsp2* knockdown and ePR are likely to share their mechanism of lifespan extension, whether early-life Lsp2 alone is sufficient to regulate lifespan remains unclear. Depleting larva-derived Lsp2 by using *Gal80*^*ts*^ (*Cg*^*ts*^*>Lsp2*^*RNAi*^) reduced both cytoRP levels and translation activity in early adulthood (Fig. 4i–l, Extended Data Fig. 9a–d and Supplementary Table 3), and was sufficient to extend female lifespan (Fig. 4m and Extended Data Fig. 9e). To exclude potential genetic background issues, we generated a *pGnmt-GeneSwitch* (*pGnmt*^*GS*^) fly line by using the promoter sequence of the metabolic gene *Gnmt*, which is highly expressed in fat bodies^48^. Crossing this line with *UAS-GFP* confirmed that the expression of *pGnmt*^*GS*^ was almost entirely confined to adipose tissue and was RU486-inducible (Extended Data Fig. 9f,g). As expected, knocking down *Lsp2* in larvae using *pGnmt*^*GS*^ decreased translation in female flies (Extended Data Fig. 9h,i) and extended the lifespan of both males and females (Extended Data Fig. 9j,k), although the suppression of translation was weaker than that seen with the *Cg*^*ts*^ driver (70% versus 47% of control). It is possible that, because wandering larvae were not fed RU486-containing food, the phenotype was not as strong as that achieved by *Lsp2* knockdown throughout the larval to pupal stages. Nevertheless, these results prove that decreasing Lsp2 levels only in the larval stage suppresses protein translation and extends lifespan.

Considering that larval *Lsp2* regulates adult *Lsp2* expression through a feed-forward response (Fig. 3h–j), it remained possible that decreasing Lsp2 protein specifically during the early adult stage could be sufficient to suppress translation capacity and extend lifespan. To test this possibility, we performed early-adult-specific *Lsp2* knockdown in the adult fat body. Notably, proteome analysis suggested that cytoRPs were not decreased by adult-specific *Lsp2* knockdown (Fig. 4n,o, Extended Data Fig. 9l–o and Supplementary Table 3). In this experiment, the decrease in Lsp2 was not as strong as that seen with developmental *Lsp2* knockdown, further confirming that larval carryover Lsp2 is still present in early adulthood (Fig. 4n). However, this manipulation resulted in a marked decrease in translation (Fig. 4p,q). This indicates that Lsp2 protein in early adulthood regulates translation independently of cytoRP abundance. In the control-diet condition, fat-body-specific knockdown of *Lsp2* restricted to the first week of adulthood using *WBFB-GeneSwitch* (*WBFB*^*GS*^) significantly extended female lifespan (Fig. 4r; 8% ethanol versus 8% 1 week RU486 (hereafter, RU)). Early-adult *Lsp2* knockdown also extended lifespan in male flies (Extended Data Fig. 9p). Treating flies with RU for their entire adult life led to an increase in lifespan that was similar to that observed with 1 week RU (Fig. 4r; 8% 1 week RU versus 8% RU). There was no statistical significance between adult-lifelong *Lsp2* knockdown (8% RU) and early-adult-specific *Lsp2* knockdown (8% 1 week RU), although median lifespan for the 8% RU condition (67 days) was greater than that for the 8%-1-week-RU condition (63 days) (Fig. 4r). As expected, ePR alone extended female lifespan in the absence of RU feeding (Fig. 4r; 8% ethanol versus 1% ethanol), and adult-lifelong *Lsp2* knockdown did not extend lifespan any further (Fig. 4r; 1% ethanol versus 1% RU), which suggests that ePR extends lifespan via Lsp2. By contrast, when ePR was combined with early-adult-specific *Lsp2* knockdown, lifespan was shortened (Fig. 4r; 1% ethanol versus 1% 1 week RU). We assume that the combination of ePR and transient suppression of *Lsp2* during early adulthood renders flies susceptible to the life-shortening effects of Lsp2 that emerge later in life once Lsp2 expression is restored.

## Lsp2-rich amino acids shape longevity

**Fig. 5:**
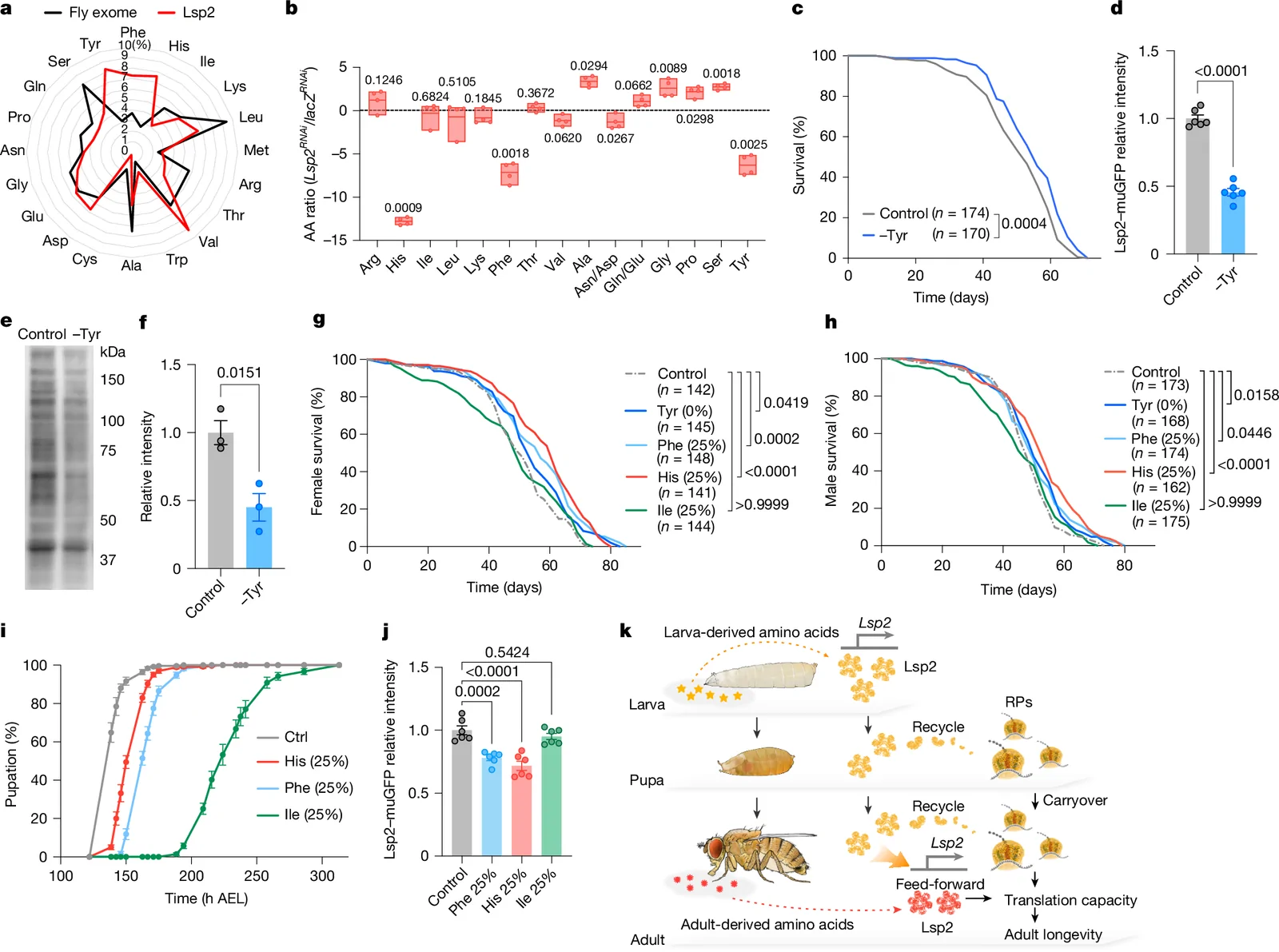
Restriction of Lsp2-enriched amino acids at the larval stage extends lifespan. **a**, Comparison of amino acid composition between the Lsp2 protein and the total *Drosophila* exome. **b**, Total amino acid composition (free and non-free amino acids) in adult female flies at day 2 after *Lsp2* knockdown using the fat-body driver *r*^*4*^*-Gal4*. *n* = 4. **c**–**f**, Larval-specific dietary Tyr restriction using a synthetic diet (holidic medium). **c**, Survival curves of Canton-S female flies. **d**, Quantification of Lsp2–muGFP reporter activity in wandering larvae. *n* = 10. **e**,**f**, Representative immunoblot (**e**) and quantification (**f**) of protein synthesis (SUnSET assay) in the heads of Canton-S female flies at day 2–3. *n* = 3. **g**–**i**, Larval-specific dietary restriction of Tyr, Phe, His or Ile using another synthetic diet (HolFast). Whereas the non-essential amino acid Tyr can be depleted, the essential amino acids Phe, His and Ile are decreased to 25% of the control diet. Ctrl, control. **g**,**h**, Survival curves of Canton-S female (**g**) and male (**h**) flies. **i**, Developmental speed of Canton-S flies. *n* = 10. **j**, Quantification of Lsp2–muGFP reporter activity in wandering larvae. *n* = 6. **k**, Schematic model illustrating how early-life diet regulates adult translational capacity and longevity through Lsp2. Sample sizes (*n*) for lifespan experiments are indicated. Data are box plots showing the median, quartiles and range (**b**) or are mean ± s.e.m. (**d**,**f**,**i**,**j**). Symbols represent biological replicates. Statistical significance was determined by two-tailed Student’s *t***-**test (**b**,**d**,**f**), log-rank test (**c**,**g**,**h**) or one-way ANOVA with Dunnett’s multiple comparison test (**j**). Source data

Lsp2 belongs to the arylphorin class of proteins, which is uniquely enriched in aromatic amino acids. Specifically, the Lsp2 protein contains 1.97-fold more Phe and 2.66-fold more Tyr than the average fly protein does (the exome)^25^ (Fig. 5a). Consequently, continuous knockdown of *Lsp2* in the fat body significantly reduced the overall composition of these amino acids in young adults (Fig. 5b). The amino acid profile of *Lsp2*-RNAi flies mirrors the altered composition of Phe and Tyr that is seen in ePR-flies (Extended Data Fig. 3f,g). These data suggest that a reduction in Lsp2 could be responsible for the shift in amino acid composition that ePR induces. Histidine is also enriched in Lsp2 protein, and *Lsp2*-RNAi reduced the total levels of histidine in the body (Fig. 5a,b). In ePR-flies, however, histidine levels were not decreased, suggesting that ePR has an effect on histidine levels independent of Lsp2 (Extended Data Fig. 3f,g).

We previously found that *Drosophila* is highly sensitive to Tyr intake, even though Tyr is a non-essential amino acid^32,49^. Indeed, when larvae were fed a synthetic diet specifically lacking Tyr from 68 h AEL, they developed into adults that were notably long-lived (Fig. 5c). Consistently, Lsp2 expression and protein translation in early adulthood was suppressed by larval Tyr deprivation (Fig. 5d–f). To test amino acid specificity, we restricted other amino acids. Because complete restriction of essential amino acids prevents adult emergence, and because the synthetic diet used above for Tyr deprivation causes semi-lethality, we used the larva-optimized HolFast diet to decrease essential amino acids, such as Phe, His and isoleucine (Ile), to 25%. We confirmed that Tyr deprivation also extends lifespan in this experimental diet (Fig. 5g,h). Restriction of Lsp2-enriched amino acids, such as Phe and His, increased lifespan in both females and males (Fig. 5g,h). By contrast, restricting Ile, which is less abundant in Lsp2, did not extend lifespan (Fig. 5g,h). Ile restriction induced a stronger developmental delay than did Phe or His restriction, potentially compromising health later in life (Fig. 5i). As expected, we found that the GFP fluorescence of Lsp2–muGFP decreased for restriction of Phe or His, but not for Ile restriction (Fig. 5j). Together, these data suggest that larval intake of dietary amino acids that are enriched in Lsp2 regulates the abundance of Lsp2 protein in early life stages, and that this, in turn, modulates adult translational capacity and lifespan (Fig. 5k).

## Discussion

This study reveals a mechanism through which early-life dietary manipulation regulates adult physiology and lifespan. Using a synthetic diet, we performed in vivo pSILAC analysis, in which we used isotope-labelled amino acids to trace dietary amino acids under physiological conditions. This nutrient-tracing technique enables the fate of juvenile dietary components during the life course of a fly to be ascertained. We combined in vivo pSILAC with nutritional or genetic manipulation, revealing adult proteome turnover in high resolution, and showing how it is affected by early-life diet.

Lsp2 functions as a central integrator of nutritional history that links dietary input to translational control. Lifespan extension induced by both ePR and *Lsp2* knockdown is observed in both sexes, indicating that this effect is independent of sex-specific reproductive investment. Although ePR reduces systemic growth, fecundity, ovariole number and insulin signalling activity, these phenotypic outcomes are not recapitulated by *Lsp2* knockdown. These data imply that ePR-induced longevity is not driven simply by the reproduction–lifespan trade-off. In parallel, an accompanying study identifies Lsp2 as a modifier of mTORC1 that regulates translation and lifespan without compromising fecundity, providing further support for the idea that Lsp2 has a central role in longevity control^47^.

As a highly abundant storage protein with dynamic turnover in early life stages, Lsp2 serves not only as an amino acid reservoir but also as a persistent molecular readout of nutritional conditions. Because the levels of Lsp2 are regulated through a feed-forward mechanism, its abundance reflects cumulative rather than transient dietary input, enabling sustained physiological responses to fluctuating environments. This property positions Lsp2 as a molecular substrate for predictive adaptive responses^50,51^, effectively encoding ‘nutritional memory’ across developmental transitions. The amino acid composition of Lsp2 might confer a selective buffering capacity; for example, adult flies are resistant to deprivation of Phe but not to that of any other essential amino acids, potentially reflecting the enrichment of Phe and Tyr in Lsp2 (ref. ^52^). It is noteworthy that restricting these selected amino acids at early life stages triggers Lsp2 loss and concomitant lifespan extension. Researchers need to take the quantity and quality of storage proteins into account when considering an animal’s nutritional response.

Lsp2 regulates translation through multiple stage-dependent mechanisms. During the pupal stage, when feeding is absent, Lsp2 probably supports translation by directly supplying amino acids, thereby sustaining the synthesis of high-demand proteins. CytoRPs are among the most abundant proteins, and are therefore particularly sensitive to this supply. Fat body protein 1 (Fbp1), one of the storage proteins required for the re-uptake of Lsp proteins into the fat body for amino acid utilization, is induced just before pupariation^43^. Consistently, a reduction in the abundance of cytoRPs was already detectable at the prepupal stage after *Lsp2* knockdown. These findings suggest that storage-derived amino acids are mobilized early and are preferentially allocated to sustain core translational machinery. In early adulthood, Lsp2 continues to support translation, although not exclusively through cytoRP abundance, indicating that there are additional regulatory layers. It is possible that the direct provision of stored amino acids to fuel translation is crucial during this period, although the possibility that Lsp2 also functions as a nutrient signal cannot be excluded. In later adult stages, Lsp2 shifts towards regulating translation efficiency through the mTORC1–4E-BP axis, thereby controlling the translation of 5′-terminal oligo pyrimidine (TOP) mRNAs, including those that encode cytoRPs^47^. Of note, the turnover rate of cytoRPs is known to be associated with ageing and lifespan in various organisms^53,54^. Together, these findings support a model in which Lsp2 coordinates translational control across developmental stages, ultimately influencing organismal lifespan.

The mechanism by which Lsp2-derived amino acids are mobilized remains incompletely understood. Lsp2 could be internalized and degraded through endosomal–lysosomal pathways, generating localized amino acid pools^43,45,55^. In this context, amino acids derived from storage proteins might not be uniformly used for protein synthesis. We therefore speculate the existence of a ‘recyclome’, defined as a subset of proteins that are preferentially synthesized from recycled amino acids rather than from newly imported ones. Given the central role of lysosomes in amino acid sensing and mTORC1 activation, such compartmentalized recycling could selectively influence the translation of nutrient-sensitive transcripts, including TOP mRNAs encoding cytoRPs. Notably, pharmacological inhibition of mTORC1 by rapamycin extends lifespan across species, with particularly strong effects when administered during early life stages, including larval development and early adulthood^56,57^. This temporal sensitivity parallels the developmental window during which Lsp2 regulates translation, raising the possibility that early-life interventions converge on a shared axis of translational control.

Epidemiological studies have established that early-life nutrition has profound and lasting effects on human health, a concept formalized as the Developmental Origins of Health and Disease (DOHaD)^14,16^. Although mammals lack a direct orthologue of Lsp2, functionally analogous systems might exist. Circulating, highly abundant proteins such as albumin and globulins could have similar roles. For example, albumin and Lsp2 share key characteristics, such as detoxification through the binding of low-solubility compounds; regulation of osmolality and nutritional status; and high synthesis and turnover rates in the liver, with sensitivity to dietary protein intake^58^. Rather than direct molecular conservation, the concept of a protein-based reservoir that buffers amino acid availability and encodes nutritional memory might be evolutionarily conserved. Our findings raise the possibility that storage proteins act as mediators of DOHaD, linking early-life diet to long-term physiological outcomes.

## Methods

### *Drosophila* stocks and husbandry

Stock flies and adult flies were reared on a standard yeast-based diet containing 4.5% cornmeal (Nippn Corporation), 6% brewer’s yeast (Asahi Breweries, HB-P02), 6% glucose (Nihon Shokuhin Kako) and 0.8% agar (Ina Food Industries, S-6) with 0.4% propionic acid (FUJIFILM Wako, 163-04726) and 0.15% butyl *p*-hydroxybenzoate (FUJIFILM Wako, 028-03685), unless otherwise stated. Flies were maintained at 25 °C. To allow synchronized development and constant density, embryos were collected using agar plates (3% agar, 1% sucrose and 0.3% acetic acid) with a live yeast paste and 10–15 μl of the embryos were spread onto bottles.

Fly lines used in this study were Canton-S, *w*^*CS*^ (*w*^*iso31*^ backcrossed to Canton-S for eight generations), *4E-BP*^*intron*^*-dsRed* (ref. ^33^), *Cg-Gal4* (Bloomington Drosophila Stock Center (BDSC), 7011), *tub-Gal80*^*ts*^ (BDSC, 7017), *r*^*4*^*-Gal4* (BDSC, 33832), *fit-Gal4* (ref. ^30^), *WBFB*^*GS*^ (ref. ^59^), *UAS-2*×*EGFP* (BDSC, 6874), *UAS-lacZ-RNAi* (from R. Carthew), *UAS-Lsp2-RNAi* (National Institute of Genetics, 6806R-2), *UAS-Lsp1α-RNAi* (Vienna Drosophila Resource Center (VDRC), 14898), *UAS-Fbp2-RNAi* (VDRC, 33173), *Lsp2-muGFP* (this study) and *pGnmt*^*GS*^ (this study). *WBFB*^*GS*^ was used as the adult fat-body driver, whereas *pGnmt*^*GS*^ was used as the larval fat-body driver. This selection was based on the observation that *WBFB*^*GS*^ exhibits significant leakiness during the larval stage^60^, whereas *pGnmt*^*GS*^ has weaker expression in the adult stage. *Cg-Gal4*, *r*^*4*^*-Gal4*, *UAS-lacZ-RNAi*, *UAS-Lsp2-RNAi*, *pGnmt*^*GS*^ are backcrossed to *w*^*CS*^ for eight generations.

To generate *Lsp2-muGFP* flies, the CRISPR–Cas9 system was used to insert *muGFP* at the C terminus of the *Lsp2* gene^61^. The *muGFP* codons were optimized for expression in *Drosophila melanogaster*. These modifications and insertions into the EcoRI/XbaI site of the pUC57 vector were performed by GenScript. An sgRNA target site of *Lsp2* was selected using CRISPR Optimal Target Finder^62^. Complementary oligonucleotides with overhangs were annealed and cloned into the BbsI-digested U6b vector using a DNA ligation kit (Takara, 6023).

Sense strand: 5′-TTCGGTCCAGGATCTAGACCACAT-3′

Antisense strand: 5′-AAACATGTGGTCTAGATCCTGGAC-3′

The targeting vector was constructed by inserting a linker sequence (GGTGGATCTGGAGGTTCCGGCGGCTCAGGGGGTAGT) and *muGFP* between 500-bp *Lsp2* homology arms, incorporating a silent mutation in the PAM sequence adjacent to the sgRNA site (TGG to TTG). The linker sequence, *muGFP* and homology arms were PCR-amplified using Q5 High-Fidelity 2× Master Mix (New England BioLabs, M0492L). PCR primers were designed using the NEBuilder Assembly Tool. The gel-purified PCR products were cloned into the EcoRI-digested pBluescript II SK(+) vector using NEBuilder HiFi DNA Assembly Master Mix (New England BioLabs, E2621X). The mixture of pU6b-sgRNA and targeting vector was microinjected into *w*^*1118*^*; attP40{nos-Cas9}/CyO* embryos by WellGenetics. F_0_ adults were crossed with balancer lines, and lines with the correct *muGFP* insertion were identified by PCR amplification and sequencing of the target locus. The primers used for PCR are listed in Supplementary Table 6.

To generate* pGnmt*^*GS*^, a 500-bp fragment upstream of the start *Gnmt* codon and the GeneSwitch sequences were PCR-amplified using PrimeSTAR Max DNA Polymerase (Takara, R045A). PCR primers were designed using the NEBuilder Assembly Tool. The gel-purified PCR products were cloned into the KpnI-digested pElav-GeneSwitch vector using NEBuilder HiFi DNA Assembly Master Mix (NEB, E2621L). The resulting plasmid was injected into *w*^*1118*^ embryos (WellGenetics). F_0_ adults were crossed with balancer lines, and *w*+ lines were selected. PCR primer sequences are provided in Supplementary Table 6. Larval expression is restricted almost entirely to the fat body, although additional expression was observed in other tissues, such as small cells within the gut (possibly enteroendocrine cells).

### Dietary manipulations

For larval yeast manipulation, diets containing 8%, 2% and 1% yeast were prepared using baker’s yeast (Lesaffre, Saf-Instant Red), 6% glucose (FUJIFILM Wako, 049-31165), 1% agar (FUJIFILM Wako, 010-15815), 0.3% propionic acid (FUJIFILM Wako, 163-04726) and 0.15% methyl *p*-hydroxybenzoate (FUJIFILM Wako, 132-02635). Cornmeal was omitted from these formulations to simplify food preparation, facilitate precise nutritional manipulation and avoid compositional variability arising from batch-to-batch differences in cornmeal. Increasing the yeast content to 8% (from the 6% used in the standard diet) resolved the slight developmental delay associated with the omission of cornmeal; consequently, the 8% yeast diet was used as the control (fully fed) condition. Embryos were collected within four hours of egg-laying in a cage (Flystuff, 59-101 or 59-100). The embryos (10–15 μl) were spread by micropipette onto a surface of 8% yeast diet and raised until the late-second-instar larval stage. At around 68 h AEL, the larvae were floated up using 30% glycerol and transferred to each diet. Normally, 150–200 adult flies were obtained per bottle. After eclosion, adult flies were collected to a standard yeast-based diet.

Chemically defined diet, specifically holidic medium^24^ or HolFast^27^, was used for amino acid manipulation. Amino acid concentrations of the holidic medium were optimized by exome matching^25^. For larval dietary manipulation using the holidic medium, the sugar source (glucose rather than sucrose) and the amount of agar and preservatives were modified according to a previous study when used for larval dietary manipulation^32^. Embryos were distributed by micropipette onto the surface of a standard yeast-based diet and reared until the late-second-instar larval stage. At approximately 68 h AEL, larvae were collected by flotation using 30% glycerol and transferred to either the holidic medium or the HolFast diet. After eclosion, adult flies were transferred back to a standard yeast-based diet. For adult dietary manipulation, ePR-flies were fed the holidic medium containing different amount of amino acid.

### Analysis of developmental speed, eclosion rate and body weight

After ePR treatment, larvae were picked up and transferred onto bottles or vials and the pupal number was counted every several hours. The number of eclosed adult flies per pupal number was calculated as eclosion rate. For the body weight measurement, each single fly was anaesthetized by CO_2_ and placed onto a microbalance (METTLER TOLEDO, XPR2).

### Lifespan analysis

Adult flies were allowed to mate in bottles for two days post-eclosion. Subsequently, 25 females or 30 males were allocated to each vial. The flies were maintained at 25 °C under 60% humidity and a 12 h light:12 h dark cycle. For time-restricted knockdown analysis with *Gal80*^*ts*^, flies were maintained at 18 °C or 29 °C. Flies were transferred to fresh vials two to three times per week, during which the number of dead or censored individuals was recorded. Survivability was calculated by OASIS2 (ref. ^63^) and the survival curve was replotted using GraphPad Prism 10 or 11. Survival analysis was performed by R using the survival package^64^. Median lifespan with 95% confidence intervals was estimated from the Kaplan–Meier model. To evaluate the effects of experimental factors and their interactions on mortality risk, Cox proportional hazards models including interaction terms were fitted where appropriate. Hazard ratios (HRs) and 95% confidence intervals (95% CIs) were estimated from the fitted models. Log-rank tests were used for survival curve comparisons.

### Fecundity analysis

Adult flies were allowed to mate in bottles for two days after eclosion. Subsequently, flies were anesthetized quickly with CO_2_ and allocated to ten vials containing each diet with five females and five males per vial. The number of eggs laid on the medium was manually counted per day. The number of ovarioles was counted by pulling out each ovariole from ovaries using forceps and averaging the left and right ovariole numbers.

### Western blot analysis

The abdominal carcasses from 8 female flies or heads from 12 female flies were dissected in phosphate-buffered saline and homogenized in 50 μl RIPA buffer (FUJIFILM Wako, 188-02453) supplemented with a protease inhibitor (FUJIFILM Wako, 165-26021) and phosphatase inhibitor cocktails (Roche, 4906845001). The gut, Malpighian tubules and ovaries were removed from the adult abdomen to prevent contamination. The supernatant was collected after centrifugation and protein was quantified by bicinchoninic acid (BCA) assay (FUJIFILM Wako, 164-25935). The samples were mixed with 6× SDS–PAGE sample buffer (Nacalai, 09499-14) and 15–20 μg proteins were subjected to standard SDS–PAGE. Gels were transferred to a PVDF membrane and blocked by EveryBlot blocking buffer (Bio-Rad, 12010020). Primary antibodies used in the study were anti-α + β-tubulin (Abcam, ab44928, 1:1,000 dilution), anti-histone H3 (CST, 14269S, 1:1,000 dilution), anti-phospho-Akt (CST, 4060T, 1:1,000 dilution), anti-total Akt (CST, 9272S, 1:1,000 dilution), anti-GFP (Nacalai, 04404-26, 1:1,000 dilution) and anti-Lsp2 (1:1,000 dilution)^43^. Horseradish peroxidase (HRP)-conjugated secondary antibodies were anti-mouse IgG, HRP-linked antibody (CST, 7076S, 1:1,000 dilution), anti-rabbit IgG, HRP-linked antibody (CST, 7074S, 1:1,000 dilution), and anti-rat IgG, HRP-linked antibody (Jackson ImmunoResearch, 81211, 1:1,000 dilution). The signals were visualized by chemiluminescence using Immobilon (Millipore, WBLUF0100) and detected by Amersham ImageQuant 800 (Cytiva). Uncropped images are shown in Supplementary Fig. 1.

### Protein synthesis assay

Protein synthesis was monitored using the SUnSET assay^65^. Adult flies were fed 600 μM puromycin (FUJIFILM Wako, 160-23154) in a standard diet for 24 h. Twelve heads of female flies were collected and lysed in RIPA buffer (FUJIFILM Wako, 188-02453) supplemented with a protease inhibitor (FUJIFILM Wako, 165-26021). Twenty micrograms of protein samples was mixed with 6× SDS–PAGE sample buffer and subjected to the standard SDS–PAGE technique using a 5–20% gradient gel (FUJIFILM Wako, 194-15021). Gels were transferred to a PVDF membrane and blocked by EveryBlot blocking buffer (Bio-Rad, 12010020). Anti-puromycin antibody (Millipore, MABE343, 1:1,000 dilution) and anti-mouse IgG2a, HRP-linked antibody (Jackson ImmunoResearch, 115-035-206, 1:15,000 dilution) were used. The signals were visualized by chemiluminescence using Immobilon (Millipore, WBLUF0100) and detected by Amersham ImageQuant 800 (Cytiva).

### RNA-seq and qRT–PCR analysis

For RNA-seq analysis, we dissected 12 heads and 8 abdominal carcasses from female flies. The gut, Malpighian tubules and ovaries were removed from the abdomen to prevent contamination. Total RNA was purified from the samples using a ReliaPrep RNA Tissue Miniprep kit (z6112, Promega). Four samples were prepared for each experimental group. Library preparation was performed by the RIKEN BDR Technical Support Facility using the Illumina Stranded mRNA Prep Ligation kit (96 samples) (20040534, Illumina K.K.) and IDT for Illumina RNA UD Indexes Set C Ligation kit (20091659, Illumina K.K.). The optimum number of PCR cycles was determined by qPCR using KAPA SYBR FAST qPCR Master Mix (KK4603, Roche). The library quality was verified using the TapeStation HS D1000 assay. The library samples were forwarded to Azenta for RNA-seq using an Illumina NovaSeq 6000 (Illumina K.K.). The paired-end 150 bp sequence data were analysed as follows: a quality check of the raw reads was performed by FastQC (v.0.12.1)^66^ and MultiQC (v.1.24)^67^. The raw reads were then filtered to remove the first base (T), adaptors and low-quality bases using Trim Galore (v.0.6.10)^68^. Filtered reads were aligned to the *Drosophila* genome (BDGP6.46) using Hisat2 (v.2.2.1)^69^. The read counts were calculated using StringTie (v.2.2.1)^70^. Differentially expressed genes were identified using edgeR (v.4.0.6)^71^. RNA-seq data have been deposited at the DDBJ under accession numbers DRR622306–DRR622313 and DRR626770–DRR626793.

For qRT–PCR analysis, total RNA was purified from six heads or eight abdominal carcasses of female flies, or four whole bodies of larvae or pupae as described above using a ReliaPrep RNA Tissue Miniprep kit (z6112, Promega). The gut, Malpighian tubules and ovaries were removed from the adult abdomen to prevent contamination. The cDNA was synthesized from 500 ng of DNase-treated total RNA using Revertra Ace Master mix (FSQ-201, Toyobo). qRT–PCR was performed using Taq Pro Universal SYBER qPCR Master Mix (Q712-02-AA, Vazyme Biotech) and qTOWER^3^ G (Analytik Jena). The ΔΔCt method was used, with *RNA pol2* as the internal control. Primer sequences are listed in Supplementary Table 6.

### Imaging analysis

For whole-body reporter fluorescence, flies were immobilized on a CO_2_ pad and dsRed or GFP fluorescence images were captured using a fluorescence stereomicroscope (MZ10F, Leica Microsystems). To analyse ATF4 reporter fluorescence in the abdomen, flies were imaged from the lateral side to minimize the interference of the basal fluorescence in the gut and Malpighian tubules. A region between stripes of the dorsal abdomen was selected as region of interest (ROI) and the fluorescence was quantified using Fiji software^72^. The fluorescence intensity of the *fit*>*GFP* reporter was quantified by measuring the fluorescence of the entire abdomen, delineated by elliptical selections using the Fiji software package^72^. The fluorescence intensity of the Lsp2–muGFP reporter was quantified by measuring the fluorescence of whole bodies of larvae, pupae and adults.

### Quantification of total amino acids

Four whole flies were collected in each crimp glass vial (Crimp Top Vials, 03-CVG, Chromacol) after body weights were measured using a microbalance (METTLER TOLEDO, XPR2). Four crimp glass vial samples were prepared for each condition and stored at −80 °C. The amino acid amount was normalized by the body weight of the flies. After vacuum drying, each crimp vial was placed in borosilicate glass vials (224832, Wheaton), and 200 μl of 6 M HCl and a small phenol crystal were then added to the outside of the crimp vials. For tryptophan analysis, 4 M methanesulfonic acid with 0.2% (w/v) tryptamine (FUJIFILM Wako) was added to the crimp vials instead of the HCl solution. After evacuating for a few minutes, the vial was sealed with a Mininert valve (SC-24, 10130, Pierce) and heated in a heating bath at 110 °C for 20 h. All procedures for amino acid analysis using precolumn derivatization with 6-aminoquinolyl-*N*-hydroxysuccinimidyl carbamate have been described previously^73^.

### In vivo pulse SILAC analysis

For isotope labelling, larvae were fed a complete HolFast diet^27^ with lysine and arginine substituted with heavy-isotope-labelled l-lysine·2HCl (^13^C_6_, 99%; ^15^N_2_, 99%, +8, Cambridge Isotope Laboratories (CIL), CNLM-291-H-0.1) and l-arginine·HCl (^13^C_6_, 99%; ^15^N_4_, 99%, +10, CIL, CNLM-539-H-0.1). These amino acids are recognized and cleaved by trypsin, resulting in peptides that terminate with a single lysine or arginine residue at their C terminus. Subsequent to eclosion, adult flies were transferred to holidic medium^24^ (exome-matched version^25^) with lysine and arginine substituted with medium isotope-labelled l-lysine·2HCl (4,4,5,5-D_4_, 96%, +4, CIL, DLM-2640-0.1) and l-arginine·HCl (^13^C_6_, 99%, +6, CIL, CLM-2265-H-0.1). After a three-day or six-day feeding period with isotope-labelled amino acids, 12 female fly heads were homogenized in 50 μl RIPA buffer (FUJIFILM Wako, 188-02453) supplemented with a protease inhibitor (FUJIFILM Wako, 165-26021). Four samples were prepared for isotope-labelled proteome analysis. Protein extracts in RIPA buffer were sonicated using a BioRuptor with ten cycles on high power, with 60 s on followed by 30 s off. Then the samples were centrifuged at 18,000*g* for 20 min at 4 °C and 10 μl of the sample supernatants was taken. The detergent concentration in the samples was decreased using methanol–chloroform extraction^74^. Then, 105 µl of 50 mM ammonium bicarbonate buffer was added to the protein pellets. Protein amounts were quantified using 5 µl of each sample with a BCA assay kit (Thermo Fisher Scientific). The average protein amount in 100 µl was 12 µg. The cysteine disulfide bonds were reduced with 10 mM Tris (2-carboxyethyl) phosphine hydrochloride (TCEP-HCl) at 37 °C for 30 min, and then the cysteines were alkylated with 50 mM 2-chloroacetamide (CAA) at room temperature for 30 min. For protein digestion, 300 ng Lys-C was added, and the samples were incubated at 37 °C for 1 h. Then, 300 ng trypsin was added, and the samples were incubated at 37 °C for 16 h. The next day, samples were acidified with around 0.5% (final concentration) trifluoroacetic acid (TFA). Tryptic peptides were desalted with polystyrene-divinylbenzene, reversed-phase sulfonate (SDB-RPS) StageTips^75^.

A nanoLC–MS/MS system comprising a Vanquish Neo UHPLC system (Thermo Fisher Scientific) and an Orbitrap Astral Zoom mass spectrometer was operated at a full-MS resolution of 240,000 with a full scan range of 380–980 *m*/*z* when stated. The full-MS automatic gain control (AGC) was set to 500%. We used 300 windows of 2 Th, scanning from 380 to 980 *m*/*z*, with a maximum injection time (maxIT) of 3 ms. The isolated ions were fragmented using higher-energy collisional dissociation (HCD) with a 25% normalized collision energy (NCE). Peptides were loaded onto an Aurora Ultimate (25 cm length, 75 μm inner diameter, 1.7 μm particle size; IonOpticks) and separated by a linear gradient (6 − 44% B in 20 min, 44 − 99% B in 1.2 min and 99% B for 2.5 min) at a flow rate of 300 nl min^−1^.

Raw data files were analysed with DIA-NN software^76^, v.2.3.0. A predicted spectral library was first made using DIA-NN, which was annotated with two sequence databases: (1) a database of 22,034 Swiss-Prot reviewed and TrEMBL unreviewed canonical and isoform *D. melanogaster* proteins (proteome ID: UP000000803) downloaded from UniProt on 30 September 2024, and (2) a database of common mass-spectrometry contaminant proteins, which was included with the DIA-NN software package. We used the following additional options for plexDIA: --fixed-mod SILAC,0.0,KR,label --lib-fixed-mod SILAC --channels SILAC,L,KR,0:0; SILAC,M,KR,4.025107:6.020129; SILAC,H,KR,8.014199:10.008269 --original-mods --channel-run-norm. For the data analysis, output was filtered at 0.01 false discovery rate (FDR), N-terminal methionine excision was enabled; the maximum number of missed cleavages was set to 1; cysteine carbamidomethylation was enabled as a fixed modification; protein inference for generating a subset of all protein IDs matched to the precursor was set to relaxed mode; and the empirical library generation mode was set to identifications, retention time and ion mobility profiling. The report.parquet files were used for subsequent data analysis. The intensity-based absolute quantification (iBAQ)^38^ algorithm computes the sum of all the peptide intensities divided by the number of theoretically observable peptides, which provides a rough estimation of protein abundance. We applied the following additional filters to compute SILAC ratios and iBAQ intensities: channel.Q.Value < 0.01, Global.PG.Q.Value < 0.01, Quantity.Quality > 0. Precursor SILAC ratios were calculated by dividing the light, medium or heavy ‘Precursor.Normalised’ intensities by the corresponding partner. Protein-level SILAC ratios were then calculated by taking the median of all SILAC ratios for all precursors of a given protein. iBAQ intensities for light, medium and heavy channels were calculated by (1) summing up all precursor intensities (‘Precursor.Normalised’) for each protein and (2) dividing summed intensities by the number of theoretically observable peptides^38^. The top 25% abundant heavy-labelled proteins were selected and used for GO analysis.

### Global proteome analysis

Standard proteome analysis was performed on a total of 12 heads of female flies or 5 whole bodies of wandering larvae. These flies were homogenized in 50 μl (heads) or 100 μl (whole larvae) of RIPA buffer (FUJIFILM Wako, 188-02453) supplemented with a protease inhibitor (FUJIFILM Wako, 165-26021). Six samples of protein extracts in RIPA buffer were sonicated using a Bioruptor BR-11 on high power for ten cycles with 1 min on followed by 30 s off. The samples were then centrifuged at 10,000*g* for 10 min at 4 °C. The phase transfer surfactant (PTS) method of sample preparation was followed^77,78^; 10.6 µl supernatant was taken from each sample and mixed with 144.4 µl PTS buffer (12 mM SDC, 12 mM SLS and 100 mM Tris-HCl pH 8.5). A 5-µl aliquot of each sample was taken for quantification using a BCA assay. The average protein amount was about 35 µg in 150 µl protein extract. TCEP was added at 10 mM and the samples were incubated at 37 °C for 30 min, then CAA was added at 50 mM and the samples were incubated at 25 °C for 30 min. Then, 600 µl 50 mM ammonium bicarbonate was added, followed by 700 ng Lys-C and 700 ng trypsin for protein digestion at 37 °C for 16 h. The following day, 780.3 µl ethyl acetate was added to each sample followed by the addition of TFA to a final concentration of 0.75%. The samples were vortexed for 2 min then centrifuged at 12,000*g*, for 5 min at room temperature. The supernatant was discarded. The samples were desalted with SDB-RPS StageTips.

The peptides were eluted from the SDB-RPS StageTips, vacuum-dried and then dissolved in around 17.5 µl 0.1% formic acid, 3% acetonitrile and 97% water. A Q Exactive Plus mass spectrometer, together with an EASY-nLC 1200 and NanoSpray Flex Ion Source (Thermo Fisher Scientific), were used for sample measurement. Analytical columns had an inner diameter of 75 µm, and contained 1.9-µm C18 particles, with an 18-cm filling length. The gradient conditions were as follows, with the percentage of acetonitrile as indicated: 0–1 min, 0–4.0%; 1–110 min, 4.0–32.0%; 110–112 min, 32.0–76.0%; 112–120 min, 76.0%; 120–121 min, 76.0–4.0%; 121–136 min, 4.0%. The flow rate was 150 nl min^−1^. The ion-transfer capillary temperature was 250 °C and the spray voltage was 2.0 kV. MS acquisition conditions were as previously described. In brief, a full-MS scan was collected from 385 to 1,015 *m*/*z* at 35,000 resolution, with an AGC target of 1e6 and a maxIT of 55 ms. This was followed by 76 data-independent acquisition (DIA) scans at 17,500 resolution, with an AGC target of 1e6, a maxIT of 55 ms, a default charge state of 3, a loop count of 38, isolation windows of 16.0 *m*/*z*, a fixed first mass of 150.0 *m*/*z* and 27% HCD collision energy. Two staggered series of 38 windows were used: 408.4355 to 1,000.7047 then 400.4319 to 992.70110. All spectrum data were centroid type. For the *Lsp2-*knockdown experiments, a nanoLC–MS/MS system comprising a Vanquish Neo UHPLC and an Orbitrap Astral mass spectrometer was operated at a full-MS resolution of 240,000 with a full scan range of 380–980 *m*/*z* when stated. The full-MS AGC was set to 500%. We used 300 windows of 2 Th, scanning from 380 to 980 *m*/*z*, with a maxIT of 3 ms. The isolated ions were fragmented using HCD with 25% NCE. Peptides were loaded onto a Aurora Ultimate (25 cm length, 75 μm inner diameter, 1.7 μm particle size; IonOpticks) and separated by a linear gradient (5–40% B in 16 min, 40–99% B in 1 min and 99% B for 3 min) at a flow rate of 350 nl min^−1^). Raw data files acquired with DIA were analysed with DIA-NN software^76^, v.1.9.2 or v.2.3.0. A predicted spectral library was first made using DIA-NN, which was annotated with two sequence databases: (1) a database of 22,034 Swiss-Prot reviewed and TrEMBL unreviewed canonical and isoform *D. melanogaster* proteins (proteome ID: UP000000803) downloaded from UniProt on 30 September 2024, and (2) a database of common mass-spectrometry contaminant proteins, which was included with the DIA-NN software package. For the data analysis, output was filtered at 0.01 FDR; N-terminal methionine excision was enabled; the maximum number of missed cleavages was set to 1; cysteine carbamidomethylation was enabled as a fixed modification; protein inference for generating a subset of all protein IDs matched to the precursor was set to relaxed mode; and the empirical library generation mode was set to identifications, retention time and ion mobility profiling. The output from the data analysis included a protein group matrix file of the relative protein abundances. Note that MS-based quantitative proteome analysis is typically based on comparisons of normalized signal intensities across samples. Therefore, although this approach is well suited to detecting relative changes in the abundance of individual proteins within the proteome, it does not necessarily capture global shifts in total protein abundance. For example, if ePR or *Lsp2*-RNAi causes a proteome-wide decrease in protein content per cell, or reduces the cell number or cell size, such global reductions might be cancelled out by normalization, leading to an underestimation of the actual fold changes. Thus, although absolute changes in protein abundance might not be accurately quantified, this analysis can still reliably detect relative changes in specific protein groups within the overall proteome, such as whether RPs are selectively reduced compared with other protein classes.

### Statistical analysis

Statistical analysis was performed using GraphPad Prism 10 or 11, R or OASIS 2 (ref. ^63^). The sample size was determined empirically. To eliminate biological bias, the flies were randomly distributed onto each diet. All data points were biological, not technical, replicates. No data were excluded. Most experiments were not done in a blinded manner because the experiment planner and the experimenter were the same person. For the experiments on lifespan, a different person performed the experiments without prior bias whenever possible. An unpaired and two-sided Student’s *t-*test was used to compare samples. One-way ANOVA with Šídák’s multiple comparison test was used to compare groups. One-way ANOVA with Dunnett’s multiple comparison test was used to compare against a control sample. Two-way ANOVA with Šídák’s multiple comparison test was used to compare groups at different time points. Two-way ANOVA with Dunnett’s multiple comparison test was used to compare against a control sample at different time points. Log-rank test was used for survival curve comparisons. All experimental results were repeated at least twice to confirm reproducibility. Bar graphs are drawn as the mean and s.e.m. or s.d. For the proteome analyses, we used only proteins that were quantified in all the biological replicates of at least one condition for further analyses. Quality checking of the proteomic data was done by an R package, DEP^79^. MS signal intensities were normalized across samples. Missing values were imputed using random draws from a Gaussian distribution centred around a minimal intensity value. Principal component analysis (PCA) was performed using the top 500 most variable proteins. *P* values were computed using limma-based moderated *t*-tests with empirical Bayes moderation. A two-sided Wilcoxon rank-sum test was used to compare groups (all versus cytoRPs or mitoRPs) (no adjustment). Outliers are not shown in the box plots. Adjusted *P *values were computed using Welch’s *t-*test and the Benjamini–Hochberg method for line plots (pSILAC combined with ePR or *Lsp2* RNAi).

### Reporting summary

Further information on research design is available in the Nature Portfolio Reporting Summary linked to this article.

## Online content

Any methods, additional references, Nature Portfolio reporting summaries, source data, extended data, supplementary information, acknowledgements, peer review information; details of author contributions and competing interests; and statements of data and code availability are available at https://doi.org/10.1038/s41586-026-11031-3.

## Supplementary information

Supplementary Fig. 1 Uncropped immunoblots from Extended Data Figs. 2, 5 and 6. **a**, Immunoblots of phosphorylated-Akt, Akt, and histone H3 in Extended Data Fig. 2a. **b**, Immunoblots of phosphorylated-Akt, Akt and histone H3 in Extended Data Fig. 2j. **c**, Immunoblots of Lsp2 and α + β-tubulin in Extended Data Fig. 5b. **d**, Immunoblots of GFP and α + β tubulin in Extended Data Fig. 5g. **e**, Immunoblots of phosphorylated-Akt and histone H3 in Extended Data Fig. 6d. **f**, Immunoblots of phosphorylated-Akt and histone H3 in Extended Data Fig. 6f.

Reporting Summary

Supplementary Table 1 Cox proportional hazards analysis of lifespan data.

Supplementary Table 2 Statistical analysis and peptide intensities of pSILAC data.

Supplementary Table 3 Statistical analysis of the adult proteome data.

Supplementary Table 4 RNA-seq analysis: differentially expressed genes (DEGs) and Gene Ontology (GO) enrichment analysis.

Supplementary Table 5 Proteomic profiles of adult ePR-flies.

Supplementary Table 6 List of primers and oligonucleotides used in this study.

Peer Review File

## Source data

Source Data Fig. 1

Source Data Fig. 2

Source Data Fig. 3

Source Data Fig. 4

Source Data Fig. 5

Source Data Extended Data Fig. 1

Source Data Extended Data Fig. 2

Source Data Extended Data Fig. 3

Source Data Extended Data Fig. 4

Source Data Extended Data Fig. 5

Source Data Extended Data Fig. 6

Source Data Extended Data Fig. 7

Source Data Extended Data Fig. 8

Source Data Extended Data Fig. 9

## Data Availability

All data supporting the findings of this study are available within the paper, its Supplementary Information and the associated data repository. The NGS data are available under accession numbers DRR622306–DRR622313 and DRR626770–DRR626793. The *D. melanogaster* reference genome and annotation (BDGP 6.46; assembly accession GCA_000001215.4) were obtained from Ensembl. The proteomics data have been deposited in the ProteomeXchange Consortium via the jPOST^80^ partner repository with the dataset identifier PXD062930 (JPST003763). Peptide sequence was annotated with two sequence databases: (1) a database of 22,034 Swiss-Prot reviewed and TrEMBL unreviewed canonical and isoform *D. melanogaster* proteins (proteome ID: UP000000803) downloaded from UniProt on 30 September 2024, and (2) a database of common mass-spectrometry contaminant proteins, which was included with the DIA-NN software package. Source data are provided with this paper.

## Code availability

All analysis code is available from the authors upon request.
