# Supplementary material for: Lsp2 links early-life diet to adult translation and lifespan in Drosophila
Source: Nature. 2026 Sep 23;658(8135):474–84. doi: 10.1038/s41586-026-11031-3 (PMC13645648; doi:10.1038/s41586-026-11031-3)
Supplement: Supplementary file 1 [file 41586_2026_11031_MOESM1_ESM.pdf]

nature portfolio

https://doi.org/10.1038/s41586-026-11031-3

Supplementary information

Lsp2 links early-life diet to adult translation and lifespan in *Drosophila*

In the format provided by the
authors and unedited

SI Figure

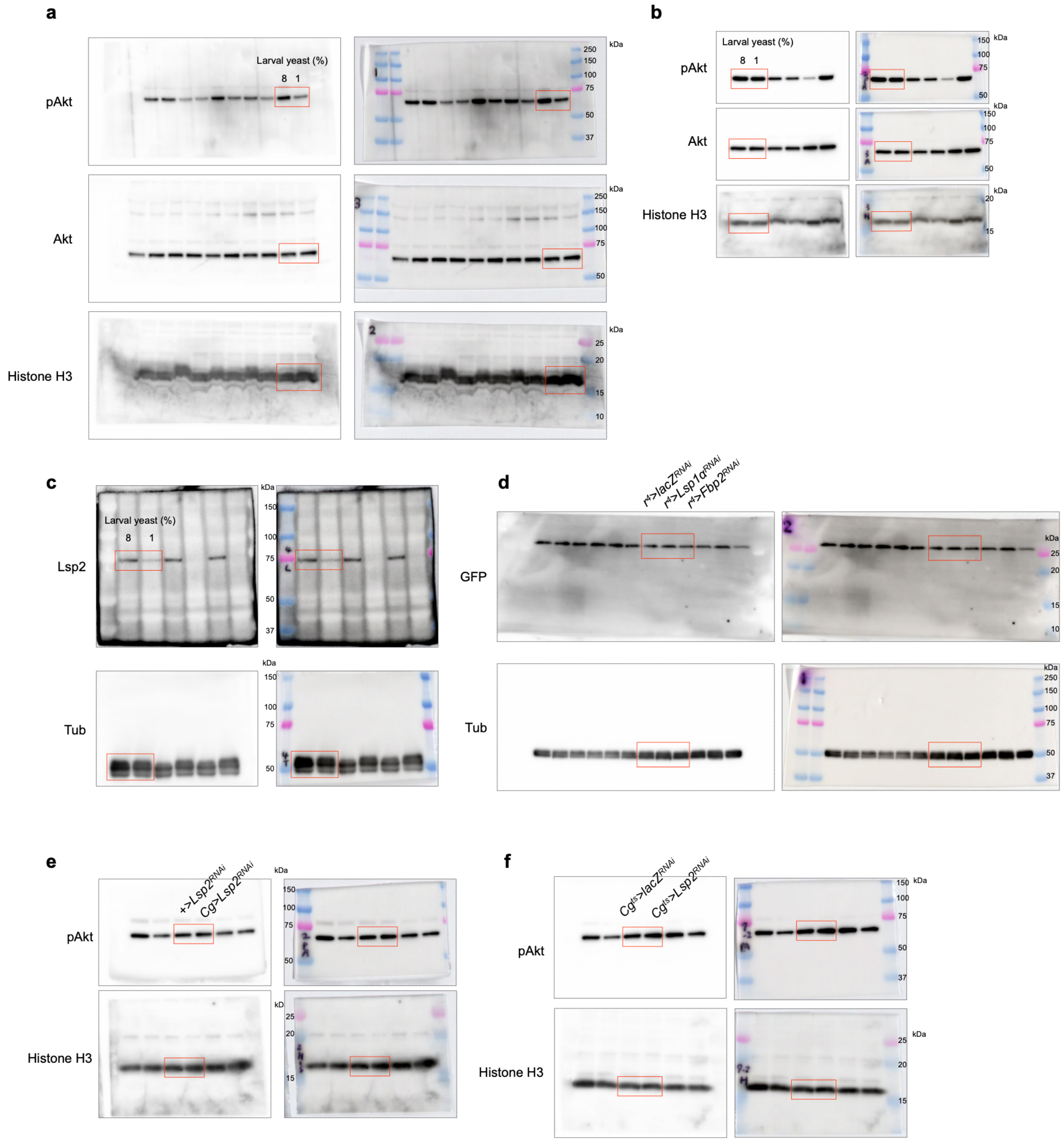

SI Figure 1 | Uncropped immunoblots from Extended Data Figure 2, 5, and 6.

a, Immunoblots of phosphor-Akt, Akt, and histone H3 in Extended Data Fig. 2a. **b**, Immunoblots of phosphorylated-Akt, Akt, and histone H3 in Extended Data Fig. 2j. **c**, Immunoblots of Lsp2 and α + β tubulin in Extended Data Fig. 5b. **d**, Immunoblots of GFP and α + β tubulin in Extended Data Fig. 5g. **e**, Immunoblots of phosphorylated-Akt and histone H3 in Extended Data Fig. 6d. **f**, Immunoblots of phosphorylated -Akt and histone H3 in Extended Data Fig. 6f.
