# Supplementary material for: Lsp2 links early-life diet to adult translation and lifespan in Drosophila
Source: Nature. 2026 Sep 23;658(8135):474–84. doi: 10.1038/s41586-026-11031-3 (PMC13645648; doi:10.1038/s41586-026-11031-3)
Supplement: Supplementary file 2 [file 41586_2026_11031_MOESM2_ESM.pdf]

nature portfolio

Corresponding author(s): Fumiaki Obata, Koshi Imami

Last updated by author(s): Jul 20, 2026

Reporting Summary

Nature Portfolio wishes to improve the reproducibility of the work that we publish. This form provides structure for consistency and transparency in reporting. For further information on Nature Portfolio policies, see our Editorial Policies and the Editorial Policy Checklist.

Statistics

For all statistical analyses, confirm that the following items are present in the figure legend, table legend, main text, or Methods section.

n/a	Confirmed	
☐	☒	The exact sample size (*n*) for each experimental group/condition, given as a discrete number and unit of measurement
☐	☒	A statement on whether measurements were taken from distinct samples or whether the same sample was measured repeatedly
☐	☒	The statistical test(s) used AND whether they are one- or two-sided<br>*Only common tests should be described solely by name; describe more complex techniques in the Methods section.*
☒	☐	A description of all covariates tested
☐	☒	A description of any assumptions or corrections, such as tests of normality and adjustment for multiple comparisons
☐	☒	A full description of the statistical parameters including central tendency (e.g. means) or other basic estimates (e.g. regression coefficient) AND variation (e.g. standard deviation) or associated estimates of uncertainty (e.g. confidence intervals)
☐	☒	For null hypothesis testing, the test statistic (e.g. *F*, *t*, *r*) with confidence intervals, effect sizes, degrees of freedom and *P* value noted<br>*Give P values as exact values whenever suitable.*
☒	☐	For Bayesian analysis, information on the choice of priors and Markov chain Monte Carlo settings
☒	☐	For hierarchical and complex designs, identification of the appropriate level for tests and full reporting of outcomes
☐	☒	Estimates of effect sizes (e.g. Cohen's *d*, Pearson's *r*), indicating how they were calculated

Our web collection on statistics for biologists contains articles on many of the points above.

Software and code

Policy information about availability of computer code

Data collection	No unpublished code to collect the data. Detailed data collection methods were described in Methods.
Data analysis	No unpublished code to analyse the data. Detailed data analysis methods were described in Methods. FastQC (v0.12.1), MultiQC (v1.24), Trim Galore (v0.6.10), Hisat2 (v2.2.1), StringTie (v2.2.1), edgeR (v4.0.6) were used for RNAseq analysis. Fiji was used for imaging analysis. OASIS 2 was used to perform log-rank test for lifespan analysis. R (v4.6.0) was used for Cox proportional hazards analysis. DIA-NN software (v1.92 or 2.3.0) and R (v.4.3.1) package DEP was used for proteome analysis. GraphPad Prism 10 or 11 was used for data presentation and statistical analysis.

For manuscripts utilizing custom algorithms or software that are central to the research but not yet described in published literature, software must be made available to editors and reviewers. We strongly encourage code deposition in a community repository (e.g. GitHub). See the Nature Portfolio guidelines for submitting code & software for further information.

Data

Policy information about availability of data

All manuscripts must include a data availability statement. This statement should provide the following information, where applicable:

- Accession codes, unique identifiers, or web links for publicly available datasets
- A description of any restrictions on data availability
- For clinical datasets or third party data, please ensure that the statement adheres to our policy

All data supporting the findings of this study are available within the paper, its Supplementary Information, and the associated data repository. Source data are provided with this paper. The NGS data are available under accession numbers DRR622306-DRR622313, DRR626770-DRR626793. The Drosophila melanogaster reference genome and annotation (BDGP 6.46; assembly accession GCA_000001215.4) were obtained from Ensembl. The proteomics data are deposited to the ProteomeXchange Consortium via the jPOST partner repository with the dataset identifier PXD062930 (JPST003763). Peptide sequence was annotated with two sequence databases: a database of 22,034 Drosophila melanogaster (UP000000803) Swiss-Prot reviewed and TrEMBL unreviewed canonical and isoform proteins downloaded from UniProt on September 30, 2024, and a database of common mass spectrometry contaminant proteins, which was included with the DIA-NN software package.

Research involving human participants, their data, or biological material

Policy information about studies with human participants or human data. See also policy information about sex, gender (identity/presentation), and sexual orientation and race, ethnicity and racism.

Reporting on sex and gender	N/A
Reporting on race, ethnicity, or other socially relevant groupings	N/A
Population characteristics	N/A
Recruitment	N/A
Ethics oversight	N/A

Note that full information on the approval of the study protocol must also be provided in the manuscript.

Field-specific reporting

Please select the one below that is the best fit for your research. If you are not sure, read the appropriate sections before making your selection.

☒ Life sciences ☐ Behavioural & social sciences ☐ Ecological, evolutionary & environmental sciences

For a reference copy of the document with all sections, see nature.com/documents/nr-reporting-summary-flat.pdf

Life sciences study design

All studies must disclose on these points even when the disclosure is negative.

Sample size	Sample size was determined empirically. A similar sample size was used in previous papers (DOI: 10.1038/ncomms9332, DOI: 10.7554/eLife.62335).
Data exclusions	No data were excluded except that proteins that matched as contaminants (e.g., trypsin) were excluded.
Replication	NGS analyses and proteome analyses were not repeated due to limitation of budget, but the leading conclusion was confirmed by qPCR or western blot. Each NGS analysis sample included 4 biological replicates. All proteomics samples included at least triplicates.<br>All other data were confirmed, at least twice, by the same or closely-related experiments.
Randomization	Animals were allocated ramdomly to different dietary conditions.
Blinding	No blind tests were implemented because the same investigator set up the experiments, collected samples, and analysed the data. However, the samples and data were collected under identical conditions.

Reporting for specific materials, systems and methods

We require information from authors about some types of materials, experimental systems and methods used in many studies. Here, indicate whether each material, system or method listed is relevant to your study. If you are not sure if a list item applies to your research, read the appropriate section before selecting a response.

Materials & experimental systems

n/a	Involved in the study	
☐	☒	Antibodies
☒	☐	Eukaryotic cell lines
☒	☐	Palaeontology and archaeology
☐	☒	Animals and other organisms
☒	☐	Clinical data
☒	☐	Dual use research of concern
☒	☐	Plants

Methods

n/a	Involved in the study	
☒	☐	ChIP-seq
☒	☐	Flow cytometry
☒	☐	MRI-based neuroimaging

Antibodies

Antibodies used

Primary antibodies used in the study were anti-α + β tubulin [DM1A +DM1B] (Abcam, ab44928, 1:1000 dilution), anti-histone H3 (1B1B2) (CST, 14269S, 1:1000 dilution), anti-phospho-Akt (Ser473) (D9E) (CST, 4060T, 1:1000 dilution), anti-total Akt (CST, 9272S, 1:1000 dilution), anti-GFP (GF090R) (nacalai, 04404-26, 1:1000 dilution), anti-Lsp2 (gifted from Dr. Pierre Leopold), and anti-puromycin (12D10) (Millipore, MABE343, 1:1000 dilution). HRP-conjugated secondary antibodies were anti-mouse IgG, HRP-linked antibody (CST, 7076S, 1:1000 dilution), anti-rabbit IgG, HRP-linked antibody (CST, 7074S, 1:1000 dilution), anti-rat IgG, HRP-linked antibody (Jackson ImmunoResearch, 81211, 1:1000 dilution), and anti-mouse IgG2a, HRP-linked antibody (Jackson ImmunoResearch, 115-035-206, 1:15000 dilution).

Validation

anti-α + β tubulin (Abcam, ab44928): Mouse Multiclonal Alpha-tubulin 1 acetyl K40 antibody. Suitable for IHC-P, ICC, Flow Cyt, WB and reacts with Human, Drosophila melanogaster, Mouse, Rat samples. Cited in 54 publications.

anti-histone H3 (CST, 14269S): Histone H3 (1B1B2) Mouse mAb recognizes endogenous levels of total histone H3 protein. Suitable for WB, IHC, IF, Flow Cyt, ChIP and reacts with Human, Mouse, Rat, Monkey samples. Cited in 213 publications.

anti-phospho-Akt (CST, 4060T): Phospho-Akt (Ser473) (D9E) XP® Rabbit mAb detects endogenous levels of Akt1 only when phosphorylated at Ser473. This antibody also recognizes Akt2 and Akt3 when phosphorylated at the corresponding residues. Suitable for WB, IP, IHC, IF, Flow Cyt and reacts with Human, Mouse, Rat, Hamster, Monkey, D. melanogaster, Zebrafish, Bovine samples. Cited in 11988 publications.

anti-total Akt (CST, 9272S): Akt antibody detects endogenous levels of total Akt1, Akt2 and Akt3 proteins. The antibody does not cross-react with related kinases. Suitable for WB, IP, IF, Flow Cyt and reacts with Human, Mouse, Rat, Hamster, Monkey, Chicken, D. melanogaster, Bovine, Dog, Pig, Guinea Pig samples. Cited in 11085 publications.

anti-GFP (nacalai, 04404-26): GFP antibody recognizes the levels of GFP protein. Suitable for WB, IHC, ELISA. Cited in 19 publications. The antibody is previously used in Drosophila (DOI: 10.21769/BioProtoc.5305).

anti-Lsp2: produced and validated in Valzania et al., Dev. Cell, 2024 (doi: 10.1016/j.devcel.2024.05.018)

anti-puromycin (Millipore, MABE343, 1:1000 dilution): Suitable for WB, ICC, IF, IHC, FACS, IP. Cited in at least 13 publications for WB application. The antibody is previously used in Drosophila (DOI: 10.14348/molcells.2019.0273).

Animals and other research organisms

Policy information about studies involving animals; ARRIVE guidelines recommended for reporting animal research, and Sex and Gender in Research

Laboratory animals

The following fly stocks were used in this study. The sex and the age of the flies used are specified in the method or figure legends.
Canton-S
wCanton-S (w[iso31] eight times backcrossed with Canton-S)
4E-BP[intron]-dsRed (doi: 10.1083/jcb.201511073)
Cg-Gal4 (Bloomington Drosophila Stock Center (BDSC), 7011, eight times backcrossed with wCanton-S)
tub-Gal80ts (BDSC 7017)
r4-Gal4 (Bloomington 33832, eight times backcrossed with wCanton-S)
fit-Gal4 (doi: 10.1038/ncomms14161)
WBFB-GS (doi: 10.18632/aging.100018)
UAS-2×EGFP (BDSC 6874)
UAS-lacZ-RNAi (from Dr. Carthew R., eight times backcrossed with wCanton-S)
UAS-Lsp2-RNAi (National Institute of Genetics (NIG), 6806R-2, eight times backcrossed with wCanton-S)
UAS-Lsp1α-RNAi (Vienna Drosophila Resource Center (VDRC), 14898)
UAS-Fbp2-RNAi (VDRC, 33173)
Lsp2-muGFP (generated in this paper)
pGnmt-GeneSwitch (generated in this paper)

Wild animals

The study did not use wild animals.

Reporting on sex

Both females and males are used in this study depending on the experiment. All the sex information is included in the manuscript.

Field-collected samples	The study did not include samples from field-collected samples.
Ethics oversight	No ethical approval was required.

Note that full information on the approval of the study protocol must also be provided in the manuscript.

Plants

Seed stocks	N/A
Novel plant genotypes	N/A
Authentication	N/A
