# Supplementary material for: Lsp2 links early-life diet to adult translation and lifespan in Drosophila
Source: Nature. 2026 Sep 23;658(8135):474–84. doi: 10.1038/s41586-026-11031-3 (PMC13645648; doi:10.1038/s41586-026-11031-3)
Supplement: Supplementary file 3 [file 41586_2026_11031_MOESM9_ESM.pdf]

nature portfolio

Peer Review File

Lsp2 links early-life diet to adult translation and lifespan in Drosophila

Corresponding Author: Dr Fumiaki Obata

Any redactions in this file are there to maintain patient confidentiality, the confidentiality of unpublished data, or to remove third-party material.

This file contains all reviewer reports in order by version, followed by all author rebuttals in order by version.

Version 1:

Reviewer comments:

Referee #1

(Remarks to the Author)
Overall

I like the theme and insights of this creative paper. Food intake (protein) affects aging. Long research has sought to understand the underlying sensing, signaling and metabolic mechanisms. Restriction in juvenile stages is effective. Kosakamoto provides an expansive and exciting study for how larval storage proteins (Lsp2) regulates adult survival. After parsing the many data, I can summarize the novel, salient conclusion: Larval diet restriction (ePR) extends adult lifespan and alters metabolic/protein features of the young adult including translation. Among various proteins, Lsp2 is reduced in the adult by ePR. RNAi of Lsp2 in larvae reduces total translation in the young adult and extends female survival.

There are many data in the paper that are interpreted with interesting implications, for instance about translation control, protein turnover, the carry-over of molecules from larvae to adults, and costs of reproduction. Yet, I feel the authors over-emphasize incidental observations and make many conclusions based on associational inferences.

Still, the paper has remarkable insights and advances a central objective in aging research. It will have more impact with streamlining and if it establishes focused, robust inferences on the function of Lsp2 that arise following ePR.

An apology: My review is perhaps overly detailed and too personal (and too long). Being so intimate with the addressed questions, I write to engage the authors constructively and to share my experience. But I pull no punches.

Introduction

Needs to be more specific with relevant background. It feels vague. The first sentences are strawmen (line 46-48) because the field does consider juvenile diet, as demonstrated in the rest of the paragraph. I suggest the paper opens with the phenomenon as a given and state a plan to understand its underlying mechanism. Discuss specifics of current knowledge and proposed mechanisms for early-life impact on adult aging: Size, metabolism, epigenetics, etc.

Provide details from the work of Stefana 2017 (which is swept under the rug), and our work (Tu/Tatar 2003, also under the rug). Tu used an almost identical design but without finding a positive effect on adult lifespan. Although awkward, explicitly recognize these ambiguities. It is better now than when others try to reproduce your observations (with or without success).

No need to justify the fly model (second intro paragraph). Instead, set up your story by telling the reader why and what we need to understand, and what you will reveal.

Results
Larval diets were reduced from 8% yeast to 2% or 1%. The Obata stocks are maintained on 6% yeast extract. 8% may be a stress condition whereby these controls are not ‘normal’.

Larval protein restriction reduces ovariole number. Include reference #18 on line 97. Please add data for the 2% ePR. Quantify/statistically establish the subjective claim 'milder' (line 99).

Good data on additivity with adult DR. Then the paragraph turns to adults on 0% protein where the benefit of ePR is lost. This observation does not imply, however, 'the outcome of ePR can be variable [via adult dietary conditions] among experimental conditions (refs 6, 18)' (line 116). This explanation obscures the real ambiguities. I can speak for ref 18 (Tu/Tatar 2003): Tu held the 3rd instar with no yeast (rather than 1% or 2%). Tu/Tatar maintain flies with baker's yeast, while Obata uses brewer's yeast. Differences are fine, but readers need to understand specifics and likely causes.

Line 120. Tu (2003) established that ePR modulates insulin signaling in the young adult. That paper also sets a stage for some (then unknown) impact of ePR on JH (we would now say, via insulin), which further connects Kosakamoto to the paper of Kim/Pickering (early adult translation).

The paragraph at line 133 does not convince that ePR 'affects nutrient sensing pathways transiently in early adulthood'. Readers will not accept that ATF4 is purely a feature of amino acid sensing. The role of phosphor-eIF2 is not obvious, and it is decoupled from ATF4. I think the paper works better without this paragraph.

The isotope study. Please (again) excuse my self-reference, but we did a similar isotope larvae/adult tracing study and found similar conclusions (Min … O'Brien JIP 2006; O'Brien … Tatar 2008 Current Biology. I attach a PDF of 2008 because the paper is hard to find). We made 15N & 13C labeled yeast, and 14N &12C. Larvae and adults were fed alternative isotope forms. We collected somatic tissue and eggs across the aging cohort. From a low concentration of label, we quantified turnover rates and equilibria, all in the context of adult DR. We found many features reported in the current submission. These papers complement, so please educate and guide the reader.

The global enrichment analysis (paragraph starting at line 184) is subjective and tangential. Can these inferences be supported with statistics? Are they essential to the paper's conclusion?

Proteomics. ePR alters the quantity and quality of amino acids in young adults, but ePR was not studied with the labeled samples. It is a conjecture that the altered profiles arise from carry-over. We can't distinguish effects from ePR relative to adult feeding.

The protein profiles in young adults suggest there is reduced translation capacity following ePR. Kim/Pickering (Nature Comm 2023) recently reported young adults have a 'spike' in translation. Suppressing the spike increased life expectancy. Kosakamoto now sees high translation in young adults that is blunted by ePR. They propose this contributes to slow aging. Kosakamoto activates mTORC1 in ePR treated flies to test this idea, and this reduces their longevity. However, Kosakamoto increases mTORC1 across all adult ages, not just in the phase where ePR is thought to inhibit translation. Most would say that elevating mTORC1 across adulthood is bad for survival. A better design requires a time-limited rescue (as in Kim/Pickering), and analysis of age-specific mortality following the manipulation period.

The research sequence at line 241-243 seems strange. Kosakamoto already conducted the proteomics to identify the 'molecules', so why bother with transcriptome analysis?

Lsp2 mRNA is selected as the focal DEG, but its expression in the adult head is not 'unexpected' (line 265). It was documented in 1990 (Benes et al.) and verified in expression studies (see FlyBase). Kosakamoto mention Benes in the discussion. To justify the Lsp2 focus at this point, directly talk about the storage protein data of Kim/Pickering (Nature Comm 2023). They identified Lsp2 as a dynamic factor in the early soluble fraction. Use this to set up the appropriate question: where does the adult LSP2 come from? The LSP2 may arise from adult translation because adults have high Lsp2 mRNA (PCR) and protein, whereas adults from ePR have lower Lsp2 mRNA and protein. (And … why Lsp2 relative to all the other storage proteins?)

I am confused by the wording in lines 279-280 and 281-283. If the idea is that ePR represses adult Lsp2 mRNA, why discuss wandering larvae and pupae? I am not convinced that knockdown of Lsp2 (or Fbp) across all larval stages suggests a 'feed forward mechanism to regulate storage protein expression'. Might knockdown block larval fat body development, which carries across to the adult. Not 'nutritional memory' (per ePR) but stunted tissue development?

The hypothesis of line 297-308. The premise is weak. Turn-over of LSP occurs in the adult, as it does for many larval-derived proteins (except perhaps those in the cuticle). See O'Brien (2008) who quantified nitrogen-molecule turnover as a function of adult DR. Kosakamoto could benefit from pSILAC of adults derived from the three larval ePR conditions. This would quantify how much LSP derives from larvae and from adult feeding and estimate the turnover in the adult. Those data would test if the 'observed reduction' (via ePR) is caused by less carryover or reduced production in the adult (need not be via 'suppressed transcription'). One caveat concerns the issue of recycled amino acids.

The development-limited Lsp2 RNAi data are central to the paper. Kosakamoto buries them behind the continuous knockdown (larva through adulthood) data. The continuous data confound what they want to understand about ePR with the impacts from Lsp2 RNAi in the adult.

The project needs fuller data from the larval-only knockdown. How does this effect the omic measures, interact with ePR (viz longevity), affect males? How effective is the larval RNAi to reduce the adult Lsp2 protein, and over what time course?

Address a genetic background issue: gal80 controls time of expression but compares different genetic backgrounds. Even with some backcrossing, there is much room for second site effects (especially in survival data). I suggest Kosakamoto measures phenotypes of both genotypes (lacz and RNAi) from animals continuously at the restrictive temperature. They should look the same. I recommend they replicate all survival trials to demonstrate the result is reproducible (one 'significant' trial is weak sauce). Include males and give data in the main figure.

The tyrosine result does not demonstrate Lsp2 as the cause, or visa versa. I suggest controls to show that lifespan is not increased when you reduce amino acids besides Tyr, Phe or His (Lsp2 specialties). Line 352-353 connects the exome observations to aging through some previous work (refs 22, 27). What is meant by 'highly sensitive to Tyr intake'? Explicitly discuss results from Parkhitko (eLIFE 2020), which established the role of Tyr in the control of aging, although paradoxically in the opposite direction.

Discussion

In my opinion, the work does not yet establish its conclusion of line 364-366 because the referenced results were based on life-long Lsp2 RNAi, not larval-limited.

I am not sure Kosakamoto's data support the conclusion asserted on lines 370-372. Kosakamoto associates a storage protein with features of translation control. While knockdown of Lsp2 in the larvae reduces total protein translation in the young adult, the work does not prove the change in translation is required to slow aging. The design with TORC1 is not convincing. What I do see is that reducing Lsp2 in the larval stage (by RNAi) can extend lifespan in females.

Indirect inferences are used for big conclusions of the discussion. I enjoyed these ideas, especially in the last several paragraphs. They are broad and exciting, but I am not convinced they are supported by the offered data.

Referee #2

(Remarks to the Author)
General comments

In this study, Kosakamoto et al. investigate the mechanisms mediating lifespan extension by protein restriction during development. Using Drosophila melanogaster, a relevant model for the study of longevity, they establish that 24-hours protein restriction in early third instar larvae (ePR) is sufficient to extend the lifespan of wild type adult animals. The effect of this treatment on lifespan with respect to diets fed to adult animals is investigated, along with the fitness cost on growth and reproduction, as well as the effect on nutrient signaling pathways in young adults. They further develop a SILAC assay using differentially labelled amino acids fed to larvae and adults in order to monitor protein turnover across developmental stages. They demonstrate that larval proteins contribute significantly to the adult proteome, in particular ribosomal proteins. Additional analyzes of the amino acid content and of the proteome indicate that ePR impacts amino acids balance and decreases translation in young adults, a process that has previously been linked to lifespan extension, thereby providing a mechanistic link between ePR and lifespan. The authors next search for a functional mediator of this process. Using transcriptomics analyzes, they identify the storage protein Lsp2 as a candidate. Using novel genetic tools, they demonstrate that Lsp2 is actively expressed both during development and in adults, and that ePR lowers its expression both in larvae and young adults. Depletion of Lsp2 during development is sufficient to lower translation in young adults and extends lifespan without compromising fitness. Finally, the authors show that Lsp2 is a reservoir of tyrosine and that tyr restriction in larvae mimics the effect of ePR and Lsp2 depletion on lifespan.

The demonstration that the storage protein Lsp2 mediates lifespan extension by ePR is novel, and the SILAC assay used to identify the proteins/amino acids carried over from larvae to adults is particularly elegant. I believe that the identification of storage proteins as potential targets for lifespan extension without fitness/ reproductive cost is highly attractive to a broad audience. Data and their presentation are of high quality. The experimental setup and the statistics used in the study are well defined.

The mechanism leading to Lsp2 depletion in larvae and in adults following ePR remains unclear, and appears to result from protein storage defects. This process is however further documented in the accompanying paper from Wang et al., which nicely complements the present study in this regard.

In my opinion, the main shortcoming remains the lack of mechanistic details to explain how the depletion of Lsp2 by ePR reduces the translation of ribosomal proteins in young adults to promote lifespan extension. The authors spent efforts to document that Lsp2 is expressed both in larvae and young adults, that ePR decreases its distinct expression in both stages, and that conditional Lsp2 depletion in larvae suppresses adult Lsp2 expression. Though they demonstrate that conditional Lsp2 knockdown in larvae has an effect on early adult translation and lifespan, whether conditional Lsp2 knockdown in young adults (days 0-2) has a similar effect is not addressed. As a result, it is not clear whether the reduced translation observed in young adults results from i) reduced amounts of ribosomal proteins carried over from larvae to adults, ii) a drop out in building blocks required for translation through a storage/recycling function of Lsp2 in larvae and adults, or iii) whether

Lsp2 in adults actively participate in the control of translation, possibly through a storage-independent function. The latter point is important to address in my opinion, in particular because the accompanying paper by Wang et al. suggests a direct control of translation by Lsp2 through 4E-BP. These points would be best addressed by further expanding the SILAC assay with and without conditional Lsp2 knockdown in larvae and young adults, respectively, to explore how Lsp2 differentially influences the persistence of larval proteins and the sourcing of amino acid substrates used for translation during early adult stages.

Specific remarks aimed at strengthening results and their interpretation

- To interpret the pSILAC assay, it would help to define the extent of protein labelling achieved after each pulse (heavy in larvae and medium in adults). It is likely that a significant fraction of proteins remains unlabeled after each pulse. This mitigates the interpretation of the labelled fractions, as well as of the light one that may not only result from feeding on unlabeled food during days 0-2.
- Is the food intake in larvae, young (d0-2) and older adults similar? Otherwise, should not the labelling values resulting from each pulse be normalized to the corresponding tracer concentration to interpret the protein turn-over and the origin of the amino acid substrates used for translation?
- What is the expression level of Lsp2 in adults +/- ePR later than day 3?
- Figure 5: Would His and Phe restriction phenocopy that of Tyr?
- In a previous study, Kosakamoto et al; 2022, the authors implicate Tyr in the control of mTOR and 4E-BP through ATF4. Given that Larval depletion of Lsp2 depletes Tyr in day2 adults (Fig.5b), and that this is associated to increased ATF4 signaling at least in new born adults (Ext data fig 9i, j), could a drop in mTOR-4E-BP signaling in early adults explain the decrease in translation?

Additional remarks aimed at improving manuscript clarity

- Page 6, while describing the pSILAC assay, it would help to mention the composition of the heavy and medium tracers in the main text (+8 Lys/+10 Arg vs +4Lys/+6Arg) if space allows to avoid switching to the methods to understand the experiment.
- Page 7 line 182 and 198. Rephrase or add a reference for His2B as a long-lived protein.
- Page 8 line 203. “some proteins can be particularly influenced by larval protein restriction”. The statement is too vague, please cite or rephrase.
- P. 9 l.236, fig.2L. It is not clear whether RU was fed only to young adults to repress early translation, or throughout adult life.
- P.11 l.301-302: please cite or refer to fig.4.
- Extended data fig. 8 a and b, please clarify the age of the animals analyzed.
- P.11l.312: I suggest rephrasing as follow: “we knocked down Lsp2 in the larval fat body"
- Fig.4 i-k. As this experiment would typically use Gene Swich drivers to avoid confounding effects of heterogeneous genetic backgrounds, please indicate in the text that all the lines used were isogenized to Canton S.
- P.12l.347: I suggest rephrasing as follow: “knocking down Lsp2 in larvae significantly reduced the overall composition of the two amino acid in young adults.

Looking forward to reading a revised version of the manuscript,
With best regards.

Referee #3

(Remarks to the Author)
The paper addresses the mechanisms by which yeast or protein restriction in the juvenile period extends adult lifespan in Drosophila. This is a potentially interesting topic, although its broader relevance outside insects is not clear. The study shows that there is an effect of yeast concentration in the diet during larval development on adult lifespan, although the magnitude of this effect depends on the adult diet and is absent at high yeast concentration. The authors also demonstrate carry over of amino acids taken up in the larval period into adult proteins. However, any mechanistic link between these two findings is unclear and the study has weaknesses that make it unsuitable for publication in its present form. Most important of these are: 1. conflation of data from yeast and protein restriction, despite the demonstration in the MS that these must be operating through different mechanisms, 2. Lack of replication and appropriate statistical analysis of lifespan data, 3. lack of clarity about the exact role of Lsp2 and other ‘storage’ proteins.
The paper is not well written. It needs a language edit and the authors need to explain why they did each experiment. For instance, on line 169 the authors say what they did next, but not why they did it, and there are several such examples.
Specific points:
1. The number of eggs in Figure 1 looks much too low
2. The authors state that the effects of juvenile and adult yeast concentration on lifespan were additive, but this is clearly not the case. In Figure 1n the effects of juvenile diet were greater in adults that were fed a lower yeast diet and were non-significant when the adults were exposed to 12% yeast. The effects of adult diet were also larger than those of juvenile diet. The data in Figure1 i-n should be analysed using Cox Proportional Hazards so that the effects of different yeast restrictions in I and m can be appropriately compared and the interaction between juvenile and adult diet in Figure 1n tested for statistical significance. It should be clearly stated that the effect of juvenile diet on lifespan was conditional on the adult diet – at the moment the opposite statement is made starting on line 101.
3. The data on the effect of amino acid (AA) concentration in Extended data Fig 1 show a consistent effect of larval diet on lifespan across a range of AA concentrations between 10% and 100%, with a reversal only when they were fed 1%. This

result is different from the result with varying yeast concentrations in Figure 1, where there is an indication of an interaction between adult and juvenile diet and the other way around, with the effect of juvenile diet being reduced by high, not low, yeast concentrations. This must indicate that the effect of AA concentration in the juvenile diet was being mediated by different mechanisms from the effect of yeast concentration. These data are thus not relevant to the effects of dietary yeast.
4. The data on insulin signalling in Extended Data Fig 2 are based on the effects of dietary yeast. The concentration of the larval diet is given, but it is not stated what was the concentration in the adult diet of the flies that were assessed – I assume 8% or 4%? This is important because the effects of larval diet were conditional on adult diet in Figure 1.
5. Line 131 has a statement that the effect of larval diet on insulin signalling was transitory. This is quite typical of the signalling domain but were the changes in gene expression at the transcript level maintained with time?
6. Line 144 refers to protein intake, but there was a disconnect in this study between the effects on lifespan of yeast and of amino acids as outlined above. The data presented are consistent with the hypothesis that the effects of yeast were not being mediated by its AA content.
7. In the SILAC analysis a synthetic diet was used, presumably at one of the AA concentrations mentioned earlier. The use of the synthetic diet is understandable given the question being asked but, as outlined above, it may not be relevant to the effects of a yeast diet. To be sure of that it would be necessary to label the yeast with H or M AAs.
8. Line 162 refers to H/M ratios but the authors have not defined H and M.
9. Starting on line 202 there is study of the effects of 'ePR' on the amino acid and protein composition of the flies. It is not stated whether this is yeast restriction or AA restriction on a defined diet. To understand the specific role of proteins synthesis in the juvenile stage it would be necessary to combine the SILAC study with the manipulation of the juvenile diet.
10. The data in Figure 2J do not prove the point being made – they could simply indicate that over expression of Rheb in fat body is harmful. To reach the conclusion claimed, it should be shown that suppressing TORC1 in the fat body mimics the effect of juvenile restriction and is epistatic to it. The overall conclusion of the paragraph is not supported by the data – a causal connection between reduced protein synthesis and increased lifespan is not demonstrated.
11. Starting on line 241 the study moves to using RNA-seq to detect changes in gene expression in response to larval diet. There is a suggestion of a feed-forward mechanism, with loss of Lsp2 protein during late larval stages leading to transcriptional suppression of the Lsp2 gene in adults. What would be the function of such a feed-forward mechanism? This section of the MS is hard to follow and the logic is not clear. The idea that protein restriction in larvae lowers LSP2 protein levels and hence substrates for translation in early adulthood makes some sense but would this not be expected to lead to higher rates of LSP synthesis in well fed adults?
12. Knockdown of Lsp in larval fat body did not mimic many of the effects of larval yeast deprivation on growth and female reproduction, bringing into question whether it operates through some of the same mechanisms as juvenile yeast restriction. The data in Figure 4j and k are worrying. No mention is made in the methods section of how mutant fly stocks were handled. If experimenters have backcrossed their mutants/transgenes into a standard background, as is essential, they normally say so, but there is no mention of this point. The data themselves are also unconvincing, with only partial epistasis, and they require both a repeat experiment and statistical analysis with Cox Proportional Hazards.
13. The data on tyrosine restriction do not contribute to the analysis because of the disconnect between the effects of AAs and of yeast, mentioned above. It would require further analysis to determine if the mechanisms of tyrosine and yeast restriction are overlapping.

Version 4:

Reviewer comments:

Referee #1

(Remarks to the Author)
This revision by Koskamoto … Obata resolves my remarks on its initial submission. I commend the authors for the extensive new work to make this paper robust with strong, important inferences. They now clearly demonstrate how Lsp2 operates as a nutrient state mechanism to regulate adult survival through translation. I am impressed by the extensive, added survival trials where they resolve questions about when and where Lsp2 must be altered to slow adult aging. They have added time course 'pulse chase' isotope tracking data to understand when and where Lsp2 is produced and carried forward. The text throughout integrates enough of the precedent literature to put their novel findings in a compelling context.

There are now (or recently) several papers triangulating on Lsp2 and the control of translation as keystones to the arch of aging (in Drosophila). These discoveries can suggest analogous molecular physiology in mammals. The field will make a quantum leap in understanding human aging once the parallels are identified.

I offer a few points where text should be clarified.

Lines 161-168 can be tightened. I suggest, ... "It was found that larval-derived nitrogen is slowly replaced by adult-derived nitrogen provisioned to eggs and in somatic tissue when adult diet is restricted. These observations pose fundamental questions. How are specific larval proteins carried into the adult stage, and how do animals transition from using larval-derived amino acids to adult-acquired ones for the synthesis of specific proteins. To answer these questions requires analysis of proteome-wide protein dynamics."

Line 359. Clarify 'decreased'. I suggest … 'was also significantly less than control in …."

Line 375. Be more specific viz 'continuous'. I suggest … 'Lsp2 knockdown by RNAi though out larval and adult stages' …

Lines 445-448. Please clarify. I don’t understand what is being compared to what in this sentence.

Line 497. The author is Wang … not Zhai?

Referee #2

(Remarks to the Author)
The authors have addressed all my comments appropriately. The manuscript now robustly demonstrates that the level of Lsp2 controlled by larval diet is a main regulator of adult lifespan, a process that associates with translational control through regulating the levels of CytoRP.
See below additional points that the authors may want to clarify in the text.
Ligne 288
I find the experiment with Rheb overexpression (ext.data.fig5) difficult to interpret. It could be deleted as it does not really address translation. What is the rationale for using a fat body driver? Also, Rheb OE seems to unexpectedly extend lifespan in fed animals. Could this be due to RU treatment? The experiment does not include controls for the effect of RU. This may also impact the interpretation of fig. 4r. I recommend that the authors clearly discuss what is comparable in these experiments, what is not, and why. In both figures, the lifespan differences are very mild and really hard to see. I suggest including a graph with the medium lifespans to simplify the presentation of the results for the reader.
Ligne 480: Interestingly, Ile restriction induced a stronger developmental delay than Phe or His restriction, suggesting that Lsp2 can act as a buffer to support larval development under nutritional stress by providing stored amino acids (Fig. 5j).
It would be best to include an LSp2 KD in this experiment to support this hypothesis.
Ligne 177: diet containing "medium" isotope-labelled Lys (+4 Lys; D4)
Shouldn’t D4 be replaced by 13C4?
Ligne 281: The suppressive effect of ePR on translation lasted only one week (Fig. 2o), consistent with the fact that decreased abundance of cytoRPs by ePR can be rescued in this time frame.
I’m not sure I understand this sentence. Which figure is referred to for the rescue

Referee #3

(Remarks to the Author)
The authors have made several beneficial modifications to their study. However, I remain unconvinced about some aspects of the nutritional manipulations.
The authors state that Interestingly, ePR-flies exhibit a further increase in lifespan when combined with adult protein restriction (4% vs 1% yeast extract [YE]) (Fig. 1q,r).' However, earlier work has suggested that yeast extract is toxic to flies, with increasing concentrations decreasing lifespan without any benefit to fecundity (Wong et al doi: 10.1093/gerona/62.10.1071). Furthermore, yeast extract is not equivalent to amino acids. Earlier in the text the authors show that they can manipulate amino acids directly, both in larvae and in adults, and they should stick to that method throughout the rest of the paper rather than introducing the use of yeast extract. The data in SF1 also do not address the point because they combine larval yeast with adult amino acids, rather than larval amino acids with adult amino acids. The issue of the interaction effect is also partially swept under the carpet by simply narrowing the range of concentrations analysed. We need to see the data for a full physiological range of concentrations of amino acids. More generally, since the authors have the relevant methodology available, they should manipulate amino acids directly rather than making conclusions about protein restriction based on manipulation of yeast. Mostly this has been done already, especially in the nice work on SILAC, but the use of yeast extract and the attempt to claim equivalence of yeast with protein without mechanistic evidence should be remedied.

Version 7:

Reviewer comments:

Referee #1

(Remarks to the Author)
The new revision refines and elevates both the data and coherent story of the project. Light editing is needed in a few spots.

Line 112: seems to be missing some words.

Line 398: most readers will not grasp the logic for why ‘not demonstrate’. It is because the ts system can’t disentangle genotype and temperature affects. Somehow make the issue clearer.

Line 410: ‘the effect was mild’. The quantity of change should be made explicit. This is a central result, a bit buried in the extended data. If the result is ‘mild’, show us how much so, warts and all.

Referee #2

(Remarks to the Author)
The authors have addressed all my comments appropriately, I do not have additional remarks, the study is ready for publication in my opinion.

Referee #3

(Remarks to the Author)
I am satisfied with the latest round of revisions

Open Access This Peer Review File is licensed under a Creative Commons Attribution 4.0 International License, which permits use, sharing, adaptation, distribution and reproduction in any medium or format, as long as you give appropriate credit to the original author(s) and the source, provide a link to the Creative Commons license, and indicate if changes were made.
In cases where reviewers are anonymous, credit should be given to 'Anonymous Referee' and the source.
The images or other third party material in this Peer Review File are included in the article's Creative Commons license, unless indicated otherwise in a credit line to the material. If material is not included in the article's Creative Commons license and your intended use is not permitted by statutory regulation or exceeds the permitted use, you will need to obtain permission directly from the copyright holder.
To view a copy of this license, visit https://creativecommons.org/licenses/by/4.0/

Referee #1 (Remarks to the Author):

Overall

I like the theme and insights of this creative paper. Food intake (protein) affects aging. Long research has sought to understand the underlying sensing, signaling and metabolic mechanisms. Restriction in juvenile stages is effective. Kosakamoto provides an expansive and exciting study for how larval storage proteins (Lsp2) regulates adult survival. After parsing the many data, I can summarize the novel, salient conclusion: Larval diet restriction (ePR) extends adult lifespan and alters metabolic/protein features of the young adult including translation. Among various proteins, Lsp2 is reduced in the adult by ePR. RNAi of Lsp2 in larvae reduces total translation in the young adult and extends female survival.

There are many data in the paper that are interpreted with interesting implications, for instance about translation control, protein turnover, the carry-over of molecules from larvae to adults, and costs of reproduction. Yet, I feel the authors over-emphasize incidental observations and make many conclusions based on associational inferences.

Still, the paper has remarkable insights and advances a central objective in aging research. It will have more impact with streamlining and if it establishes focused, robust inferences on the function of Lsp2 that arise following ePR.

An apology: My review is perhaps overly detailed and too personal (and too long). Being so intimate with the addressed questions, I write to engage the authors constructively and to share my experience. But I pull no punches.

Response 1-1. We thank the reviewer for this generous evaluation and the critical comments. We greatly value this constructive feedback, which will help us improve the clarity, rigor, and focus of the study.

Introduction

Needs to be more specific with relevant background. It feels vague. The first sentences are strawmen (line 46-48) because the field does consider juvenile diet, as demonstrated in the rest of the paragraph. I suggest the paper opens with the phenomenon as a given and state a plan to understand its underlying mechanism. Discuss specifics of current knowledge and proposed mechanisms for early-life impact on adult aging: Size, metabolism, epigenetics, etc.

Provide details from the work of Stefana 2017 (which is swept under the rug), and our work (Tu/Tatar 2003, also under the rug). Tu used an almost identical design but without finding a positive effect on adult lifespan. Although awkward, explicitly recognize these ambiguities. It is better now than when others try to reproduce your observations (with or without success).

No need to justify the fly model (second intro paragraph). Instead, set up your story by telling the reader why and what we need to understand, and what you will reveal.

Response 1-2. We will revise the Introduction to be more specific and to provide appropriate background. We will incorporate the work of Stefana (2017) and Tu/Tatar (2003), explicitly recognizing both consistencies and ambiguities in the literature. We will remove unnecessary justification for the fly model and instead set up the study by outlining the key mechanistic questions.

Results

Larval diets were reduced from 8% yeast to 2% or 1%. The Obata stocks are maintained on 6% yeast extract. 8% may be a stress condition whereby these controls are not ‘normal’.

Response 1-3. We will clarify why we used 8% yeast as the control condition.

Larval protein restriction reduces ovariole number. Include reference #18 on line 97. Please add data for the 2% ePR. Quantify/statistically establish the subjective claim ‘milder’ (line 99).

Response 1-4. We will include quantitative data on ovariole number under 2% ePR and provide statistical comparisons to support the description of “milder” effects.

Good data on additivity with adult DR. Then the paragraph turns to adults on 0% protein where the benefit of ePR is lost. This observation does not imply, however, ‘the outcome of ePR can be variable [via adult dietary conditions] among experimental conditions (refs 6, 18)’ (line 116). This explanation obscures the real ambiguities. I can speak for ref 18 (Tu/Tatar 2003): Tu held the 3rd instar with no yeast (rather than 1% or 2%). Tu/Tatar maintain flies with baker’s yeast, while Obata uses brewer’s yeast. Differences are fine, but readers need to understand specifics and likely causes.

Response 1-5. We will revise the relevant paragraph, add the Tu/Tatar 2003 citation, and clarify that differences between studies likely stem from specific diet compositions.

Line 120. Tu (2003) established that ePR modulates insulin signaling in the young adult. That paper also sets a stage for some (then unknown) impact of ePR on JH (we would now say, via insulin), which further connects Kosakamoto to the paper of Kim/Pickering (early adult translation).

Response 1-6. We will add this reference and explain how our findings extend the earlier observations on insulin signaling and potential links to juvenile hormone.

The paragraph at line 133 does not convince that ePR ‘affects nutrient sensing pathways transiently in early adulthood’. Readers will not accept that ATF4 is purely a feature of amino acid sensing. The role of phosphor-eIF2 is not obvious, and it is decoupled from ATF4. I think the paper works better without this paragraph.

Response 1-7. We will remove the ATF4 paragraph and associated data, as suggested.

The isotope study. Please (again) excuse my self-reference, but we did a similar isotope larvae/adult tracing study and found similar conclusions (Min … O'Brien JIP 2006; O'Brien … Tatar 2008 Current Biology. I attach a PDF of 2008 because the paper is hard to find). We made 15N & 13C labeled yeast, and 14N &12C. Larvae and adults were fed alternative isotope forms. We collected somatic tissue and eggs across the aging cohort. From a low concentration of label, we quantified turnover rates and equilibria, all in the context of adult DR. We found many features reported in the current submission. These papers complement, so please educate and guide the reader.
Response 1-8. We will revise the text to incorporate and cite the isotope-labeling studies, highlighting how our results complement these findings.

The global enrichment analysis (paragraph starting at line 184) is subjective and tangential. Can these inferences be supported with statistics? Are they essential to the paper's conclusion?
Response 1-9. While our enrichment analysis is statistically supported, it is not central to the main conclusions. We will move these results to the Supplementary Figures.

Proteomics. ePR alters the quantity and quality of amino acids in young adults, but ePR was not studied with the labeled samples. It is a conjecture that the altered profiles arise from carry-over. We can't distinguish effects from ePR relative to adult feeding.
Response 1-10. We will perform pSILAC analysis in flies subjected to ePR as well as in flies with Lsp2-RNAi. These experiments will directly test whether ePR shapes the adult proteome via storage proteins.

The protein profiles in young adults suggest there is reduced translation capacity following ePR. Kim/Pickering (Nature Comm 2023) recently reported young adults have a 'spike' in translation. Suppressing the spike increased life expectancy. Kosakamoto now sees high translation in young adults that is blunted by ePR. They propose this contributes to slow aging. Kosakamoto activates mTORC1 in ePR treated flies to test this idea, and this reduces their longevity. However, Kosakamoto increases mTORC1 across all adult ages, not just in the phase where ePR is thought to inhibit translation. Most would say that elevating mTORC1 across adulthood is bad for survival. A better design requires a time-limited rescue (as in Kim/Pickering), and analysis of age-specific mortality following the manipulation period.
Response 1-11. To better address the causal role of translation, we will restrict mTORC1 activation to early adulthood (as in Kim/Pickering, Nat Commun 2023). This design will more directly test whether blunting the early translation spike mediates the longevity effect of ePR.

The research sequence at line 241-243 seems strange. Kosakamoto already conducted the proteomics to identify the 'molecules', so why bother with transcriptome analysis?

Response 1-12. We will streamline this section to clarify the logic and sequencing of proteomics versus transcriptomics.

Lsp2 mRNA is selected as the focal DEG, but its expression in the adult head is not 'unexpected' (line 265). It was documented in 1990 (Benes et al.) and verified in expression studies (see FlyBase). Kosakamoto mention Benes in the discussion. To justify the Lsp2 focus at this point, directly talk about the storage protein data of Kim/Pickering (Nature Comm 2023). They identified Lsp2 as a dynamic factor in the early soluble fraction. Use this to set up the appropriate question: where does the adult LSP2 come from? The LSP2 may arise from adult translation because adults have high Lsp2 mRNA (PCR) and protein, whereas adults from ePR have lower Lsp2 mRNA and protein. (And … why Lsp2 relative to all the other storage proteins?)

Response 1-13, We will revise the text accordingly.

I am confused by the wording in lines 279-280 and 281-283. If the idea is that ePR represses adult Lsp2 mRNA, why discuss wandering larvae and pupae? I am not convinced that knockdown of Lsp2 (or Fbp) across all larval stages suggests a 'feed forward mechanism to regulate storage protein expression'. Might knockdown block larval fat body development, which carries across to the adult. Not 'nutritional memory' (per ePR) but stunted tissue development?

Response 1-14. We will examine larval fat body morphology to rule out developmental artifacts. In addition, we will cite the independent work from Dr. Zhao's group supporting a feed-forward mechanism of Lsp2 regulation.

The hypothesis of line 297-308. The premise is weak. Turn-over of LSP occurs in the adult, as it does for many larval-derived proteins (except perhaps those in the cuticle). See O'Brien (2008) who quantified nitrogen-molecule turnover as a function of adult DR. Kosakamoto could benefit from pSILAC of adults derived from the three larval ePR conditions. This would quantify how much LSP derives from larvae and from adult feeding and estimate the turnover in the adult. Those data would test if the 'observed reduction' (via ePR) is caused by less carryover or reduced production in the adult (need not be via 'suppressed transcription'). One caveat concerns the issue of recycled amino acids.

Response 1-15. As noted above (Response 1-10), we will perform pSILAC in adults derived from 8% versus 1% yeast larval diets. This will allow us to distinguish between reduced carry-over and reduced adult production.

The development-limited Lsp2 RNAi data are central to the paper. Kosakamoto buries them behind the continuous knockdown (larva through adulthood) data. The continuous data confound what they want to understand about ePR with the impacts from Lsp2 RNAi in the adult.
The project needs fuller data from the larval-only knockdown. How does this effect the omic measures, interact with ePR (viz longevity), affect males? How effective is the larval RNAi to reduce the adult Lsp2 protein, and over what time course? Address a genetic background issue: gal80 controls time of expression but compares different genetic backgrounds. Even with some backcrossing, there is much room for second site effects (especially in survival data). I suggest Kosakamoto measures phenotypes of both genotypes (lacz and RNAi) from animals continuously at the restrictive temperature. They should look the same. I recommend they replicate all survival trials to demonstrate the result is reproducible (one 'significant' trial is weak sauce). Include males and give data in the main figure.

Response 1-16. We agree that larva-specific Lsp2-RNAi is critical. Although larval knockdown also reduces adult Lsp2 levels, we will perform additional larval-stage–specific RNAi experiments in both males and females. We will quantify Lsp2 protein reduction over time, replicate survival assays, and provide Cox proportional hazards analyses. We will also control for genetic background effects by including appropriate comparisons at restrictive temperature.

The tyrosine result does not demonstrate Lsp2 as the cause, or visa versa. I suggest controls to show that lifespan is not increased when you reduce amino acids besides Tyr, Phe or His (Lsp2 specialties). Line 352-353 connects the exome observations to aging through some previous work (refs 22, 27). What is meant by 'highly sensitive to Tyr intake'? Explicitly discuss results from Parkhitko (eLIFE 2020), which established the role of Tyr in the control of aging, although paradoxically in the opposite direction.

Response 1-17. We will extend our larval amino acid restriction experiments to include select amino acids such as Phe, His, Ile, Thr, Ser, or Pro. We will also explicitly discuss the findings of Parkhitko et al. regarding tyrosine's role in ageing.

Discussion

In my opinion, the work does not yet establish its conclusion of line 364-366 because the referenced results were based on life-long Lsp2 RNAi, not larval-limited.

Response 1-18. We will revise this section to ensure that our conclusions are based only on larval-specific RNAi results, not continuous knockdowns.

I am not sure Kosakamoto's data support the conclusion asserted on lines 370-372. Kosakamoto

associates a storage protein with features of translation control. While knockdown of Lsp2 in the larvae reduces total protein translation in the young adult, the work does not prove the change in translation is required to slow aging. The design with TORC1 is not convincing. What I do see is that reducing Lsp2 in the larval stage (by RNAi) can extend lifespan in females.

Response 1-19. We will temper our conclusions and revise the text accordingly.

Indirect inferences are used for big conclusions of the discussion. I enjoyed these ideas, especially in the last several paragraphs. They are broad and exciting, but I am not convinced they are supported by the offered data.

Response 1-20. We will edit the discussion to better align speculative ideas with the supporting data, ensuring that broader interpretations are clearly framed as hypotheses.

Referee #2 (Remarks to the Author):

General comments

In this study, Kosakamoto et al. investigate the mechanisms mediating lifespan extension by protein restriction during development. Using Drosophila melanogaster, a relevant model for the study of longevity, they establish that 24-hours protein restriction in early third instar larvae (ePR) is sufficient to extend the lifespan of wild type adult animals. The effect of this treatment on lifespan with respect to diets fed to adult animals is investigated, along with the fitness cost on growth and reproduction, as well as the effect on nutrient signaling pathways in young adults. They further develop a SILAC assay using differentially labelled amino acids fed to larvae and adults in order to monitor protein turnover across developmental stages. They demonstrate that larval proteins contribute significantly to the adult proteome, in particular ribosomal proteins. Additional analyzes of the amino acid content and of the proteome indicate that ePR impacts amino acids balance and decreases translation in young adults, a process that has previously been linked to lifespan extension, thereby providing a mechanistic link between ePR and lifespan. The authors next search for a functional mediator of this process. Using transcriptomics analyzes, they identify the storage protein Lsp2 as a candidate. Using novel genetic tools, they demonstrate that Lsp2 is actively expressed both during development and in adults, and that ePR lowers its expression both in larvae and young adults. Depletion of Lsp2 during development is sufficient to lower translation in young adults and extends lifespan without compromising fitness. Finally, the authors show that Lsp2 is a reservoir of tyrosine and that tyr restriction in larvae mimics the effect of ePR and Lsp2 depletion on lifespan.
The demonstration that the storage protein Lsp2 mediates lifespan extension by ePR is novel, and the SILAC assay used to identify the proteins/amino acids carried over from larvae to adults is particularly elegant. I believe that the identification of storage proteins as potential targets for lifespan extension without fitness/ reproductive cost is highly attractive to a broad audience. Data and their presentation are of high quality. The experimental setup and the statistics used in the study are well defined.
The mechanism leading to Lsp2 depletion in larvae and in adults following ePR remains unclear, and appears to result from protein storage defects. This process is however further documented in the accompanying paper from Wang et al., which nicely complements the present study in this regard.
Response 2-1. We greatly appreciate the reviewer's generous and encouraging comments. We are pleased that the novelty of the findings and the strength of the SILAC assay were recognized.

In my opinion, the main shortcoming remains the lack of mechanistic details to explain how the depletion of Lsp2 by ePR reduces the translation of ribosomal proteins in young adults to promote

lifespan extension. The authors spent efforts to document that Lsp2 is expressed both in larvae and young adults, that ePR decreases its distinct expression in both stages, and that conditional Lsp2 depletion in larvae suppresses adult Lsp2 expression. Though they demonstrate that conditional Lsp2 knockdown in larvae has an effect on early adult translation and lifespan, whether conditional Lsp2 knockdown in young adults (days 0-2) has a similar effect is not addressed. As a result, it is not clear whether the reduced translation observed in young adults results from i) reduced amounts of ribosomal proteins carried over from larvae to adults, ii) a drop out in building blocks required for translation through a storage/recycling function of Lsp2 in larvae and adults, or iii) whether Lsp2 in adults actively participate in the control of translation, possibly through a storage-independent function. The latter point is important to address in my opinion, in particular because the accompanying paper by Wang et al. suggests a direct control of translation by Lsp2 through 4E-BP. These points would be best addressed by further expanding the SILAC assay with and without conditional Lsp2 knockdown in larvae and young adults, respectively, to explore how Lsp2 differentially influences the persistence of larval proteins and the sourcing of amino acid substrates used for translation during early adult stages.
Response 2-2. We agree this is a central point. To address it, we will perform Lsp2-RNAi specifically in early adulthood and assess effects on translation and lifespan. In addition, we will extend the pSILAC analysis under Lsp2-RNAi conditions to directly test whether Lsp2 influences persistence of larval proteins and amino acid sourcing for early-adult translation.

Specific remarks aimed at strengthening results and their interpretation

- To interpret the pSILAC assay, it would help to define the extent of protein labelling achieved after each pulse (heavy in larvae and medium in adults). It is likely that a significant fraction of proteins remains unlabeled after each pulse. This mitigates the interpretation of the labelled fractions, as well as of the light one that may not only result from feeding on unlabeled food during days 0-2.

Response 2-3. We will measure labeling efficiency at the end of the larval stage to quantify incorporation of heavy isotopes into the larval proteome. Adult labeling efficiency with medium isotopes was already assessed in the original manuscript.

- Is the food intake in larvae, young (d0-2) and older adults similar? Otherwise, should not the labelling values resulting from each pulse be normalized to the corresponding tracer concentration to interpret the protein turn-over and the origin of the amino acid substrates used for translation?

Response 2-4. We will include food intake comparisons for ePR and Lsp2-RNAi flies, and interpret the pSILAC data with these controls in mind. Additional pSILAC datasets will also help refine interpretation of turnover and substrate origin.

- What is the expression level of Lsp2 in adults +/- ePR later than day 3?

Response 2-5. We will perform qPCR analyses of Lsp2 expression in later adult stages, with and without ePR.

- Figure 5: Would His and Phe restriction phenocopy that of Tyr?

Response 2-6. We will test larval restriction of His and Phe using synthetic diets, and compare the phenotypes to those induced by Tyr restriction.

- In a previous study, Kosakamoto et al; 2022, the authors implicate Tyr in the control of mTOR and 4E-BP through ATF4. Given that Larval depletion of Lsp2 depletes Tyr in day2 adults (Fig.5b), and that this is associated to increased ATF4 signaling at least in new born adults (Ext data fig 9i, j), could a drop in mTOR-4E-BP signaling in early adults explain the decrease in translation?

Response 2-7. We will perform SUnSET assays in Rheb-overexpressing flies with or without ePR to assess the contribution of mTOR activity to translation. In line with Reviewer 1's advice, we will remove the ATF4 data, as it is not central to the current study.

Additional remarks aimed at improving manuscript clarity

- Page 6, while describing the pSILAC assay, it would help to mention the composition of the heavy and medium tracers in the main text (+8 Lys/+10 Arg vs +4Lys/+6Arg) if space allows to avoid switching to the methods to understand the experiment.

Response 2-8. We will specify heavy (+8 Lys/+10 Arg) and medium (+4 Lys/+6 Arg) tracers in the main text for clarity.

- Page 7 line 182 and 198. Rephrase or add a reference for His2B as a long-lived protein.

Response 2-9. We will rephrase the text.

- Page 8 line 203. "some proteins can be particularly influenced by larval protein restriction". The statement is too vague, please cite or rephrase.

Response 2-10. We will revise the statement for precision.

- P. 9 l.236, fig.2L. It is not clear whether RU was fed only to young adults to repress early translation, or throughout adult life.

Response 2-11. We will clarify whether RU was administered only during early adulthood or throughout life.

- P.11 l.301-302: please cite or refer to fig.4.

- Extended data fig. 8 a and b, please clarify the age of the animals analyzed.
- P.11l.312: I suggest rephrasing as follow: “we knocked down Lsp2 in the larval fat body"
- Fig.4 i-k. As this experiment would typically use Gene Swich drivers to avoid confounding effects of heterogeneous genetic backgrounds, please indicate in the text that all the lines used were isogenized to Canton S.
- P.12l.347: I suggest rephrasing as follow: “knocking down Lsp2 in larvae significantly reduced the overall composition of the two amino acid in young adults.

Response 2-12. We will revise these points accordingly.

Looking forward to reading a revised version of the manuscript,
With best regards.

Response 2-13. We are very encouraged by the reviewer’s concluding remarks. Thank you so much for the supportive assessment.

Referee #3 (Remarks to the Author):

The paper addresses the mechanisms by which yeast or protein restriction in the juvenile period extends adult lifespan in Drosophila. This is a potentially interesting topic, although its broader relevance outside insects is not clear. The study shows that there is an effect of yeast concentration in the diet during larval development on adult lifespan, although the magnitude of this effect depends on the adult diet and is absent at high yeast concentration. The authors also demonstrate carry over of amino acids taken up in the larval period into adult proteins. However, any mechanistic link between these two findings is unclear and the study has weaknesses that make it unsuitable for publication in its present form. Most important of these are: 1. conflation of data from yeast and protein restriction, despite the demonstration in the MS that these must be operating through different mechanisms, 2. Lack of replication and appropriate statistical analysis of lifespan data, 3. lack of clarity about the exact role of Lsp2 and other 'storage' proteins.

The paper is not well written. It needs a language edit and the authors need to explain why they did each experiment. For instance, on line 169 the authors say what they did next, but not why they did it, and there are several such examples.

Response 3-0. We thank the referee for the constructive assessment. We will carefully revise the manuscript to make the rationale for each experiment clear, and to emphasize why we believe this study provides impactful insights into how a brief nutritional environment can shape long-term lifespan. We will also edit the text for clarity and flow.

Specific points:

1. The number of eggs in Figure 1 looks much too low

Response 3-1, We thank the reviewer for this notion. We will repeat the experiment and compare the number of eggs in our standard condition.

2. The authors state that the effects of juvenile and adult yeast concentration on lifespan were additive, but this is clearly not the case. In Figure 1n the effects of juvenile diet were greater in adults that were fed a lower yeast diet and were non-significant when the adults were exposed to 12% yeast. The effects of adult diet were also larger than those of juvenile diet. The data in Figure1 i-n should be analysed using Cox Proportional Hazards so that the effects of different yeast restrictions in I and m can be appropriately compared and the interaction between juvenile and adult diet in Figure 1n tested for statistical significance. It should be clearly stated that the effect of juvenile diet on lifespan was conditional on the adult diet – at the moment the opposite statement is made starting on line 101.

Response 3-2. We will reanalyse these lifespan data using Cox proportional hazards models to test for main effects and interactions. The text will be revised accordingly. We also note that 12% yeast represents an extremely high and toxic condition, which may explain the lack of

additivity.

3. The data on the effect of amino acid (AA) concentration in Extended data Fig 1 show a consistent effect of larval diet on lifespan across a range of AA concentrations between 10% and 100%, with a reversal only when they were fed 1%. This result is different from the result with varying yeast concentrations in Figure 1, where there is an indication of an interaction between adult and juvenile diet and the other way around, with the effect of juvenile diet being reduced by high, not low, yeast concentrations. This must indicate that the effect of AA concentration in the juvenile diet was being mediated by different mechanisms from the effect of yeast concentration. These data are thus not relevant to the effects of dietary yeast.

Response 3-3. We apologize for any confusion. Our interpretation is that both datasets support a similar principle: extreme conditions (very high yeast, or 0% amino acids) abolish the ePR effect, whereas intermediate conditions show additive effects with adult DR. To strengthen this point, we will test 0% yeast and/or 300% amino acids in the adult diet. In our hands, yeast and amino acid manipulations yield largely comparable phenotypes.

4. The data on insulin signalling in Extended Data Fig 2 are based on the effects of dietary yeast. The concentration of the larval diet is given, but it is not stated what was the concentration in the adult diet of the flies that were assessed – I assume 8% or 4%? This is important because the effects of larval diet were conditional on adult diet in Figure 1.

Response 3-4. The adult diet was our standard 6% yeast diet (not yeast extract). We will revise the text to clarify this. We emphasize that larval diet effects are generally consistent, but become conditional under extreme adult diets (e.g., 0% AA or 12% yeast).

5. Line 131 has a statement that the effect of larval diet on insulin signalling was transitory. This is quite typical of the signalling domain but were the changes in gene expression at the transcript level maintained with time?

Response 3-5. We will perform qPCR at day 7 to determine whether transcript-level changes persist.

6. Line 144 refers to protein intake, but there was a disconnect in this study between the effects on lifespan of yeast and of amino acids as outlined above. The data presented are consistent with the hypothesis that the effects of yeast were not being mediated by its AA content.

Response 3-6. We respectfully disagree. As explained in our response 3-3, yeast and amino acids are equivalent. To test this more directly, we will perform larval-stage experiments combining yeast restriction with amino acid supplementation, as well as general amino acid restriction, to

assess whether yeast effects are mediated via amino acids.

7. In the SILAC analysis a synthetic diet was used, presumably at one of the AA concentrations mentioned earlier. The use of the synthetic diet is understandable given the question being asked but, as outlined above, it may not be relevant to the effects of a yeast diet. To be sure of that it would be necessary to label the yeast with H or M AAs.

Response 3-7. We believe responses 3-3 and 3-6 clarify that yeast and amino acid manipulations produce similar outcomes. Therefore, we consider additional pSILAC using isotope-labelled yeast unnecessary.

8. Line 162 refers to H/M ratios but the authors have not defined H and M.

Response 3-8. We will revise the text to define H and M clearly.

9. Starting on line 202 there is study of the effects of 'ePR' on the amino acid and protein composition of the flies. It is not stated whether this is yeast restriction or AA restriction on a defined diet. To understand the specific role of proteins synthesis in the juvenile stage it would be necessary to combine the SILAC study with the manipulation of the juvenile diet.

Response 3-9. We will perform pSILAC analysis in ePR flies to directly link juvenile restriction to proteomic changes.

10. The data in Figure 2J do not prove the point being made – they could simply indicate that over expression of Rheb in fat body is harmful. To reach the conclusion claimed, it should be shown that suppressing TORC1 in the fat body mimics the effect of juvenile restriction and is epistatic to it. The overall conclusion of the paragraph is not supported by the data – a causal connection between reduced protein synthesis and increased lifespan is not demonstrated.

Response 3-10. Previous work (Kim et al., Nat Commun 2023) shows that TORC1 suppression in early adulthood reduces translation and extends lifespan. Building on this, we will perform early-adult–specific Rheb overexpression, with and without ePR, to assess effects on translation and lifespan. We believe this will provide sufficient causal evidence.

11. Starting on line 241 the study moves to using RNA-seq to detect changes in gene expression in response to larval diet. There is a suggestion of a feed-forward mechanism, with loss of Lsp2 protein during late larval stages leading to transcriptional suppression of the Lsp2 gene in adults. What would be the function of such a feed-forward mechanism? This section of the MS is hard to follow and the logic is not clear. The idea that protein restriction in larvae lowers LSP2 protein levels and hence substrates for translation in early adulthood makes some sense but would this not be expected to lead

to higher rates of LSP synthesis in well fed adults?

Response 3-11. We appreciate this insightful point. We hypothesize that this feed-forward mechanism establishes a form of nutritional memory: Lsp2 abundance reflects protein availability and may set the anabolic rate for adulthood. This aligns with the concept of a predicted adaptive response (PAR), in which early-life nutrition calibrates later-life physiology. We will revise the text to explain this more clearly.

12. Knockdown of Lsp in larval fat body did not mimic many of the effects of larval yeast deprivation on growth and female reproduction, bringing into question whether it operates through some of the same mechanisms as juvenile yeast restriction. The data in Figure 4j and k are worrying. No mention is made in the methods section of how mutant fly stocks were handled. If experimenters have backcrossed their mutants/transgenes into a standard background, as is essential, they normally say so, but there is no mention of this point. The data themselves are also unconvincing, with only partial epistasis, and they require both a repeat experiment and statistical analysis with Cox Proportional Hazards.

Response 3-12. We will add detailed descriptions of fly stock handling and genetic background in the Methods. We will repeat these experiments and present the data with Cox proportional hazards analysis.

13. The data on tyrosine restriction do not contribute to the analysis because of the disconnect between the effects of AAs and of yeast, mentioned above. It would require further analysis to determine if the mechanisms of tyrosine and yeast restriction are overlapping.

Response 3-13. We will extend our larval-stage amino acid manipulation experiments, as outlined in Response 1-17 and 2-6, to clarify the relationship between specific amino acids and yeast restriction.

Referee #1 (Remarks to the Author):
Overall
I like the theme and insights of this creative paper. Food intake (protein) affects aging. Long research has sought to understand the underlying sensing, signaling and metabolic mechanisms. Restriction in juvenile stages is effective. Kosakamoto provides an expansive and exciting study for how larval storage proteins (Lsp2) regulates adult survival. After parsing the many data, I can summarize the novel, salient conclusion: Larval diet restriction (ePR) extends adult lifespan and alters metabolic/protein features of the young adult including translation. Among various proteins, Lsp2 is reduced in the adult by ePR. RNAi of Lsp2 in larvae reduces total translation in the young adult and extends female survival.
There are many data in the paper that are interpreted with interesting implications, for instance about translation control, protein turnover, the carry-over of molecules from larvae to adults, and costs of reproduction. Yet, I feel the authors over-emphasize incidental observations and make many conclusions based on associational inferences.
Still, the paper has remarkable insights and advances a central objective in aging research. It will have more impact with streamlining and if it establishes focused, robust inferences on the function of Lsp2 that arise following ePR.
An apology: My review is perhaps overly detailed and too personal (and too long). Being so intimate with the addressed questions, I write to engage the authors constructively and to share my experience. But I pull no punches.
Response 1-1. We thank Prof. Marc Tatar for this generous evaluation and the critical comments. We greatly value this constructive feedback, which helped us improve the clarity, rigor, and focus of the study significantly. As you will see in our following point-by-point responses, we have substantially revised our manuscript by adding new experimental data. I hope you will be satisfied with our revision.

Introduction
Needs to be more specific with relevant background. It feels vague. The first sentences are strawmen (line 46-48) because the field does consider juvenile diet, as demonstrated in the rest of the paragraph. I suggest the paper opens with the phenomenon as a given and state a plan to understand its underlying mechanism. Discuss specifics of current knowledge and proposed mechanisms for early-life impact on adult aging: Size, metabolism, epigenetics, etc.
Provide details from the work of Stefana 2017 (which is swept under the rug), and our work (Tu/Tatar 2003, also under the rug). Tu used an almost identical design but without finding a positive effect on adult lifespan. Although awkward, explicitly recognize these ambiguities. It is better now than when others try to reproduce your observations (with or without success).
No need to justify the fly model (second intro paragraph). Instead, set up your story by telling the reader why and what we need to understand, and what you will reveal.
Response 1-2. We have revised the Introductory paragraph (including the Summary) to be more specific and to provide appropriate background. Due to the limit of the length, we did not fully explain the detail of the proposed mechanisms of nutritional programming, but we do have incorporated the work of Stefana et al., Nat Commun, 2017 and Tu and Tatar, Aging Cell, 2003, to discuss what has been known in the field of larval dietary restriction and lifespan. We hope that the revised manuscript flows better and is more focused.

Results
Larval diets were reduced from 8% yeast to 2% or 1%. The Obata stocks are maintained on 6% yeast extract. 8% may be a stress condition whereby these controls are not 'normal'.
Response 1-3. Our standard fly food contains 4.5% cornmeal in addition to 6% yeast extract (brewer's yeast) and sugar, whereas the ePR diet does not include cornmeal and is prepared by whole yeast (baker's yeast). The exclusion of cornmeal was intended to simplify food preparation, facilitate precise nutritional manipulation, and avoid variability in dietary composition due to batch-to-batch differences in cornmeal. We first prepared ePR control diet with 6% yeast, but it resulted in delayed

development. Increasing yeast content to 8% resolved this issue, assuming 8% yeast is appropriate as the control (fully-fed) diet. This point has been added to the methods section as shown below.

Methods: Dietary manipulations
For larval yeast restriction, diets containing 8%, 2%, and 1% yeast were prepared using baker's yeast (Lesaffre, Saf-Instant Red), 6% glucose (FUJIFILM Wako Pure Chemical Corporation, 049-31165), 1% agar (FUJIFILM Wako Pure Chemical Corporation, 010-15815), 0.3% propionic acid (FUJIFILM Wako Pure Chemical Corporation, 163-04726), and 0.15% methyl p-hydroxybenzoate (FUJIFILM Wako Pure Chemical Corporation, 132-02635). Cornmeal was omitted from these formulations to simplify food preparation, facilitate precise nutritional manipulation, and avoid compositional variability arising from batch-to-batch differences in cornmeal. Increasing the yeast content to 8% (from the 6% used in the standard diet) resolved the slight developmental delay associated with the omission of cornmeal; consequently, the 8% yeast diet was utilised as the control (fully-fed) condition.

Larval protein restriction reduces ovariole number. Include reference #18 on line 97. Please add data for the 2% ePR. Quantify/statistically establish the subjective claim 'milder' (line 99).
Response 1-4. We cited the reference and included quantitative data on ovariole numbers under 2% ePR (Fig. 1j) as shown below.

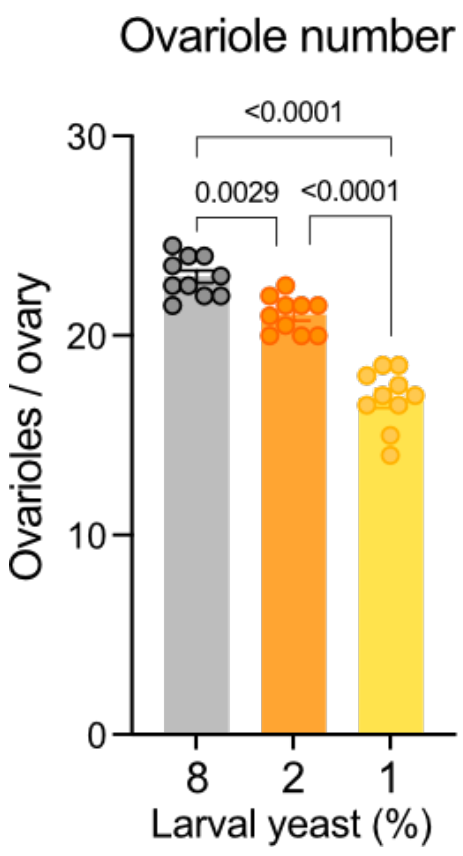

Fig. 1j, Ovariole number in ePR-flies.

Good data on additivity with adult DR. Then the paragraph turns to adults on 0% protein where the benefit of ePR is lost. This observation does not imply, however, 'the outcome of ePR can be variable [via adult dietary conditions] among experimental conditions (refs 6, 18)' (line 116). This explanation obscures the real ambiguities. I can speak for ref 18 (Tu/Tatar 2003): Tu held the 3rd instar with no yeast (rather than 1% or 2%). Tu/Tatar maintain flies with baker's yeast, while Obata uses brewer's yeast. Differences are fine, but readers need to understand specifics and likely causes.
Response 1-5. The original data in Fig. 1n and Extended Data Fig. 1 have been updated by incorporating a full spectrum of adult protein levels (0-12% yeast extract) (Fig. 1q,r). These data together clearly demonstrated that the effect of ePR was maintained and additive with adult yeast restriction under physiological range of protein (4% or 1% yeast extract), The ePR's effect was reversible if adult encounters excessive protein (yeast extract 12%) or none (0%). This was also the case when we manipulated adult amino acid levels by holidic medium (Extended Data Fig. 1c-p).

We performed CoxPH analysis for moderate dietary conditions (4% and 1% yeast extract, 10-100% amino acid conditions). There is no significant interaction between larval protein restriction and adult protein/amino acid intake (Supplementary Table 1). Thus, the effects from two stages are independent and can act additively.

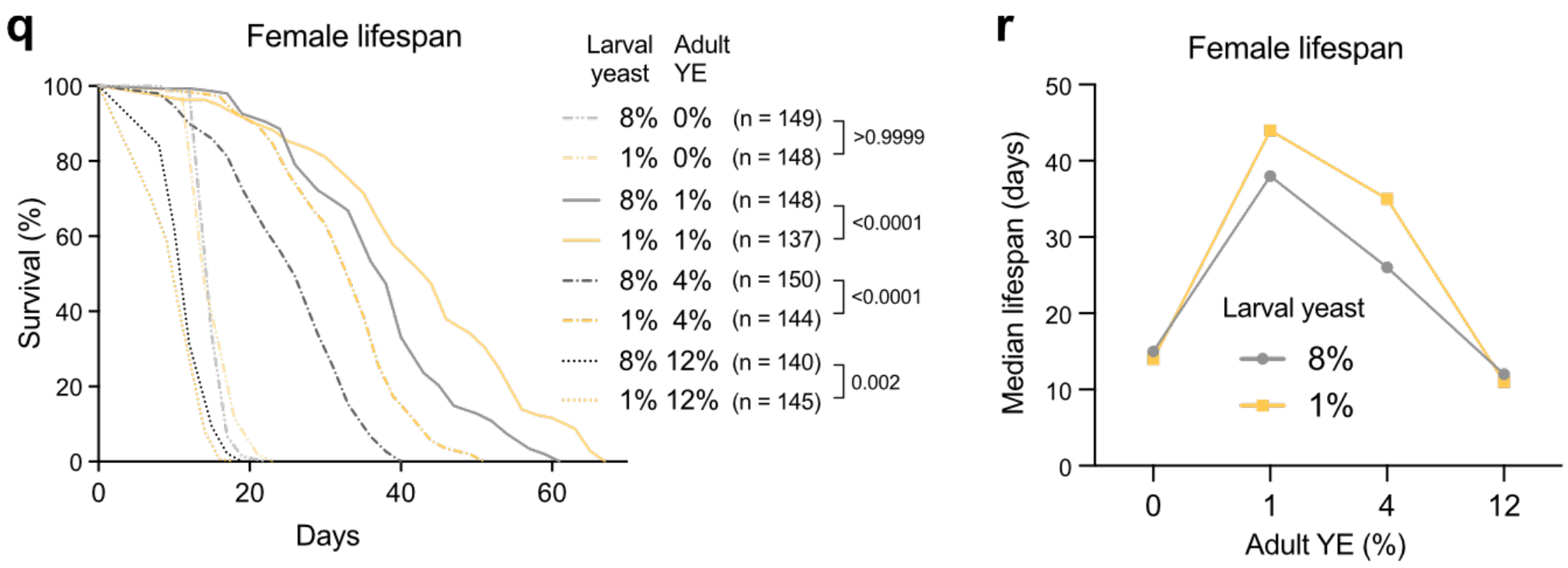

Fig. 1q,r, Lifespan analysis of ePR-flies under varying concentrations of yeast extract in adult.

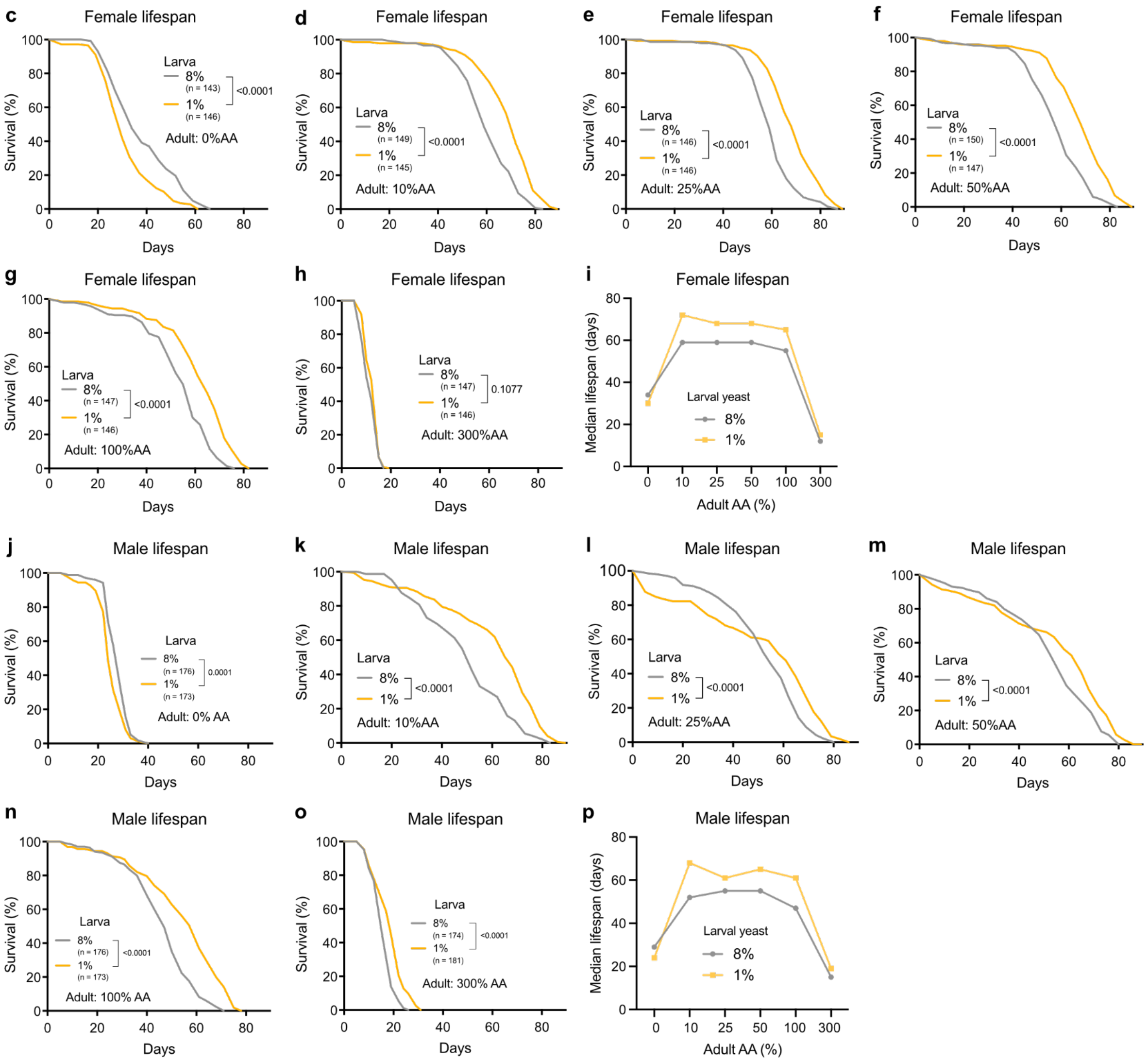

Fig. 1q,r, Lifespan analysis of ePR-flies under varying concentrations of amino acids in adult.

We also experimentally addressed the discrepancy with the Tu/Tatar study. In our experimental system, ePR treatment begins around 68 hr AEL, whereas the Tu/Tatar 2023 study adopted yeast restriction starting at 92-96 hr AEL. When larvae are fed a 0% yeast diet from 68 hr AEL, larvae did not survive, whereas 0% yeast from 94 hr AEL allowed us to obtain some escaper adult flies.

Interestingly, both females and males showed an increase in lifespan upon 0% yeast feeding, even greater than 1% yeast (Extended Data Fig. 1a,b). We assume that the cause of this discrepancy may stem from differences in the standard (control) diet composition. The Tu/Tatar study uses a diet composed of 2% yeast, 10.5% sucrose, 5% cornmeal, 0.7% agar, supplemented with live yeast as a control. The protein/carbohydrates ratio is much lower than our control diet (our control diet: 0.44, Tu/Tatar 2003: 0.09 (supplemented live yeast is not considered though)). Indeed, the ovariole number of the control flies in Tu/Tatar study is about 13.5, while our control flies have about 23. Given that the general range of ovariole number is 12-23 (Green 2nd and Extavour, *Dev Biol* 372, 120, 2012), the control group in Tu/Tatar's experimental system might already exhibit protein restriction-like phenotype. While we added these data into the revised manuscript, we did not discuss the difference given that this discussion is too detailed (and too speculative). However, if you believe this is critical, we will incorporate this discussion in the revised manuscript.

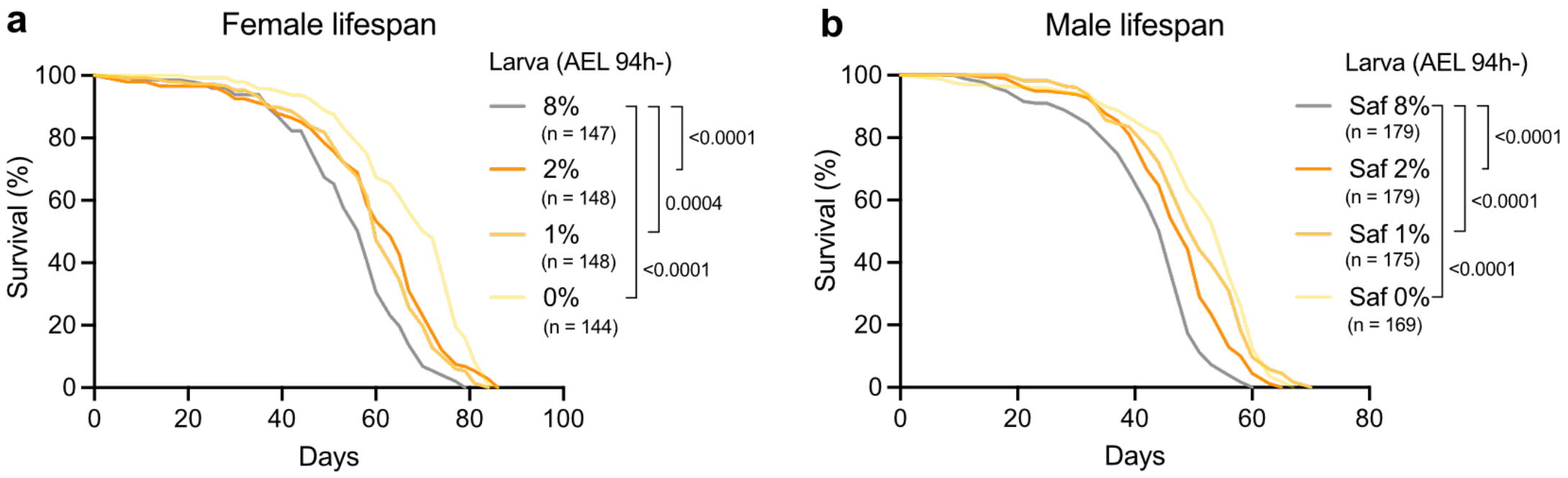

Extended Data Fig. 1a,b Lifespan analysis of flies after ePR at AEL94h.

Dietary composition based on DDCC (https://brodericklab.com/DDCC.php#boxCalc)

Our ePR control (8% yeast) diet:

Nutritional Values

g/liter of diet:		g/100 mL (i.e. percent) of diet:		Ratio:	
539.70	Calories	53.97	Calories	0.44	Protein:Carbohydrates
27.20	Fiber	2.72	Fiber		
60.00	Sugars	6.00	Sugars		
42.03	Protein	4.20	Protein		
4.30	Fat	0.43	Fat		
95.20	Carbohydrates	9.52	Carbohydrates		

Tu/Tatar 2003 control diet:

Nutritional Values

g/liter of diet:		g/100 mL (i.e. percent) of diet:		Ratio:	
650.04	Calories	65.00	Calories	0.09	Protein:Carbohydrates
14.10	Fiber	1.41	Fiber		
104.89	Sugars	10.49	Sugars		
14.22	Protein	1.42	Protein		
2.16	Fat	0.22	Fat		
156.30	Carbohydrates	15.63	Carbohydrates		

Line 120. Tu (2003) established that ePR modulates insulin signaling in the young adult. That paper also sets a stage for some (then unknown) impact of ePR on JH (we would now say, via insulin),

which further connects Kosakamoto to the paper of Kim/Pickering (early adult translation).
Response 1-6. We added the references relevant to discuss the involvement of JH on ePR as shown below. In light of the suggestion, we have performed the qPCR analysis of *Kr-h1*, a readout of JH signalling. ePR did not affect *Kr-h1* expression, suggesting that JH was not altered in this context, although this does not necessarily mean JH would not be involved in ePR. We have added the data to the revised manuscript (Extended Data Fig. 2e).

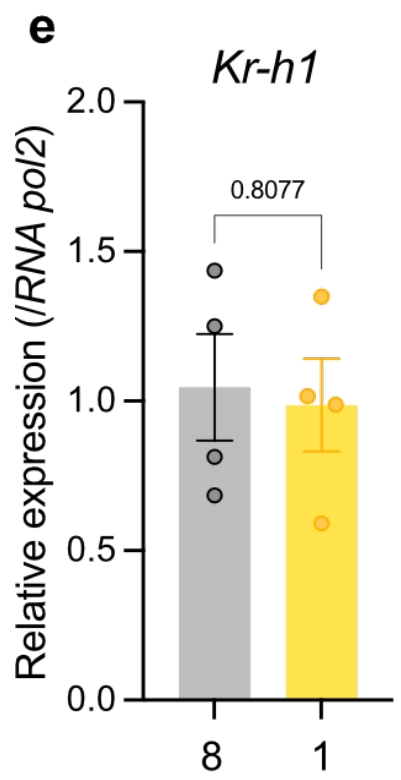

Extended Data Fig. 2e qPCR analysis of Kr-h1 in ePR-flies.

Results section "ePR alters nutrient signalling in early adulthood":
It has been reported that yeast deprivation during the late larval stage decreases both insulin and juvenile hormone (JH) signaling in adult flies[18], both of which can regulate Drosophila lifespan[29].

The paragraph at line 133 does not convince that ePR 'affects nutrient sensing pathways transiently in early adulthood'. Readers will not accept that ATF4 is purely a feature of amino acid sensing. The role of phosphor-eIF2 is not obvious, and it is decoupled from ATF4. I think the paper works better without this paragraph.
Response 1-7. Although ATF4 is regulated by various stressors, it is regarded as one of the amino acid sensing pathways in animals. It is therefore informative whether ATF4 activation alters in early adulthood after ePR, especially given that we have previously shown the ATF4 activation in the larval stage upon yeast restriction (Kosakamoto *et al., Nat Metab*, 2022). Indeed, the accompanying paper by Zhai's group has demonstrated that ATF4 regulates Lsp2 expression. In light of the reviewer's input, we have omitted the p-eIF2 data but kept ATF4 reporter results. We have rephrased the text to avoid overstatement and made this paragraph succinct as shown below. However, if you still believe these data should be removed, we are happy to do so in the final manuscript.

Results section "ePR alters nutrient signalling in early adulthood":
Activating Transcription Factor 4 (ATF4) is another transcription factor activated upon decrease of dietary amino acid[32]. We monitored its activity using the 4E-BP^{intron}-dsRed reporter line[33] and found that ATF4 remained active in the ePR-flies until day 2 of adulthood. However, this activity returned to baseline levels by day 5 (Extended Data Fig. 2m,n).

The isotope study. Please (again) excuse my self-reference, but we did a similar isotope larvae/adult tracing study and found similar conclusions (Min … O'Brien JIP 2006; O'Brien … Tatar 2008 Current Biology. I attach a PDF of 2008 because the paper is hard to find). We made 15N & 13C labeled yeast, and 14N &12C. Larvae and adults were fed alternative isotope forms. We collected somatic tissue and eggs across the aging cohort. From a low concentration of label, we quantified turnover rates and equilibria, all in the context of adult DR. We found many features reported in the current submission. These papers complement, so please educate and guide the reader.

Response 1-8. Thank you very much for informing us about this pioneering work. We have revised the text to discuss these studies as shown below. Together with our newly-added, *in vivo* time-course pSILAC analysis combined with ePR, we have improved the depth of description. Please check our writing below. We are happy to accommodate any further change to the text.

Results section "Larval diet leaves a lasting impact on the adult proteome"
Our phenotypic analysis suggests that early-life protein intake exerts a prolonged impact on early adult physiology and lifespan. Previous efforts to track larval-derived nutrients into adulthood have utilised stable isotope-labelled yeast (^{15}N, ^{13}C vs. ^{14}N, ^{12}C) to quantify the turnover of carbon and nitrogen[28,29]. These analyses, combined with adult dietary restriction, have been used to evaluate nutrient investment strategies in both somatic and reproductive tissues. It has been proposed that larval-derived nitrogen turns over slowly in the ovaries and soma when the adult diet is restricted. However, several fundamental questions remain unanswered. Specifically, it remains unclear how individual larval proteins are carried over into the adult stage, and how animals regulate the transition from utilising larva-derived amino acids to incorporating adult-acquired ones for the synthesis of specific proteins. To date, such proteome-wide protein dynamics have not been directly quantified, leaving a gap in our understanding of how nutritional history is molecularly preserved.

The global enrichment analysis (paragraph starting at line 184) is subjective and tangential. Can these inferences be supported with statistics? Are they essential to the paper's conclusion?
Response 1-9. The analysis is indeed supported by statistics and informative since this is where, for the first time, we described how early-adult flies retained the larva-derived proteome. Please note that we updated *in vivo* pSILAC assay using a more sophisticated mass spectrometry with increased analytical depth (see Methods). We also simplified the methodology. Briefly, for the new experiment, heavy isotope-labeled amino acids were incorporated during the larval stage, while medium isotope-labelled amino acids were used in the adult stage starting from day 0. This eliminates the inclusion of natural (light) amino acids at adult day 0-2, indicating that we trace larval amino acids more precisely and accurately. Instead, now we have sequentially sampled at day 3 and day 6 to show how labelled proteome shifts in the first week of adulthood. We performed the enrichment analysis again with the new data, demonstrating that the GO terms for Heavy-rich proteins based on abundance yielded the results shown in the figure below, revealing a significant enrichment in cytoplasmic translation (Fig. 2c,e, Extended Data Fig. 3b).

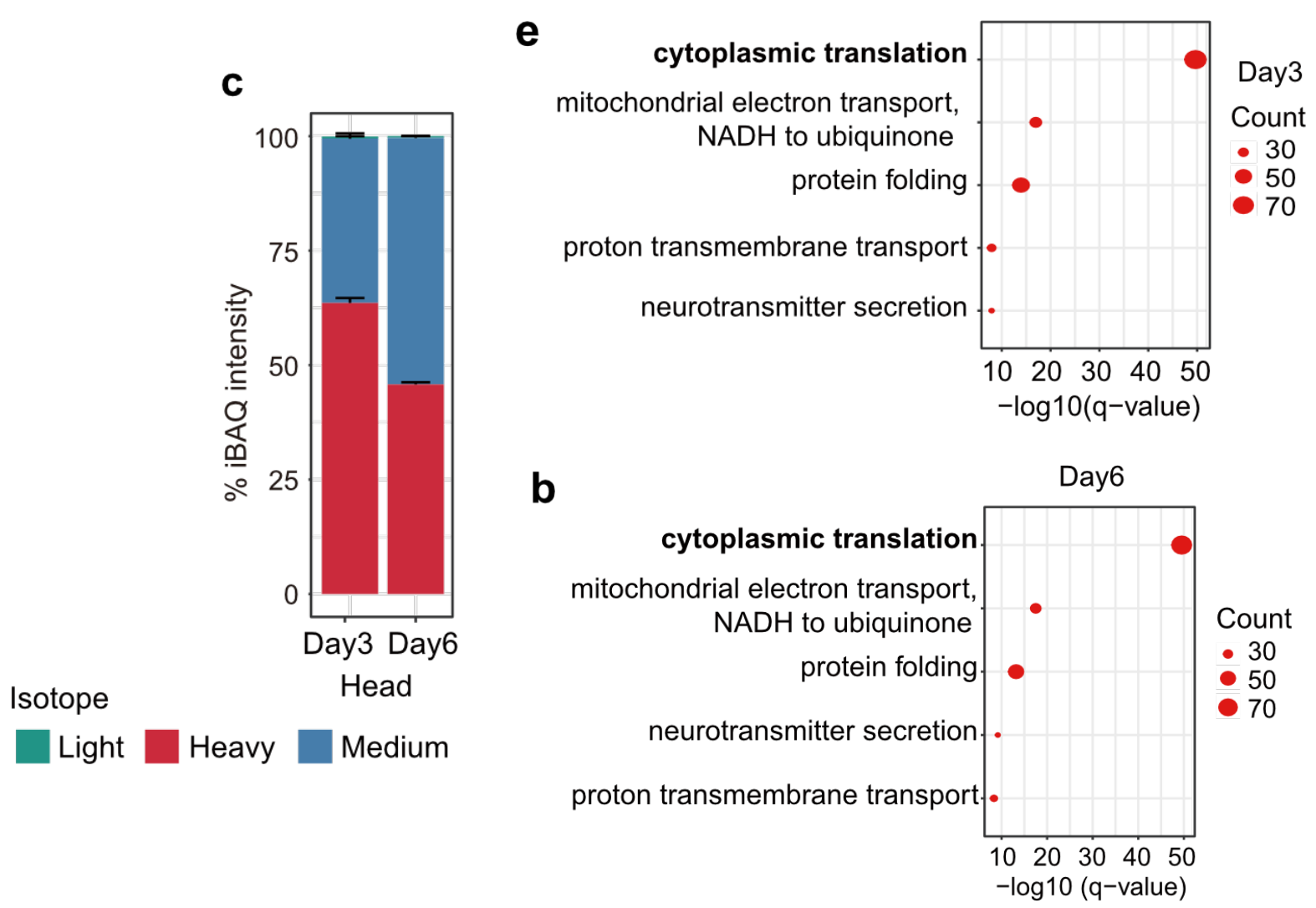

Fig. 2c,e, Extended Data Fig. 3b, Updated *in vivo* pSILAC analysis in the standard diet.

Proteomics. ePR alters the quantity and quality of amino acids in young adults, but ePR was not studied with the labeled samples. It is a conjecture that the altered profiles arise from carry-over. We can't distinguish effects from ePR relative to adult feeding.

Response 1-10. Thank you very much for this transformative suggestion. We have now performed *in vivo* pSILAC analysis on flies with ePR to directly test how ePR modulates the proteome turnover. In this experiment, larvae were fed yeast containing light amino acids (either 8% or 1% yeast diet) during the larval stage and the adult flies were fed a holidic medium containing heavy-labelled amino acids (from day 0) (Fig. 2i). We found that ePR-flies (1% yeast) showed lower "heavy" fraction on day 1, but from day 2 onwards, the heavy:light ratio became nearly identical to that of control (Fig. 2j). These data suggested that global protein synthesis using adult-derived amino acids are only transiently decreased at day1. However, we could not deny the possibility that ePR-flies have less feeding at day 1. At least from day 2, the global reliance on amino acids derived from the adult stage did not change significantly with ePR. This is rather ideal to directly compare how ePR affects the quality of proteome turnover at this stage. When we looked at the ribosomal proteins (RPs) and histones, as expected, both cytosolic RPs (cytoRPs) and mitochondrial RPs (mitoRPs) were found to be gradually replaced by heavy-labelled proteins (adult-derived amino acids) (Fig. 2k–m). cytoRPs have faster replacement than mitoRPs, and at day 6, half of the cytoRPs were now heavy-labelled. Histones have a much slower turnover rate. In ePR-flies, light-labelled cytoRPs were reduced during the first three days. Heavy-labelled cytoRPs were only transiently decreased at day 1 by ePR but became comparable at day 2. The decrease of heavy-labelling at day 1 was not specific to cytoRPs, further supporting that protein synthesis and/or feeding was very low in the day 1 ePR-flies. In contrast, day 2-6 samples had comparable heavy-labelling in all cytoRPs, mitoRPs, and histones. Together, these data suggested that ePR influenced light-labelled (larva-derived) cytoRPs selectively and did not have an effect on synthesis of RPs from adult-derived amino acids. These data have been added to the revised manuscript.

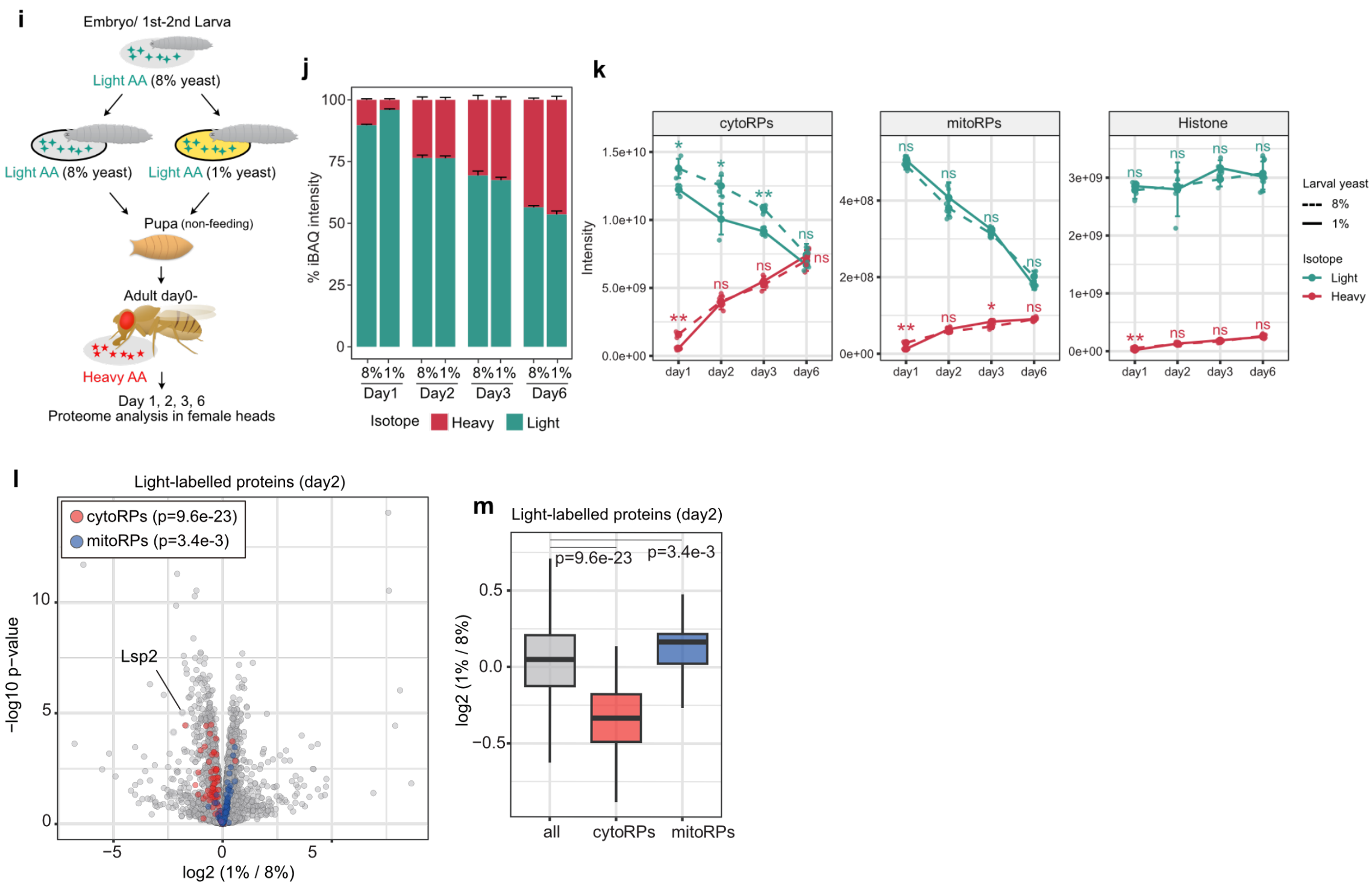

Fig. 2i-m, time-course *in vivo* pSILAC analysis of ePR-flies.

Results section "ePR decreases ribosomal proteins and decreases translation in early adulthood"

To determine if ePR modulates the adult proteome turnover, we performed a time-course in vivo pSILAC assay (Fig. 2i, Extended Data Fig. 4h-k, and Supplementary Table 2). In this experiment, larvae were reared on either an 8% or 1% yeast diet (containing light amino acids), and the newly-eclosed adults were switched to a synthetic diet containing heavy amino acids (+8 Lys/+10 Arg). We prepared samples at days 1, 2, 3, and 6 to monitor the proteome turnover over time (Fig. 2i). Consistent with the results in Fig. 2c, the heavy labelled proteomes were enriched over time and reached about half at day 6 (Fig. 2j). At day 1, ePR-flies exhibited a lower heavy fraction; however, from day 2 onwards, the heavy/light ratio became nearly identical to controls (Fig. 2j). The reduced incorporation of adult-derived (heavy) amino acids at day 1 in ePR-flies suggests not only reduced protein synthesis but also decreased feeding rates. Nevertheless, after day 2, the relative reliance on larva- versus adult-derived amino acids for the global proteome did not differ significantly between groups (Fig. 2j).

We then specifically examined the turnover of ribosomal proteins (RPs). In control flies, both cytoRPs and mitoRPs were gradually replaced by heavy-labelled (adult-derived) proteins, with cytoRPs showing faster replacement than mitoRPs (Fig. 2k). In ePR-flies, however, light-labelled (larva-derived) cytoRPs were significantly lower during the first three days (Fig. 2k). From the volcano plot from the day 2 samples, we confirmed that larva-derived cytoRPs are lower in ePR-flies (Fig. 2l,m). Notably, while heavy-labelled (adult-derived) cytoRPs were also transiently decreased at day 1, they became comparable to controls by day 2 (Fig. 2k). The transient decrease at day 1 in heavy-labelled proteins was commonly observed across cytoRPs, mitoRPs and histones, reflecting a general reduction in protein synthesis or feeding at the onset of adulthood by ePR (Fig. 2j,k). Together, these data suggest that ePR selectively reduces the pool of larval carry-over cytoRPs without affecting the synthesis of new RPs from adult-acquired amino acids.

Thanks to this great suggestion, we are now able to analyse how much adult Lsp2 is derived from larval amino acids and how it turns over (also related to response 1-15). In the control flies, Lsp2 protein at day 1 is predominantly light-labelled, which is gradually replaced by heavy-labelled Lsp2. At day 6, heavy-labelled Lsp2 are more than light-labelled Lsp2. As we expected, light-labeled Lsp2 is significantly lower by ePR for all time points (days 1, 2, 3, and 6). In ePR-flies (1% yeast), heavy-labeled Lsp2 is also decreased during the first three days, which is consistent with the decreased Lsp2 expression in ePR-flies. We incorporated these data in the revised manuscript (Fig. 3k).

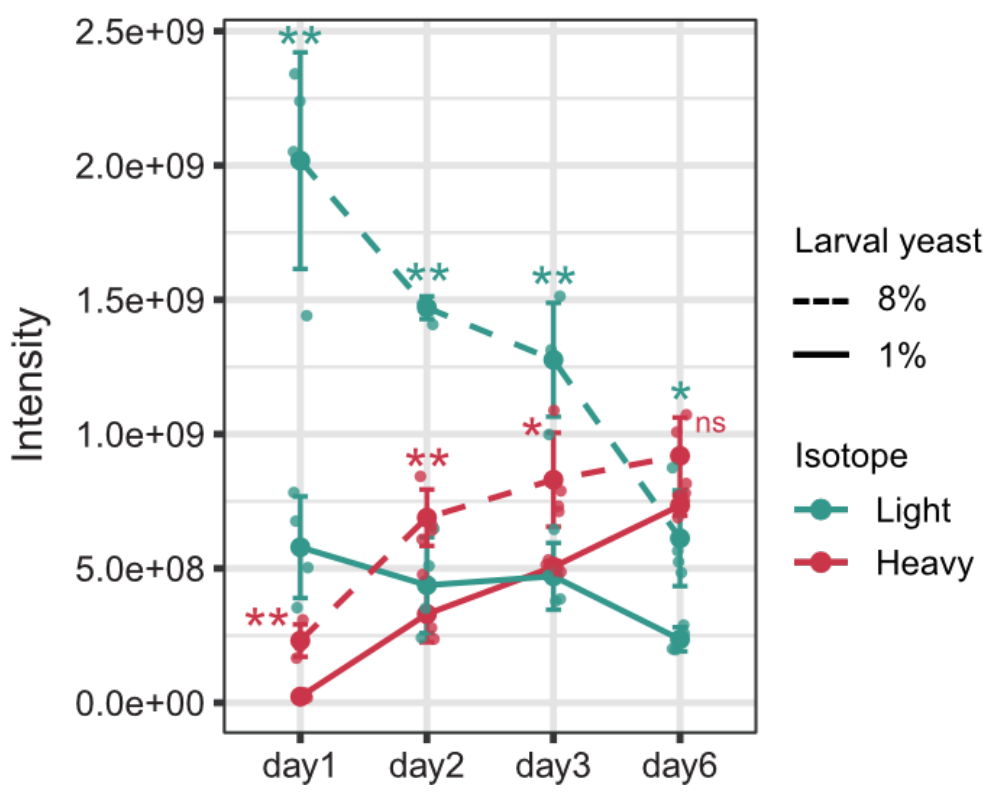

Fig. 3k, Lsp2 turnover revealed by time-course *in vivo* pSILAC analysis of ePR-flies.

Results section "ePR decreases both carry-over and synthesis of Lsp2 in early adulthood"

The combination of in vivo pSILAC and ePR further elucidated the Lsp2 protein turnover in early adulthood. In control flies, Lsp2 at day 1 is predominantly light-labelled (larva-derived), which is gradually replaced by heavy-labelled (adult-synthesised) Lsp2 over time (Fig. 3k, Supplementary Table 2). By day 6, the heavy-labelled fraction exceeds the light-labelled pool. As expected, light-labelled Lsp2 levels were significantly lower in ePR-flies across all time points (days 1, 2, 3, and 6), confirming a reduced carry-over of larval protein reserves. Notably, heavy-labeled Lsp2 was also significantly

decreased in ePR-flies during the first three days of adulthood (Fig. 3k), indicating that ePR suppresses both the legacy of larval Lsp2 and its de novo synthesis in early adulthood.

The protein profiles in young adults suggest there is reduced translation capacity following ePR. Kim/Pickering (Nature Comm 2023) recently reported young adults have a 'spike' in translation. Suppressing the spike increased life expectancy. Kosakamoto now sees high translation in young adults that is blunted by ePR. They propose this contributes to slow aging. Kosakamoto activates mTORC1 in ePR treated flies to test this idea, and this reduces their longevity. However, Kosakamoto increases mTORC1 across all adult ages, not just in the phase where ePR is thought to inhibit translation. Most would say that elevating mTORC1 across adulthood is bad for survival. A better design requires a time-limited rescue (as in Kim/Pickering), and analysis of age-specific mortality following the manipulation period.
Response 1-11. As suggested, we restricted the activation of the mTORC1 pathway (by *Rheb* overexpression) only during the first week of adulthood. We found that ePR failed to extend lifespan when *Rheb* was overexpressed in the first week (Extended Data Fig. 5a,b). However, we self-criticised that forced mTORC1 activation would impact many biological processes other than translation. Although the Kim/Pickering paper clearly suggested the connection between decreased early-adult translation and lifespan, we cannot fully conclude the causal link, due to the lack of genetic tools to specifically restore the decreased cytoRPs abundance and translational suppression. We added this explanation in the revised text honestly and therefore amended the manuscript title.

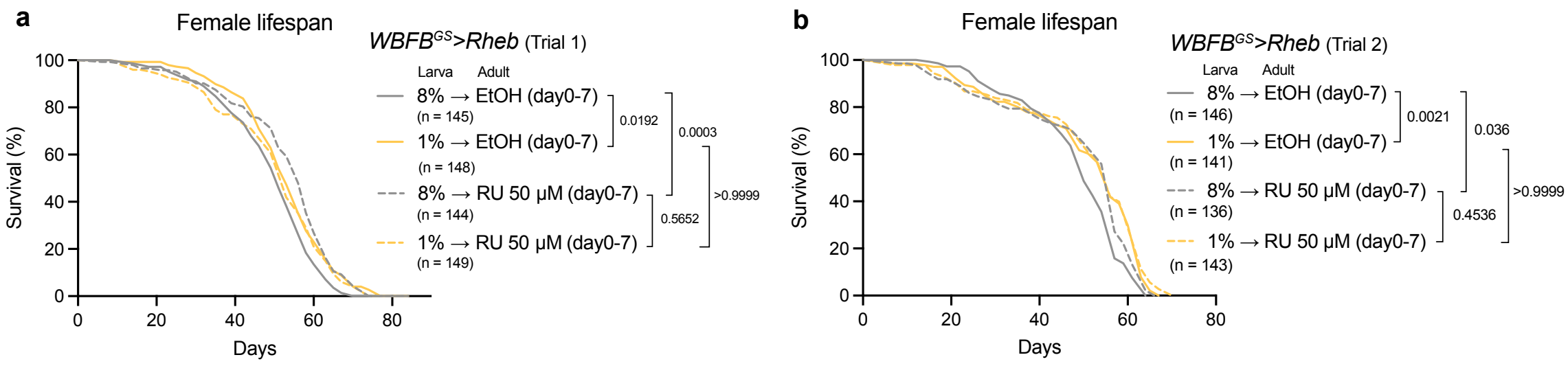

Extended Data Fig. 5a,b, Lifespan analysis of ePR flies with *Rheb* overexpression at day0-7.

Results section "ePR decreases ribosomal proteins and decreases translation in early adulthood":
To ask the functional significance of this translation suppression for the ePR-mediated lifespan extension, we attempted to enhance translation by overexpressing Rheb, a potent mTORC1 activator, in the adult fat body during the first week of adulthood. We found that ePR failed to extend lifespan when Rheb was overexpressed in the first week (Extended Data Fig. 5a,b), implying that lifespan extension by ePR is driven, at least partly, by suppressed translation. We acknowledge, however, that mTORC1 signaling does not exclusively stimulate protein synthesis but also regulates various processes. It is challenging to definitively prove the causal relationship between, cytoRPs, lower protein synthesis, and lifespan extension in ePR-flies without tools to specifically restore the entire cytoRP pool and to boost translation specifically. Nevertheless, given that previous studies connecting early adult translation and organismal lifespan[34], they are likely to be mechanistically interconnected.

The research sequence at line 241-243 seems strange. Kosakamoto already conducted the proteomics to identify the 'molecules', so why bother with transcriptome analysis?
Response 1-12. Thank you for raising this issue. Indeed, we came to Lsp2 from a lot of different aspects. In the newly added experiment of *in vivo* pSILAC with ePR, we found that larval amino acid-derived Lsp2 were decreased in ePR (Fig. 2l). We have revised the result section to improve the logical flow.

l

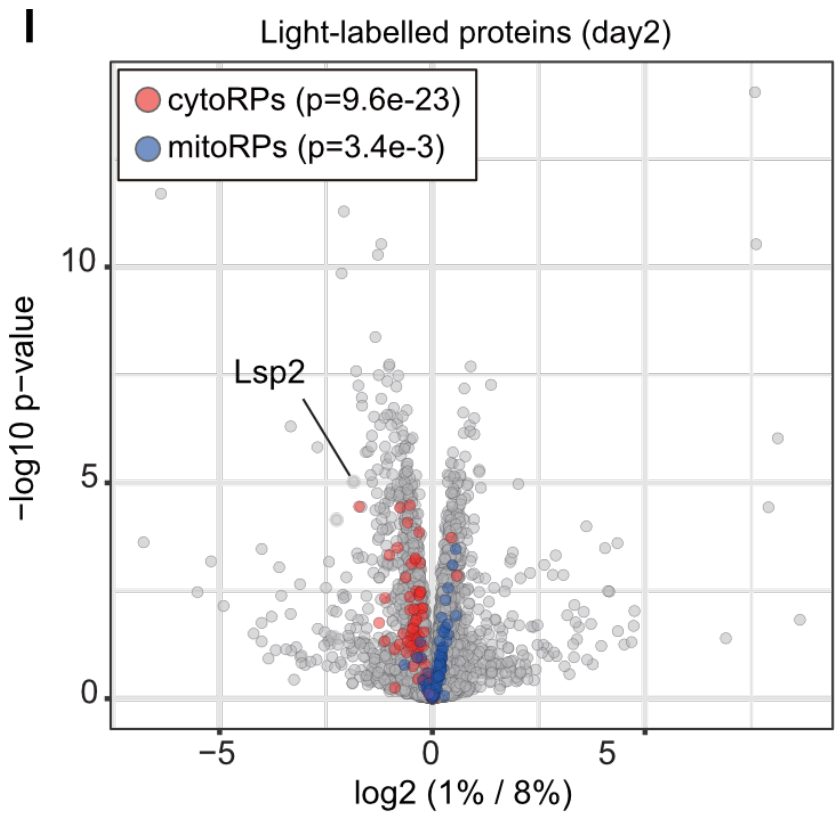

Fig. 2l, Volcano plot from time-course *in vivo* pSILAC analysis of ePR flies.

Lsp2 mRNA is selected as the focal DEG, but its expression in the adult head is not 'unexpected' (line 265). It was documented in 1990 (Benes et al.) and verified in expression studies (see FlyBase). Kosakamoto mention Benes in the discussion. To justify the Lsp2 focus at this point, directly talk about the storage protein data of Kim/Pickering (Nature Comm 2023). They identified Lsp2 as a dynamic factor in the early soluble fraction. Use this to set up the appropriate question: where does the adult LSP2 come from? The LSP2 may arise from adult translation because adults have high Lsp2 mRNA (PCR) and protein, whereas adults from ePR have lower Lsp2 mRNA and protein. (And … why Lsp2 relative to all the other storage proteins?)
Response 1-13. We have revised the paragraph based on what the reviewer suggested as shown below. Please also see our Response 1-12.

Results section "ePR decreases both carry-over and synthesis of Lsp2 in early adulthood"
Our data indicate that ePR significantly reduces cytoRP levels, which correlates with decreased translation in early adulthood. We hypothesised that protein(s) which are carried over from the larval stage and are affected by larval nutrition could mediate the nutritional memory effect of ePR. From this criteria, we identified the storage protein Larval serum protein 2 (Lsp2) in the pSILAC assay (Fig. 2l). LSPs are storage proteins characteristic of holometabolous insects, which face nutrient restriction during non-feeding pupal development[41,42]. Only recently has the detailed functional study of these storage proteins been done, revealing their specific requirement for late-developed adult tissues[43]. These proteins are primarily synthesised in the larval fat body, secreted into the haemolymph, and subsequently taken up by the fat body during pupal development. Lsp2 and other storage proteins are highly expressed in late larvae and early pupae, and their expression typically declines sharply toward the end of metamorphosis[43]. Although long considered mere transient reservoirs for development, Lsp2 is known to persist and be expressed in adult flies[44]. Indeed, Lsp2 is one of the major soluble proteins in the early adult hemolymph[40]. However, the physiological role of adult Lsp2 remains largely unexplored, except suggestions that storage proteins support adult fecundity[45,46].

I am confused by the wording in lines 279-280 and 281-283. If the idea is that ePR represses adult Lsp2 mRNA, why discuss wandering larvae and pupae? I am not convinced that knockdown of Lsp2 (or Fbp) across all larval stages suggests a 'feed forward mechanism to regulate storage protein expression'. Might knockdown block larval fat body development, which carries across to the adult. Not 'nutritional memory' (per ePR) but stunted tissue development?
Response 1-14. Our data (Fig. 3h) showed that decrease in *Lsps* by RNAi in the larvae led to decrease in *Lsp2* expression in the adult, suggesting that Lsps protein regulates *Lsp2* gene expression as a feedforward mechanism, which was also suggested in the accompanying Wang et al.

To eliminate the effects of developmental defects in the fat body tissue, we quantified 1) fat body size in wandering larvae and 2) fat body-expressed GFP in day-2 adult flies. *Lsp1α* or *Fbp2* knockdown did not impact on these parameters (Extended Data Fig. 6g–j), suggesting that fat body

development occurs normally. These data have been added to the revised manuscript as shown below.

As discussed in Response 1-16, we have highlighted the difference of larval vs early adult *Lsp2*-RNAi, based on the data obtained during the revision. We hope the revision clarified our conclusion.

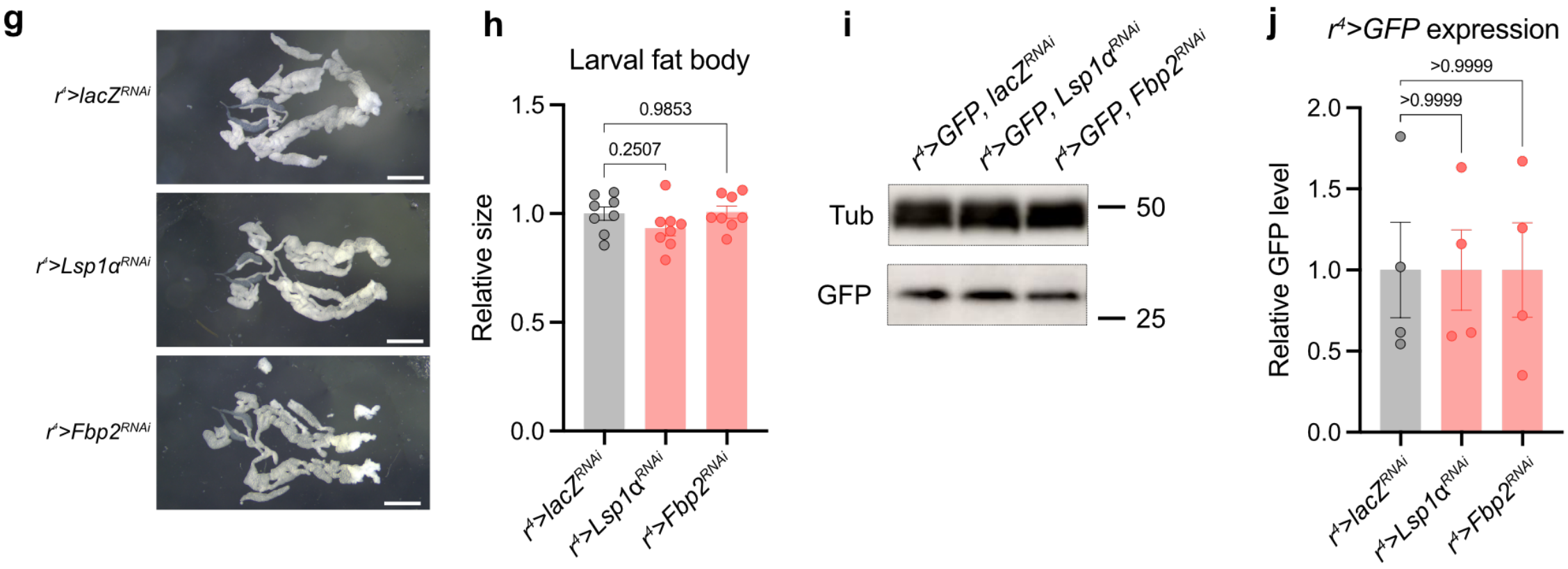

Extended Data Fig. 6g–j, Fat body analysis in larvae and in adults with loss of function of storage proteins.

Results section "ePR decreases both carry-over and synthesis of Lsp2 in early adulthood"

To test whether larval Lsp2 levels directly influence subsequent expression in adulthood, we transiently knocked down Lsp2 in the larval fat body using the Cg-Gal4 driver combined with Gal80ts. Interestingly, this larval-specific knockdown was sufficient to reduce Lsp2 mRNA expression in early adults (Fig. 3h,i). Interestingly, when we knocked down genes encoding other major storage proteins, including Larval serum protein 1α (Lsp1α) and Fat body protein 2 (Fbp2), using RNAi lines previously verified for their specificity[43], Lsp2 expression in adult flies were significantly decreased (Fig. 3j). This manipulation did not cause apparent defects in fat body development (Extended Data Fig. 6e-h). These results suggest the existence of a feed-forward regulatory mechanism that maintains storage protein expression, a concept also demonstrated in the accompanying study by Wang et al[47].

The hypothesis of line 297-308. The premise is weak. Turn-over of LSP occurs in the adult, as it does for many larval-derived proteins (except perhaps those in the cuticle). See O'Brien (2008) who quantified nitrogen-molecule turnover as a function of adult DR. Kosakamoto could benefit from pSILAC of adults derived from the three larval ePR conditions. This would quantify how much LSP derives from larvae and from adult feeding and estimate the turnover in the adult. Those data would test if the 'observed reduction' (via ePR) is caused by less carryover or reduced production in the adult (need not be via 'suppressed transcription'). One caveat concerns the issue of recycled amino acids.

Response 1-15. Please refer to our Response 1-10 about pSILAC with ePR. We incorporated the O'Brien et al., Curr Biol, 2008 and have revised the manuscript accordingly.

The development-limited Lsp2 RNAi data are central to the paper. Kosakamoto buries them behind the continuous knockdown (larva through adulthood) data. The continuous data confound what they want to understand about ePR with the impacts from Lsp2 RNAi in the adult.

The project needs fuller data from the larval-only knockdown. How does this effect the omic measures, interact with ePR (viz longevity), affect males? How effective is the larval RNAi to reduce the adult Lsp2 protein, and over what time course? Address a genetic background issue: gal80 controls time of expression but compares different genetic backgrounds. Even with some backcrossing, there is much room for second site effects (especially in survival data). I suggest

Kosakamoto measures phenotypes of both genotypes (lacz and RNAi) from animals continuously at the restrictive temperature. They should look the same. I recommend they replicate all survival trials to demonstrate the result is reproducible (one 'significant' trial is weak sauce). Include males and give data in the main figure.

Response 1-16. As the reviewer pointed out, we performed additional experiments for larva-specific *Lsp2* knockdown to the maximum extent allowed by our resources. In experiments using *tub-Gal80*^{ts}, both *UAS-lacZ-RNAi* and *UAS-Lsp2-RNAi* lines were backcrossed eight times into *w*^{CS}. While mentioned in the Methods section, we added this clarification to the legend for greater clarity. We also included the lifespan curves from repeated experiments in the supplement (Extended Data Fig. 10e). Proteome analysis of the early adults after developmental knockdown of *Lsp2* was also added (Fig. 4i,j), which essentially suggested the same conclusion of conventional *Lsp2*-RNAi.

Furthermore, since background effects still can exist even after backcrossing, we attempted knockdown using the GeneSwitch system. Since no GeneSwitch driver line suitable for larval fat bodies existed, we created *pGnmt-GeneSwitch* (*pGnmt*^{GS}) using the promoter sequence of the metabolic enzyme gene *Gnmt*, which is highly expressed in fat bodies, and used this to perform knockdown only during the larval stage. Crossing with *UAS-GFP* confirmed that *pGnmt*^{GS} expression is almost exclusively confined to adipose tissue and is RU-inducible (Extended Data Fig. 10f.g). As expected, knockdown of *Lsp2* in larvae using *pGnmt*^{GS} suppressed protein translation and extended lifespan in both males and females albeit its effect is mild (Extended Data Fig. 10h–k). It is possible that, since wandering larvae were not fed on RU-containing food, the knockdown efficiency might not be effective enough to phenocopy *Lsp2* knockdown through the developmental stage. We have added these data in the revised manuscript.

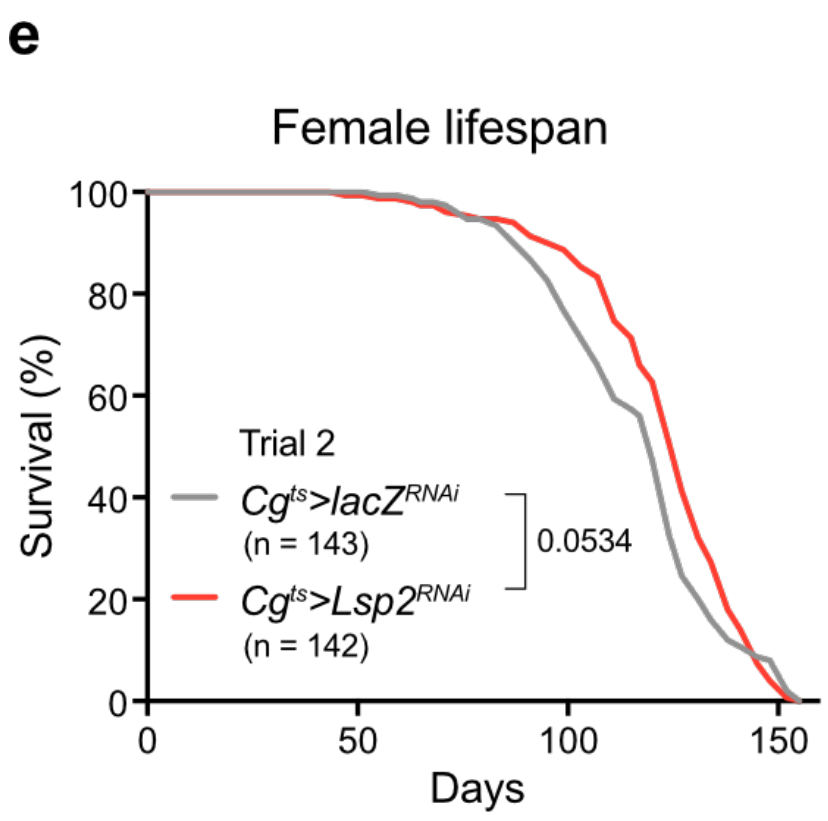

Extended Data Fig. 10e. Lifespan analysis of larva-specific Lsp2 RNAi using *Cg*^{ts} (2nd trial).

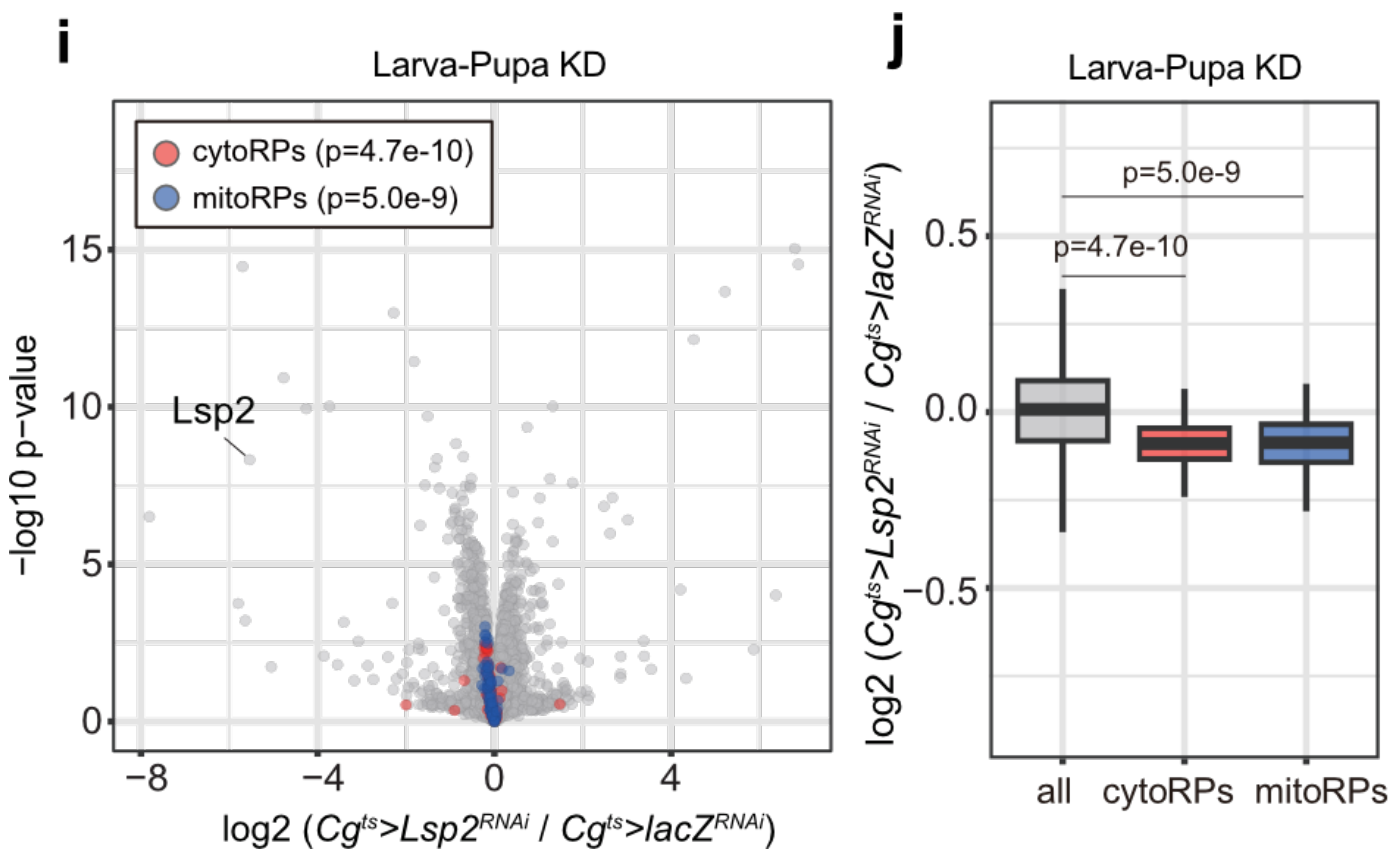

Fig. 4i,j, Proteome analysis of early-life specific Lsp2-RNAi using *Cg*^{ts}

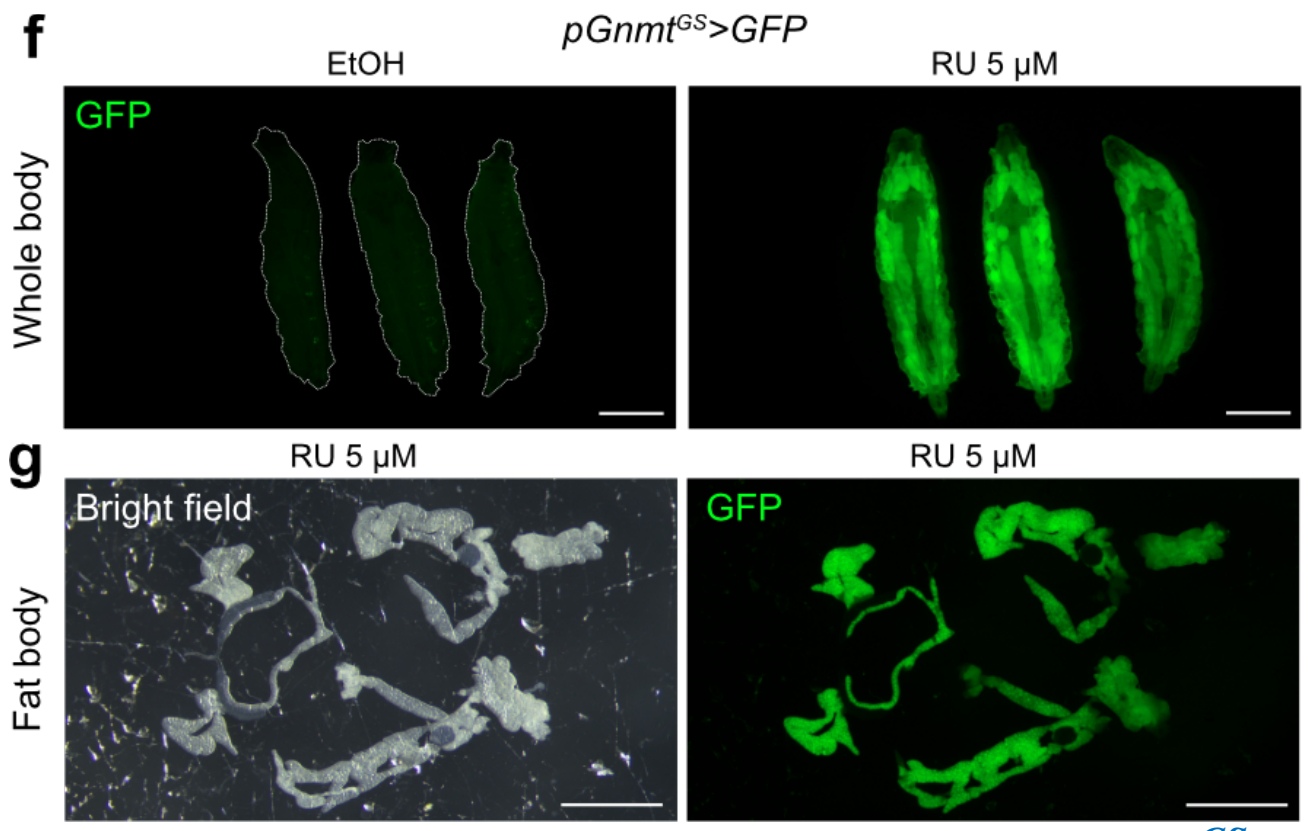

Extended Data Fig. 10f.g Visualization of *pGNMT*^{GS} driver activity.

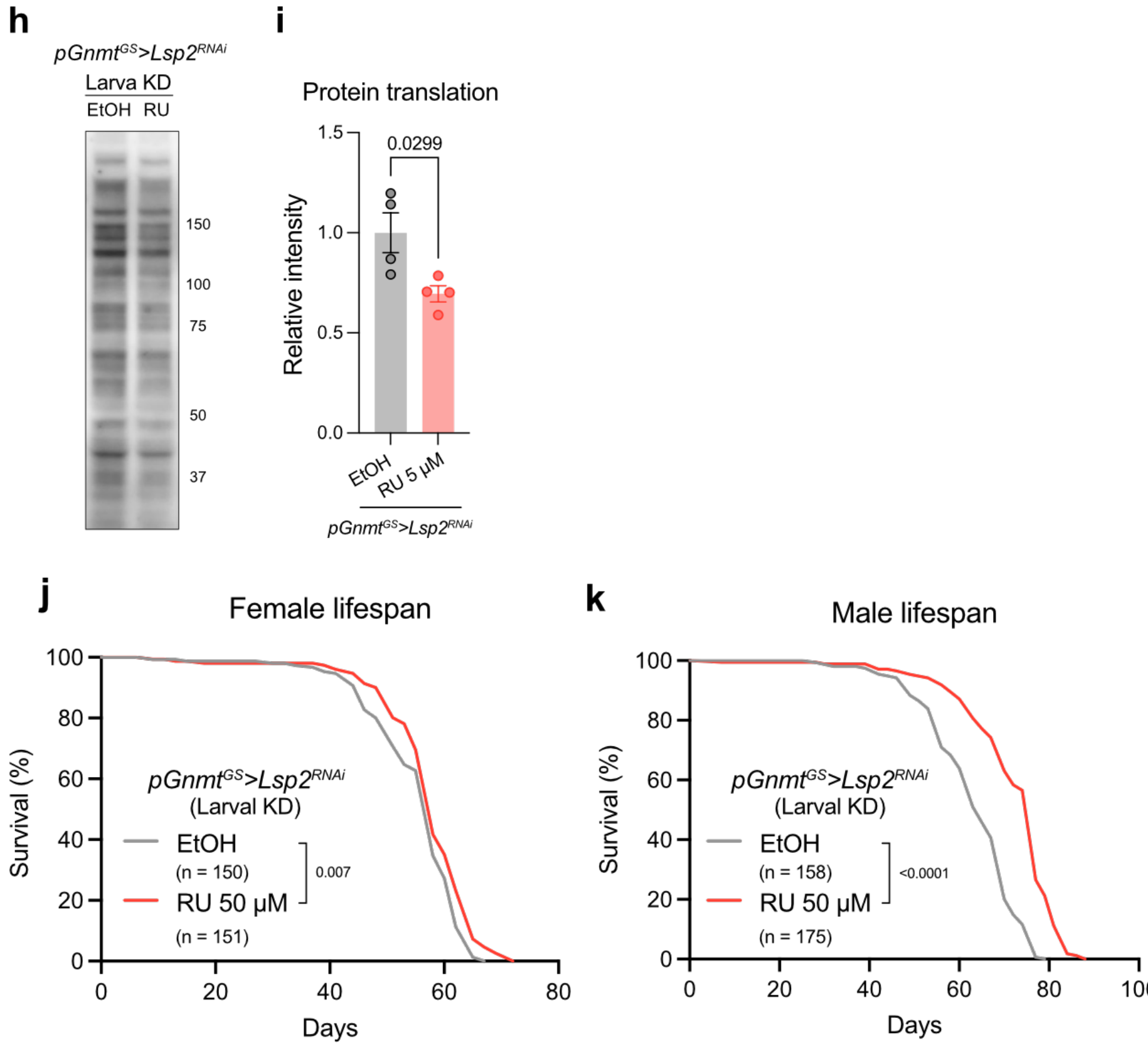

Extended Data Fig. 10h–k, Translation assay and lifespan analysis of larval *Lsp2*-RNAi using *pGNMT*^{GS}.

Results section "Loss of Lsp2 suppresses protein translation and extends lifespan in adulthood"

Although Lsp2-RNAi and ePR share the mechanism for lifespan extension, our data did not demonstrate that early-life Lsp2 regulates lifespan. Using a temperature-sensitive Gal80 system, we tested whether the decrease in larva-derived Lsp2 was sufficient for reduced translation and lifespan extension. In Cg^{ts}>Lsp2-RNAi flies, we found that both cytoRP levels and translation activity were reduced compared to control flies (Fig. 4i–l, Extended Data Fig. 10a–d, and Supplementary Table 3), suggesting the role of larva-derived Lsp2 in regulating early adult translation. Accordingly, Lsp2 knockdown restricted to the larval stage (Cg^{ts}>Lsp2-RNAi) was sufficient to extend female lifespan (Fig. 4m, Extended Data Fig. 10e). To exclude potential genetic background issues, we generated the pGnmt-GeneSwitch (pGnmt^{GS}) fly line by using the promoter sequence of the metabolic gene Gnmt, which is

highly expressed in fat bodies[48]. Crossing this line with UAS-GFP confirmed that pGnmt^{GS} expression is almost entirely confined to adipose tissue and is RU486-inducible (Extended Data Fig. 10f,g). As expected, the knockdown of Lsp2 in larvae using pGnmt^{GS} decreased translation in female flies (Extended Data Fig. 10h,i) and extended the lifespan of both males and females (Extended Data Fig. 10j,k), albeit the effect was mild. It is possible that, since wandering larvae were not fed RU-containing food, the phenotype was not as strong as that achieved by Lsp2 knockdown throughout the larval to pupal stages. Nevertheless, these results prove that decreasing Lsp2 levels only in the larval stage suppresses protein translation and extends lifespan. However, considering that larval Lsp2 expression regulates adult Lsp2 expression through a feed-forward response (Fig. 3h–j), it remains possible that the Lsp2-RNAi in larvae decrease Lsp2 protein in early adulthood and exerts translational control.

The tyrosine result does not demonstrate Lsp2 as the cause, or visa versa. I suggest controls to show that lifespan is not increased when you reduce amino acids besides Tyr, Phe or His (Lsp2 specialties). Line 352-353 connects the exome observations to aging through some previous work (refs 22, 27). What is meant by 'highly sensitive to Tyr intake'? Explicitly discuss results from Parkhitko (eLIFE 2020), which established the role of Tyr in the control of aging, although paradoxically in the opposite direction.

Response 1-17. We have analysed the flies with dietary restriction of individual amino acids in the larval stage. Since the complete restriction of essential amino acids prevents adult emergence and the synthetic diet used previously for Tyr deprivation produces semi-lethality, we utilised the larva-optimised HolFast diet to manipulate essential amino acids, such as Phe, His, and isoleucine (Ile). We confirmed that Tyr deprivation also extends lifespan in this experimental diet (Fig. 5g,h). Restriction of Lsp2-enriched amino acids, such as Phe and His, increased lifespan in both males and females (Fig. 5g,h). In contrast, restriction of Ile, which is less abundant in Lsp2, did not extend lifespan (Fig. 5g,h). Consistently, we found that the GFP fluorescence of Lsp2-muGFP decreased upon restriction of Phe or His, but not Ile (Fig. 5i). Interestingly, Ile restriction induced a stronger developmental delay than Phe or His restriction, suggesting that Lsp2 can act as a buffer to support larval development under nutritional stress by providing stored amino acids (Fig. 5j). We have added these data into the revised manuscript as shown below.

Regarding Parkhitko's paper, it particularly focuses on the late life change in tyrosine metabolism. We have already discussed the discrepancy in our previous study (Kosakamoto *et al., Sci Adv*, 2024) and would not repeat the discussion here in this manuscript.

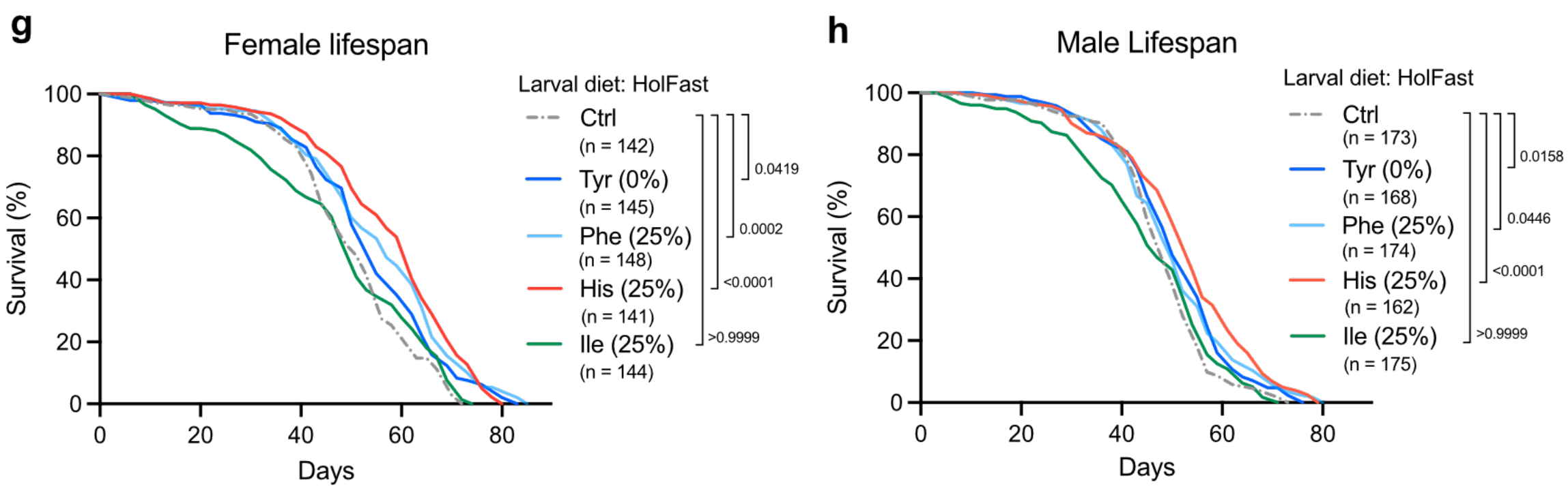

Fig. 5g,h, Lifespan analysis of the flies with larva-specific single amino acid restrictions.

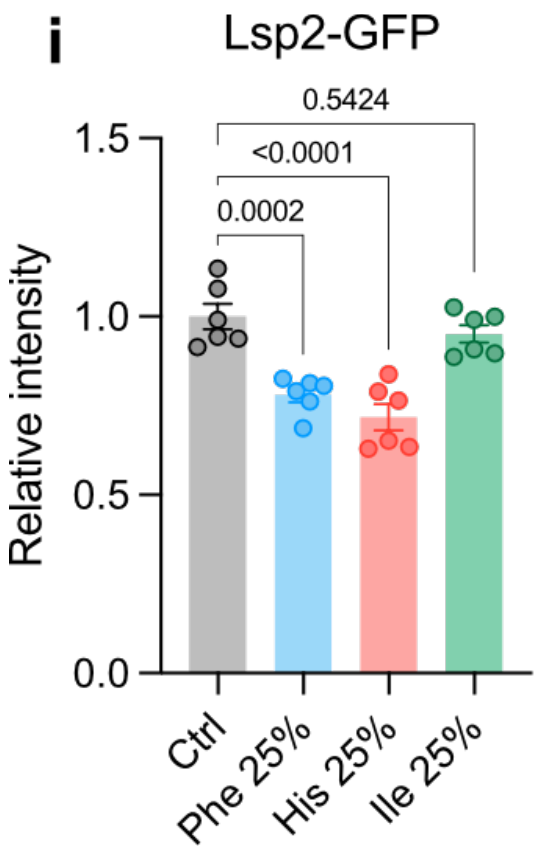

Fig. 5i, Lsp2-GFP quantification of the flies with larva-specific single amino acid restrictions.

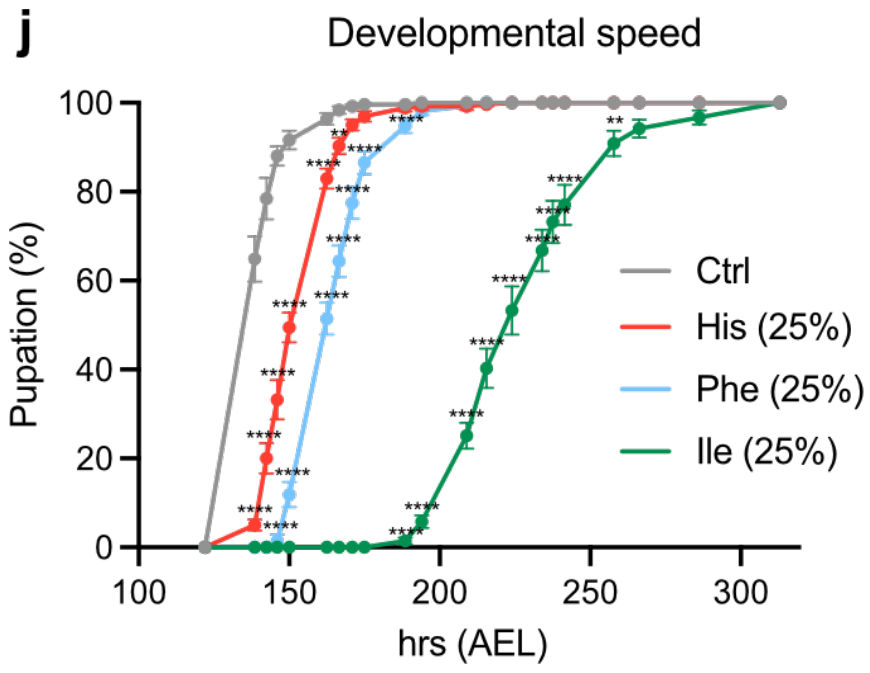

Fig. 5j, Developmental speed under single amino acid restrictions.

Results section "Single amino acid manipulation in the larval stage extends lifespan"

To test amino acid specificity, we restricted other amino acids. Since the complete restriction of essential amino acids prevents adult emergence and the synthetic diet used above for Tyr deprivation produces semi-lethality, we utilised the larva-optimised HolFast diet to decrease essential amino acids, such as Phe, His, and isoleucine (Ile), to 25%. We confirmed that Tyr deprivation also extends lifespan in this experimental diet (Fig. 5g,h). Restriction of Lsp2-enriched amino acids, such as Phe and His, increased lifespan in both males and females (Fig. 5g,h). In contrast, restriction of Ile, which is less abundant in Lsp2, did not extend lifespan (Fig. 5g,h). Consistently, we found that the GFP fluorescence of Lsp2-muGFP decreased upon restriction of Phe or His, but not Ile (Fig. 5i). Interestingly, Ile restriction induced a stronger developmental delay than Phe or His restriction, suggesting that Lsp2 can act as a buffer to support larval development under nutritional stress by providing stored amino acids (Fig. 5j). Taken together, these data suggest that the larval intake of dietary amino acids enriched in Lsp2 regulates the abundance of Lsp2 proteins in early life stages, which in turn modulates adult translational capacity and lifespan (Fig. 5k).

Discussion

In my opinion, the work does not yet establish its conclusion of line 364-366 because the referenced results were based on life-long Lsp2 RNAi, not larval-limited.

I am not sure Kosakamoto's data support the conclusion asserted on lines 370-372. Kosakamoto associates a storage protein with features of translation control. While knockdown of Lsp2 in the larvae reduces total protein translation in the young adult, the work does not prove the change in translation is required to slow aging. The design with TORC1 is not convincing. What I do see is that reducing Lsp2 in the larval stage (by RNAi) can extend lifespan in females.

Indirect inferences are used for big conclusions of the discussion. I enjoyed these ideas, especially in the last several paragraphs. They are broad and exciting, but I am not convinced they are supported by the offered data.

Response 1-18. We appreciate Dr. Tatar for this and above all critical comments. Based on the new results we obtained during revision, we have revised the Discussion section. We avoid overinterpretation but try to keep what we believe interesting when considering the function of Lsp2 in the context of nutritional programming. We hope the revised manuscript does sound exciting.

Referee #2 (Remarks to the Author):

General comments

In this study, Kosakamoto et al. investigate the mechanisms mediating lifespan extension by protein restriction during development. Using Drosophila melanogaster, a relevant model for the study of longevity, they establish that 24-hours protein restriction in early third instar larvae (ePR) is sufficient to extend the lifespan of wild type adult animals. The effect of this treatment on lifespan with respect to diets fed to adult animals is investigated, along with the fitness cost on growth and reproduction, as well as the effect on nutrient signaling pathways in young adults. They further develop a SILAC assay using differentially labelled amino acids fed to larvae and adults in order to monitor protein turnover across developmental stages. They demonstrate that larval proteins contribute significantly to the adult proteome, in particular ribosomal proteins. Additional analyzes of the amino acid content and of the proteome indicate that ePR impacts amino acids balance and decreases translation in young adults, a process that has previously been linked to lifespan extension, thereby providing a mechanistic link between ePR and lifespan. The authors next search for a functional mediator of this process. Using transcriptomics analyzes, they identify the storage protein Lsp2 as a candidate. Using novel genetic tools, they demonstrate that Lsp2 is actively expressed both during development and in adults, and that ePR lowers its expression both in larvae and young adults. Depletion of Lsp2 during development is sufficient to lower translation in young adults and extends lifespan without compromising fitness. Finally, the authors show that Lsp2 is a reservoir of tyrosine and that tyr restriction in larvae mimics the effect of ePR and Lsp2 depletion on lifespan. The demonstration that the storage protein Lsp2 mediates lifespan extension by ePR is novel, and the SILAC assay used to identify the proteins/amino acids carried over from larvae to adults is particularly elegant. I believe that the identification of storage proteins as potential targets for lifespan extension without fitness/ reproductive cost is highly attractive to a broad audience. Data and their presentation are of high quality. The experimental setup and the statistics used in the study are well defined.

The mechanism leading to Lsp2 depletion in larvae and in adults following ePR remains unclear, and appears to result from protein storage defects. This process is however further documented in the accompanying paper from Wang et al., which nicely complements the present study in this regard.

Response 2-1. We greatly appreciate the reviewer's generous and encouraging comments. We are pleased that the novelty of the findings and the strength of the pSILAC assay were recognised.

In my opinion, the main shortcoming remains the lack of mechanistic details to explain how the depletion of Lsp2 by ePR reduces the translation of ribosomal proteins in young adults to promote lifespan extension. The authors spent efforts to document that Lsp2 is expressed both in larvae and young adults, that ePR decreases its distinct expression in both stages, and that conditional Lsp2 depletion in larvae suppresses adult Lsp2 expression. Though they demonstrate that conditional Lsp2 knockdown in larvae has an effect on early adult translation and lifespan, whether conditional Lsp2 knockdown in young adults (days 0-2) has a similar effect is not addressed. As a result, it is not clear whether the reduced translation observed in young adults results from i) reduced amounts of ribosomal proteins carried over from larvae to adults, ii) a drop out in building blocks required for translation through a storage/recycling function of Lsp2 in larvae and adults, or iii) whether Lsp2 in adults actively participate in the control of translation, possibly through a storage-independent function. The latter point is important to address in my opinion, in particular because the accompanying paper by Wang et al. suggests a direct control of translation by Lsp2 through 4E-BP. These points would be best addressed by further expanding the SILAC assay with and without conditional Lsp2 knockdown in larvae and young adults, respectively, to explore how Lsp2 differentially influences the persistence of larval proteins and the sourcing of amino acid substrates used for translation during early adult stages.

Response 2-2. We agree that this is a central point. To address this, we updated the *in vivo* pSILAC experiment and performed the analysis in flies with or without *Lsp2*-RNAi. We improved our pSILAC assay. Larvae were fed a HolFast diet containing heavy-labeled amino acids, and the

eclosed adults were then switched to a holidic diet containing medium-labeled amino acids starting on day 0. A new mass spectrometer was used to increase the analysis depth. We also performed additional proteomics, translation, and lifespan analyses to test the three possibilities raised by the reviewer by addressing stage-specific Lsp2 function.

During revision, we performed *in vivo* pSILAC analysis in flies with *Lsp2* knockdown. Larva-derived cytoRPs were found to be decreased by *Lsp2* knockdown, while adult-derived cytoRPs seemed to be rather increased (Fig. 4g,h, Extended Data Fig. 9i, Supplementary Table 2). Of note, this increase may be due to the increased food intake or compensatory incorporation of adult-derived amino acids.

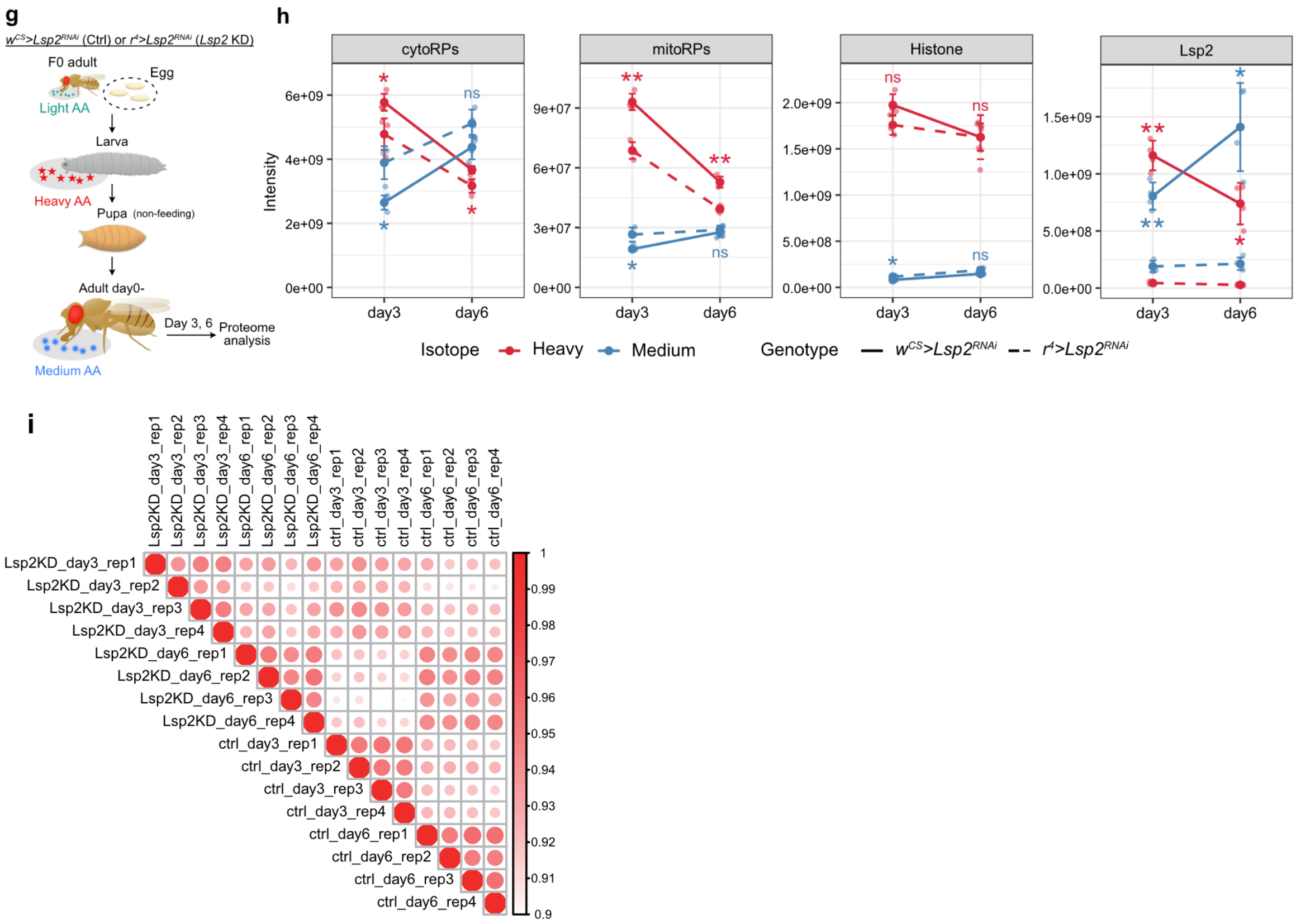

Fig. 4g,h, Extended Data Fig. 9i, *in vivo* pSILAC assay of Lsp2-RNAi flies.

Proteomic analysis of *Lsp2* knockdown flies revealed the suppression of both cytoRPs and mitoRPs, as well as metabolic proteins in early adulthood (Extended Data Fig. 8a-k and Supplementary Table 3), which is reminiscent of the effects of ePR. Intriguingly, *Lsp2*-RNAi did not affect cytoRP levels during the larval stage but reduced them by the prepupal stage, suggesting that Lsp2-dependent regulation of cytoRPs initiates during metamorphosis (Fig. 4c–f, Extended Data Fig. 9a–h, Supplementary Table 3).

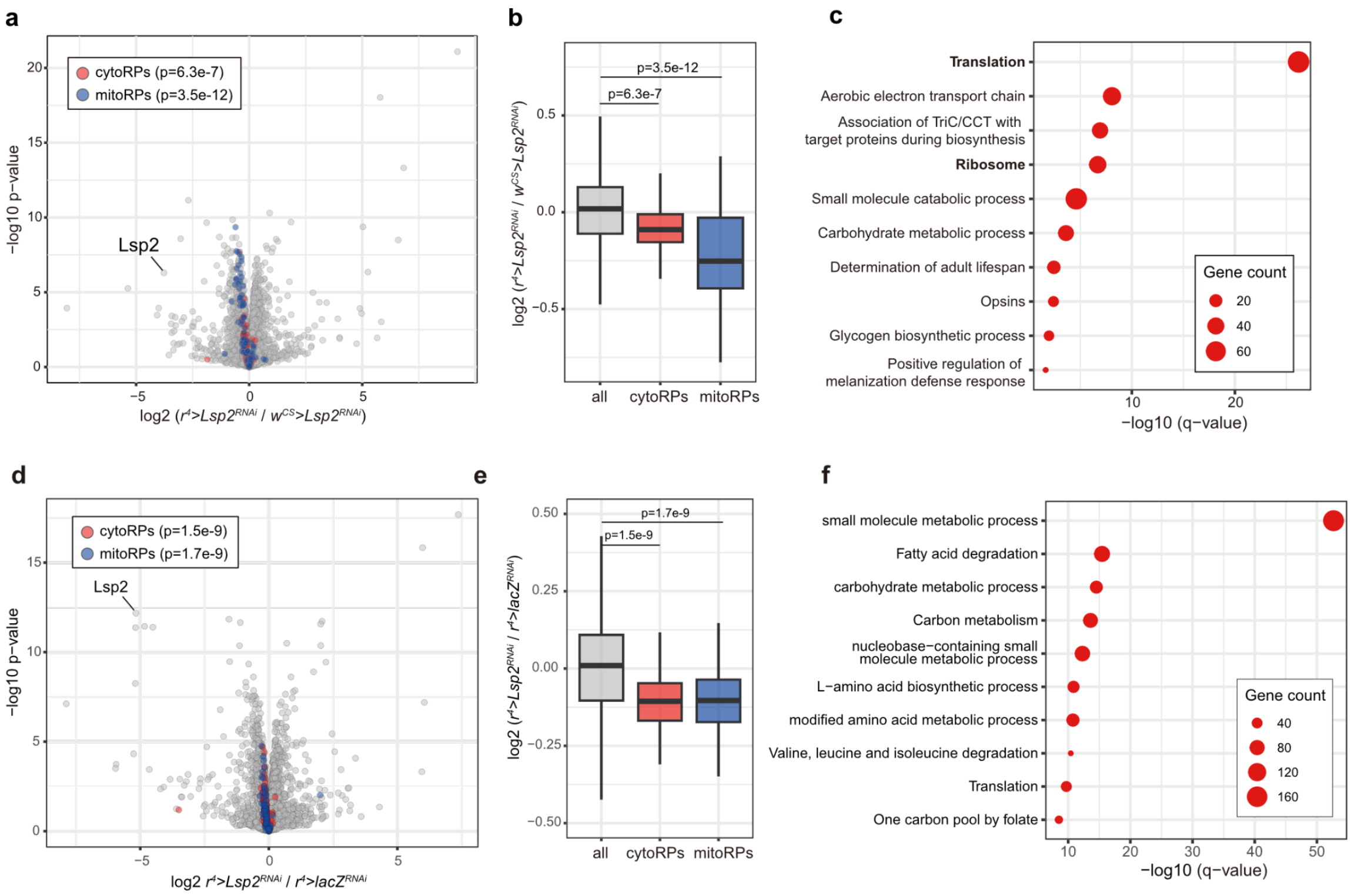

Extended Data Fig. 8a-f, Proteome analysis of adult flies with Lsp2-RNAi.

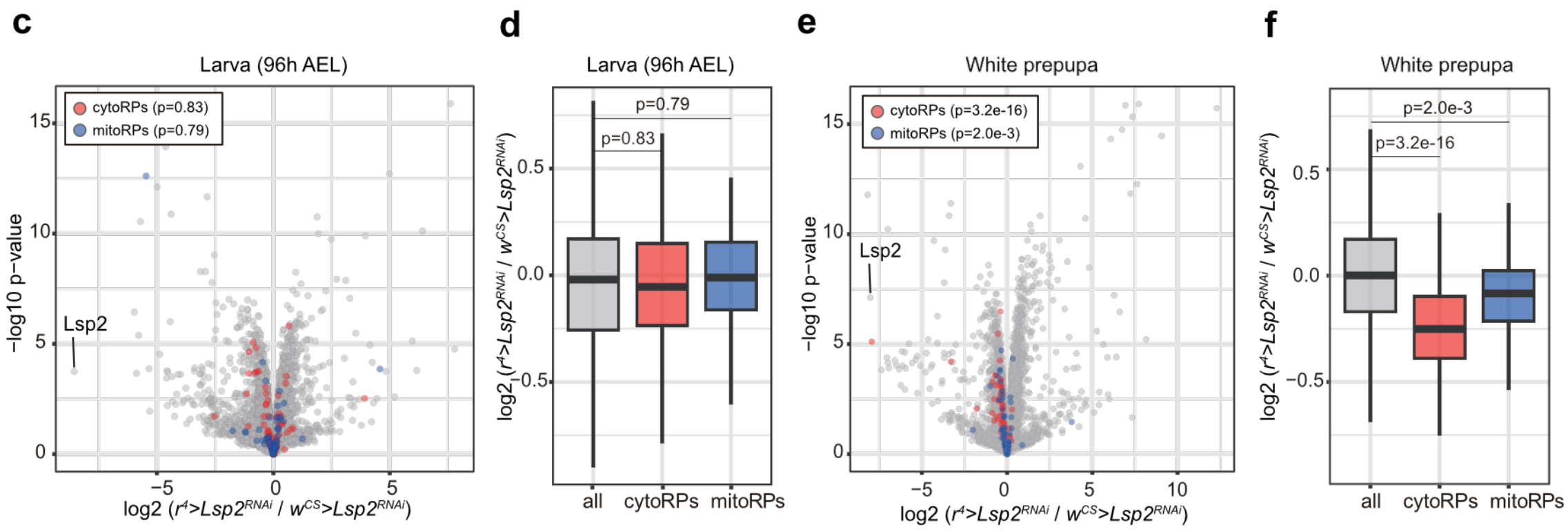

Fig. 4c–f, Proteome analysis of larvae and prepupae with Lsp2-RNAi.

To test whether decrease in Lsp2 protein specifically during the early adult stage is sufficient for suppressing translation capacity and extending lifespan, we performed early adult-specific *Lsp2*-RNAi in the adult fat body. Intriguingly, proteome analysis suggested that cytoRPs were not decreased by adult-specific *Lsp2* knockdown (Fig. 4n,o, Extended Data Fig. 10l–o, Supplementary Table 3). In this experiment, the decrease in Lsp2 is not as strong as developmental *Lsp2*-RNAi, further confirming that larval carry-over Lsp2 is still present in early adulthood (Fig. 4n). However, this manipulation resulted in a remarkable decrease in translation (Fig. 4p,q). This indicates that Lsp2 in early adulthood regulates translation independently of cytoRP abundance. Importantly, adult-specific *Lsp2*-RNAi restricted to the first week of adulthood in the fat body by WBFB-GeneSwitch ($WBFB^{GS}$) extended the lifespan of both females and males (Fig. 4r, Extended Data Fig. 10p). When combined with ePR, *Lsp2*-RNAi in early adulthood rather shortened lifespan of female flies (Fig. 4r). This differs from life-long *Lsp2*-RNAi, which maintained a lifespan comparable to that of ePR-flies (Fig. 4r, Extended Fig. 9m). This result implies that the Lsp2 can exert a life-shortening effect in later-life, to which ePR-flies might be more sensitive. This is consistent with the

findings in an accompanying paper, Wang et al., suggested that *Lsp2* expression increases with ageing that limits lifespan by regulating translation efficiency of cytoRPs.

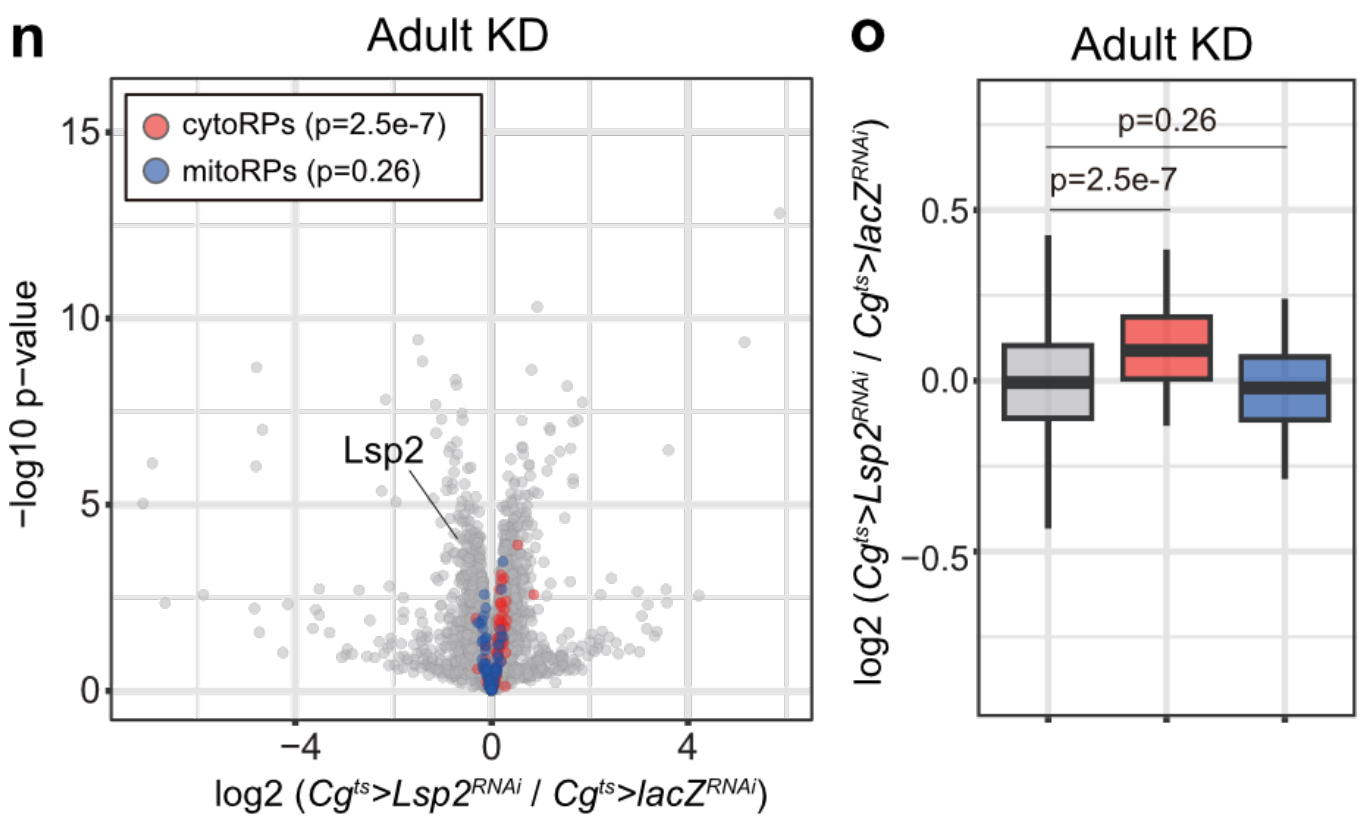

Fig. 4n,o, Proteome analysis in flies with adult-specific Lsp2-RNAi.

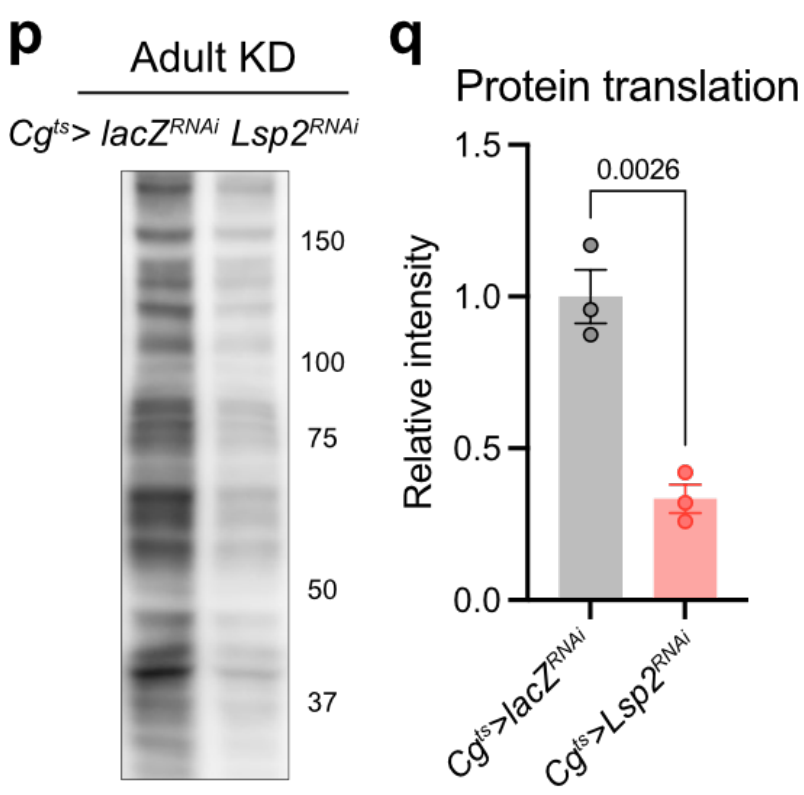

Fig. 4p,q, Translation assay in flies with adult-specific Lsp2-RNAi.

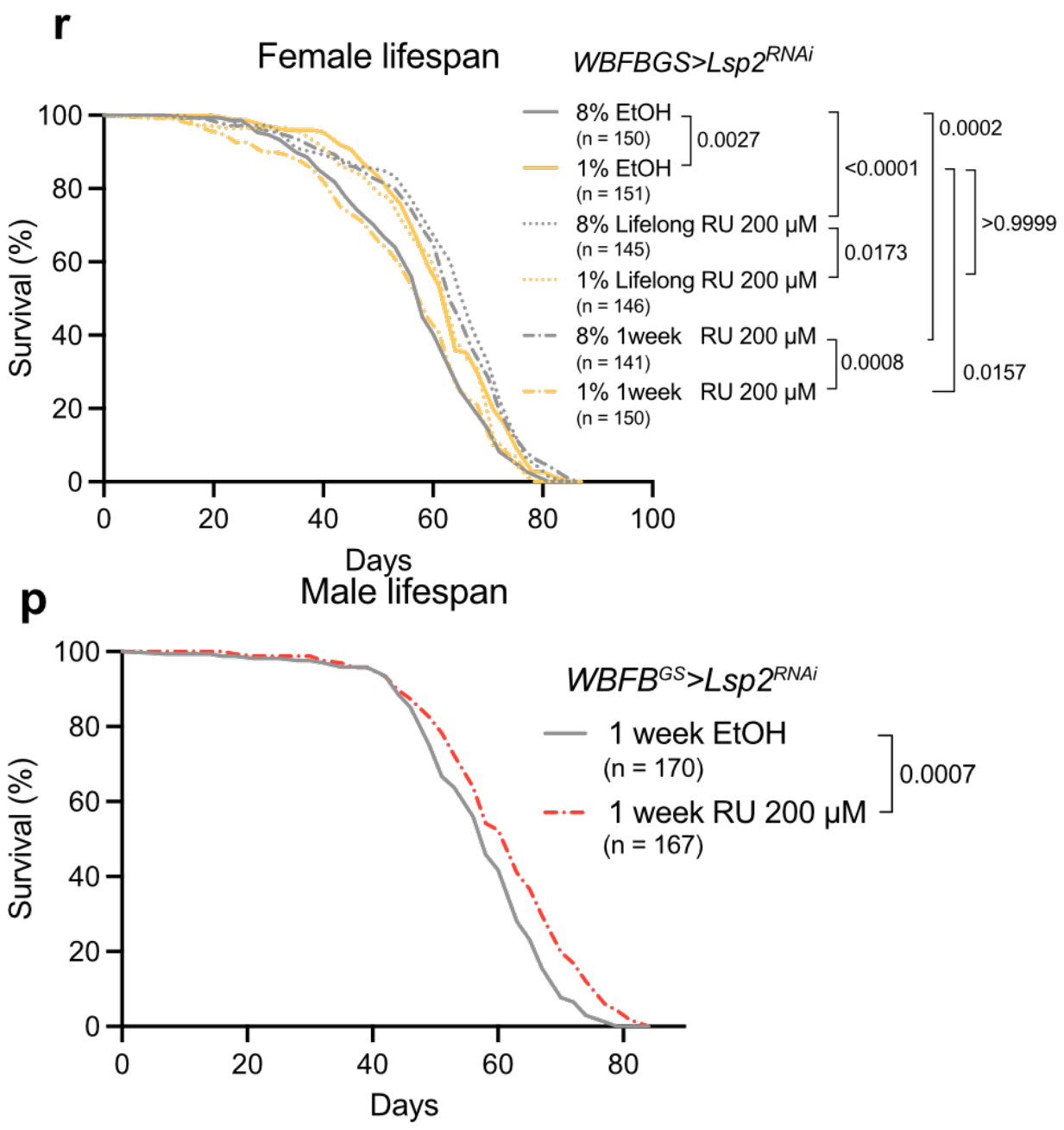

Fig. 4r, Lifespan analysis with adult-specific Lsp2-RNAi.

Based on these data, we have concluded that Lsp2 regulates translation through multiple mechanisms in a life-stage dependent manner as discussed in the revised manuscript shown below.

Discussion:

Lsp2 regulates translation through multiple, stage-dependent mechanisms. During the pupal stage, when feeding is absent, Lsp2 likely supports translation by directly supplying amino acids, thereby sustaining the synthesis of high-demand proteins. cytoRPs are among the most abundant proteins and therefore particularly sensitive to this supply. Fat body protein 1 (Fbp1), one of the storage proteins required for re-uptake of Lsp proteins into the fat body for amino acid utilisation, is induced just before pupariation[43]. Consistent with this model, Lsp2 knockdown reduces cytoRP abundance, with effects detectable already at the prepupal stage. These findings suggest that storage-derived amino acids are mobilised early and preferentially allocated to sustain core translational machinery. In early adulthood, Lsp2 continues to support translation, although not exclusively through cytoRP abundance, indicating additional regulatory layers. It is possible that the direct provision of stored amino acids to fuel translation is crucial during this period, although the possibility that Lsp2 also functions as a nutrient signal cannot be excluded. In later adult stages, Lsp2 shifts toward regulating translation efficiency via the mTORC1–4E-BP axis, as demonstrated by Wang et al., thereby controlling the translation of 5' Terminal Oligo Pyrimidine (TOP) mRNAs, including those encoding cytoRPs[47]. Interestingly, the turnover rate of cytoRPs is known to be associated with ageing and lifespan in various organisms[53,54]. Together, these findings support a model in which Lsp2 coordinates translational control across developmental stages, ultimately influencing organismal lifespan.

Specific remarks aimed at strengthening results and their interpretation

- To interpret the pSILAC assay, it would help to define the extent of protein labelling achieved after each pulse (heavy in larvae and medium in adults). It is likely that a significant fraction of proteins remains unlabeled after each pulse. This mitigates the interpretation of the labelled fractions, as well as of the light one that may not only result from feeding on unlabeled food during days 0-2.

Response 2-3. We appreciate the reviewer very much for this critical suggestion. We performed pSILAC assay in the larval stage. As expected, the labeling efficiency in wandering larvae was 99.4%, suggesting nearly complete substitution with heavy-labeled amino acids at this stage (Fig. 2b).

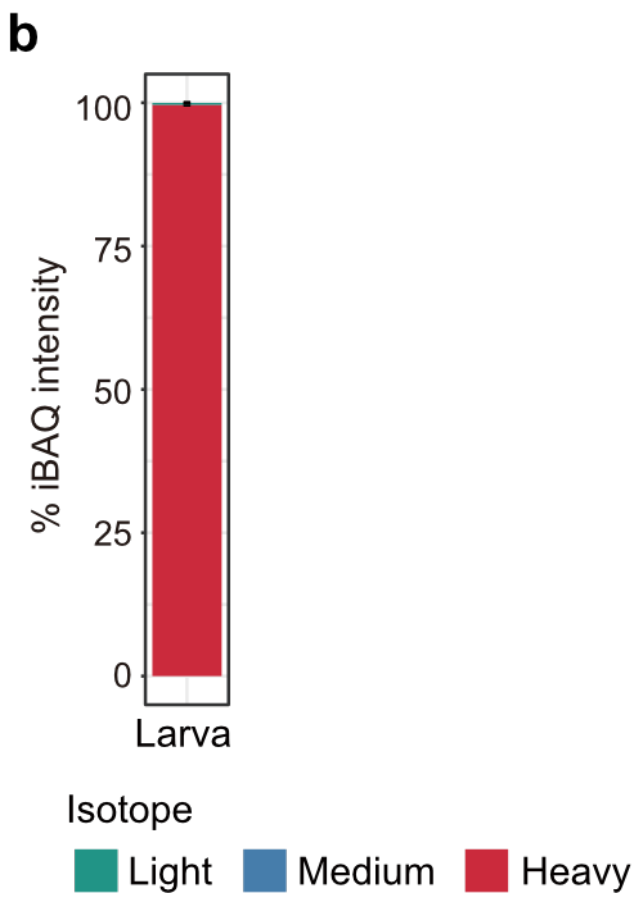

Fig.2b, Quantification of isotope labelling efficiency in the larval stage.

- Is the food intake in larvae, young (d0-2) and older adults similar? Otherwise, should not the labelling values resulting from each pulse be normalized to the corresponding tracer concentration to interpret the protein turn-over and the origin of the amino acid substrates used for translation?

Response 2-4. We agree with the reviewer for this point. Indeed, interpretation of the pulse labelling results between different life stages was not appropriate. The following sentence has been removed in the revised text.
The duration of isotope labelling (four days) in the larval stage (heavy isotope) and the adult stage (medium isotope) were matched to enable direct comparison.

- What is the expression level of Lsp2 in adults +/- ePR later than day 3?

Response 2-5. At least by day 7, Lsp2 RNA expression continued to be declined as shown in Extended Data Fig. 6a. However, our new pSILAC analysis in ePR-flies also showed that Lsp2 protein levels were gradually recovered (Fig. 3k). Therefore, the effect of ePR on Lsp2 persists for at least one week, but it can recover in later stages.

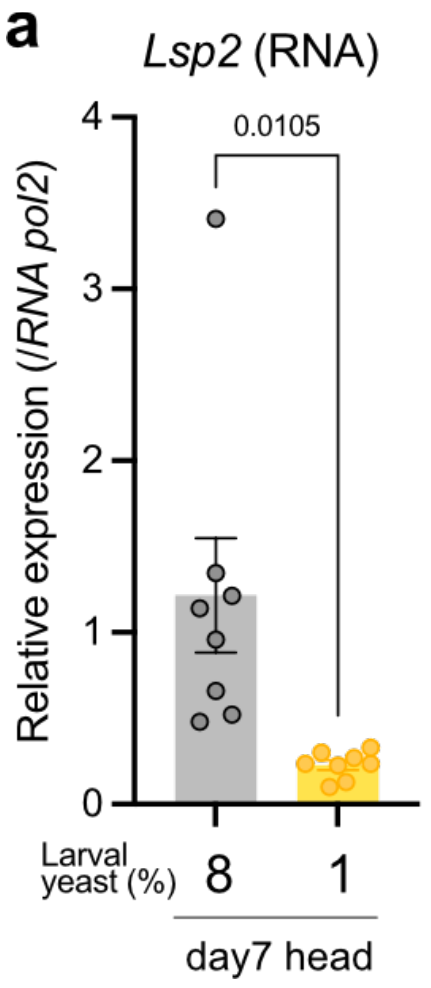

Extended Data Fig. 6a, Quantitative RT–PCR analysis of *Lsp2* mRNA levels at adult day 7.

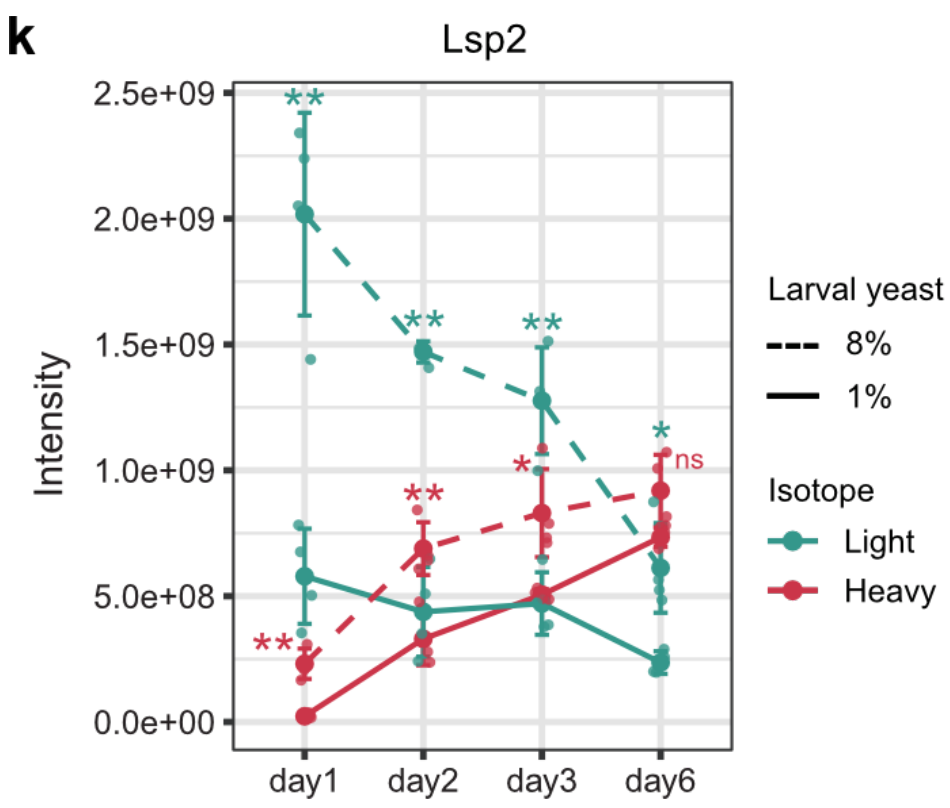

Fig. 3k, pSILAC analysis of Lsp2 in the first week.

- Figure 5: Would His and Phe restriction phenocopy that of Tyr?

Response 2-6. We performed restriction of His, Phe, and Ile during the larval stage. We found that restriction of His and Phe extended lifespan, while Ile restriction did not. We have added these data and revised the manuscript as shown below. Please also refer to our Response 1-17.

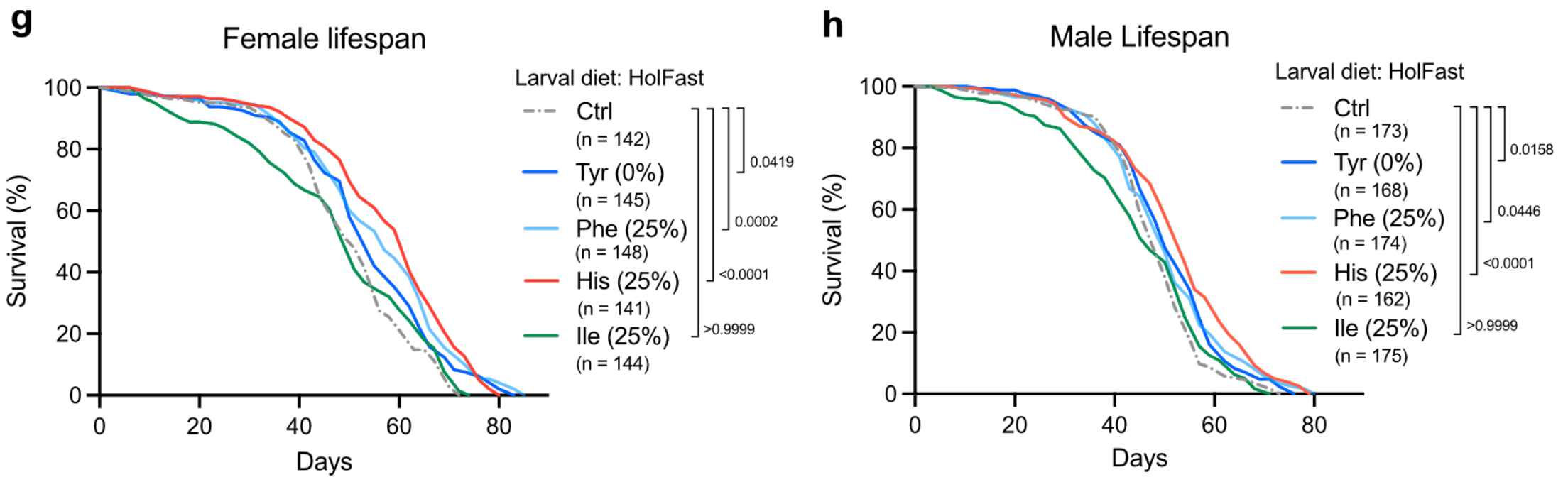

Fig. 5g,h, Lifespan analysis of the flies with larva-specific single amino acid restrictions.

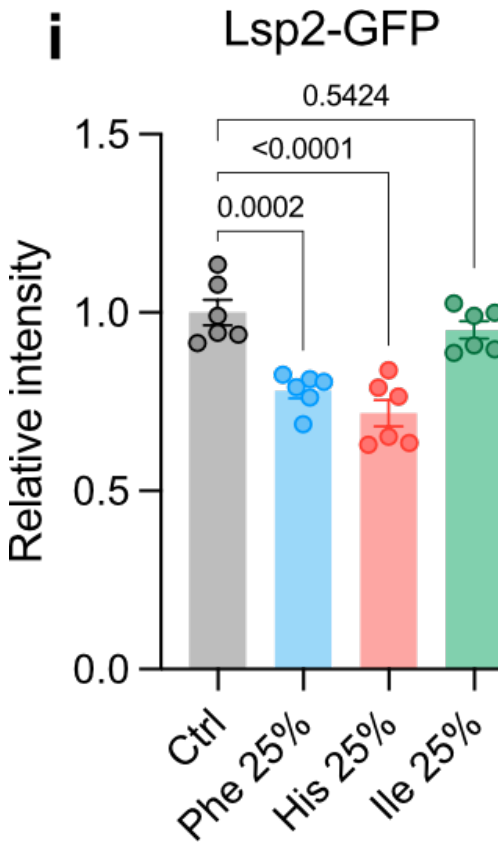

Fig. 5i, Lsp2-GFP quantification of the flies with larva-specific single amino acid restrictions.

Results section "Single amino acid manipulation in the larval stage extends lifespan"

To test amino acid specificity, we restricted other amino acids. Since the complete restriction of essential amino acids prevents adult emergence and the synthetic diet used above for Tyr deprivation produces semi-lethality, we utilised the larva-optimised HolFast diet to decrease essential amino acids, such as Phe, His, and isoleucine (Ile), to 25%. We confirmed that Tyr deprivation also extends lifespan in this experimental diet (Fig. 5g,h). Restriction of Lsp2-enriched amino acids, such as Phe and His, increased lifespan in both males and females (Fig. 5g,h). In contrast, restriction of Ile, which is less abundant in Lsp2, did not extend lifespan (Fig. 5g,h). Consistently, we found that the GFP fluorescence of Lsp2-muGFP decreased upon restriction of Phe or His, but not Ile (Fig. 5i). Interestingly, Ile restriction induced a stronger developmental delay than Phe or His restriction, suggesting that Lsp2 can act as a buffer to support larval development under nutritional stress by providing stored amino acids (Fig. 5j). Taken together, these data suggest that the larval intake of dietary amino acids enriched in Lsp2 regulates the abundance of Lsp2 proteins in early life stages, which in turn modulates adult translational capacity and lifespan (Fig. 5k).

- In a previous study, Kosakamoto et al; 2022, the authors implicate Tyr in the control of mTOR and 4E-BP through ATF4. Given that Larval depletion of Lsp2 depletes Tyr in day2 adults (Fig.5b), and that this is associated to increased ATF4 signaling at least in new born adults (Ext data fig 9i, j), could a drop in mTOR-4E-BP signaling in early adults explain the decrease in translation?

Response 2-7. We speculate that mTOR signalling in early adulthood might be decreased, given that mTORC1 regulators, including ATF4, insulin signalling, and Lsp2, are all altered. Due to the lack of technical tools, we could not directly test this. Nevertheless, in the revised manuscript of Wang et al., they showed that in the early adult stage, Lsp2-mTOR-4E-BP signalling was not working. Furthermore, as discussed in Response 1-11, mTORC1 activation by *Rheb* overexpression in early adulthood influenced the effect of ePR on lifespan. We acknowledge, however, that mTORC1 signalling does not exclusively stimulate translation but also regulates various processes, including Lsp2 level *per se*. We discussed these points in the revised manuscript.

Additional remarks aimed at improving manuscript clarity

- Page 6, while describing the pSILAC assay, it would help to mention the composition of the heavy and medium tracers in the main text (+8 Lys/+10 Arg vs +4Lys/+6Arg) if space allows to avoid switching to the methods to understand the experiment.

Response 2-8. We specified heavy (+8 Lys/+10 Arg) and medium (+4 Lys/+6 Arg) tracers in the main text for clarity.

Results "Larval diet leaves a lasting impact on the adult proteome":
Larvae were reared on a "heavy" synthetic diet containing isotope-labelled lysine (+8 Lys; $^{13}C_6$, $^{15}N_2$) and arginine (+10 Arg; $^{13}C_6$, $^{15}N_4$) in place of their "light (natural)" counterparts (Fig. 2a). This approach allowed for the complete replacement of these dietary amino acids with stable isotopes without perturbing larval development, enabling precise quantification of proteome turnover under physiological conditions. Upon eclosion, adult flies were switched to a synthetic diet containing "medium" isotope-labelled Lys (+4 Lys; D_4) and Arg (+6 Arg; $^{13}C_6$).

- Page 7 line 182 and 198. Rephrase or add a reference for His2B as a long-lived protein.

Response 2-9. We added citations in the revised manuscript.

- Page 8 line 203. "some proteins can be particularly influenced by larval protein restriction". The statement is too vague, please cite or rephrase.

Response 2-10. We removed the sentence and edited the relevant text.

- P. 9 l.236, fig.2L. It is not clear whether RU was fed only to young adults to repress early translation, or throughout adult life.

Response 2-11. The original experiment was life-time manipulation. We added the early-adult specific manipulations here as discussed in Response 1-11.

- P.11 l.301-302: please cite or refer to fig.4.
- Extended data fig. 8 a and b, please clarify the age of the animals analyzed.
- P.11l.312: I suggest rephrasing as follow: "we knocked down Lsp2 in the larval fat body"
- Fig.4 i-k. As this experiment would typically use Gene Swich drivers to avoid confounding effects of heterogeneous genetic backgrounds, please indicate in the text that all the lines used were isogenized to Canton S.
- P.12l.347: I suggest rephrasing as follow: "knocking down Lsp2 in larvae significantly reduced the overall composition of the two amino acid in young adults.

Response 2-12. We revised these points accordingly.

Looking forward to reading a revised version of the manuscript,
With best regards.

Response 2-13. We are very encouraged by the reviewer's concluding remarks. Thank you so much for the supportive assessment.

Referee #3 (Remarks to the Author):
The paper addresses the mechanisms by which yeast or protein restriction in the juvenile period extends adult lifespan in Drosophila. This is a potentially interesting topic, although its broader relevance outside insects is not clear. The study shows that there is an effect of yeast concentration in the diet during larval development on adult lifespan, although the magnitude of this effect depends on the adult diet and is absent at high yeast concentration. The authors also demonstrate carry over of amino acids taken up in the larval period into adult proteins. However, any mechanistic link between these two findings is unclear and the study has weaknesses that make it unsuitable for publication in its present form. Most important of these are: 1. conflation of data from yeast and protein restriction, despite the demonstration in the MS that these must be operating through different mechanisms, 2. Lack of replication and appropriate statistical analysis of lifespan data, 3. lack of clarity about the exact role of Lsp2 and other 'storage' proteins.
The paper is not well written. It needs a language edit and the authors need to explain why they did each experiment. For instance, on line 169 the authors say what they did next, but not why they did it, and there are several such examples.
Response 3-0. We thank the referee for the constructive assessment. We have carefully revised the manuscript to make the rationale for each experiment and logical flow of the study clear.

Specific points:
1. The number of eggs in Figure 1 looks much too low
Response 3-1. We thank the reviewer for this notion. Fecundity varies among conditions and we are aware that the original data we provided was from the batch of unideal conditions (relatively old diet). We have repeated the experiment with a freshly prepared diet. We confirmed that the control group produced standard levels of eggs and ePR-flies have decreased egg-laying rates (Fig. 1h,i). We have revised the manuscript.

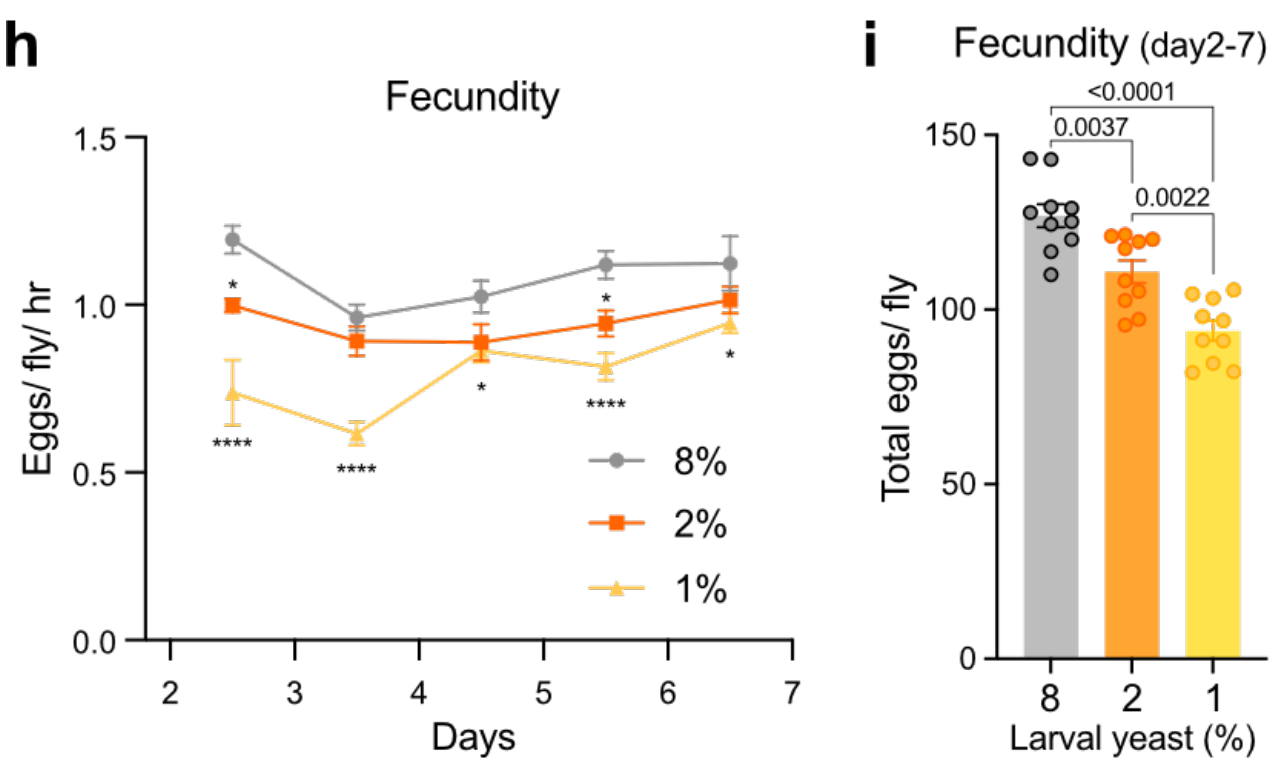

Fig. 1h,i, Fecundity of Canton-S females after ePR.

2. The authors state that the effects of juvenile and adult yeast concentration on lifespan were additive, but this is clearly not the case. In Figure 1n the effects of juvenile diet were greater in adults that were fed a lower yeast diet and were non-significant when the adults were exposed to 12% yeast. The effects of adult diet were also larger than those of juvenile diet. The data in Figure1 i-n should be analysed using Cox Proportional Hazards so that the effects of different yeast restrictions in I and m can be appropriately compared and the interaction between juvenile and adult diet in Figure 1n tested for statistical significance. It should be clearly stated that the effect of juvenile diet on lifespan was conditional on the adult diet – at the moment the opposite statement is made starting on line 101.
Response 3-2. In the revised manuscript, we incorporated the 0% yeast condition in this lifespan analysis and replaced the data, in line with the Response 3-3. We reanalysed the data using CoxPH models to test for main effects and interactions. Within the physiological range of the yeast extract (4% or 1%) during adulthood, adult protein restriction (from YE 4% to 1%) alone led to a 46% increase in median lifespan, while ePR (yeast 8% to 1%, with 4% YE in adulthood) alone led to a 35% increase. The combination of both manipulations resulted in a 69% increase. CoxPH analysis revealed no

significant interaction between larval and adult diets within this range, suggesting that ePR extends lifespan independently of the adult diet (Supplementary Table 1). However, this benefit was abolished when flies were subjected to extreme dietary conditions in adulthood, such as a very high-yeast (12% YE) diet or a yeast-deprived (0% YE) diet (Fig. 1q,r). We have revised the text accordingly. Regarding this point, please also see our Response 3-3.

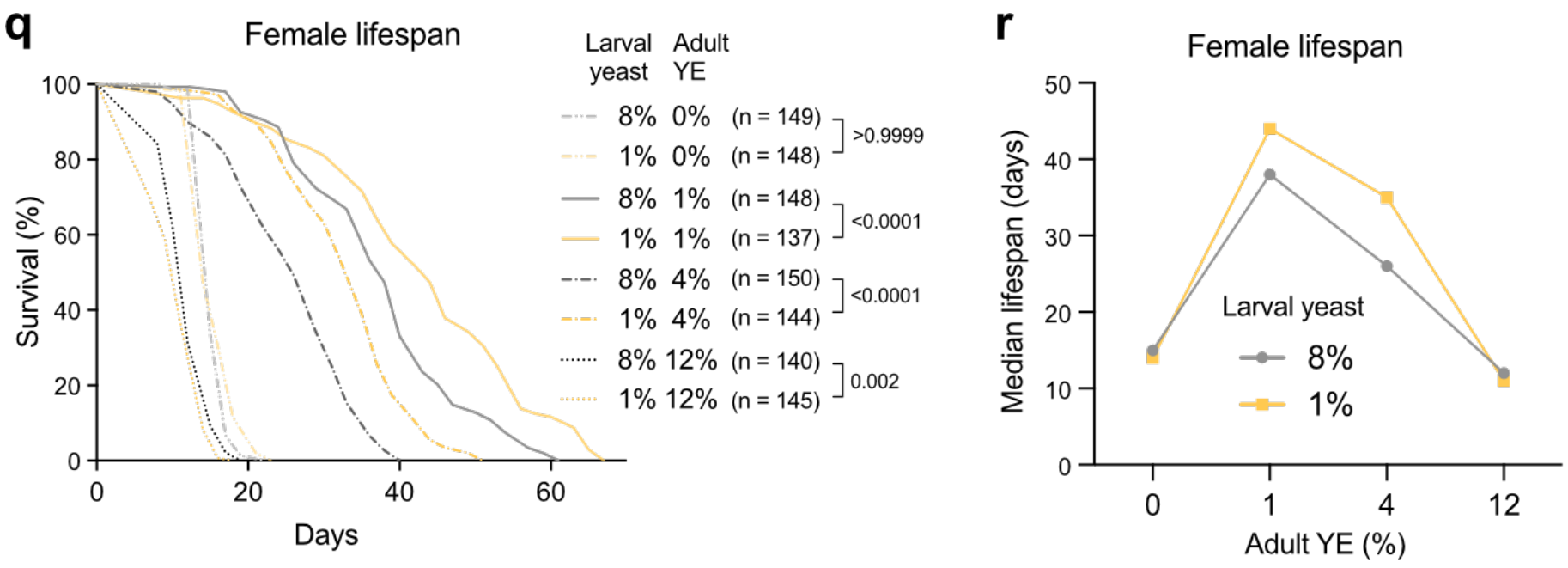

Fig. 1q,r, Lifespan analysis of ePR-flies under varying concentrations of yeast extract in adult.

3. The data on the effect of amino acid (AA) concentration in Extended data Fig 1 show a consistent effect of larval diet on lifespan across a range of AA concentrations between 10% and 100%, with a reversal only when they were fed 1%. This result is different from the result with varying yeast concentrations in Figure 1, where there is an indication of an interaction between adult and juvenile diet and the other way around, with the effect of juvenile diet being reduced by high, not low, yeast concentrations. This must indicate that the effect of AA concentration in the juvenile diet was being mediated by different mechanisms from the effect of yeast concentration. These data are thus not relevant to the effects of dietary yeast.

Response 3-3. Our interpretation is that both datasets support a similar principle: extremely high protein conditions (12% YE), or complete deprivation (0% YE) abolish the ePR effect, whereas intermediate conditions show additive effects with adult DR. To strengthen this point, we tested 0% yeast extract (Fig. 1q,r, see our Response 3-2) and 300% amino acids (Extended Data Fig. 1h,i,o,p) in the adult diet. Lifespan extension by ePR persisted within a range of 10%–100% amino acids but was lost when adult flies were kept under extreme nutritional environments (0% or 300% amino acids) (Extended Data Fig. 1i,p). CoxPH analysis of moderate amino acid conditions in adulthood (10%–100%) revealed no significant interaction between larval diet and adult diets (Supplementary Table 1). Thus, the mechanisms underlying ePR-induced longevity appear not to completely overlap with those of adult amino acid restriction. As discussed here and in our Response 3-6, in our hands, yeast extract and amino acid manipulations yield largely comparable phenotypes.

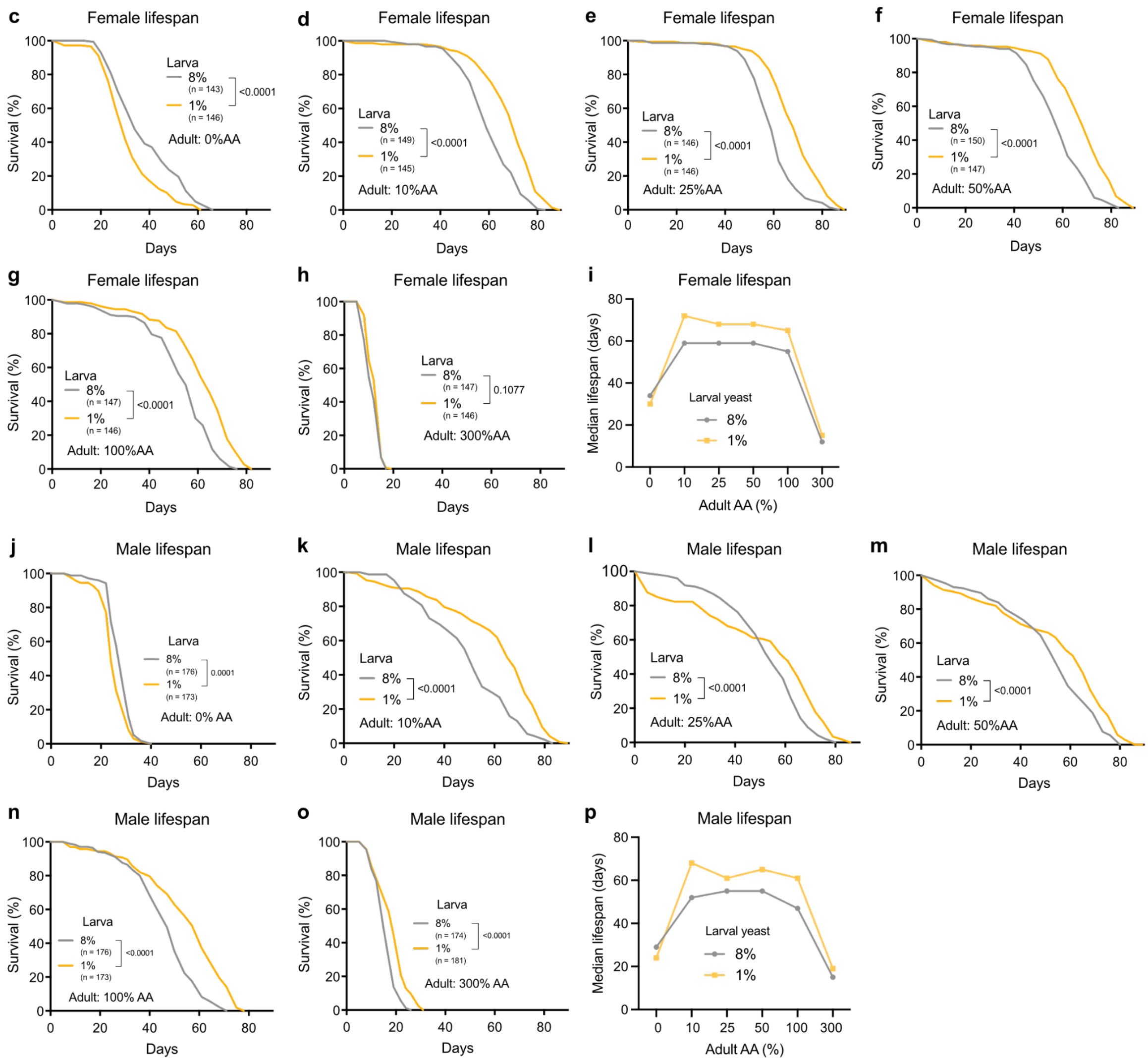

Extended Data Fig. 1c–p, Lifespan analysis of ePR-flies under varying concentrations of amino acids in adult.

Results "Early-life protein restriction extends lifespan":

Interestingly, ePR-flies exhibit a further increase in lifespan when combined with adult protein restriction (4% vs 1% yeast extract [YE]) (Fig. 1q,r). Cox proportional hazard (CoxPH) analysis revealed no significant interaction between larval and adult diets within the moderate range of adult diets (4% or 1% YE), suggesting that ePR extends lifespan independently of the adult diet (Supplementary Table 1). However, this benefit was abolished when flies were subjected to extreme dietary conditions in adulthood, such as a very high-yeast (12% YE) diet or a yeast-deprived (0% YE) diet (Fig. 1q,r). Similar trends were observed in both sexes using a holidic medium to adjust amino acid levels in the adult stage (Extended Data Fig. 1c–p). Lifespan extension by ePR persisted within a range of 10%–100% amino acids during adulthood but was lost when adult flies were kept under extreme nutritional environments (0% or 300% amino acids) (Extended Data Fig. 1i,p). CoxPH analysis within moderate amino acid conditions in adulthood (10%–100%) revealed no significant interaction between larval diet and adult diets (Supplementary Table 1). Thus, the mechanisms underlying ePR-induced longevity appear not to substantially overlap with those of adult amino acid restriction.

4. The data on insulin signalling in Extended Data Fig 2 are based on the effects of dietary yeast. The concentration of the larval diet is given, but it is not stated what was the concentration in the adult diet of the flies that were assessed – I assume 8% or 4%? This is important because the effects of larval

diet were conditional on adult diet in Figure 1.

Response 3-4. The adult diet was our standard 6% yeast diet (not yeast extract). For all ePR manipulation except for the experiments combined with adult amino acid manipulation or pSILAC analysis, we used this standard yeast diet for adults as mentioned in the Methods section. We would like to emphasise that ePR phenotype are consistent across adult diets (except an extreme case, such as 0% or 12% yeast extract).

5. Line 131 has a statement that the effect of larval diet on insulin signalling was transitory. This is quite typical of the signalling domain but were the changes in gene expression at the transcript level maintained with time?

Response 3-5. We performed qPCR at day 7 to determine whether transcript-level changes persist. InR expression was not altered in ePR-flies at day7, suggesting transient change of insulin signalling (Extended Data Fig. 2l).

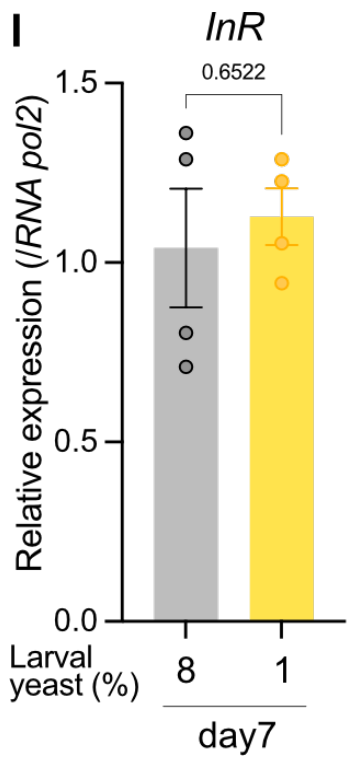

Extended Data Fig. 2l, Quantitative RT–PCR analysis of *InR* at day 7.

6. Line 144 refers to protein intake, but there was a disconnect in this study between the effects on lifespan of yeast and of amino acids as outlined above. The data presented are consistent with the hypothesis that the effects of yeast were not being mediated by its AA content.

Response 3-6. As explained in our response 3-3, yeast and amino acids are equivalent. To test this more directly, we performed additional dietary manipulations during larval stage, namely 1) ePR with amino acid supplementation, and 2) amino acid restriction, in order to assess whether yeast effects are mediated via amino acids. The results clearly showed that the effect of yeast deprivation was recovered by adding amino acids (Fig. 1m,n), and amino acid restriction mimicked yeast restriction (Fig. 1o,p). We appreciate the reviewer's criticism on this matter, which led us to significantly improve the quality of the study.

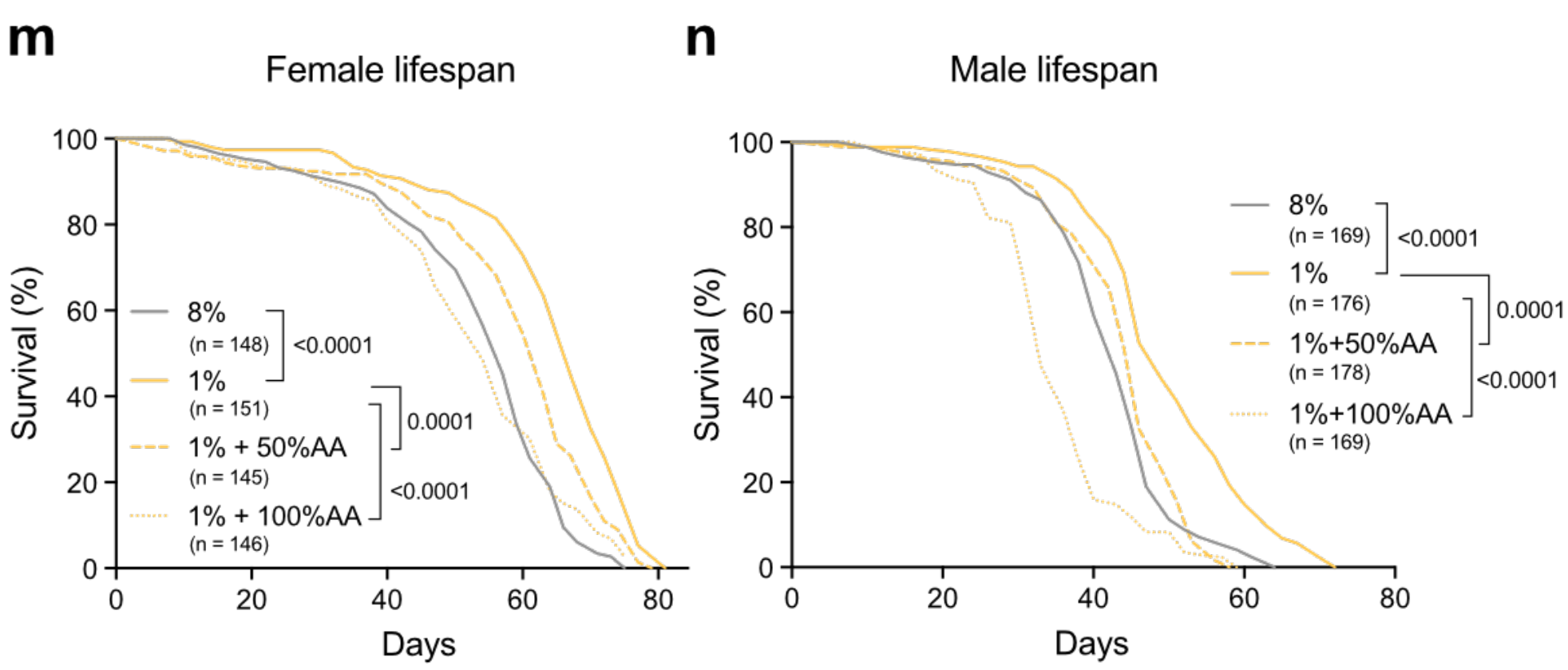

Fig. 1m,n, Lifespan analysis of ePR-flies with or without amino acid supplementation in the larval diet.

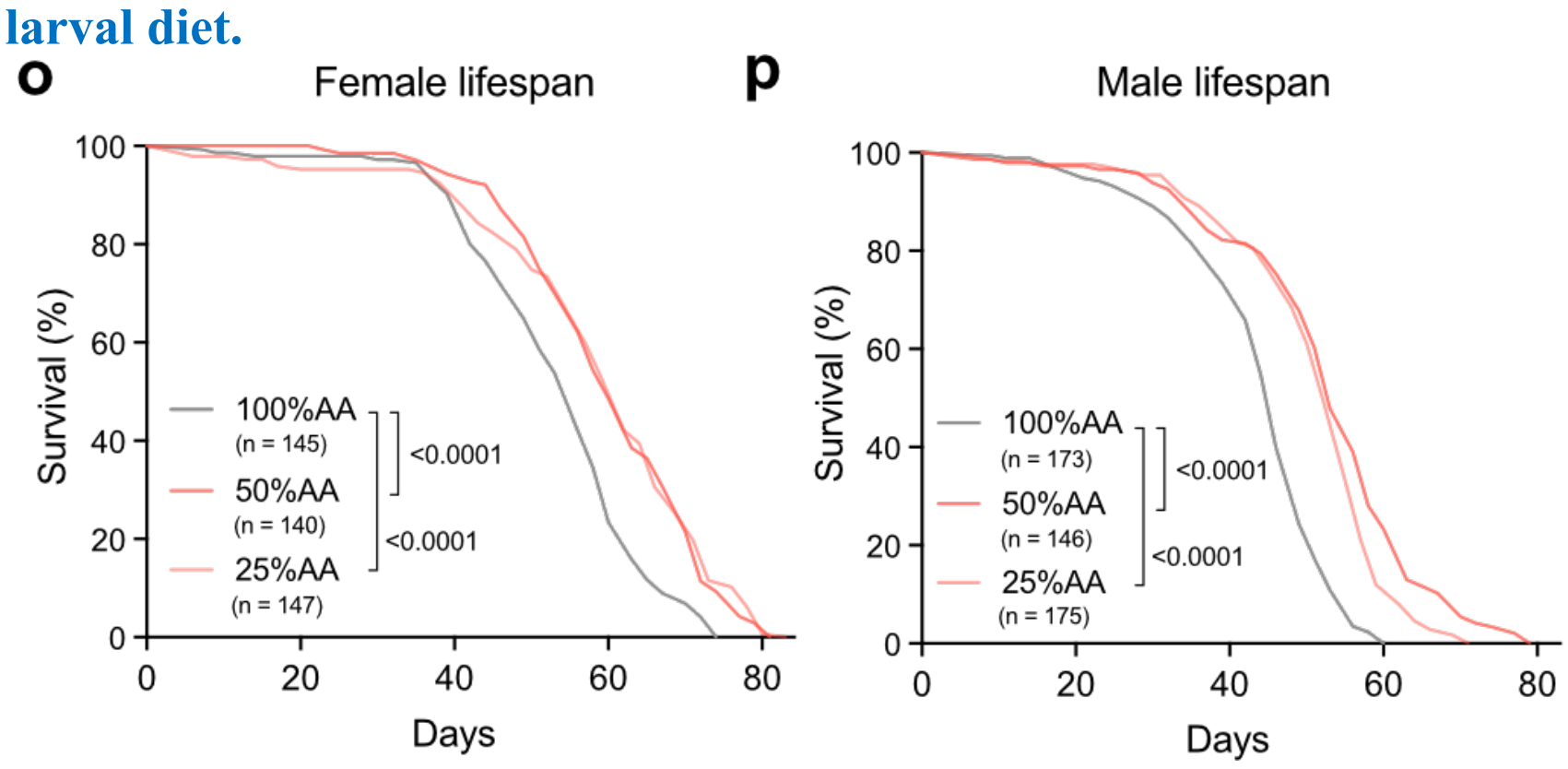

Fig. 1o,p, Lifespan of Canton-S flies with larval-specific amino acid restriction.

Results "Early-life protein restriction extends lifespan":
To determine whether this longevity was specifically due to reduced protein (i.e. amino acid) levels rather than other yeast-derived nutrients, we supplemented the 1% yeast diet with an amino acid cocktail based on the defined composition of a synthetic diet[24–26] (see Methods). This supplementation effectively abolished the lifespan extension in ePR-flies of both sexes (Fig. 1m,n), suggesting that ePR-induced longevity is driven by amino acid availability. This was further corroborated using HolFast, a chemically defined diet designed for larvae[27]. Decreasing dietary amino acids to half or one-fourth specifically during the larval stage extended adult lifespan under standard diet conditions (Fig. 1o,p).

7. In the SILAC analysis a synthetic diet was used, presumably at one of the AA concentrations mentioned earlier. The use of the synthetic diet is understandable given the question being asked but, as outlined above, it may not be relevant to the effects of a yeast diet. To be sure of that it would be necessary to label the yeast with H or M AAs.
Response 3-7. We believe responses 3-3 and 3-6 clarify that yeast and amino acid manipulations produce similar outcomes. We believe additional pSILAC using isotope-labelled yeast unnecessary.

8. Line 162 refers to H/M ratios but the authors have not defined H and M.
Response 3-8. We spelled out H and M to indicate heavy and medium respectively.

9. Starting on line 202 there is study of the effects of 'ePR' on the amino acid and protein composition of the flies. It is not stated whether this is yeast restriction or AA restriction on a defined diet. To understand the specific role of proteins synthesis in the juvenile stage it would be necessary to combine the SILAC study with the manipulation of the juvenile diet.
Response 3-9. We thank the reviewer for this transformative suggestion. As mentioned in our Response 1-10, we conducted an additional pSILAC analysis in ePR-flies, and demonstrated that larval protein restriction reduces adult cytoplasmic ribosomal proteins levels that depend on larval-derived amino acids. These data have been added to the revised manuscript as shown below.

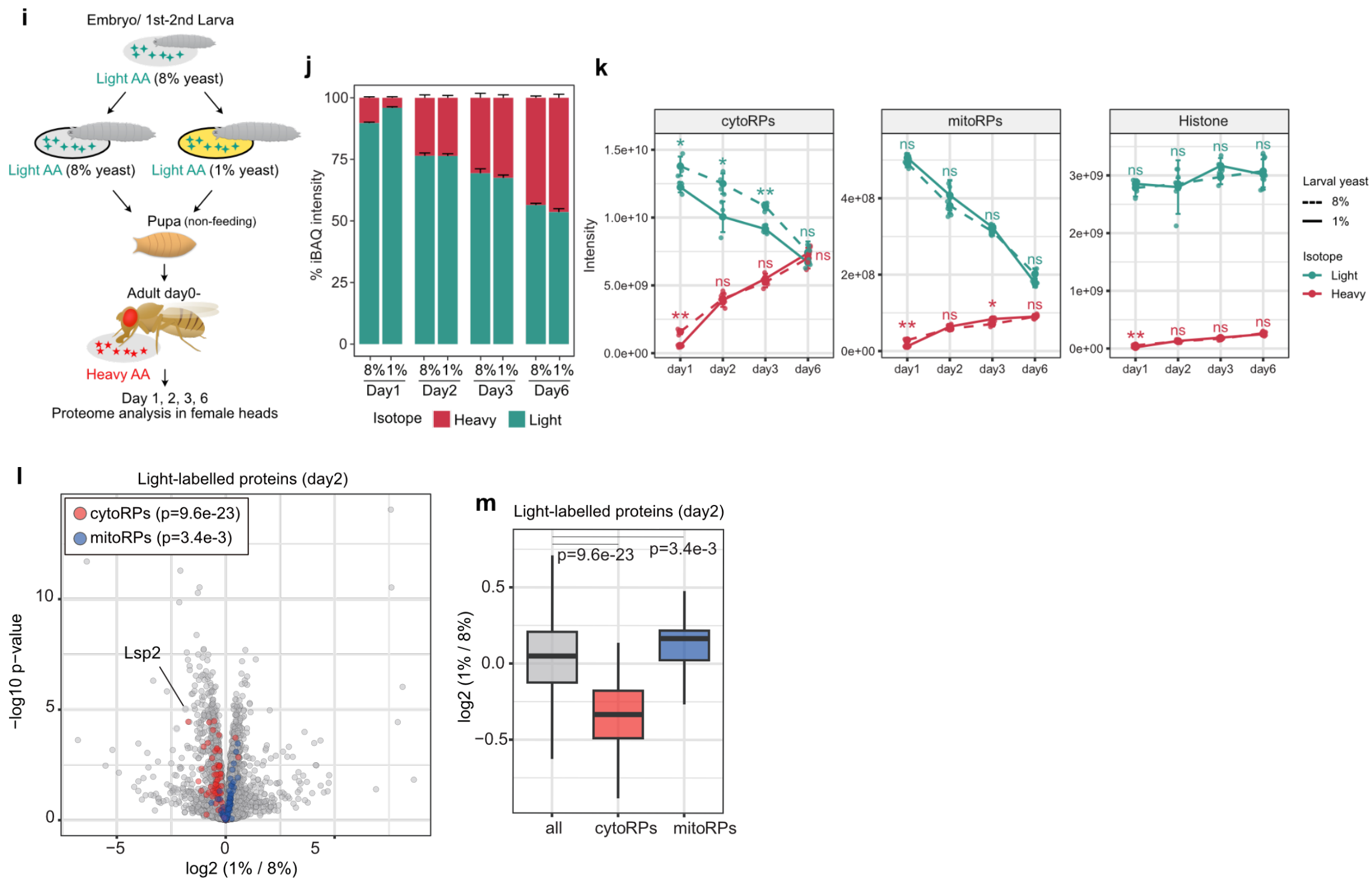

Fig. 2i-m, time-course *in vivo* pSILAC analysis of ePR-flies.

Results section "ePR decreases ribosomal proteins and decreases translation in early adulthood"

To determine if ePR modulates the adult proteome turnover, we performed a time-course in vivo pSILAC assay (Fig. 2i, Extended Data Fig. 4h-k, and Supplementary Table 2). In this experiment, larvae were reared on either an 8% or 1% yeast diet (containing light amino acids), and the newly-eclosed adults were switched to a synthetic diet containing heavy amino acids (+8 Lys/+10 Arg). We prepared samples at days 1, 2, 3, and 6 to monitor the proteome turnover over time (Fig. 2i). Consistent with the results in Fig. 2c, the heavy labelled proteomes were enriched over time and reached about half at day 6 (Fig. 2j). At day 1, ePR-flies exhibited a lower heavy fraction; however, from day 2 onwards, the heavy/light ratio became nearly identical to controls (Fig. 2j). The reduced incorporation of adult-derived (heavy) amino acids at day 1 in ePR-flies suggests not only reduced protein synthesis but also decreased feeding rates. Nevertheless, after day 2, the relative reliance on larva- versus adult-derived amino acids for the global proteome did not differ significantly between groups (Fig. 2j).

We then specifically examined the turnover of ribosomal proteins (RPs). In control flies, both cytoRPs and mitoRPs were gradually replaced by heavy-labelled (adult-derived) proteins, with cytoRPs showing faster replacement than mitoRPs (Fig. 2k). In ePR-flies, however, light-labelled (larva-derived) cytoRPs were significantly lower during the first three days (Fig. 2k). From the volcano plot from the day 2 samples, we confirmed that larva-derived cytoRPs are lower in ePR-flies (Fig. 2l,m). Notably, while heavy-labelled (adult-derived) cytoRPs were also transiently decreased at day 1, they became comparable to controls by day 2 (Fig. 2k). The transient decrease at day 1 in heavy-labelled proteins was commonly observed across cytoRPs, mitoRPs and histones, reflecting a general reduction in protein synthesis or feeding at the onset of adulthood by ePR (Fig. 2j,k). Together, these data suggest that ePR selectively reduces the pool of larval carry-over cytoRPs without affecting the synthesis of new RPs from adult-acquired amino acids.

Thanks to this great suggestion, we are now able to analyse how much adult Lsp2 is derived from larval amino acids and how they turn over (also related to response 1-15). In the control flies, Lsp2 protein at day 1 is predominantly light-labelled, which is gradually replaced by heavy-labelled Lsp2. At day 6, heavy-labelled Lsp2 are more than light-labelled Lsp2. As we expected, light-labeled Lsp2 is significantly lower by ePR for all time points (days 1, 2, 3, and 6). In ePR-flies (1% yeast), heavy-

labeled Lsp2 is also decreased during the first three days, which is consistent with the decreased Lsp2 expression in ePR-flies. We incorporated these data in the revised manuscript (Fig. 3k).

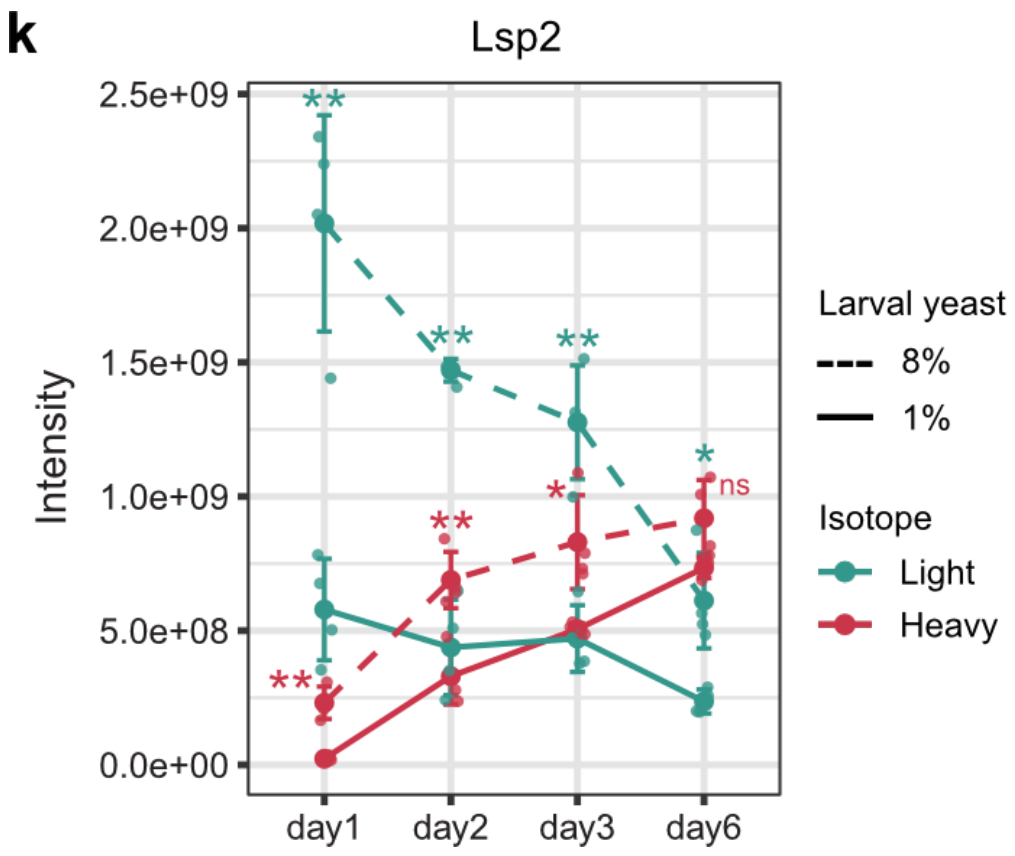

Fig. 3k, Lsp2 turnover revealed by time-course *in vivo* pSILAC analysis of ePR-flies.

Results section "ePR decreases both carry-over and synthesis of Lsp2 in early adulthood"

The combination of in vivo pSILAC and ePR further elucidated the Lsp2 protein turnover in early adulthood. In control flies, Lsp2 at day 1 is predominantly light-labelled (larva-derived), which is gradually replaced by heavy-labelled (adult-synthesised) Lsp2 over time (Fig. 3k, Supplementary Table 2). By day 6, the heavy-labelled fraction exceeds the light-labelled pool. As expected, light-labelled Lsp2 levels were significantly lower in ePR-flies across all time points (days 1, 2, 3, and 6), confirming a reduced carry-over of larval protein reserves. Notably, heavy-labeled Lsp2 was also significantly decreased in ePR-flies during the first three days of adulthood (Fig. 3k), indicating that ePR suppresses both the legacy of larval Lsp2 and its de novo synthesis in early adulthood.

10. The data in Figure 2J do not prove the point being made – they could simply indicate that over expression of Rheb in fat body is harmful. To reach the conclusion claimed, it should be shown that suppressing TORC1 in the fat body mimics the effect of juvenile restriction and is epistatic to it. The overall conclusion of the paragraph is not supported by the data – a causal connection between reduced protein synthesis and increased lifespan is not demonstrated.

Response 3-10. To better understand the role of early adult translation in lifespan extension, we restricted the activation of the mTORC1 pathway (by *Rheb* overexpression) only during the first week of adulthood. We found that, with the *Rheb* overexpression in the first week, ePR failed to extend lifespan (Extended Data Fig. 5a,b). However, we self-criticised that forced mTORC1 activation would impact many biological processes other than translation. Although the Kim/Pickering paper clearly suggested the connection between decreased early-adult translation and lifespan, we cannot fully conclude the causal link, due to the lack of genetic tools to specifically restore the decreased cytoRPs abundance and translational suppression. We added this explanation in the revised text honestly and therefore amended the manuscript title.

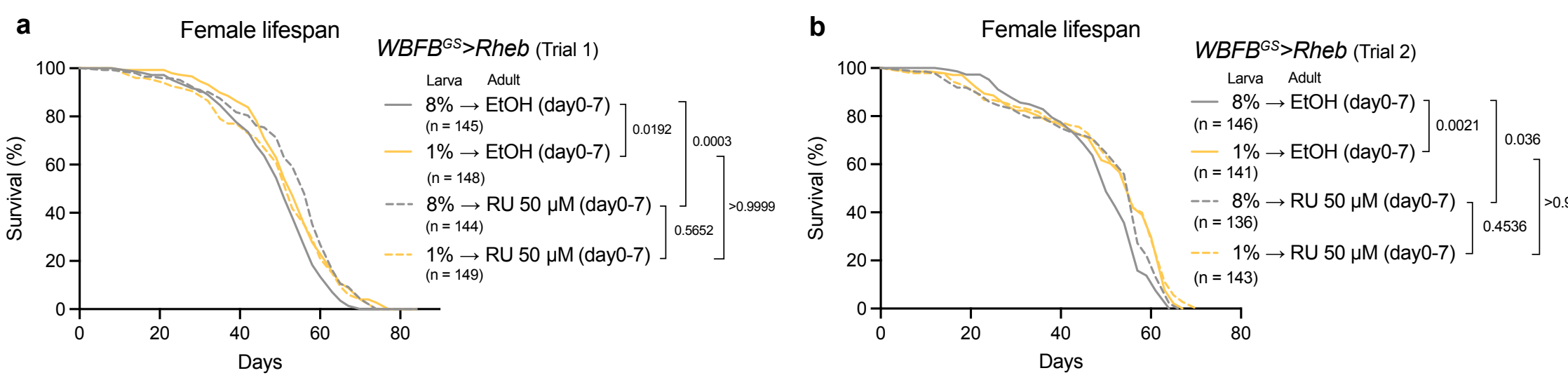

Extended Data Fig. 5a,b, Lifespan analysis of ePR flies with *Rheb* overexpression at day0-7.

Results section "ePR decreases ribosomal proteins and decreases translation in early adulthood":

To ask the functional significance of this translation suppression for the ePR-mediated lifespan extension, we attempted to enhance translation by overexpressing Rheb, a potent mTORC1 activator, in the adult fat body during the first week of adulthood. We found that ePR failed to extend lifespan when Rheb was overexpressed in the first week (Extended Data Fig. 5a,b), implying that lifespan extension by ePR is driven, at least partly, by suppressed translation.We acknowledge, however, that mTORC1 signaling does not exclusively stimulate protein synthesis but also regulates various processes. It is challenging to definitively prove the causal relationship between, cytoRPs, lower protein synthesis, and lifespan extension in ePR-flies without tools to specifically restore the entire cytoRP pool and to boost translation specifically. Nevertheless, given that previous studies connecting early adult translation and organismal lifespan[34], they are likely to be mechanistically interconnected.

11. Starting on line 241 the study moves to using RNA-seq to detect changes in gene expression in response to larval diet. There is a suggestion of a feed-forward mechanism, with loss of Lsp2 protein during late larval stages leading to transcriptional suppression of the Lsp2 gene in adults. What would be the function of such a feed-forward mechanism? This section of the MS is hard to follow and the logic is not clear. The idea that protein restriction in larvae lowers LSP2 protein levels and hence substrates for translation in early adulthood makes some sense but would this not be expected to lead to higher rates of LSP synthesis in well fed adults?

Response 3-11. We appreciate for pointing out this insight. We hypothesise that this feed-forward mechanism establishes a form of nutritional memory: Lsp2 abundance reflects protein availability and may set the anabolic rate for adulthood. This aligns with the concept of a predicted adaptive response, in which early-life nutrition calibrates later-life physiology. We revised the text to explain this more clearly.

Discussion:

Lsp2 functions as a central integrator of nutritional history that links dietary input to translational control. As a highly abundant storage protein with dynamic turnover in early-life stages, Lsp2 serves not only as an amino acid reservoir but also as a persistent molecular readout of nutritional conditions. Because Lsp2 levels are regulated through a feed-forward mechanism, its abundance reflects cumulative rather than transient dietary input, enabling sustained physiological responses to fluctuating environments. This property positions Lsp2 as a molecular substrate for predictive adaptive responses[50,51], effectively encoding "nutritional memory" across developmental transitions. Consistent with this idea, the amino acid composition of Lsp2 may confer selective buffering capacity; for example, adult flies are resistant to deprivation of Phe but no other essential amino acids, potentially reflecting the enrichment of Phe and Tyr in Lsp2[52].

12. Knockdown of Lsp in larval fat body did not mimic many of the effects of larval yeast deprivation on growth and female reproduction, bringing into question whether it operates through some of the same mechanisms as juvenile yeast restriction. The data in Figure 4j and k are worrying. No mention is made in the methods section of how mutant fly stocks were handled. If experimenters have backcrossed their mutants/transgenes into a standard background, as is essential, they normally say so, but there is no mention of this point. The data themselves are also unconvincing, with only partial epistasis, and they require both a repeat experiment and statistical analysis with Cox Proportional Hazards.

Response 3-12. We specified in the methods section that the lines used for lifespan analysis—*Cg-Gal4*, *r^4-Gal4*, *UAS-lacZ-RNAi*, and *UAS-Lsp2-RNAi*—were backcrossed eight times with w^{CS}. We repeated the experiment (Extended Fig. 10e). CoxPH analysis was also performed for the original Fig. 4j (now moved to Extended Data Fig. 9m), and the significant interaction between genotype (*Lsp2-*

RNAi) and larval diet (ePR) was confirmed (Supplementary Table 1). For the larval-specific *Lsp2* knockdown, as mentioned in Response 1-16, lifespan data using *pGnmt*^{GS} were also added (Extended Data Fig. 10j,k).

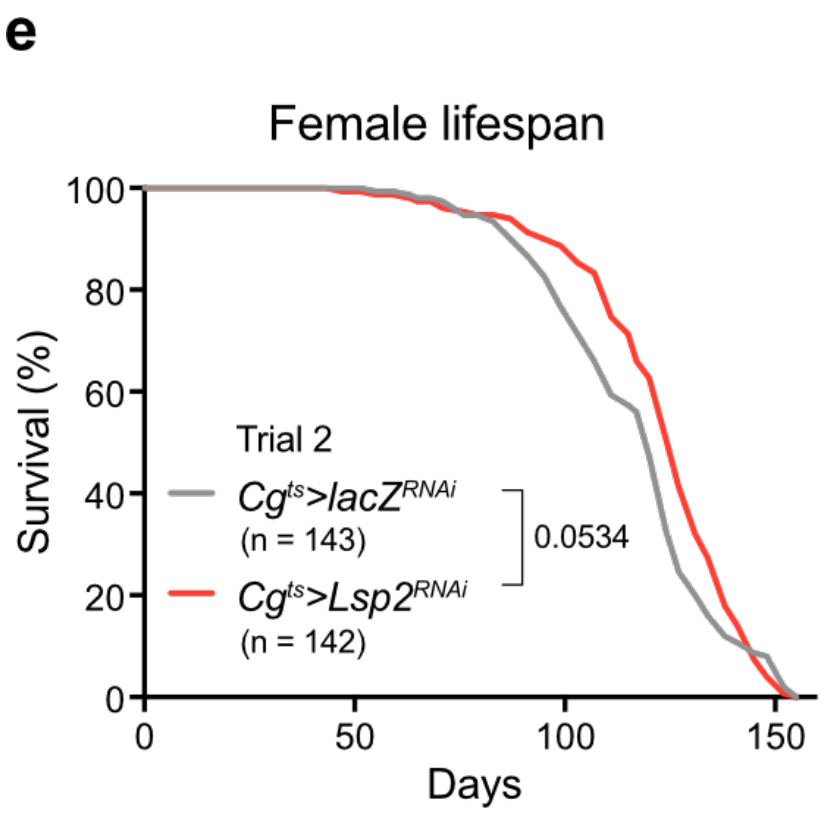

Extended Data Fig. 10e. Lifespan analysis of larva-specific Lsp2 RNAi using *Cg*^{ts} (2nd trial).

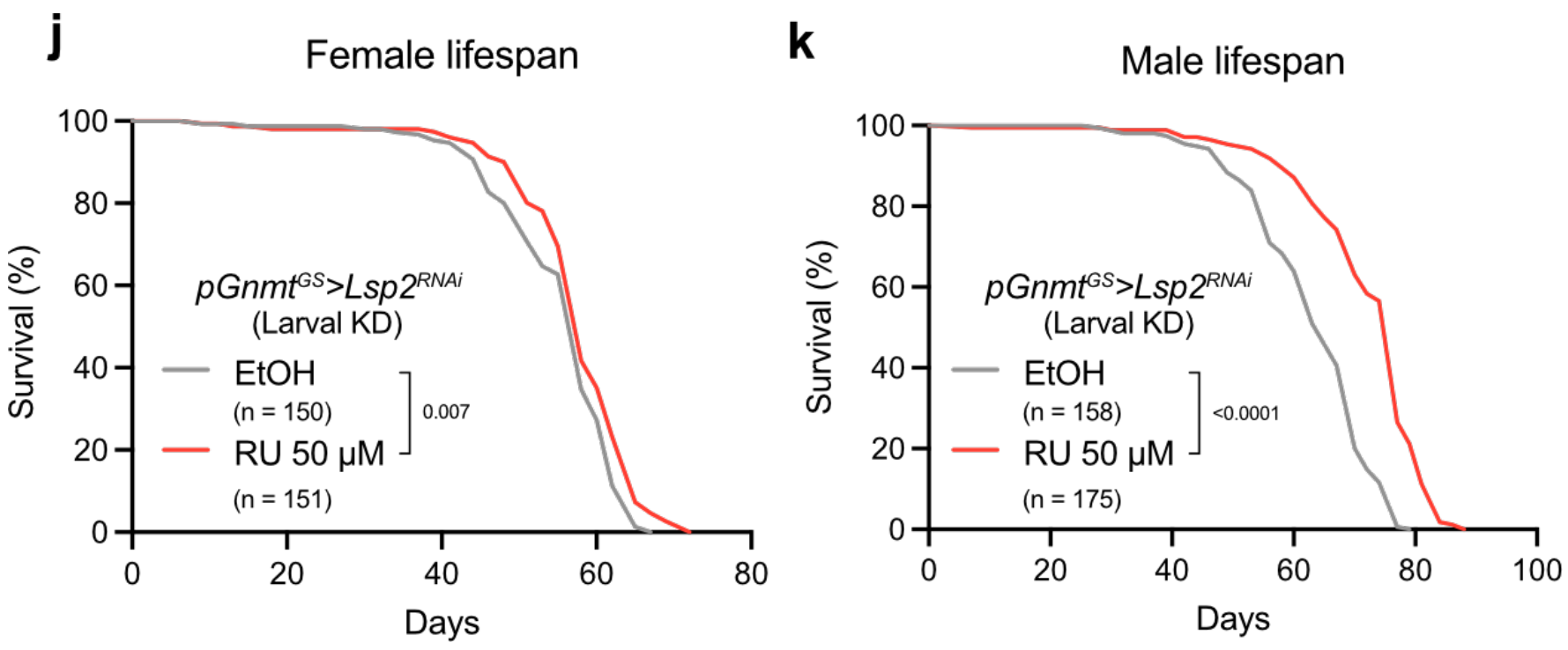

Extended Data Fig. 10h–k, Lifespan analysis of larval Lsp2-RNAi using *pGNMT*^{GS}.

Results section "Loss of Lsp2 suppresses protein translation and extends lifespan in adulthood"

Although Lsp2-RNAi and ePR share the mechanism for lifespan extension, our data did not demonstrate that early-life Lsp2 regulates lifespan. Using a temperature-sensitive Gal80 system, we tested whether the decrease in larva-derived Lsp2 was sufficient for reduced translation and lifespan extension. In Cg^{ts}*>Lsp2-RNAi flies, we found that both cytoRP levels and translation activity were reduced compared to control flies (Fig. 4i–l, Extended Data Fig. 10a–d, and Supplementary Table 3), suggesting the role of larva-derived Lsp2 in regulating early adult translation. Accordingly, Lsp2 knockdown restricted to the larval stage (Cg*^{ts}*>Lsp2-RNAi) was sufficient to extend female lifespan (Fig. 4m, Extended Data Fig. 10e). To exclude potential genetic background issues, we generated the pGnmt-GeneSwitch (pGnmt*^{GS}*) fly line by using the promoter sequence of the metabolic gene Gnmt, which is highly expressed in fat bodies*[48]*. Crossing this line with UAS-GFP confirmed that pGnmt*^{GS} *expression is almost entirely confined to adipose tissue and is RU486-inducible (Extended Data Fig. 10f,g). As expected, the knockdown of Lsp2 in larvae using pGnmt*^{GS} *decreased translation in female flies (Extended Data Fig. 10h,i) and extended the lifespan of both males and females (Extended Data Fig. 10j,k), albeit the effect was mild. It is possible that, since wandering larvae were not fed RU-containing food, the phenotype was not as strong as that achieved by Lsp2 knockdown throughout the larval to pupal stages. Nevertheless, these results prove that decreasing Lsp2 levels only in the larval stage suppresses protein translation and extends lifespan. However, considering that larval Lsp2 expression regulates adult Lsp2 expression through a feed-forward response (Fig. 3h–j), it remains possible that the Lsp2-RNAi in larvae decrease Lsp2 protein in early adulthood and exerts translational control.*

It is indeed interesting that not all the phenotypes of ePR depend on Lsp2. For example, the fact that

ePR is involved in ovariole number determination, whereas Lsp2 is not, may also reflect a temporal difference: Lsp2 is produced in large quantities and acts from late larval stages through the pupal stage, while the critical period of ovariole determination is during mid-late 3rd instar larva. Lsp2 is one of the key players in lifespan determination, but ePR may influence other developmental phenotypes as well.

13. The data on tyrosine restriction do not contribute to the analysis because of the disconnect between the effects of AAs and of yeast, mentioned above. It would require further analysis to determine if the mechanisms of tyrosine and yeast restriction are overlapping.

Response 3-13. We extended our larval-stage amino acid manipulation experiments, as outlined in Response 1-17 and 2-6, to clarify the impact of specific amino acids and yeast restriction (Fig. 5g–j). Since the complete restriction of essential amino acids prevents adult emergence and the synthetic diet used previously for Tyr deprivation produces semi-lethality, we utilised the larva-optimised HolFast diet to decrease essential amino acids, such as Phe, His, and isoleucine (Ile), to 25%. We confirmed that Tyr deprivation also extends lifespan in this experimental diet (Fig. 5g,h). Restriction of Lsp2-enriched amino acids, such as Phe and His, increased lifespan in both males and females (Fig. 5g,h). In contrast, restriction of Ile, which is less abundant in Lsp2, did not extend lifespan (Fig. 5g,h). Consistently, we found that the GFP fluorescence of Lsp2-muGFP decreased upon restriction of Phe or His, but not Ile (Fig. 5i). Interestingly, Ile restriction induced a stronger developmental delay than Phe or His restriction, suggesting that Lsp2 can act as a buffer to support larval development under nutritional stress by providing stored amino acids (Fig. 5j). We have added these data into the revised manuscript as shown below.

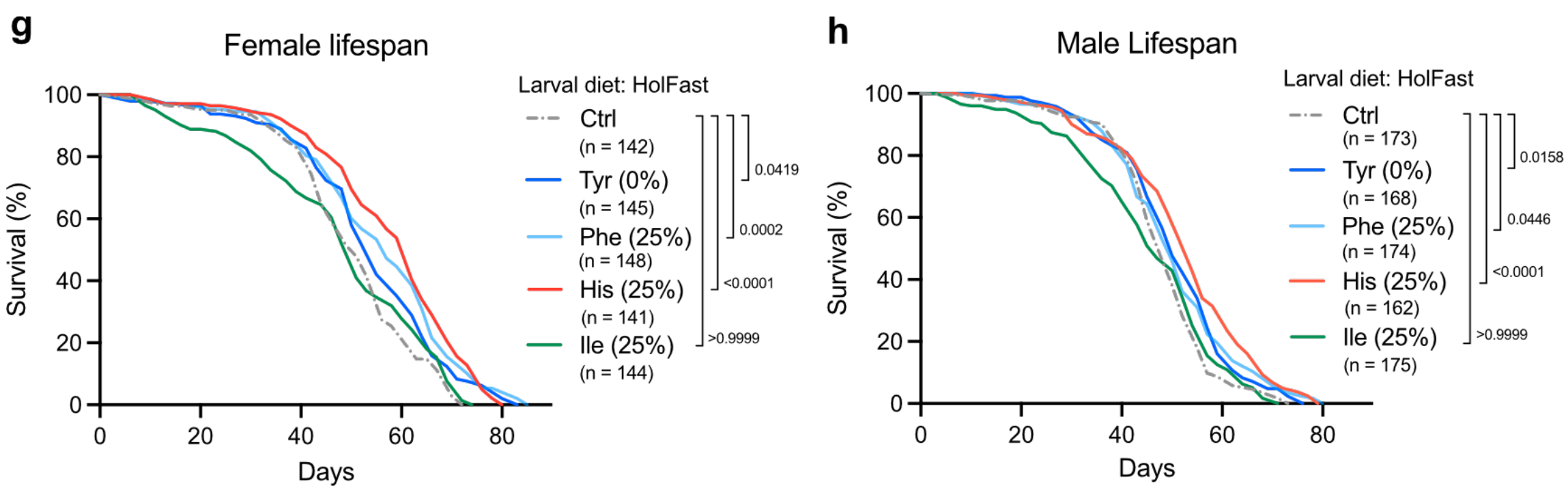

Fig. 5g,h, Lifespan analysis of the flies with larva-specific single amino acid restrictions.

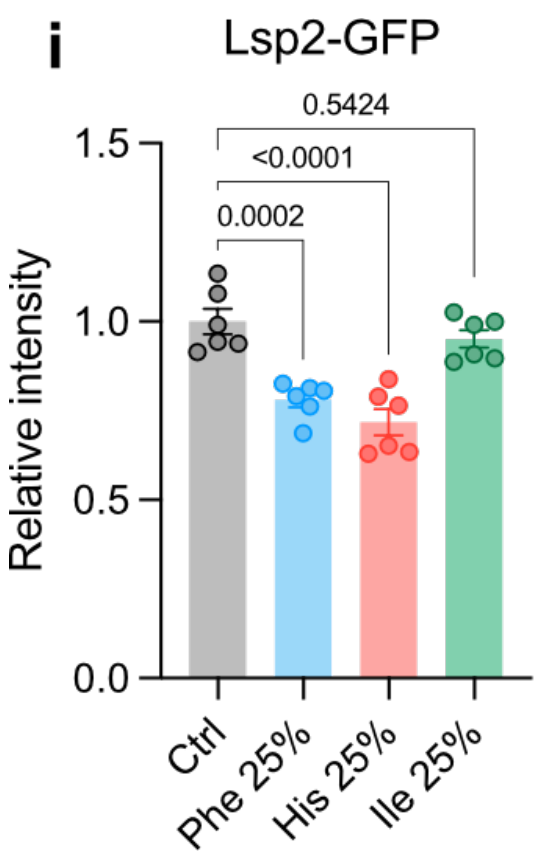

Fig. 5i, Lsp2-GFP quantification of the flies with larva-specific single amino acid restrictions.

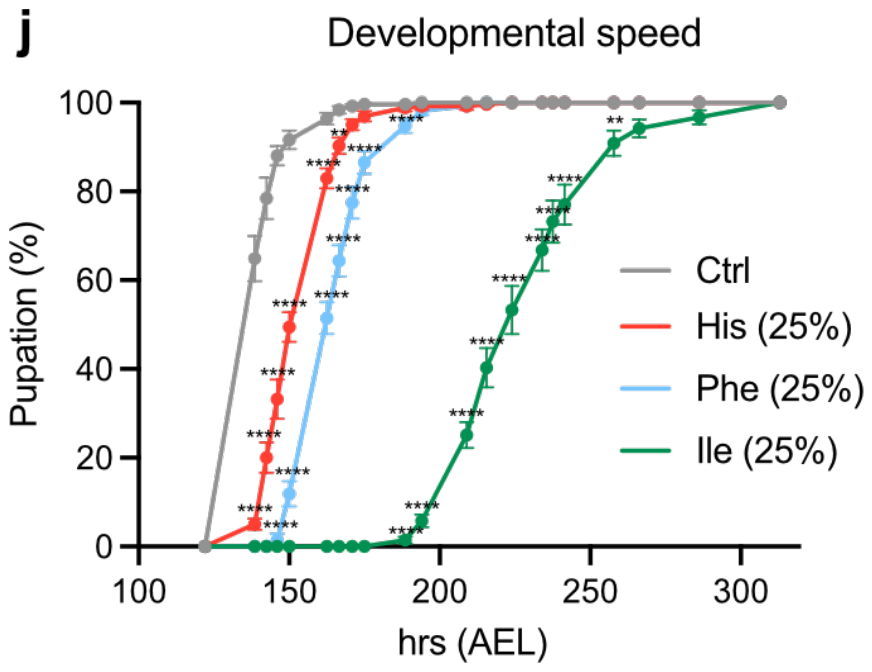

Fig. 5j, Developmental speed under single amino acid restrictions.

Results section "Single amino acid manipulation in the larval stage extends lifespan"

To test amino acid specificity, we restricted other amino acids. Since the complete restriction of essential amino acids prevents adult emergence and the synthetic diet used above for Tyr deprivation produces semi-lethality, we utilised the larva-optimised HolFast diet to decrease essential amino acids, such as Phe, His, and isoleucine (Ile), to 25%. We confirmed that Tyr deprivation also extends lifespan in this experimental diet (Fig. 5g,h). Restriction of Lsp2-enriched amino acids, such as Phe and His, increased lifespan in both males and females (Fig. 5g,h). In contrast, restriction of Ile, which is less abundant in Lsp2, did not extend lifespan (Fig. 5g,h). Consistently, we found that the GFP fluorescence of Lsp2-muGFP decreased upon restriction of Phe or His, but not Ile (Fig. 5i). Interestingly, Ile restriction induced a stronger developmental delay than Phe or His restriction, suggesting that Lsp2 can act as a buffer to support larval development under nutritional stress by providing stored amino acids (Fig. 5j). Taken together, these data suggest that the larval intake of dietary amino acids enriched in Lsp2 regulates the abundance of Lsp2 proteins in early life stages, which in turn modulates adult translational capacity and lifespan (Fig. 5k).

We hope that our revision has satisfactorily addressed the reviewer's concerns.

Referee #3 (Remarks to the Author):

The authors have made several beneficial modifications to their study. However, I remain unconvinced about some aspects of the nutritional manipulations.

The authors state that Interestingly, ePR-flies exhibit a further increase in lifespan when combined with adult protein restriction (4% vs 1% yeast extract [YE]) (Fig. 1q,r).' However, earlier work has suggested that yeast extract is toxic to flies, with increasing concentrations decreasing lifespan without any benefit to fecundity (Wong et al doi: 10.1093/gerona/62.10.1071). Furthermore, yeast extract is not equivalent to amino acids. Earlier in the text the authors show that they can manipulate amino acids directly, both in larvae and in adults, and they should stick to that method throughout the rest of the paper rather than introducing the use of yeast extract. The data in SF1 also do not address the point because they combine larval yeast with adult amino acids, rather than larval amino acids with adult amino acids. The issue of the interaction effect is also partially swept under the carpet by simply narrowing the range of concentrations analysed. We need to see the data for a full physiological range of concentrations of amino acids. More generally, since the authors have the relevant methodology available, they should manipulate amino acids directly rather than making conclusions about protein restriction based on manipulation of yeast. Mostly this has been done already, especially in the nice work on SILAC, but the use of yeast extract and the attempt to claim equivalence of yeast with protein without mechanistic evidence should be remedied.

Our Response: We thank the reviewer for raising a concern for the use of yeast extract (YE). First, we agree with the reviewer that yeast extract is somewhat toxic, so we are happy to remove the relevant data (Fig. 1q,r), which is not essential for our conclusion. Instead, we now frame the adult amino acid manipulation experiments (Extended Data Fig. 1) more cautiously as demonstrating that adult nutritional conditions can modulate the phenotype established during development. We note that these adult dietary manipulations were not intended to serve as a mechanistic dissection of protein versus other yeast-derived components, but rather as a physiological test of whether the phenotype remains responsive to changes in adult nutrition. We clarify this point in the revised manuscript.

Regarding the comment "*The issue of the interaction effect is also partially swept under the carpet by simply narrowing the range of concentrations analysed. We need to see the data for a full physiological range of concentrations of amino acids.*", we appreciate the reviewer’s concern. In our initial analysis, we focused on a physiological range of amino acid concentrations (10–100%), in part following prior

concerns raised by reviewer 1 to an accompanying paper (Wang et al.). When the full range of concentrations is included, we indeed observe significant interaction effects, and we now incorporate this full-range analysis into the revised manuscript. Importantly, the most extreme conditions (e.g., 0% or 300% amino acids) are associated with markedly reduced lifespan, likely reflecting toxicity or severe nutritional imbalance. When these extreme conditions are excluded, the interaction effect is substantially reduced, suggesting that the apparent interaction is largely driven by non-physiological conditions. We have clarified this point in the text and now present both analyses for transparency.

The reviewer wrote "*they should manipulate amino acids directly rather than making conclusions about protein restriction based on manipulation of yeast*" but we cannot agree with this. Our primary design for this study is to use a standard yeast diet (not yeast extract), which is considered to be a physiological diet in fruit fly studies. As the reviewer notes, and as shown in our manuscript, we have already demonstrated that modulation of amino acid levels during development is sufficient to recapitulate the effects (Fig. 1m–p, Fig. 5). Therefore, we believe that the key conceptual question—whether the developmental effect reflects protein/amino acid restriction—has already been directly addressed using defined nutritional manipulations. We do not believe that repeating all experiments using exclusively defined amino acid diets would meaningfully strengthen the conclusions of the study. Note that fully defined diets are not without limitations: there may be unintended consequences arising from the absence of complex dietary components existing in the natural food source. In contrast, yeast diets provide an important and complementary framework, as they represent a nutritionally complex and ecologically relevant food source for Drosophila. Accordingly, we consider the combined use of defined amino acid and yeast-based manipulations to be both appropriate and necessary, and we revise the manuscript to clarify the distinct scope and interpretation of these approaches.

Referee #1:

This revision by Koskamoto … Obata resolves my remarks on its initial submission. I commend the authors for the extensive new work to make this paper robust with strong, important inferences. They now clearly demonstrate how Lsp2 operates as a nutrient state mechanism to regulate adult survival through translation. I am impressed by the extensive, added survival trials where they resolve questions about when and where Lsp2 must be altered to slow adult aging. They have added time course 'pulse chase' isotope tracking data to understand when and where Lsp2 is produced and carried forward. The text throughout integrates enough of the precedent literature to put their novel findings in a compelling context.

There are now (or recently) several papers triangulating on Lsp2 and the control of translation as keystones to the arch of aging (in Drosophila). These discoveries can suggest analogous molecular physiology in mammals. The field will make a quantum leap in understanding human aging once the parallels are identified.

Response 1-1. We sincerely thank the reviewer for the highly positive and encouraging evaluation of our revised manuscript. We greatly appreciate the reviewer's recognition of the additional experiments and analyses performed in response to the initial comments. We are encouraged by the reviewer's thoughtful perspective on the broader implications of these findings for understanding conserved mechanisms of ageing. Thank you so much!

I offer a few points where text should be clarified.

Lines 161-168 can be tightened. I suggest, ... "It was found that larval-derived nitrogen is slowly replaced by adult-derived nitrogen provisioned to eggs and in somatic tissue when adult diet is restricted. These observations pose fundamental questions. How are specific larval proteins carried into the adult stage, and how do animals transition from using larval-derived amino acids to adult-acquired ones for the synthesis of specific proteins. To answer these questions requires analysis of proteome-wide protein dynamics."

Line 359. Clarify 'decreased'. I suggest … 'was also significantly less than control in …."

Line 375. Be more specific viz 'continuous'. I suggest … 'Lsp2 knockdown by RNAi though out larval and adult stages' …

Response 1-2. We appreciate the reviewer's helpful suggestions regarding text clarity and wording. We have revised the corresponding sections throughout the manuscript as proposed.

Lines 445-448. Please clarify. I don't understand what is being compared to what in this sentence.

Response 1-3. We revised the text to more clearly explain the lifespan comparisons shown in the figure and added the figure for median lifespan.

(Results) *Under control diet, fat-body-specific Lsp2 knockdown restricted to the first week of adulthood using WBFB-GeneSwitch (WBFBGS) significantly extended female lifespan (Fig. 4r; 8% EtOH vs. 8% 1week RU). Lifespan was also extended in males by early adult Lsp2 knockdown (Extended Data Fig. 9p). Adult-lifelong RU treatment increased lifespan similarly to 1 week RU (Fig. 4r; 8% EtOH vs. 8% RU). There was no statistical significance between adult-lifelong Lsp2 knockdown (8% RU) and early adult-specific Lsp2 knockdown (8% 1week RU), although median lifespan of "8% RU" (67 days) was greater than "8% 1week RU" (63 days) (Fig. 4r). Expectedly, ePR alone extended female lifespan in the absence of RU486 feeding (Fig. 4r; 8% EtOH vs. 1% EtOH), and adult-lifelong Lsp2 knockdown failed to extend lifespan further (Fig. 4r; 1% EtOH vs. 1% RU), which suggests that ePR extends lifespan via Lsp2. In contrast, when ePR was combined with early-adult-specific Lsp2 knockdown, lifespan was rather shortened (Fig. 4r; 1% EtOH vs. 1% 1week RU). We assume that the combination of ePR and transient suppression of Lsp2 during early adulthood renders flies susceptible to the life-shortening effects of Lsp2 that emerge later in life once Lsp2 expression is restored.*

Line 497. The author is Wang … not Zhai?

Response 1-4. We thank the reviewer for catching this error. We have corrected the author's name from "Zhai" to "Wang."

Referee #2:

The authors have addressed all my comments appropriately. The manuscript now robustly demonstrates that the level of Lsp2 controlled by larval diet is a main regulator of adult lifespan, a process that associates with translational control through regulating the levels of CytoRP.
See below additional points that the authors may want to clarify in the text.

Response 2-1. We thank the reviewer for the positive evaluation of our revised manuscript and for recognising the robustness of our findings. We are very much encouraged by the reviewer's comments during revision. We also appreciate the additional helpful comments provided below.

Ligne 288
I find the experiment with Rheb overexpression (ext.data.fig5) difficult to interpret. It could be deleted as it does not really address translation. What is the rationale for using a fat body driver? Also, Rheb OE seems to unexpectedly extend lifespan in fed animals. Could this be due to RU treatment? The experiment does not include controls for the effect of RU. This may also impact the interpretation of fig. 4r. I recommend that the authors clearly discuss what is comparable in these experiments, what is not, and why. In both figures, the lifespan differences are very mild and really hard to see. I suggest including a graph with the medium lifespans to simplify the presentation of the results for the reader.

Response 2-2. We thank the reviewer for these important comments and suggestions regarding the interpretation and presentation of the lifespan experiments. Following the reviewer's recommendation, we removed the *Rheb* overexpression experiment from Extended Data Fig. 5. The RU concentration (200 µM) used in the adult specific *Lsp2* knockdown experiment (Fig. 4r) is widely used in previous literatures (Bai et al., Aging Cell, 2012; Lu et al., Nature Aging, 2021; Kabil et al., PNAS, 2011) and our laboratory also empirically know that this dose of RU is not toxic to flies. For fig. 4r, we added more explanation to this analysis and a graph summarising median lifespan values to facilitate interpretation of the lifespan data.

(Results) *Under control diet, fat-body-specific Lsp2 knockdown restricted to the first week of adulthood using WBFB-GeneSwitch (WBFBGS) significantly extended female lifespan (Fig. 4r; 8% EtOH vs. 8% 1week RU). Lifespan was also extended in males by early adult Lsp2 knockdown (Extended Data Fig. 9p). Adult-lifelong RU treatment increased lifespan similarly to 1 week RU (Fig. 4r; 8% EtOH vs. 8% RU). There was no statistical significance between adult-lifelong Lsp2 knockdown (8% RU) and early adult-specific Lsp2 knockdown (8% 1week RU), although median lifespan of "8% RU" (67 days) was greater than "8% 1week RU" (63 days) (Fig. 4r). Expectedly,*

ePR alone extended female lifespan in the absence of RU486 feeding (Fig. 4r; 8% EtOH vs. 1% EtOH), and adult-lifelong Lsp2 knockdown failed to extend lifespan further (Fig. 4r; 1% EtOH vs. 1% RU), which suggests that ePR extends lifespan via Lsp2. In contrast, when ePR was combined with early-adult-specific Lsp2 knockdown, lifespan was rather shortened (Fig. 4r; 1% EtOH vs. 1% 1week RU). We assume that the combination of ePR and transient suppression of Lsp2 during early adulthood renders flies susceptible to the life-shortening effects of Lsp2 that emerge later in life once Lsp2 expression is restored.

Ligne 480: Interestingly, Ile restriction induced a stronger developmental delay than Phe or His restriction, suggesting that Lsp2 can act as a buffer to support larval development under nutritional stress by providing stored amino acids (Fig. 5j).
It would be best to include an LSp2 KD in this experiment to support this hypothesis.
Response 2-3. We appreciate the reviewer for this critical comment. We have examined developmental timing following *Lsp2* knockdown using the fat body driver *Cg-Gal4*. Overall, *Lsp2* knockdown did not impact the phenotype as we expected. It slightly accelerated development under His or Ile restriction (Fig. R1). We have therefore revised the manuscript to weaken the corresponding statement, as shown below.

(Fig. R1)

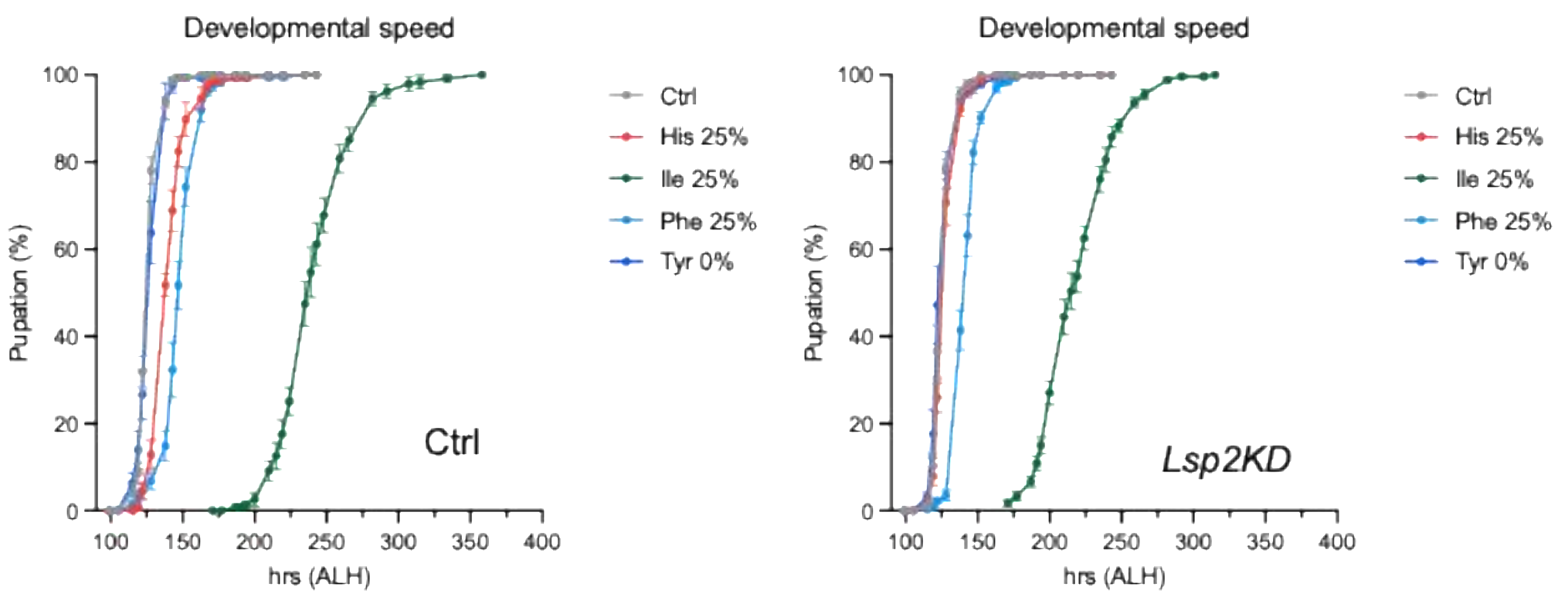

(Result) *Ile restriction induced a stronger developmental delay than Phe or His restriction, potentially compromising health later in life (Fig. 5i).*

Ligne 177: diet containing "medium" isotope-labelled Lys (+4 Lys; D4)
Shouldn’t D4 be replaced by 13C4?

Response 2-4. In this experiment, we used deuterium-labelled lysine (+4 Lys; D_4) rather than $^{13}C_4$-labelled lysine. This is because $^{13}C_4$ Lys is not commercially accessible and pSILAC experiments historically utilize D_4 Lys.

Ligne 281: The suppressive effect of ePR on translation lasted only one week (Fig. 2o), consistent with the fact that decreased abundance of cytoRPs by ePR can be rescued in this time frame.
I'm not sure I understand this sentence. Which figure is referred to for the rescue

Response 2-5. We have added the corresponding figure reference "(Fig. 2k)" to clarify the statement.

Referee #3:

The authors have made several beneficial modifications to their study. However, I remain unconvinced about some aspects of the nutritional manipulations.

The authors state that Interestingly, ePR-flies exhibit a further increase in lifespan when combined with adult protein restriction (4% vs 1% yeast extract [YE]) (Fig. 1q,r).; However, earlier work has suggested that yeast extract is toxic to flies, with increasing concentrations decreasing lifespan without any benefit to fecundity (Wong et al doi: 10.1093/gerona/62.10.1071). Furthermore, yeast extract is not equivalent to amino acids. Earlier in the text the authors show that they can manipulate amino acids directly, both in larvae and in adults, and they should stick to that method throughout the rest of the paper rather than introducing the use of yeast extract. The data in SF1 also do not address the point because they combine larval yeast with adult amino acids, rather than larval amino acids with adult amino acids.

Response 3-1. We thank the reviewer for raising concerns regarding the manipulation of dietary yeast and yeast extract (YE), and their relationship to amino acid restriction. Firstly, we agree with the reviewer that YE is somewhat toxic to flies. To address this concern, we removed the corresponding YE-based lifespan data.

Regarding SF1, we now present the adult amino acid manipulations more cautiously as physiological tests examining whether the developmental phenotype remains responsive to changes in adult nutritional conditions (Fig. 1o-r). We have avoided mechanistic interpretations based on these experiments and clarified this point in the revised manuscript.

The issue of the interaction effect is also partially swept under the carpet by simply narrowing the range of concentrations analysed. We need to see the data for a full physiological range of concentrations of amino acids.

Response 3-2. We appreciate the reviewer's suggestion to examine the full range of amino acid concentrations. In the original analysis, we focused primarily on intermediate concentrations (10–100%), based on previous considerations regarding physiological relevance. Following the reviewer's suggestion, we have now included analyses across all concentration range tested. Although significant interaction effects are observed in female flies, these effects are largely driven by extreme dietary conditions (e.g., 0% or 300% amino acids), which substantially shorten lifespan (median lifespan <40 days) and likely reflect severe nutritional imbalance or toxicity that limits lifespan regardless of larval dietary condition. When these extreme conditions are excluded, the interaction effect is no longer significant. For transparency we now present both analyses in the revised manuscript and clarify this point in the text.

More generally, since the authors have the relevant methodology available, they should manipulate amino acids directly rather than making conclusions about protein restriction based on manipulation of yeast. Mostly this has been done already, especially in the nice work on SILAC, but the use of yeast extract and the attempt to claim equivalence of yeast with protein without mechanistic evidence should be remedied.

Response 3-3. We appreciate that chemically-defined synthetic diets provide the straightforward approach for examining the role of amino acid availability. We would like to mention that the synthetic diets have limitations, including potential secondary physiological effects arising from the absence of complex nutritional components normally present in natural food sources. Indeed, although much better than the previous versions, HolFast still induces a developmental delay (Sorge et al., Dev Cell, 2025). In contrast, yeast-based diets provide a nutritionally complex and ecologically relevant dietary context for *Drosophila*. Based on this philosophy, we used yeast manipulations throughout the study as a primary tool, with key aspects confirmed by synthetic diets. We have carefully revised the manuscript not to mix up yeast and amino acid manipulations and to discuss what has been shown in each experiment honestly.

Importantly, we have shown that adding back amino acids into the low-yeast diet abolishes the lifespan extension (Fig. 1m,n), demonstrating that amino acids are key nutrients mediating ePR's longevity. This conclusion is supported by larval amino acid restriction, using the synthetic diets, which decreases Lsp2 protein, early-adult translation and extends lifespan (Extended Data Fig. 1, and Fig. 5). These results clearly show that developmental amino acid availability modulates Lsp2-dependent regulation of longevity. In the accompanying manuscript by Wang et al., they also show how dietary amino acids regulate Lsp2 in molecular level. Together, we believe our study provides sufficient evidence to claim our conclusions.

Referees' comments:

Referee #1 (Remarks to the Author):

The new revision refines and elevates both the data and coherent story of the project. Light editing is needed in a few spots.

Response 1-0: We thank the reviewer for the positive assessment of our revised manuscript and for the helpful suggestions.

Line 112: seems to be missing some words.

Response 1-1: Thank you for pointing this out. We have amended the sentence as follows:

To determine whether this longevity effect depends on adult dietary context, we examined the lifespan of ePR-flies across a range of adult dietary amino acid concentrations. ePR-flies exhibited lifespan extension throughout a broad physiological range of amino acid concentrations (10–100%), whereas this effect was abolished under extreme dietary conditions (0% or 300% amino acids) (Fig. 1o–r).

Line 398: most readers will not grasp the logic for why 'not demonstrate'. It is because the ts system can't disentangle genotype and temperature affects. Somehow make the issue clearer.

Response 1-2: Thank you for this helpful suggestion. Our original statement was intended to convey that, although whole-life Lsp2 knockdown extends lifespan, our current data do not establish whether early-life Lsp2 alone is sufficient to regulate lifespan. To clarify this point, we have revised the sentence as follows:

Although Lsp2 knockdown and ePR are likely to share the mechanism for lifespan extension, whether early-life Lsp2 alone is sufficient to regulate lifespan remains unclear.

Line 410: 'the effect was mild'. The quantity of change should be made explicit. This is a central result, a bit buried in the extended data. If the result is 'mild', show us how much so, warts and all.

Response1-3: Thank you for this suggestion. We have revised the text to explicitly indicate the extent of translational suppression:

As expected, the knockdown of Lsp2 in larvae using pGnmt^{GS} decreased translation in female flies (Extended Data Fig. 9h,i) and extended the lifespan of both males and females (Extended Data Fig. 9j,k), despite a weaker suppression of translation than with Cg^{ts} driver (70% versus 47% of control).

Referee #2 (Remarks to the Author):

The authors have addressed all my comments appropriately, I do not have additional remarks, the study is ready for publication in my opinion.

Response2: We sincerely thank the reviewer for the positive evaluation of our work and for the constructive comments throughout the review process.

Referee #3 (Remarks to the Author):

I am satisfied with the latest round of revisions

Response 3: We sincerely thank the reviewer for the positive evaluation of our work and for the constructive comments throughout the review process.
